# AI-powered Immune Cell Knowledge Graph (ICKG) with granular immune contexts enables immune program interpretation

**DOI:** 10.1038/s44387-025-00060-4

**Published:** 2026-01-27

**Authors:** Shan He, Yukun Tan, Qing Ye, Matthew M Gubin, Merve Dede, Hind Rafei, Weiyi Peng, Katayoun Rezvani, Vakul Mohanty, Ken Chen

**Affiliations:** 1 Department of Bioinformatics and Computational Biology, The University of Texas MD Anderson Cancer Center, Houston, TX USA; 2 Department of Immunology, The University of Texas MD Anderson Cancer Center, Houston, TX 77030 USA; 3 Department of Stem Cell Transplantation and Cellular Therapy, The University of Texas MD Anderson Cancer Center, Houston, TX USA; 4 Department of Biology and Biochemistry, The University of Houston, Houston, TX USA

**Keywords:** Complex networks, Computer science, Computational science, Computer science, Computational models, Computational platforms and environments

## Abstract

The widespread application of single-cell and spatial omics to models and patient samples has transformed immune cell profiling across physiological conditions. However, knowledge of immune cell states, functions, and gene regulation remains fragmented across publications, limiting our ability to synthesize insights and derive mechanistic understanding from the literature. To address this gap and facilitate literature integration, we constructed Immune Cell Knowledge Graphs (ICKGs)—four cell type-specific graphs derived from over 24,000 cancer immunotherapy-focused PubMed abstracts using large language models (LLMs) with “human verifiable” validation. Unlike conventional databases, which provide context-agnostic pathways, ICKGs capture directed, literature-supported relationships among genes, pathways and immune functions, enabling context-aware reasoning. We validated ICKGs using perturbation datasets from cytokine stimulation and CRISPR experiments, demonstrating that ICKGs contain more accurate and immunologically coherent contexts than canonical databases. As a key application, ICKGs provide interpretable and accurate pathway annotations, including signatures unannotated by canonical databases or used in immuno-oncology. To support community use, we created an interactive portal (https://kchen-lab.github.io/immune-knowledgegraph.github.io/) to perform ICKG-based pathway annotations, allowing researchers to explore immune cell-specific insights grounded in literature. This work establishes ICKGs as a scalable framework for immune-specific functional interpretation and mechanistic hypothesis generation in single-cell and spatial omics.

## Introduction

Immune cells play a central role in human health and disease. In cancer, the interaction between immune and tumor cells influences tumor progression, therapeutic response and overall patient outcomes^1–3^. The functional behavior of these immune cells is governed by complex, cell-type-specific transcriptional programs that shape their roles within the tumor microenvironment (TME). To improve therapeutic strategies and advance immunological understanding, it is imperative to develop systematic approaches that can integrate existing knowledge and deliver a precise, comprehensive understanding of immune cell function across diverse contexts.

Advancing our understanding of complex immune ecosystems requires not only high-resolution data but also the integration of knowledge that connects and interprets those data meaningfully. The rapid growth of single-cell and spatial omics technologies applied to human specimens has revealed unprecedented complexity and heterogeneity in immune signaling, cell-to-cell interactions, and regulatory patterns in human tissues and disease states. In cancer immunology, for example, recent pan-cancer cell atlases have delineated cell differentiation trajectories, identified condition-specific immune cell types and states, and uncovered molecular drivers of cell fate and function^4–9^. However, these insights remain scattered across individual studies, each constrained by specific experimental conditions and analytical frameworks. Without integration, this fragmented knowledge limits our ability to generalize findings and extract broader mechanistic insights. By synthesizing dispersed findings, effective literature integration can contextualize gene set interpretation, enable mechanistic reasoning grounded in repetitive experimental evidence, discovery of functional relationships between genes and immune processes, and systematic hypothesis generation for novel emergent targets.

One of the most tangible benefits of literature integration is the potential to improve current methods for immune gene set annotation, as integration allows for context-specific and mechanistically grounded interpretations that extend far beyond what current pathway databases can offer. For the most part, the gold standard for gene set annotation has been over-representation analysis (ORA) against well-established pathway databases. Currently, numerous curated resources have been developed for context-agnostic annotation of gene signatures. For example, RNA profiling of bulk specimens under immune perturbations has been used to catalog immune-related gene sets such as those in MSigDB C7^10^. More broadly, functional annotation databases such as Hallmarks and KEGG have become widely adopted for enrichment analysis. While these resources are valuable, they lack the granularity and context specificity needed to interpret complex gene signatures derived from single-cell and spatial omics studies. Annotations obtained through these databases are often generic, lack cell type context specificity and are biased towards well-characterized pathways (e.g., metabolism)^10–12^ and poorly suited to capturing the diversity of immune cell states. This limited capacity is a major bottleneck in data-driven discovery in immunology. Consequently, researchers are often left to interpret novel gene sets through ad hoc reasoning or manual literature review, leading to annotations that are incomplete, subjective and difficult to reproduce. In contrast, the development of large language models (LLMs) has prompted new approaches that move beyond traditional ORA, with several studies attempting to apply LLMs directly for gene set annotation or biological summarization^13–16^. Although LLMs are trained on vast biomedical text and, in theory, can broadly encode extensive experimentally validated knowledge of gene function, regulation, and immune processes expressed in context-specific, biologically meaningful ways. Specifically, Hu et al.^14^ applied flagship LLMs for gene set summarization. Boyce et al.^16^ sought to enhance LLM prompts with ORA-derived annotations. Zhu et al.^13^ explored generating new gene sets from query sets and reanalyzing them with ORA. Chen et al.^15^ provided NCBI gene summaries as input, which yielded useful individual gene descriptions. While these approaches open exciting avenues for scalable and context-aware annotation, they potentially suffer from the intrinsic limitation of LLM, particularly hallucination and lack of accountability, which calls for additional systematic frameworks that may better balance automation with interpretability and reliability.

To overcome the above-mentioned limitations, we constructed Immune Cell Knowledge Graphs (ICKGs) by systematically extracting and organizing information from over 24,000 PubMed-indexed abstracts using a combination of AI techniques, including LLMs. Since immunology is highly context dependent, each ICKG is tailored to a major human immune cell type and contains structured entities such as genes, protein complexes, diseases, cell types, pathways, and immunologically relevant biomedical concepts, as well as directional relationships such as activation and inhibition. All edges are traceable to the source publications, enabling transparent reasoning and “human verifiable” verification. These knowledge graphs serve as a foundation for knowledge graph reasoning (KGR)^17–20^, which can uncover hidden immunological insights, prioritize downstream effectors and annotate complex gene sets. We rigorously validated each step of ICKG construction and evaluated the breadth and fidelity of their encoded immune contexts using independent, cell-type-specific, functional perturbation datasets. Our results show that ICKGs capture more granular and functionally relevant immune contexts than canonical pathway databases. Furthermore, performing KGR over ICKGs can enhance gene set interpretation in a way that reflects the rich and mechanistically specific language of the immunology literature, offering a powerful new approach that can substantially advance knowledge acquisition, interpretation, and hypothesis generation in immune-oncology research.

## Results

### Construction of ICKGs to represent the immune system’s complexity

**Fig. 1:**
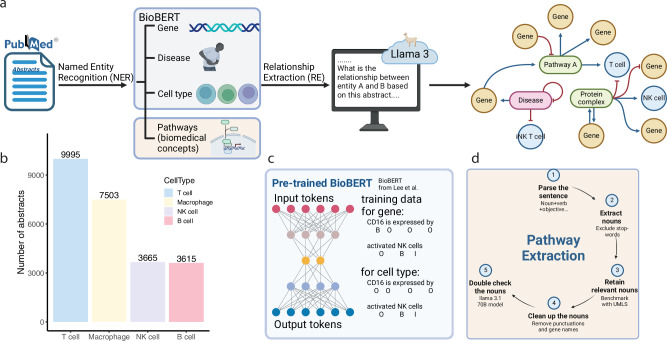
Schematic for immune cell knowledge graph construction. **a** Overall workflow: abstracts download based on keywords. Named entity recognition (NER) based on a finetuned BioBERT model. Relationship extraction based on prompt engineering. Network assembly based on relations (nodes are colored based on node types, and edges are colored based on the relationship). **b** Number of abstracts extracted for each immune cell type of interest. **c** BioBERT methodology demonstration. **d** Step-by-step explanation for pathway extractions from abstracts.

The overall schematic for ICKG construction follows Fig. 1a. To enhance context-specificity and achieve cellular granularity, we constructed ICKGs for specific cell types, including T cells (including CD4/CD8^+^ T cells and regulatory T cells), natural killer (NK) cells, B cells and macrophages. To obtain the most up-to-date publications on immune cells in the setting of cancer, we searched PubMed and retained abstracts published between 2020 and 2024 using “cell type” and “cancer immunotherapy” as the keywords (Fig. 1b). It is important to note that the retrieved abstracts encompass a mix of study types and conclusions derived from diverse data modalities -including genetics, transcriptomic, experimental, and review-based studies. Among these publications, genes, diseases, and cell types are important entities to be extracted from abstracts, as they provide both the molecular and functional basis to characterize immune cells. To this end, we finetuned the respective named-entity-recognition (NER) tasks using a pretrained BioBERT model with corresponding training datasets and selected the best one based on the highest precision (Fig. 1c, see “Methods”). Ultimately, BioBERT models finetuned on BioNLP13PC^21^, NCBI^22^, and GENIA were selected to perform the NER task for gene, disease, and cell type recognition, respectively, with a precision of 87.87%, 85.23%, and 61.77% (Table 1).

**Table 1:** Performance evaluation for named entity recognition (NER) across different entity types and training datasets

Training data	No. of words	Precision	Recall	F1 Score
Gene name NER				
BC2GM^39^	148,529	82.85	84.21	83.52
JNLPBA	118,582	70.78	83.07	76.43
BioNLP13PC*	47,896	**87.87**	**90.09**	**88.97**
Disease name NER				
BC5CDR^40^	129,562	85.00	87.07	86.02
NCBI*	25,438	**85.23**	**88.96**	**87.05**
Cell type name NER				
GENIA*	50,031	61.77	73.42	67.09
Pathways/Biomedical phrases NER				
GENIA	50,031	52.63	60.58	56.33
Rule-based + prompt*	48,222	**72.81**	**80.64**	**76.53**

The bold values correspond to the highest performance.

Pathways (or biological concepts) are also important entities that need to be extracted from abstracts, as they provide the basis for new vocabularies that we can use to annotate a gene set. However, definitions of pathways are vague, challenging our entity extraction approach. In addition, since data with pre-labeled pathway terms is rare, with only GENIA providing limited pathway labels, the ground truth is lacking to validate any pathway extraction approach. To resolve these issues, we established a lenient but effective definition of “pathways” as any informative biomedical noun phrase in an abstract. Since abstracts are usually written in succinct sentences, we designed a rule-based approach based on the grammatical structure of each sentence to extract biomedical phrases, followed by prompt engineering to retain the biomedically relevant nouns (Fig. 1d, see “Methods”). We also manually annotated a database with 48,000 words based on our definition and found that this combined method was able to extract concise and informative biomedical phrases from abstracts with a precision of 72.81% (Table 1).

A variety of pathway databases have been constructed and widely applied for gene set annotation purposes^10,12^. However, these resources capture only a limited range of relationships across biological concepts—often restricted to undirected gene–gene links—and tend to encode context-agnostic interactions. Because they are largely derived from cell lines and bulk-tissue data, they lack the resolution needed to delineate immune cell-type-specific relationships. By contrast, advances in single-cell technologies, particularly when applied to patient tissue samples, have reported a wealth of immune cell-type-specific knowledge, including novel relationships across levels of biomedical concepts, that are yet to be integrated. To robustly capture this new information, we defined the relationships between entities using two biologically interpretable properties—activation and inhibition—and prompted the LLM to infer these relationships directly from the abstracts from which the entities were extracted, leveraging the power of the LLM in understanding the semantic context and establishing directionalities.

We employed a zero-shot prompt engineering approach using the open-source Llama 3.1 model (70B parameters) with a carefully designed prompt (see “Methods”). We requested the model to infer the directional relationship (i.e., activation, inhibition, or no association) between pairwise entities based only on the abstract. To validate the authenticity of the constructed edges, we evaluated Relationship Extraction (RE) performance by benchmarking our inferred relationships against well-established relational databases: KEGG^12^ for gene–gene and Immune Cell Atlas^7^ for gene-cell type, respectively. For gene–gene relationships, we test if ICKG can capture KEGG-defined activation and inhibition interactions between gene pairs. For gene-cell type relationships, we test if the genes enriched in major immune cell types are captured by ICKG as genes that directly activate the cell type node. We extracted 58,456 pairwise directional relationships from 361 KEGG pathways and simplified the relationships into either activation or inhibition based on interaction types (see “Methods”). The Immune Cell Atlas was profiled from more than 300,000 individual immune cells extracted from 16 different healthy tissues, and we compared our gene-cell type relationship with the cell type DEGs from this atlas (see “Methods”). Table 2 includes a summarized performance across the different RE tasks, with precision ranging from 77.18% to 87.66%.

**Table 2:** Performance evaluation for relationship extraction for gene–gene and gene-cell type across the different immune cell-specific knowledge graphs

Cell type	Precision	Recall	F1 Score
Gene-Gene relationship extraction			
T cell	83.08%	95.16%	88.70%
B cell	85.65%	93.43%	89.40%
Macrophage	80.57%	90.36%	85.19%
NK cell	84.47%	96.80%	90.22%
Gene-Cell type relationship extraction			
T cell	77.18%	75.43%	76.29%
B cell	81.08%	86.77%	83.83%
Macrophage	82.67%	84.26%	83.46%
NK cell	87.66%	82.79%	85.16%

**Fig. 2:**
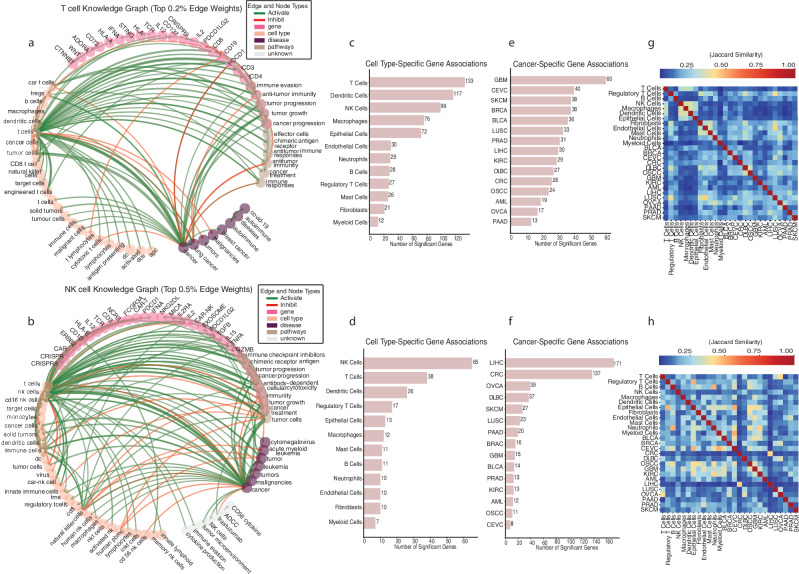
Overview of the cell type specific knowledge graph. **a**, **b** T and NK cell-specific knowledge graph subset containing only edges with top 0.5% weight. Nodes are colored based on node types (gene, disease, cell type, pathways and others). Edges are colored based on activation or inhibition between the two connecting nodes. The thickness of the edges represents the number of published abstracts that support such a relationship (**c**, **d**). Number of significantly associated genes with each major cell type derived from the T and NK cell-specific knowledge graph (**e**, **f**). Number of significantly associated genes with each major TCGA cancer type according to PageRank scores derived from T- and NK-cell-specific knowledge graphs. **g**, **h** Pairwise Jaccard distance (measure for the number of overlapping genes) across cancer types and cell types derived respectively from T- and NK-cell-specific knowledge graphs.

The constructed knowledge graphs have nodes of various types (i.e., gene, disease, cell type, and biomedical concepts) and edges of either activating or inhibiting relationships with weights representing the number of abstracts that mention such relationships. The thickness of the edges represents the number of PubMed publications that were identified to support the relationship and is manually verifiable to ensure accuracy and verifiability. Table 3 summarizes basic node and edge information for the constructed knowledge graphs. For visualization purposes, we have presented the core section of the T and the NK cell graphs with thick edges, representing relationships with more literature supports, in Fig. 2a and Fig. 2b, respectively. Subgraphs for the B cells and the macrophages are presented in Fig. 2a, b.

**Table 3:** Basic information for the constructed knowledge graphs

Cell types	No. of nodes	No. of edges	No. of genes	No. of diseases	No. of cell types	No. of pathways	No. of others
NK cell	6001	24,901 (total) 17,729 (activate) 7172 (inhibit)	1608	330	1128	1083	1852
T cell	11,981	52,454 38,214 (activate) 14,240 (inhibit)	3268	721	1925	2445	3622
B cell	4810	16,722 13,022 (activate) 3700 (inhibit)	1368	439	827	904	1272
Macrophage	7763	21,624 16,061 (activate) 5563 (inhibit)	1925	480	1051	1517	2790

Our graphs are more complex than currently available pathway databases in terms of the breadth of relationships they capture—linking genes, cell types, diseases, and pathways. For reference, KEGG contains 19,583 edges, whereas our ICKGs capture 115,701 cell-type specific edges: 24,901, 52,454, 16,722, and 21,624 respectively in the NK, T, B, and macrophage graphs, respectively. Most of these edges originate from recent publications of cell-type-specific immunological findings that are not represented in KEGG. By leveraging these cell-type-resolved graphs—each characterized by richer connectivity—they enable forms of knowledge querying and reasoning that existing databases cannot support. One way to perform such querying/reasoning is to use PageRank, an algorithm that assigns a numerical weight to each node in a graph based on both the structure of its incoming and outgoing edges and the weights associated with these edges, effectively measuring the relative importance of each node within the network. This capacity to resolve functional relationships at the cell-type level is exemplified by IL-6, which exerts opposite effects in T cells versus NK cells^23–25^: in T cells, IL-6 drives inflammatory differentiation by promoting Th17 development while blocking FOXP3⁺ Treg induction, whereas in NK cells, IL-6 suppresses cytotoxicity by downregulating perforin and granzyme B. Traversing the T cell versus NK cell ICKGs, we identified differential downstream associations consistent with these roles: in T cells, IL-6 induction was coupled to STAT3, IL17, IL12, and IFNG, alongside checkpoint and activation markers (PDCD1, PDCD1LG2, CD8, HLA), reflecting a pro-inflammatory, Th17-skewed response with sustained effector activity (Figure S1a). In contrast, in NK cells, IL-6 was linked to TGFB, IL10, IL4, CCL2, ARG1, VEGFA, and PDCD1LG2, a network profile aligning with functional suppression, metabolic reprogramming, and tolerogenic signaling (Fig. S1b).

The ICKGs also reflect the bibliometric landscape of cancer immunology research over the past five years, capturing cell-type-specific contexts and highlighting how different communities of literature emphasize distinct genes and associations. Firstly, by comparing the centrality measure of each gene across the four ICKGs, we revealed differential sets of genes being prioritized as important depending on the cell type under study (Fig. S1c). Secondly, we can effectively quantify the number of genes reported in association with specific concepts (e.g., cell type or cancer type), providing a measure of the relative emphasis placed on each concept in the literature. We leveraged the PageRank algorithm and extracted genes that are significantly associated with major cell types and TCGA cancer subtypes in respective cell type graphs (Fig. 2c–f, Fig. S2c–f, see “Methods”). As expected, we found that more genes are reported to be associated with T cells in the T cell graph, and more genes are reported to be associated with NK cells in the NK cell graph. Interestingly, cancer types are associated with variable numbers of genes in the T cell graph vs those in the NK cell graph. For example, more genes are reported to be associated with Glioblastoma (GBM) in the T cell graph, whereas in the NK cell graphs, more genes are reported to be associated with Liver Hepatocellular Carcinoma (LIHC). By analyzing these gene associations, we gain insight into the research landscape—identifying which genes researchers are studying in relation to various immune cell types and cancer types. Thirdly, we can quantify the similarity between concepts based on the overlap in genes studied in association with them, which provides a structured view of the current research landscape, pointing to frequently co-studied concepts, revealing underexplored areas, and enabling hypothesis generation. Figure 2g, h and Figure S2g, h summarize disease and cell type similarities based on the number of shared genes to be reported in association with that concept in the corresponding graphs, highlighting both genetic commonality and their relative proximity within the current research landscape in terms of the genes most frequently studied.

### Cell-type-specific ICKGs contain granular immune contexts and enable biological reasoning

With the construction of immune-cell-specific knowledge graphs, we sought to evaluate their capacity for biological reasoning; specifically, their ability to infer and predict transcriptional consequences of immunological perturbations. The performance of a knowledge graph reasoning (KGR) task, however, can vary depending on the quality (e.g., accuracy, completeness) of the graph and the efficacy of the inference method. Methods for KGR range from rule-based logical reasoning to graph neural networks^20^. Among the available methods, PageRank has been widely used to perform graph-based reasoning and to quantify the influence of each node within a network, thereby enabling more informed inferences based on the relative importance and connectivity of nodes in complex systems.

To test the predictive accuracy of ICKG-based reasoning, we leveraged two complementary experimental datasets that profiled transcriptional responses to immune perturbations. The first was a scCRISPR screen in primary CD8 + T cells targeting 180 transcription factors (TFs), capturing their regulatory impacts at single-cell resolution^26^. The second was a comprehensive cytokine perturbation atlas that measured transcriptional changes in 17 immune cell types following stimulation with 86 cytokines in murine lymph nodes^27^. From each dataset, we identified ground-truth differentially expressed genes (DEGs) by comparing perturbed and control conditions (see “Methods”). These empirical DEGs serve as benchmarks to assess the biological accuracy of ICKG-based predictions.

For each perturbation, we performed in silico perturbation on the corresponding cell type-specific ICKG graph by locating the genes targeted by a perturbation, whether cytokine or TF, and performed reasoning on the graph to find the activated/inhibited genes (see “Methods”). To systematically assess the robustness and specificity of our graph-based reasoning approach, we compared the performance of 3 graph reasoning approaches: PageRank on ICKGs, adjusted random walk on ICKGs, and PageRank on randomly shuffled graphs against the ground truth. These comparisons were designed to disentangle the influence of the graph structure from that of the reasoning algorithm, providing a clearer understanding of each component’s contribution to the predictive accuracy. In parallel, to benchmark these results against conventional context-agnostic knowledge bases, we also calculated the Jaccard index, indicating the degree of overlap between the ground truth DEGs and all 50 Hallmark gene sets from MSigDB, using the top-scoring gene set as a reference for baseline performance of the Hallmark knowledge base.

**Fig. 3:**
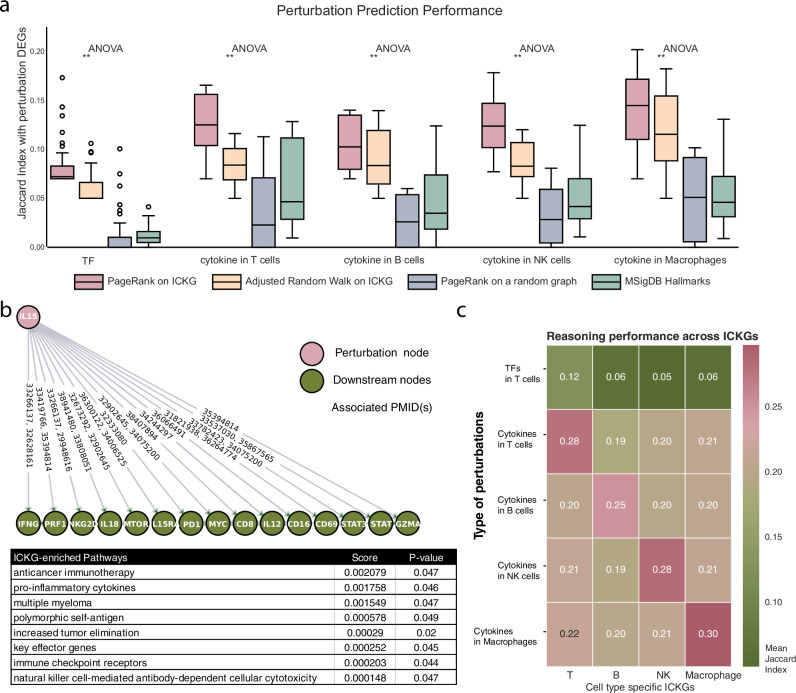
The reasoning performance of the constructed knowledge graphs. **a** Perturbation prediction performance across different methods. Jaccard index between ground truth differentially expressed genes (DEGs) and gene sets derived using PageRank on ICKGs, adjusted random walk on ICKGs, PageRank on random graphs, and MSigDB hallmark gene sets. Results are shown across five perturbation categories: transcription factors (TF) and cytokine stimulation in T cells, B cells, NK cells, and macrophages. PageRank on ICKGs generally yields higher overlap with ground truth DEGs, highlighting its utility for context-specific gene set annotation. **b** Shortest paths from IL15 to downstream genes in the NK-specific ICKG reveal predicted activation of key cytotoxic effectors (e.g., IFNG, PRF1, NKG2D, IL18, PDCD1). The ICKG-based annotations of these genes are highly enriched for NK-specific immune functions, including antibody-dependent cellular cytotoxicity. **c** Heatmap comparing the Jaccard index between predicted and ground truth DEGs for various perturbation types using different immune cell type ICKGs.

In Fig. 3a, we illustrate the superior predictive power of immune-cell-specific ICKGs in modeling transcriptional outcomes across diverse perturbations. Across all perturbations, PageRank, applied to the respective ICKG, consistently yielded significantly higher degrees of overlap with the ground truth DEGs than the control experiments. This performance far exceeded that of random walks or reasoning on randomized graphs, validating the specificity of both the graph topology and the inference method. Importantly, the ICKG-based predictions performed significantly better than the Hallmark-based annotations, indicating that ICKGs encode significantly more context-specific knowledge than the Hallmarks. A representative case study illustrates this distinction. IL-15 is a well-characterized cytokine essential for NK cell maturation and effector function, known to upregulate cytotoxic mediators including IFNG, PRF1, GZMB, and the activating receptor NKG2D^28,29^. We therefore used this cytokine to benchmark mechanistic fidelity. The best Hallmark set, *MYC Targets*, captured 14 of the DEGs (Jaccard=0.05) and the term “*MYC Targets*” clearly lacked NK-specific contexts. In contrast, PageRank on the NK-specific ICKG recovered 30 of the 442 DEGs (Jaccard=0.11), and our NK knowledge graph, using PageRank reasoning, prioritizes “natural killer cell-mediated antibody-dependent cellular cytotoxicity” (NK-mediated ADCC), a term that more accurately reflects the phenotypical changes induced by IL15 in NK cells, highlighting the value of the rich immune context embedded within the ICKGs. Notably, among the activated genes predicted by the ICKG, we identified IFNG, PRF1, NKG2D, CD8, and GZMA—all associated with enhanced NK cytotoxicity (Fig. 3b). Importantly, the ICKG also predicted STAT activation, which aligns with the known mechanism whereby IL-15 signals through JAK and activates STAT, providing additional mechanistic insight.

The example of IL-15 illustrated how a matched ICKG recovers canonical effector genes. We next asked whether this advantage holds systematically across all perturbations and cell types represented in our benchmark. For every perturbation (N = 46 TF knockouts and N = 43 cytokine stimulations), we recomputed PageRank four times, once on the corresponding immune cell type ICKG and once on each of the three mismatched graphs. We show that cell-type specificity is a dominant determinant of predictive accuracy (Fig. 3c), with ICKGs tailored to the corresponding immune cell type consistently achieving the highest Jaccard index with the ground truth DEGs relative to the mismatched graphs. For example, macrophage-specific ICKGs most accurately predicted macrophage cytokine responses, and the T-cell-specific ICKG outperformed others in predicting T-cell responses to both cytokine and transcription factor perturbations. This pattern indicates that lineage-specific wiring of intracellular signaling represented in each graph drives accurate inference. In aggregate, our results demonstrate that the four ICKGs embed clearly distinct cellular contexts, and recognizing the correct context adds substantial predictive power and biological reasoning performance across diverse immune settings.

To further pinpoint the suboptimal performance of certain perturbation predictions, we dichotomized the perturbations into low and high-performance groups by fitting a two-component Gaussian-mixture model to the Jaccard index distribution (see “Methods”). Perturbations in the high-performance group were driven by regulators that are network hubs, representing genes with a significantly higher degree of centrality in ICKGs (Fig. S3). In practical terms, when a cytokine receptor or transcription factor is highly represented in the literature and therefore highly connected, PageRank can propagate a signal through multiple, convergent paths to recover the downstream DEGs. Sparse nodes provide few routes and result in weaker predictions. This relationship points to a clear direction for refinement, where continual integration of new and emerging publications, especially those describing understudied genes or recently discovered regulators, will densify the graph and increase the predictive accuracy for the current low-performance group. Taken together, these findings demonstrate that the ICKGs not only capture granular, cell-type-specific immunological relationships but also provide a scalable framework for biologically grounded inference. Consistency between ICKG-based predictions and experimental perturbation data supports the utility of these knowledge graphs as robust and interpretable tools for functional gene set analysis in complex immune contexts. Moreover, PageRank emerges as a particularly effective reasoning strategy, offering a biologically meaningful way to prioritize downstream genes and pathways based on their relevance to the graph.

### ICKG-based gene set annotation outperforms conventional enrichment tools

Having demonstrated the predictive accuracy of ICKG-based biological reasoning, we next evaluated whether ICKG could be leveraged to enhance gene set annotation.

Unlike the canonical pathway databases that feature static and broadly defined gene sets and the LLM-based approach that outputs summarizations with limited contextual information, ICKG connects dynamic and literature-derived vocabularies that include genes, pathways, cell types and biomedical concepts, which allows more context-specific descriptions for gene set functions. This comprehensive network of curated relationships allows for biologically coherent enrichment, particularly enabling interpretation of complex gene signatures emerging from single-cell and spatial omics studies. To illustrate this, we used PageRank^30^ on each ICKG to annotate queried gene sets by identifying the most relevant terms to the input genes in the ICKG (see “Methods”). For example, when presented with a gene set consisting of four well-known NK inhibitory receptor genes: KIR2DL4, KLRC1, TIGIT and CD96, the NK-specific ICKG picked the annotation “*inhibitory immune checkpoints*” as the most relevant phenotype that reasoned through literature-supported paths. This approach provides not only accurate functional summaries but also enables a transparent and verifiable way for human experts to audit and interpret the annotation results (Fig. S4).

For systematic evaluation and benchmarking of this annotation strategy, we obtained gene sets from the meta programs (MPs) of four immune cell types (12 B, 10 CD4^+^ T cell, 12 CD8^+^ T cell, and 13 macrophage gene sets) constructed by Gavish et al.^31^, 4 tumor-reactive T cells signature^32–35^, and 4 NK gene sets related to cytotoxicity, inhibitory, stimulatory, and tumor-induced stress functions^9^. These gene sets reflect important molecular programs in immunology: Gavish’s MPs were derived by integrating 77 studies covering 24 tumor types, presenting conserved transcriptomic programs in different immune cell types; the tumor-reactive T cells signatures were derived experimentally to characterize clonally expanded T cells; and NK genes sets were derived from 716 patients with 24 cancer types, thus accurately annotating the functions of these gene sets with rich and relevant immune languages would indicate the broad applicability of our approach (Fig. S5a). We compared our method against both an LLM-based and an ORA-based method. We first annotated each gene set using the gold standard ORA based on Hallmarks, REACTOME, KEGG, and Wiki-Pathways from the MSigDB database^36^, which sum up to 2515 pathways, comparable to the number of pathway nodes in ICKGs, then we utilized the Gene Set AI (GSAI) tool developed by Hu et al.^14^, which tasks an LLM to analyze a gene set and generate a single summarizing phrase. We then annotated the same gene sets (*N* = 55) based on the respective ICKGs. As shown from the annotation results (Supplementary Tables), the ICKG-based approach produced terms that were more concise, interpretable, and better aligned with the gene set names originally chosen by the authors compared to ORA annotations.

**Fig. 4:**
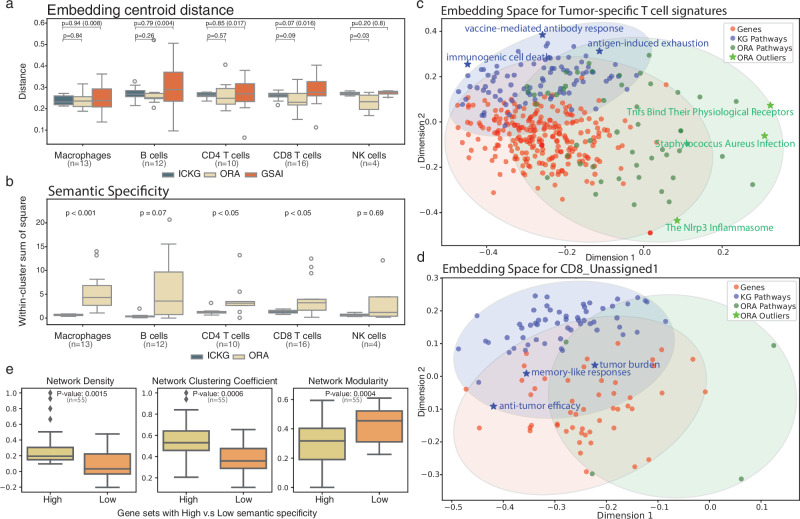
The gene set annotation performance of the constructed knowledge graphs. **a** Comparison of centroid distance resulting from the ICKG PageRank, MSigDB ORA and GSAI-based method. (Statistical significance for Levene’s variance test is shown in parentheses). **b** Comparison of semantic specificity resulting from the ICKG PageRank and MSigDB ORA method. Semantic specificity is approximated by the within-cluster sum of squares (WCSS) in the BERT embedding space, representing the degree of coherence of the functional annotation, with lower values indicating more homogeneous (more specific) annotations. Statistical significance between ICKG and MSigDB was assessed using the Mann–Whitney *U* test for each cell type. Metrics are shown separately for B cells, CD4 T cells, CD8 T cells, NK cells, and Macrophages. **c**, **d** Two-dimensional representation of genes in antigen-specific T cell gene set and CD8_unassigned1 gene set, and respective enriched pathways in the BERT embedding space. Individual genes (red dots) are shown alongside two types of pathways: ICKG Pathways (blue dots) and ORA Pathways (green dots). The proximity between dots suggests semantic or functional proximity. **e** Comparison of PPI network density, average clustering coefficient, and modularity between gene sets with high vs low semantic specificity.

To quantify the differences objectively, we projected the functional annotations obtained via ORA, LLM and ICKGs and individual genes onto the BERT embedding space and assessed their semantic relevance to the input gene set (see “Methods”). It has been shown that LLMs such as BERT can meaningfully represent biological terms in their embeddings^15^. Compared with the ORA annotations, we found that the ICKG annotations are as relevant (similar distances between cluster centroids in the embedding space) to the gene set of interest but are more specific, with a significantly smaller within-cluster sum of squares (Fig. 4a, b; Figs. S5b and S6), indicating more functionally coherent annotations. These advantages are clearly illustrated when annotating challenging or ambiguous datasets. For example, when we map the embeddings for an antigen-specific T cell signature^33^, we see ICKG annotations are more condensed with highly relevant annotations, such as “*vaccine-mediated antibody response*” and “*antigen-induced exhaustion*”, while ORA-annotations are more spread out with functions not immediately relevant to the core functionality of the gene set (Fig. 4c). Serving as a sanity check that the BERT-based semantic representations align with biological interpretation, we include in Fig. S5c a comparison of ICKG and ORA embeddings against randomly generated pathway embeddings. As expected, the randomly generated terms produce embeddings that are widely scattered and located far from the biologically meaningful clusters. In addition, the annotations generated by ICKGs are shorter in string length (average length in characters is 25 for ICKG terms and 47 for ORA terms), suggesting that our ICKG approach can use concise language while capturing the core functionality. Compared with the GSAI annotations, we observed that they generally align with the center of our ICKG-based annotations. However, for a subset of gene sets, the GSAI-derived results had significantly larger variance than the other approaches with *p*-values = 0.008, 0.004, 0.017, 0.016, and 0.8 for Macrophages, B cells, CD4/CD8 T cells and NK cells, respectively (Levene’s test) (Fig. 4a and Fig. S6). The benchmarking results support the validity of our ICKG approach as it returned terms of similar semantic distance as terms identified independently by GSAI, with higher reliability (smaller variance). For example, on the *B_cells_Memory* and *B_cells* gene sets, the GSAI annotation significantly diverged from those represented by the individual genes and by the ICKG terms, annotating them as “*Immune Response and Cell Adhesion*” and “*Chromatin Remodeling and Transcriptional Regulation*” Additionally, ICKGs provide literature verified reasoning and inference contexts through their edge metadata, whereas GSAI reduces each gene set to a single functional label with minimal contextual information. While this reduction can be convenient for clear-cut annotation, it may oversimplify pathway-level biology where multiple, interacting functions must be represented. Because ICKGs are systematically derived from curated literature, they capture both functional labels and the associated mechanistic context, offering a more informative and biologically grounded framework for gene set annotation

Notably, ICKGs succeeded in annotating gene sets when traditional enrichment tests failed, potentially broadening the applicability of ICKG in annotating small or novel gene sets. Gavish et al. manually named each gene set based on empirical knowledge and the ORA results, but they did not name three T cell-related MPs, likely due to challenges and limitations of summarizing small gene sets when the enriched terms are spread out in functionality in ORA-based annotation using MSigDB (Fig. 4b). Our approach was able to annotate the three MPs with literature supports. According to the T cell KG (Supplementary Table), CD4_Unassigned is enriched in “*cytotoxic antitumor effects*” and “*memory cell differentiation*”, suggesting a tissue resident T cell phenotype. When we further queried its constituent genes in the T KG, we found this gene set is enriched in genes linked to “*cytotoxic antitumor effects*” (ISG15, GZMA, PRDX1, KLRB1) and “*memory t cells*” (LGALS1, LY6E, COTL1) (Fig. S7a). The CD8_Unassigned1 and CD8_Unassigned2 correspond to cytotoxic CD8 T cells but differ in their functional states and roles. According to the T KG, CD8_Unassigned1 is associated with “*anti-tumor efficacy*” and “*tumor burden*“ (Fig. 4d and Supplementary Table), likely representing CD8 T cells in an intermediate or early stage of exhaustion with genes linked to “*cytotoxic T cells*” (GZMK, KLRG1) and “*memory T cells*“ (CD27, IL7R), though facing metabolic stress (TXNIP) as they progress towards exhaustion (Fig. S7b). In contrast, CD8_Unassigned2 is enriched in “*cytotoxic extracellular vesicles*” and “*tumoricidal factors*“ (Supplementary Table), suggesting tissue-resident memory (TRM) phenotypes with genes linked to “*antitumor cytotoxicity*“ (LY6E, IL32, GZMA, ACTB) and “*cytotoxic extracellular vesicles*“ (TXN) (Fig. S7c).

To further substantiate the semantic specificity metric obtained from BERT, we dichotomized the gene sets by their semantic specificity using a Gaussian mixture model and examined the distribution of the genes in the protein–protein interaction (PPI) network for each gene set (see “Methods”). We found that gene sets in the low semantic specificity group exhibit significantly lower network density and clustering coefficient, and they have higher modularity scores, indicating a higher degree of heterogeneity (Fig. 4e).

## Discussion

In this study, we present a scalable and transparent knowledge graph-based framework to unify fragmented insights dispersed across the immunological literature. By constructing cell-type-specific knowledge graphs from over 24,000 PubMed abstracts, we created a structured, explainable system for capturing immune-specific biological relationships. This framework enables literature-supported reasoning at scale, and offers an interpretable foundation for discovery, gene set interpretation and hypothesis generation in immunology research. We constructed ICKGs for four major immune cell types: T cells, B cells, NK cells, and macrophages by parsing and integrating immune cell-related abstracts published on PubMed between January 1, 2020, and September 1, 2024. This construction pipeline combines LLMs with “human verifiable” verification, striking a balance between the scalability of automated extraction and the precision of expert curation. This approach ensures factual accuracy, minimizes hallucination, and results in a biologically meaningful, literature traceable graph for each cell type. We showed that ICKGs capture cell-type-specific immune contexts with a level of granularity and biological specificity that go beyond current pathway databases. While our results show that incorporating cell type specificity improves predictive performance, we currently establish the graphs at the major cell type level, as increasing granularity to cell subtype or cell states would likely reduce the accuracy and usability of the graphs. Nonetheless, as knowledge and literature about immune cells increase, it will likely be productive to further extend the ICKGs to represent the granular cell type context. By leveraging literature-derived relationships, ICKGs can systematically annotate novel gene sets using immune-specific vocabularies, achieving accurate and mechanistically based interpretations that are often missed by conventional enrichment approaches. As novel gene sets and signatures are being discovered from large-scale single-cell and spatial omics studies, our approach offers a scalable solution for contextualizing these findings, facilitating data interpretation and hypothesis generation, and guiding experimental validation in both basic and translational immunology. Importantly, our work can support initiatives such as the Immune Cell Atlas projects^7,37^ by providing interpretable characterization of immune cellular subtypes based on respective DEGs. This could enhance our ability to map diverse immune states, resolve translational phenotypes and prioritize targets for experimental validation based on regulatory relationships supported by literature.

Our ICKG-based annotation is transparent and verifiable, overcoming key limitations in approaches that directly apply LLMs or deep learning methods to gene set annotation. Unlike LLMs that generate texts without grounded references, which are prone to generate non-reproducible or “hallucinated” outputs, our approach ensures that all inferences are grounded in peer-reviewed literature and are traceable to specific source publications. This traceability preserves scientific rigor, enhances reproducibility and supports reliable knowledge acquisition and interpretation. In addition, the reasoning paths behind each annotation task can be visualized through queryable ICKG subgraphs, providing clear and interpretable insights into how and why specific biological concepts are prioritized. This transparency facilitates expert validation and allows researchers to explore the underlying logic, bridging the gap between AI-based inference and human interpretability.

Beyond enabling transparent annotation, our framework also provides an advancement in biomedical text mining, particularly in pathway-level named entity recognition (NER), which continues to lack systematically labeled training data. To address this, we established a set of clear and universally generalizable criteria and manually annotated over 48,000 words instrumental for the training and fine-tuning of pathway NER models. This resource provides a significant benchmark for improving the extraction of pathway-level concepts from biomedical literature. In parallel, we also formulated a hybrid pipeline that combines rule-based methods and prompt engineering to robustly identify and extract biomedical concepts from texts that contain phrases that are both rich and specific in immunobiology descriptions. Automating the extraction of these biomedical concepts from literature is essential for maintaining a continuously expandable, up-to-date knowledge base that evolves in parallel with the immunology field and enhances the interpretive power of ICKGs over time.

While ICKGs constructed in this study can be powerful tools, several limitations should be considered. First, the knowledge graphs reflect inherent literature biases, tending to overrepresent frequently studied genes and recent publications. While normalization techniques and expanded coverage of older literature could address these biases, our current approach effectively captures the research landscape, offering a good starting point for further development. Second, our analysis was based on abstracts instead of full-text. Full texts potentially provide comprehensive textual descriptions of molecular mechanisms and pathway relationships, but can pose substantial challenges (such as parsing errors, redundancy and high computational cost, etc.) that should be carefully investigated in a future study. Third, we tasked LLMs to dichotomize entity relationships into either activation or inhibition. Although this facilitates efficient and intuitive reasoning, the simplification can limit biological granularity and obscure context-dependent regulatory relationships. Incorporating probabilistic relationships or expanding to additional interaction types, such as binding or co-expression, could further enhance the biological fidelity of future ICKG iterations.

While our initial focus was on four key immune cell types, the ICKG framework is highly adaptable and can be extended to other cell types and omics modalities. Derived from curated cancer immunology–focused abstracts published within the past five years, our ICKGs are enriched for context-specific and immune-relevant genes, which, as a result, do not capture the whole transcriptome. However, the graph’s extensible structure allows for seamless integration of additional biological content as the literature base grows. This scalability supports a wide range of applications, including molecular stratification, outcome prediction, genetic screening, and risk assessment in both research and clinical contexts. The scalability of ICKG construction is warranted by a complete Python-based pipeline consisting of the following automated steps: (1) automatically retrieve relevant PubMed abstracts based on keyword queries, (2) perform NER, (3) perform RE, and (4) assemble the graph. Pretrained NER models are provided to identify genes, diseases, cell types, and pathways at no additional cost. For RE, we implemented a carefully designed prompt and utilized Groq to interface with the LLaMA 3.1 API. This approach incurs minimal cost—processing a graph from approximately 3,000 abstracts typically costs under $5 and completes within an hour. For example, querying 3,665 NK-related publications took approximately 40 minutes at a cost of $6.58, producing an NK ICKG comprising 6,001 nodes and 24,901 edges. Alternatively, users can eliminate this cost by deploying an open-source version of the LLaMA locally.

Our study focused on four immune cell types as a proof of concept, confirming that biomedical abstracts can be systematically organized into a structured framework (i.e., ICKG) to enable accurate cell-type-specific annotation. While we did not explicitly test a unified graph encompassing all abstracts, such an approach is conceptually feasible and could facilitate broader annotation across diverse cell types. However, the advantages of such a unified model—namely, comprehensive coverage—must be balanced against potential limitations, including reduced specificity and potential bias toward well-studied cell types, which may limit its performance on rare or less-characterized cell populations.

More broadly, our study highlights the transformative potential of AI-driven knowledge integration and graph-based reasoning in advancing immunological research. While gene set annotation serves as a compelling use case, the underlying framework offers a platform to accelerate understanding in complex omics studies – enabling mechanistic inference of immune cell functions, prioritization of key gene targets, identification of cell–cell communication axes, contextualization of pathway activities, and generation of testable hypotheses for experimental or clinical validations. While these applications are beyond the scope of this current study, we have provided a Supplementary Note to highlight future use cases along with supporting examples. To facilitate user-friendly access, we developed an interactive web-based application that allows researchers to perform customized cell-type-specific gene set annotation using the four ICKGs. (https://kchen-lab.github.io/immune-knowledgegraph.github.io/). This platform enables systematic querying of recent literature to support transparent, biologically informed interpretation of complex datasets. Moving forward, continued integration of diverse data sources, algorithmic refinement for accurate and efficient reasoning, broader applications with these ICKGs, and close collaboration between computational and experimental immunology will be key to realizing the full potential of this framework. As high-dimensional immunology datasets continue to expand, transparent and literature-aware tools such as ICKGs will be essential for closing the gap between raw data and mechanistic insights, advancing both basic science discovery and translational innovation.

## Methods

### Named entity recognition (NER) task

We finetuned the BioBERT-Large v1.1 model, which was pretrained on 1 million PubMed articles and built on the BERT-large-Cased architecture with a custom 30,000 vocabulary, using PyTorch. The fine-tuning was performed using multiple NER datasets that were previously labeled to recognize relevant entities. For gene names, we used BC2GM, BioNLP13PC, and JNLPBA. For disease names, we used NCBI and BC5CDR. For cell type names, we used GENIA. We finetuned the BioBERT model with these datasets and selected the final finetuned model for each NER task based on precision (i.e., the proportion of correctly recognized entities out of all recognized entities), ensuring optimized performance in domain-specific NER tasks.

$${\rm{Precision}}=\frac{{\rm{True\; Positives}}}{{\rm{True\; Positives}}+{\rm{False\; Positives}}}$$

### Pathway extraction

We developed a pipeline to extract pathways and relevant biomedical phrases from PubMed abstracts using spaCy^38^ and a custom set of rules based on grammatical structures. We split each abstract into sentences, each of which is then processed using the en_core_web_sm language model in spaCy. We extracted noun phrases that are not single tokens and excluded common stop words, appending these to a set of candidate pathway terms. To refine the extracted terms, we utilized the Unified Medical Language System (UMLS) API query to identify biomedical terms, applying Levenshtein similarity to ensure close matches to known biomedical terms. Terms are further filtered using a set of criteria that prioritize biological relevance, such as specific suffixes (“ase,” “in,” “ion,” “itis,” “osis,” “oma,” “icity,” and “pathy”), substrings (“cell,” “gene,” “protein,” “receptor,” “antibody,” “immune,” “tumor,” “cancer,” “virus,” and “bacteria”) related to biology, and exclusion of non-biomedical entities. Finally, we fed the cleaned terms into the llama 3 70B-parameter model, requesting it to review the terms and output only the informative ones. This approach allows us to systematically extract and validate pathways, improving the accuracy of entity recognition in biomedical texts.

### Relationship extraction (RE)

To infer pairwise relationships between extracted entities in each abstract, we utilized a zero-shot prompt engineering approach. First, all possible pairs of terms were generated using pairwise permutations of the extracted entities. For each entity pair, we provided a detailed prompt to the Llama 3.1 model (70B parameters), instructing it to determine the directional relationship between the two terms. The model inferred whether the first term activated, inhibited, or had no association with the second term based on the content of the abstract. Clear guidelines were given to the model, specifying that relationships should only be inferred from the first term to the second, with ambiguous or biologically irrelevant relationships marked as “no association.” The model’s output was parsed and filtered to retain only relationships classified as “Activate” or “Inhibit,” which were subsequently used for downstream analyses.

To evaluate the performance of RE, we leveraged different databases. Gene-gene relationships were benchmarked with the KEGG database, and Gene-cell type relationships were validated using an Immune Cell Atlas. For gene–gene relationships, we used the *KEGGREST* package in R and extracted more than 55,000 pairwise relationships between genes based on 361 KEGG pathways. For comparability, we simplified the relationships into either activation or inhibition based on interaction types: activation will encompass all of the following KEGG-defined interaction types: “activation,” “activation-indirect effect,” and “activation-indirect;” inhibition includes the following: “inhibition,” “inhibition-indirect effect,” “inhibition-repression,” and “repression-indirect effect,” which sum up to 15,832 activate and 3751 inhibit relationships. We output all the gene–gene pairs along with their directionality from ICKG and constructed a confusion matrix based on the overlapping gene–gene pairs.

**Table:** 

	Activate in KG	Inhibit in KG	
Activate in KEGG	A	B	A + B
Inhibit in KEGG	C	D	C + D
	A + C	B + D	*E* = No. of gene–gene pairs in both KG and KEGG

For gene-cell type relationships, we output all the gene nodes that were direct neighbors of that cell type of interest in the KG and benchmarked them with differentially expressed genes (DEGs) for the corresponding cell type of interest using the immune cell atlas dataset, then we constructed a confusion matrix.

**Table:** 

	Positive log2FC	Negative log2FC	
Directly activate the cell type	A	B	A + B
Directly inhibits the cell type	C	D	C + D
	A + C	B + D	*E* = No. of genes in both the KG and DEG lists

$${\rm{Precision}}=\frac{A}{A+B}$$

$${\rm{Recall}}=\frac{A}{A+C}$$

$${\rm{F}}1-{\rm{score}}=2* \frac{{Precision}* {Recall}}{{Precision}+{Recall}}$$

For both RE evaluations, we used the *select* function from the *AnnotationDbi* package in R to output a possible alias for each gene, and we considered a match if a gene matched any of the aliases of the genes in the benchmarking database.

The inferred relationship between other types of biomedical entities (e.g., gene-disease, disease-pathway) is subject to further validation using independent sources.

### PageRank

We implemented PageRank using the NetworkX package (version 2.6) in Python (version 3.8.18). We constructed our network graph using NetworkX, and we then applied the PageRank algorithm with the following function: *nx.pagerank(G, alpha* = *0.85, personalization=phenotype_node)*, where G is our weighted network, alpha is the damping factor (default to 0.85), personalization is set to assign higher importance to specific nodes of interest (a list of genes if we are interested in gene set annotation, one gene if we are interested in its downstream impact). Other parameters in this function were set to default values.

### Permutation test

We implemented a permutation test to assess the significance of PageRank scores for the selected nodes in the network. We first computed personalized PageRank scores using the input gene set as personalization nodes. We then performed 1000 permutations, each time randomly selecting a gene set of the same size as the input set and computing PageRank scores. For each selected node, we compared its actual PageRank score to the distribution of scores from the permutations. *p*-values were calculated as *n*/1000, where *n* is the number of permutations yielding a score greater than or equal to the actual score. This approach allowed us to determine whether the PageRank scores for pathway nodes were significantly higher than expected by chance, given the network structure. Additionally, we computed 95% confidence intervals for each selected node’s PageRank score using the 2.5th and 97.5th percentiles of the permutation distribution.

### Adjusted Random Walk

We implemented an adjusted random walk using the NetworkX package (version 2.6) in Python (version 3.8.18). We used *numpy.random.choices()* for weighted path selection, with transition probabilities proportional to exp(w/T), where w is the edge weight, and T is a temperature parameter. For each node, 1000 random walks of length 20 steps are performed. Node importance is quantified by normalized visit frequency.

### Over-representation analysis (ORA)

We performed ORA using the *enricher* function in the *clusterProfiler* package in R. For gene set definitions, we utilized the *msigdbr* package, which provides access to the Molecular Signatures Database (MSigDB) for Hallmark and C2 collections.

### Jaccard Index

To assess the performance of perturbation prediction, we used experimentally derived DEGs for each perturbation as the ground truth. For each method, we computed the Jaccard index between the DEGs and the predicted gene sets generated by: (1) PageRank on the ICKG, (2) adjusted random walk on the ICKG, and (3) PageRank on a randomly permuted graph. We also calculated the maximum Jaccard index between the DEGs and all MSigDB hallmark gene sets.

### Gene and annotation embeddings

Since BERT is trained on natural language, inputting gene name lists alone does not yield meaningful embeddings, as biological abbreviations lack contextual depth. To address this, we extracted NCBI summary paragraphs for each gene, capturing their functional descriptions. For fair comparison, we also generated a summary paragraph for each annotation. For MSigDB annotations, we looked for a full description (or a brief description, if a full description was not available) on the MSigDB webpage. For ICKG annotations, we created a prompt and asked Llama3-70B parameter model to generate a short summary paragraph for each term. As all dimensions in BERT embedding contribute collectively to represent contextual meaning, we fed the summary paragraphs for genes and annotations into a pretrained BERT base model to obtain contextual embeddings. These high-dimensional embeddings were then projected into two dimensions using a PCA to visualize the semantic relationships between genes and annotations in a 2D space

### Gaussian Mixture Model

We employed the Gaussian Mixture Model (GMM) to categorize within-cluster sum of squares (WCSS) data into two groups, aiming to identify clusters that indicate high and low levels of variance. We used the *GaussianMixture* function from *sklearn.mixture* python library, specifying two components and a random seed for reproducibility *(n_components* = *2, random_state* = *0)*. After fitting GMM to the WCSS data using the *fit* method, we extracted the mean of each component from the model. The component with the higher mean was labeled as “High”, representing clusters with higher variance (lower specificity), whereas the component with the lower mean was labeled as “Low,” indicative of clusters with lower variance (higher specificity).

### Protein–protein interaction network

We fetched protein–protein interaction (PPI) data from the STRING database for each gene set. This interaction data was then employed to construct functional gene interaction networks using the *NetworkX* library. Density reflects how tightly nodes are linked. Clustering coefficient reveals local connectivity. Modularity evaluates how well a network divides into tightly-knit communities with sparse interconnections, shedding light on the network’s overall structure. Specifically, we utilized the *nx.Graph()* function to create the graph structure, populating it with nodes and weighted edges based on interaction scores. The constructed networks were analyzed to calculate key structural metrics: network density, clustering coefficient, and modularity. These metrics were computed with *nx.density(), nx.average_clustering()*, and *nx.algorithms.community.modularity()*.

### Differential gene expression

Differential expression gene (DEG) analysis was performed using R (version 4.2.3) and the Seurat package (version 4.1.4). The preprocessed and normalized single-cell RNA sequencing data were analyzed using the *FindAllMarkers* function from Seurat with the default parameter settings. Genes were considered DEGs with |log2FC | > 1 and adjusted *p*-value < 0.05. The *p*-values were adjusted for multiple testing using the Bonferroni correction method.

### Llama3-70B Prompt

Figure S8 shows the full prompt to guide the agent for relationship extraction.

## Supplementary information

Supplementary Information

## Data Availability

All codes have been made available in https://github.com/KChen-lab/ICKG/tree/main/, including the extraction of PubMed abstracts, biomedical entities recognition, knowledge graphs construction, and gene set annotations. The finetuned NER models for genes, diseases and cell types, and the manually annotated pathway database based on the GENIA database have also been uploaded on https://github.com/KChen-lab/ICKG/tree/main/finetuned_NER_models. In addition, the manually annotated pathway corpus used for NER fine-tuning (approximately 48,000 words) is also publicly available at https://github.com/KChen-lab/ICKG/tree/main/finetuned_NER_models. To facilitate a user-friendly application, we have created a website for immune cell type-specific gene set annotations: https://kchen-lab.github.io/immune-knowledgegraph.github.io/.

## Code availability

All codes have been made available in https://github.com/KChen-lab/ICKG/tree/main/, including the extraction of PubMed abstracts, biomedical entities recognition, knowledge graphs construction, and gene set annotations. The finetuned NER models for genes, diseases and cell types, and the manually annotated pathway database based on the GENIA database have also been uploaded on https://github.com/KChen-lab/ICKG/tree/main/finetuned_NER_models. In addition, the manually annotated pathway corpus used for NER fine-tuning (approximately 48,000 words) is also publicly available at https://github.com/KChen-lab/ICKG/tree/main/finetuned_NER_models. To facilitate a user-friendly application, we have created a website for immune cell type-specific gene set annotations: https://kchen-lab.github.io/immune-knowledgegraph.github.io/.
