# Supplementary material for: AI-powered Immune Cell Knowledge Graph (ICKG) with granular immune contexts enables immune program interpretation
Source: NPJ Artif Intell. 2026 Jan 27;2(1):13. doi: 10.1038/s44387-025-00060-4 (PMC12846910; doi:10.1038/s44387-025-00060-4)
Supplement: Supplementary file 1 — Supplementary Information [file 44387_2025_60_MOESM1_ESM.pdf]

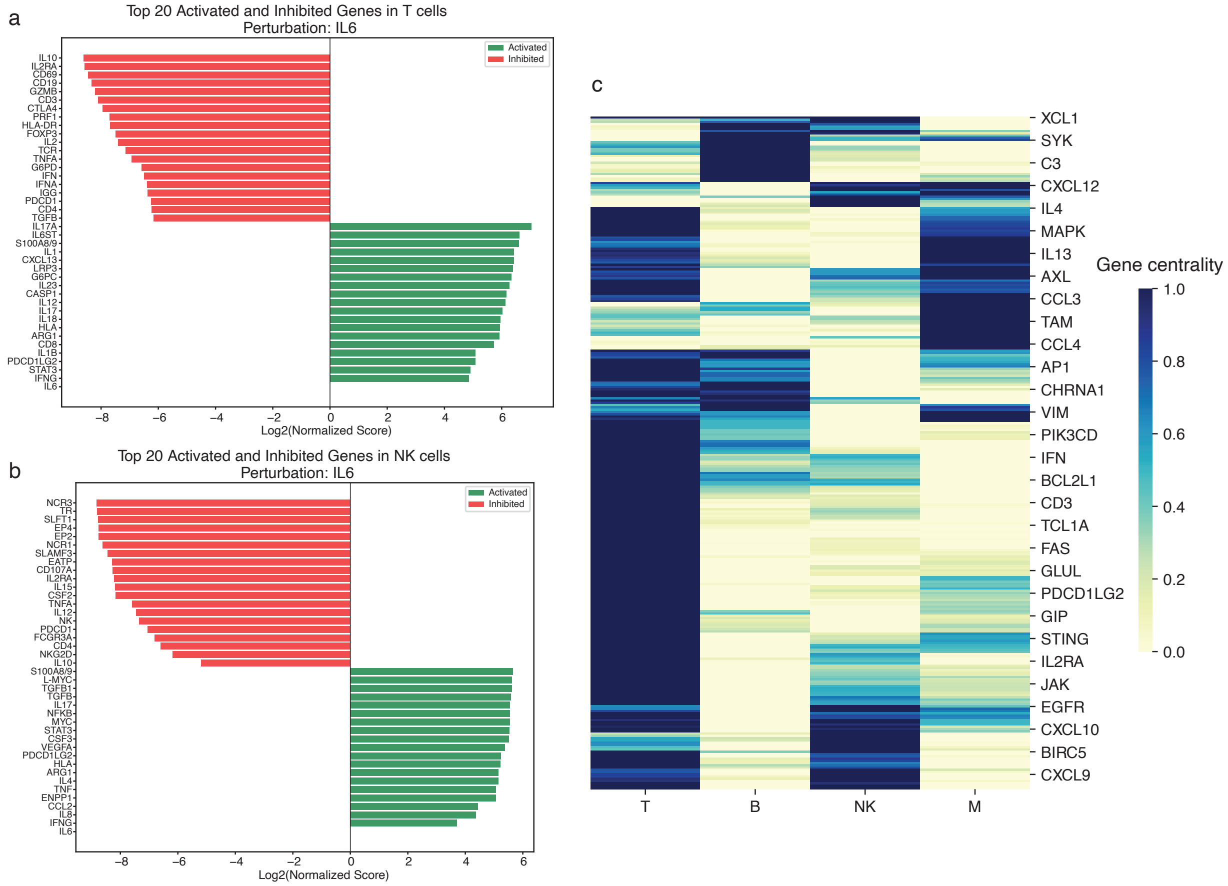

Figure S1. (a-b) Bar plots show the top 20 genes significantly activated (green) or inhibited (red) following IL-6 perturbation in (top) T cells and (bottom) NK cells. (c) Heatmap of overlapping genes present in all four ICKGs, showing their degree centrality (number of edges connected to the gene) within each network. Values are row-normalized to highlight relative differences across T cell, B cell, NK cell, and myeloid cell–focused knowledge graphs. Genes are clustered by similarity in centrality profiles, illustrating distinct patterns of literature emphasis across cell type–specific contexts.

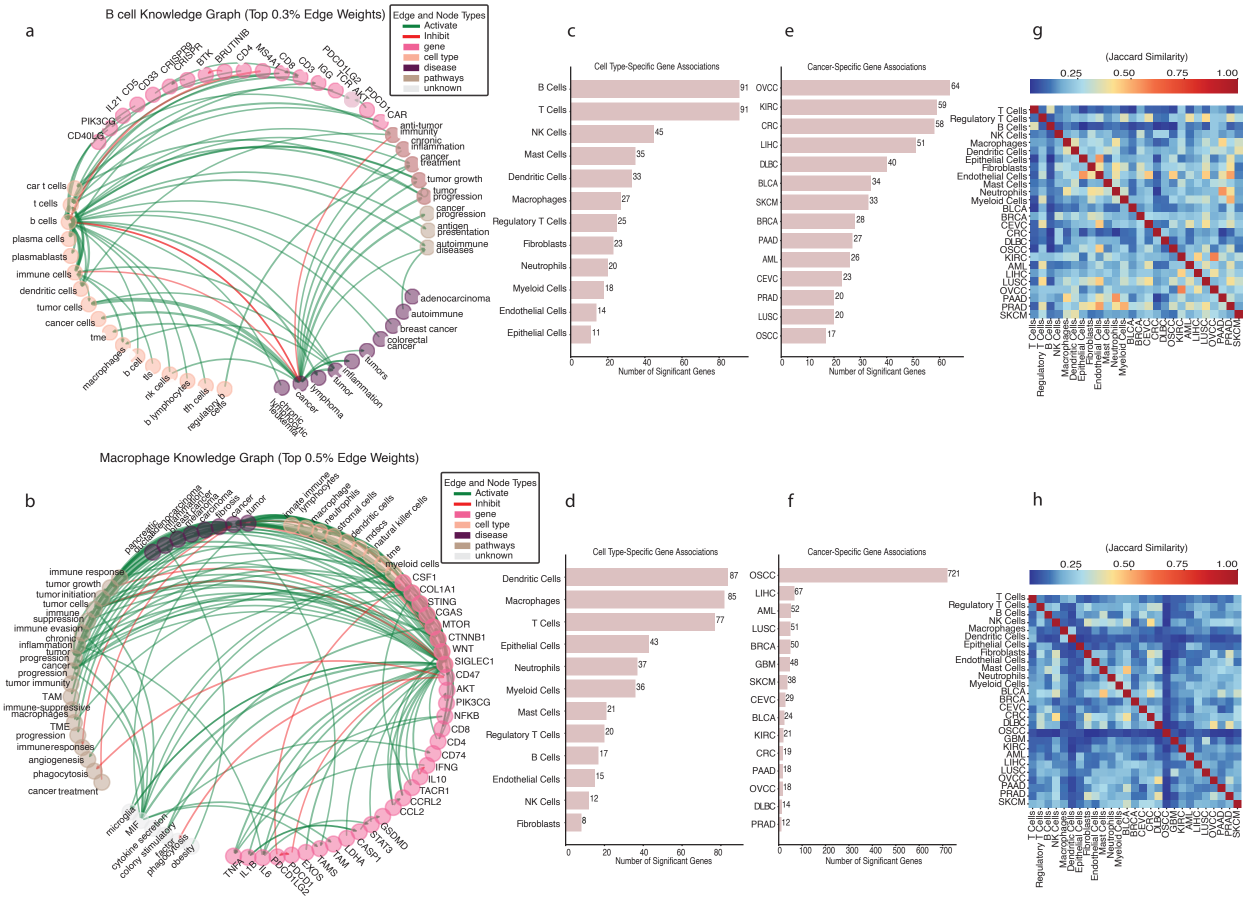

Figure S2. (a-b) B cell and Macrophage specific knowledge graph subset containing only edges with top weight. Nodes are colored based on node types (gene, disease, cell type, pathways and others). Edges are colored based on activation or inhibition between the two connecting nodes. The thickness of the edges represents the number of published abstracts that support such relationship (c-d). Number of significantly associated genes with each major cell types derived from B cell and macrophage specific knowledge graph (e-f). Number of significantly associated genes with each major TCGA cancer type according to PageRank scores derived from B cell and macrophage specific knowledge graphs. (g-h) Pairwise jaccard distance (measure for the number of overlapping genes) across cancer types and cell types derived respectively from B cell and macrophage specific knowledge graphs.

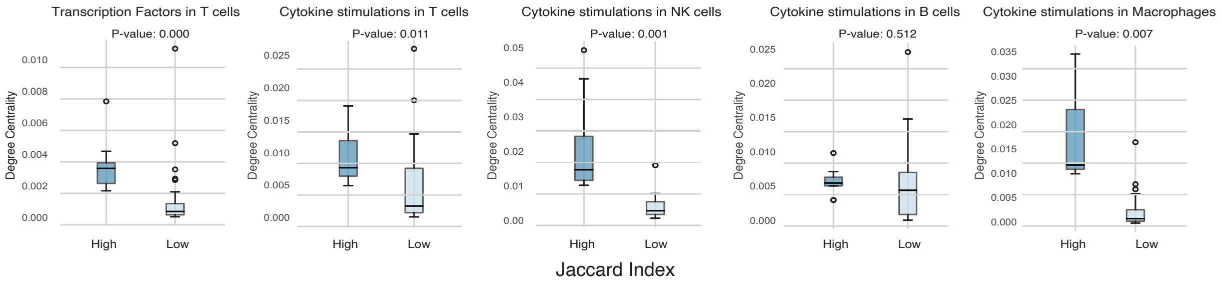

Figure S3. The degree centrality of the perturbed genes in ICKGs for experiments achieving high vs those achieving low jaccard index.

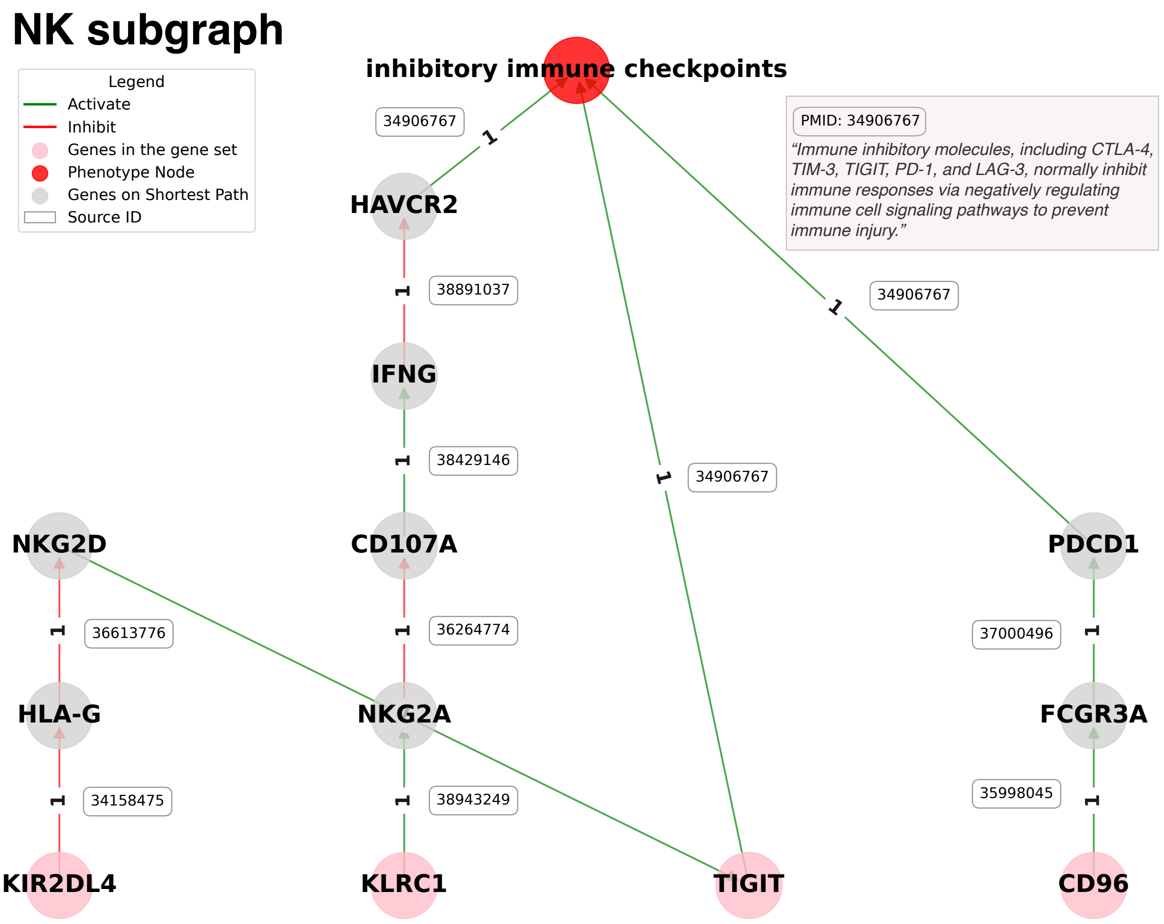

Figure S4. This figure displays a subnetwork generated by querying the NK Immune Cell Knowledge Graph (ICKG) with specific input nodes. The query included four genes of interest (pink nodes: TIGIT, KIR2DL4, KLRC1, and CD96) and one phenotype of interest (red node: "inhibitory immune checkpoints"). The graph identified the shortest paths connecting these input nodes, revealing additional intermediate genes (gray nodes) and their regulatory relationships. Green edges represent activating interactions, while red edges indicate inhibitory relationships. Each connection is annotated with source identifiers (PMIDs) from the scientific literature.

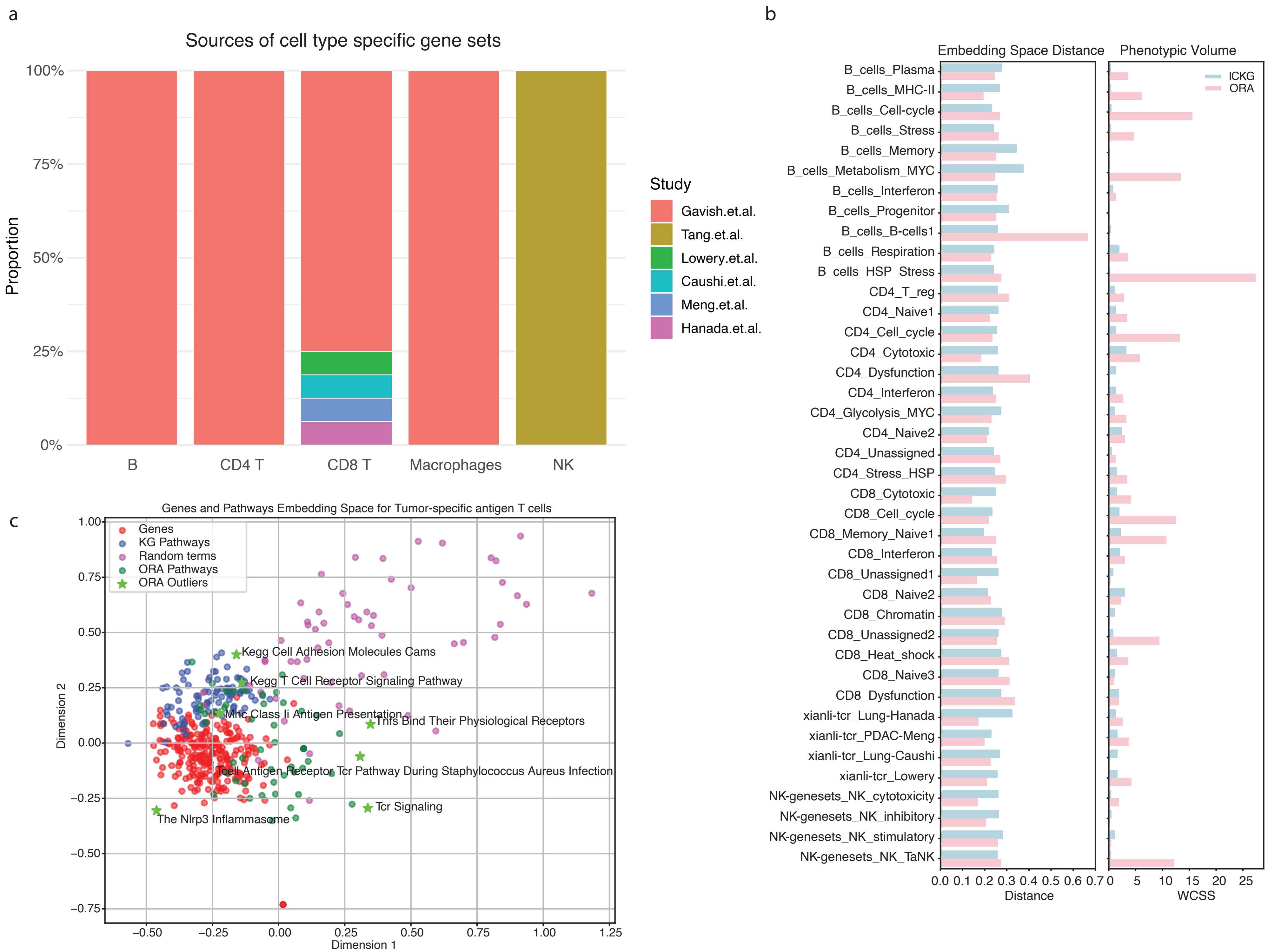
a
Sources of cell type specific gene sets
Proportion
100%
75%
50%
25%
0%
B
CD4 T
CD8 T
Macrophages
NK
Study
Gavish.et.al.
Tang.et.al.
Lowery.et.al.
Caushi.et.al.
Meng.et.al.
Hanada.et.al.
b
Embedding Space Distance
Phenotypic Volume
ICKG
ORA
B_cells_Plasma
B_cells_MHC-II
B_cells_Cell-cycle
B_cells_Stress
B_cells_Memory
B_cells_Metabolism_MYC
B_cells_Interferon
B_cells_Progenitor
B_cells_B-cells1
B_cells_Respiration
B_cells_HSP_Stress
CD4_T_reg
CD4_Naive1
CD4_Cell_cycle
CD4_Cytotoxic
CD4_Dysfunction
CD4_Interferon
CD4_Glycolysis_MYC
CD4_Naive2
CD4_Unassigned
CD4_Stress_HSP
CD8_Cytotoxic
CD8_Cell_cycle
CD8_Memory_Naive1
CD8_Interferon
CD8_Unassigned1
CD8_Naive2
CD8_Chromatin
CD8_Unassigned2
CD8_Heat_shock
CD8_Naive3
CD8_Dysfunction
xianli-tcr_Lung-Hanada
xianli-tcr_PDAC-Meng
xianli-tcr_Lung-Caushi
xianli-tcr_Lowery
NK-genesets_NK_cytotoxicity
NK-genesets_NK_inhibitory
NK-genesets_NK_stimulatory
NK-genesets_NK_TaNK
0.0 0.1 0.2 0.3 0.4 0.5 0.6 0.7
Distance
0 5 10 15 20 25
WCSS
c
Genes and Pathways Embedding Space for Tumor-specific antigen T cells
Genes
KG Pathways
Random terms
ORA Pathways
ORA Outliers
Kegg Cell Adhesion Molecules Cams
Kegg T Cell Receptor Signaling Pathway
Mhc Class Ii Antigen Presentation
Tnfs Bind Their Physiological Receptors
Tcell Antigen Receptor Tcr Pathway During Staphylococcus Aureus Infection
Tcr Signaling
The Nlrp3 Inflammasome
Dimension 2
Dimension 1
1.00
0.75
0.50
0.25
0.00
−0.25
−0.50
−0.75
−0.50
−0.25
0.00
0.25
0.50
0.75
1.00
1.25

Figure S5. (a) Proportion of immune cell type–specific gene sets derived from 6 published studies, grouped into B cells, CD4 T cells, CD8 T cells, macrophages, and NK cells. (b) Comparison of ICKG vs ORA MSigDB-derived annotations across 55 immune gene sets. Embedding space distance (left) measures annotation similarity between the two methods, while phenotypic volume (WCSS, right) quantifies within-method coherence, which lower values representing more semantic specificity. Red boxes highlight the three gene sets failed to be annotated by ORA method. (c) Two-dimensional representation of genes in antigen-specific T cell gene set and respective enriched pathways in the BERT embedding space. Individual genes (red dots) are shown alongside three types of pathways: ICKG Pathways (blue dots), ORA Pathways (green dots), and randomly selected pathways (purple). The proximity between dots suggests semantic or functional proximity.

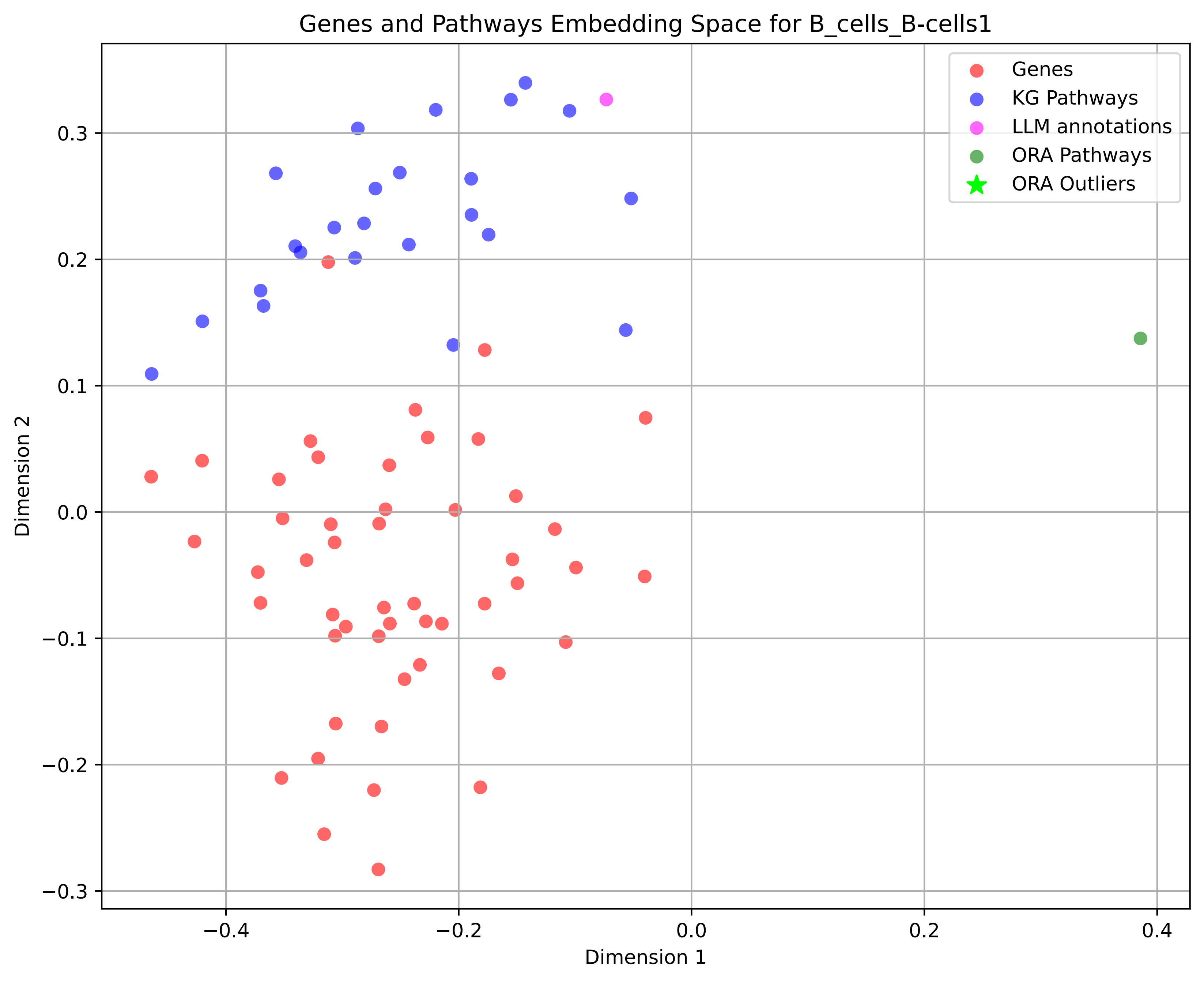
Genes and Pathways Embedding Space for B_cells_B-cells1
Genes
KG Pathways
LLM annotations
ORA Pathways
ORA Outliers
Dimension 1
Dimension 2
−0.4
−0.2
0.0
0.2
0.4
−0.3
−0.2
−0.1
0.0
0.1
0.2
0.3

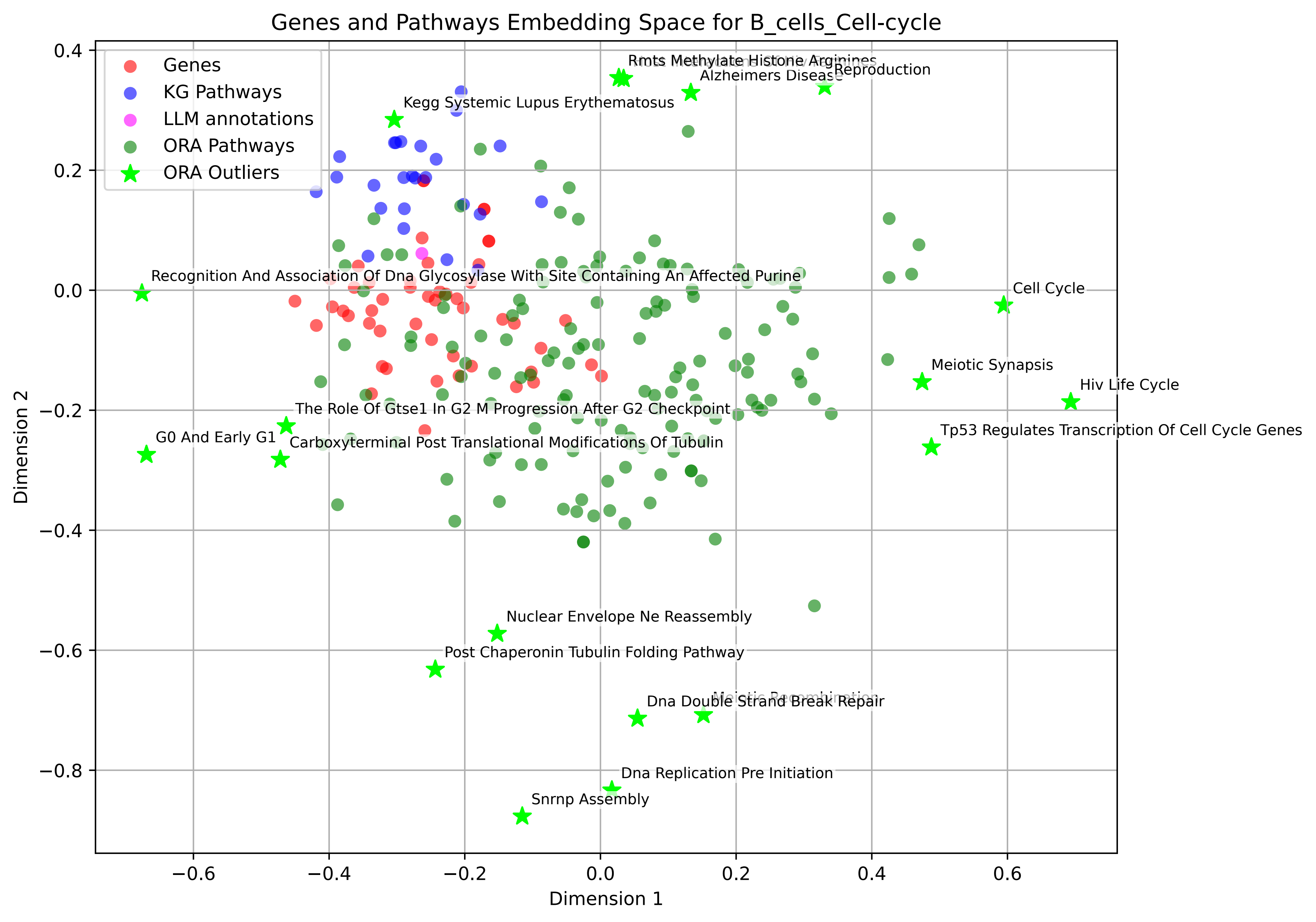

Genes and Pathways Embedding Space for B_cells_Cell-cycle
Genes
KG Pathways
LLM annotations
ORA Pathways
ORA Outliers
Rmts Methylate Histone Arginines
Alzheimers Disease
Reproduction
Kegg Systemic Lupus Erythematosus
Recognition And Association Of Dna Glycosylase With Site Containing An Affected Purine
Cell Cycle
Meiotic Synapsis
Hiv Life Cycle
The Role Of Gtse1 In G2 M Progression After G2 Checkpoint
G0 And Early G1
Carboxyterminal Post Translational Modifications Of Tubulin
Tp53 Regulates Transcription Of Cell Cycle Genes
Nuclear Envelope Ne Reassembly
Post Chaperonin Tubulin Folding Pathway
Dna Double Strand Break Repair
Meiotic Recombination
Dna Replication Pre Initiation
Snrnp Assembly
Dimension 1
Dimension 2

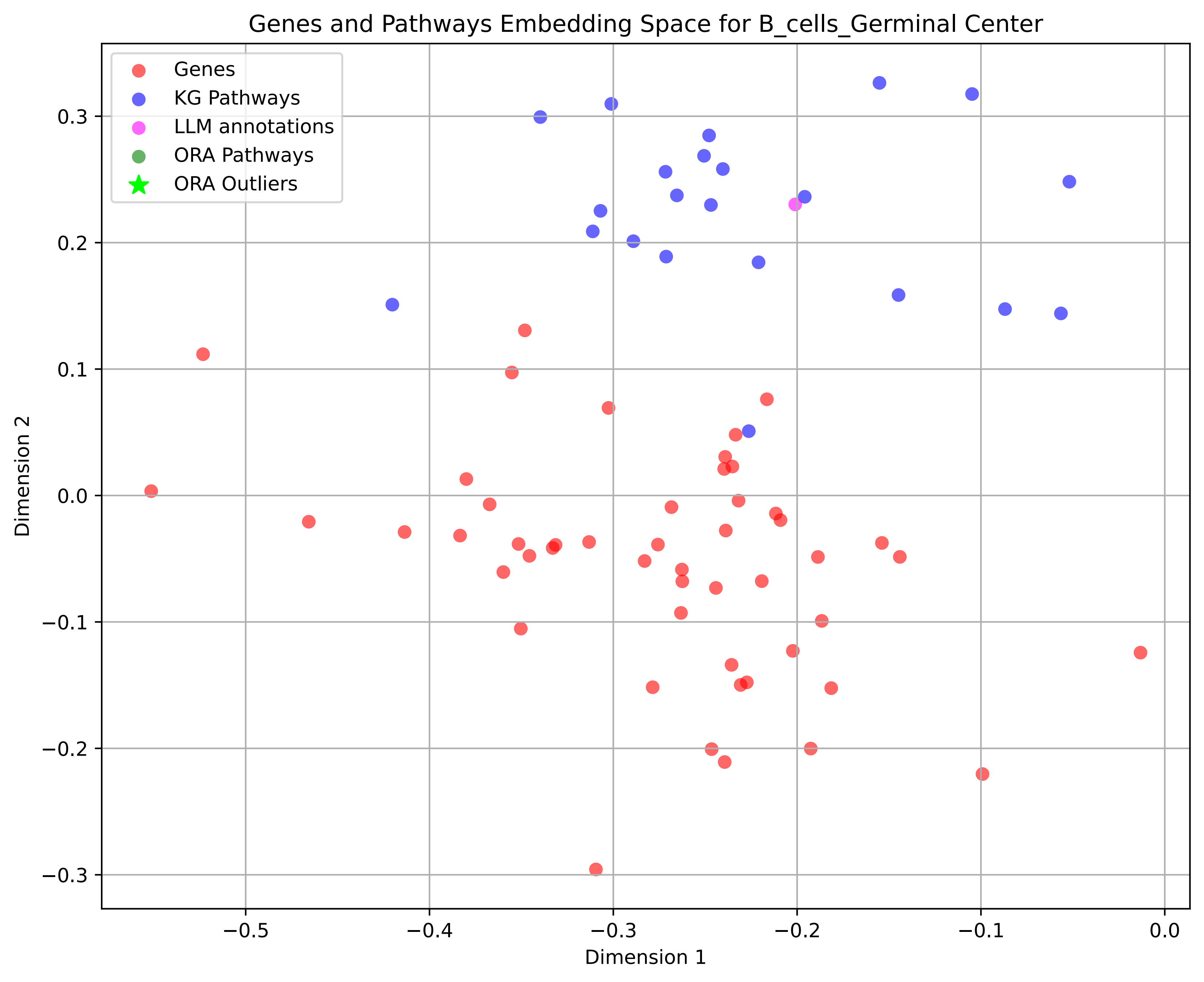
Genes and Pathways Embedding Space for B_cells_Germinal Center
Genes
KG Pathways
LLM annotations
ORA Pathways
ORA Outliers
Dimension 1
Dimension 2
−0.5
−0.4
−0.3
−0.2
−0.1
0.0
0.3
0.2
0.1
0.0
−0.1
−0.2
−0.3

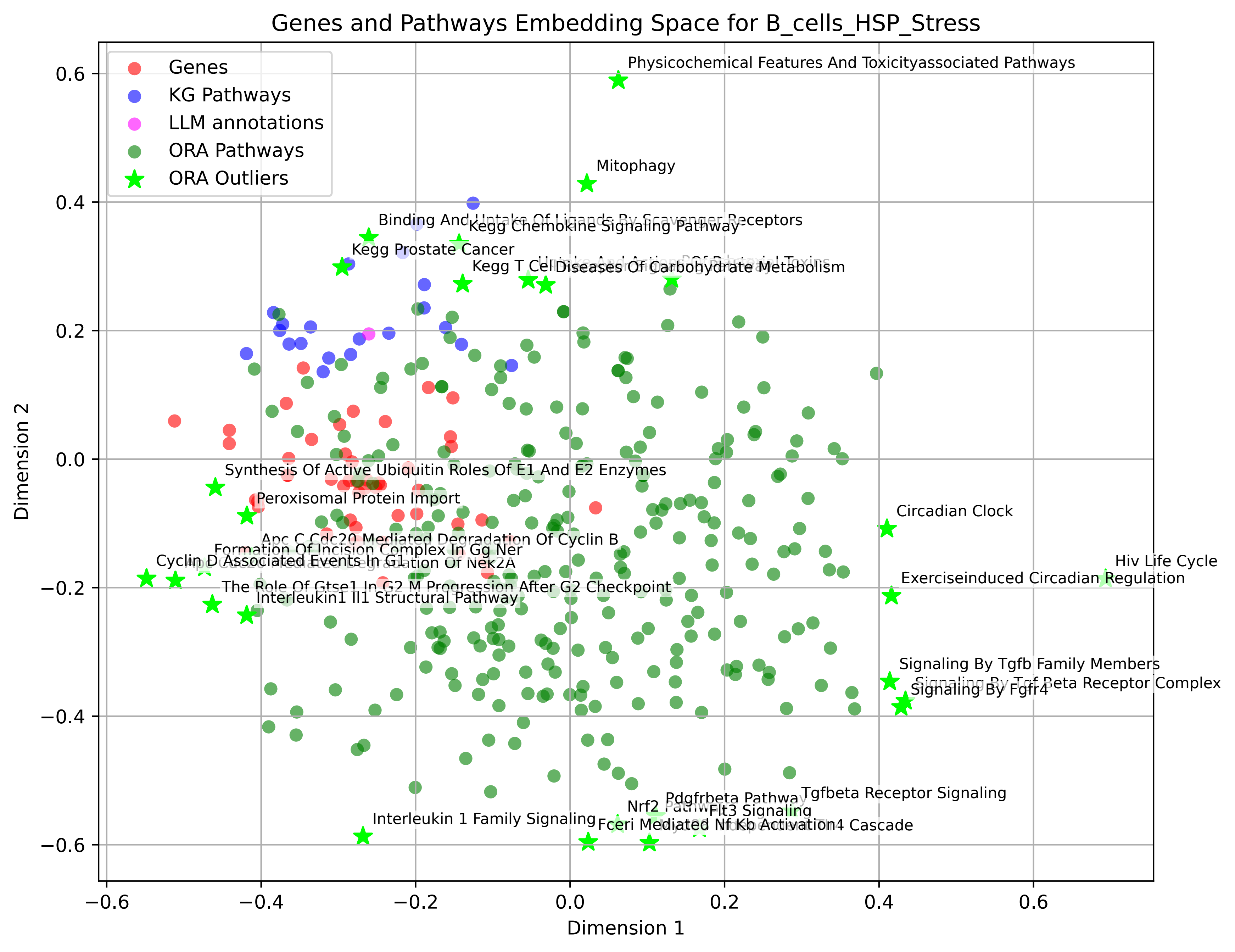

Genes and Pathways Embedding Space for B_cells_HSP_Stress
Genes
KG Pathways
LLM annotations
ORA Pathways
ORA Outliers
Physicochemical Features And Toxicityassociated Pathways
Mitophagy
Binding And Uptake Of Ligands By Scavenger Receptors
Kegg Chemokine Signaling Pathway
Kegg Prostate Cancer
Diseases Of Carbohydrate Metabolism
Synthesis Of Active Ubiquitin Roles Of E1 And E2 Enzymes
Peroxisomal Protein Import
Circadian Clock
Apc C Cdc20 Mediated Degradation Of Cyclin B
Formation Of Incision Complex In Gg Ner
Cyclin D Associated Events In G1
Hiv Life Cycle
Exerciseinduced Circadian Regulation
The Role Of Gtse1 In G2 M Progression After G2 Checkpoint
Interleukin1 Il1 Structural Pathway
Signaling By Tgfb Family Members
Signaling By Tgf Beta Receptor Complex
Signaling By Fgfr4
Tgfbeta Receptor Signaling
Pdgfrbeta Pathway
Nrf2
Flt3 Signaling
Interleukin 1 Family Signaling
Fceri Mediated Nf Kb Activation
Dimension 1
Dimension 2
0.6
0.4
0.2
0.0
−0.2
−0.4
−0.6
−0.6
−0.4
−0.2
0.0
0.2
0.4
0.6

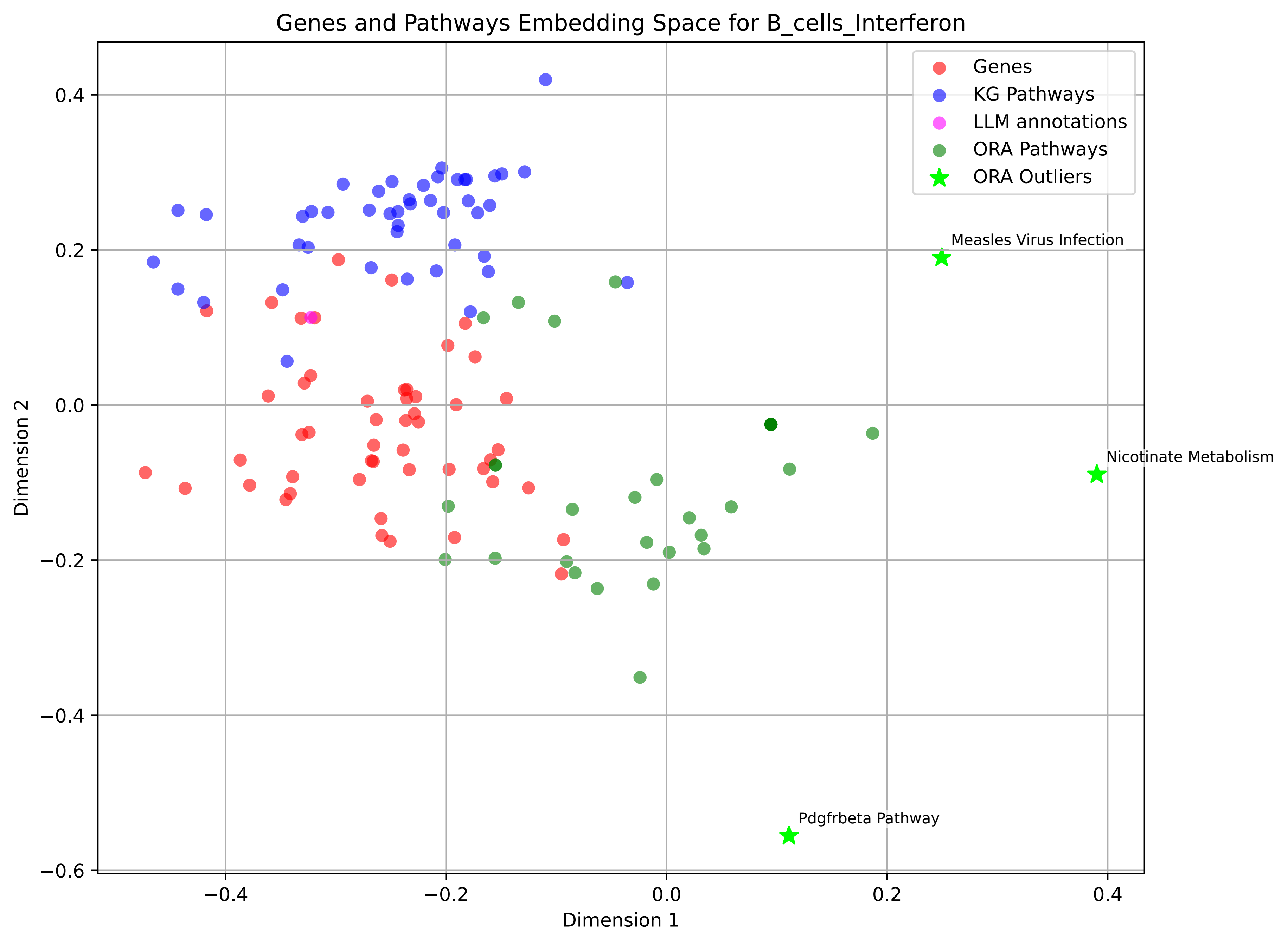

Genes and Pathways Embedding Space for B_cells_Interferon
Genes
KG Pathways
LLM annotations
ORA Pathways
ORA Outliers
Measles Virus Infection
Nicotinate Metabolism
Pdgfrbeta Pathway
Dimension 1
Dimension 2
0.4
0.2
0.0
−0.2
−0.4
−0.6
−0.4
−0.2
0.0
0.2
0.4

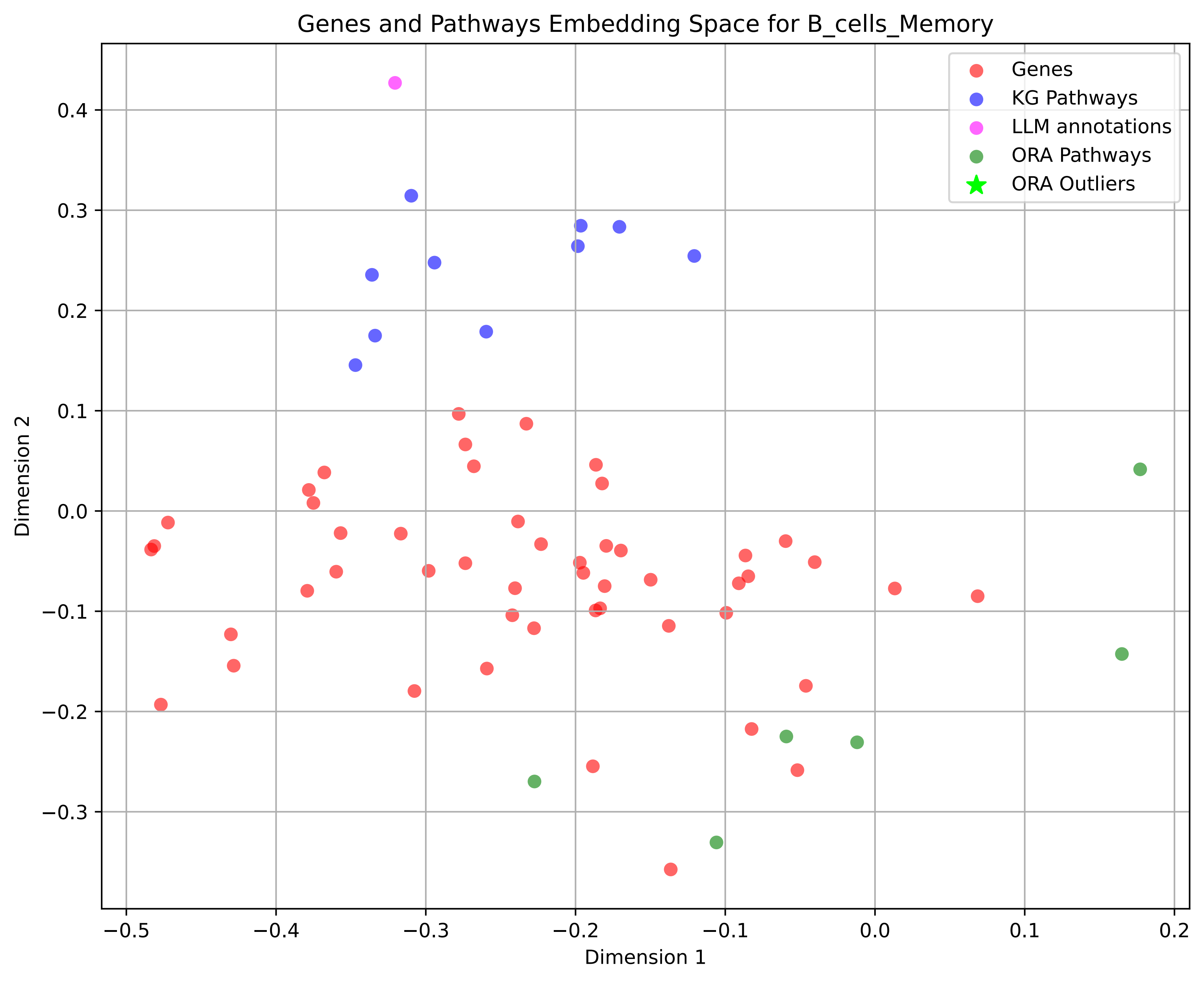
Genes and Pathways Embedding Space for B_cells_Memory
Genes
KG Pathways
LLM annotations
ORA Pathways
ORA Outliers
Dimension 1
Dimension 2
0.4
0.3
0.2
0.1
0.0
−0.1
−0.2
−0.3
−0.5
−0.4
−0.3
−0.2
−0.1
0.0
0.1
0.2

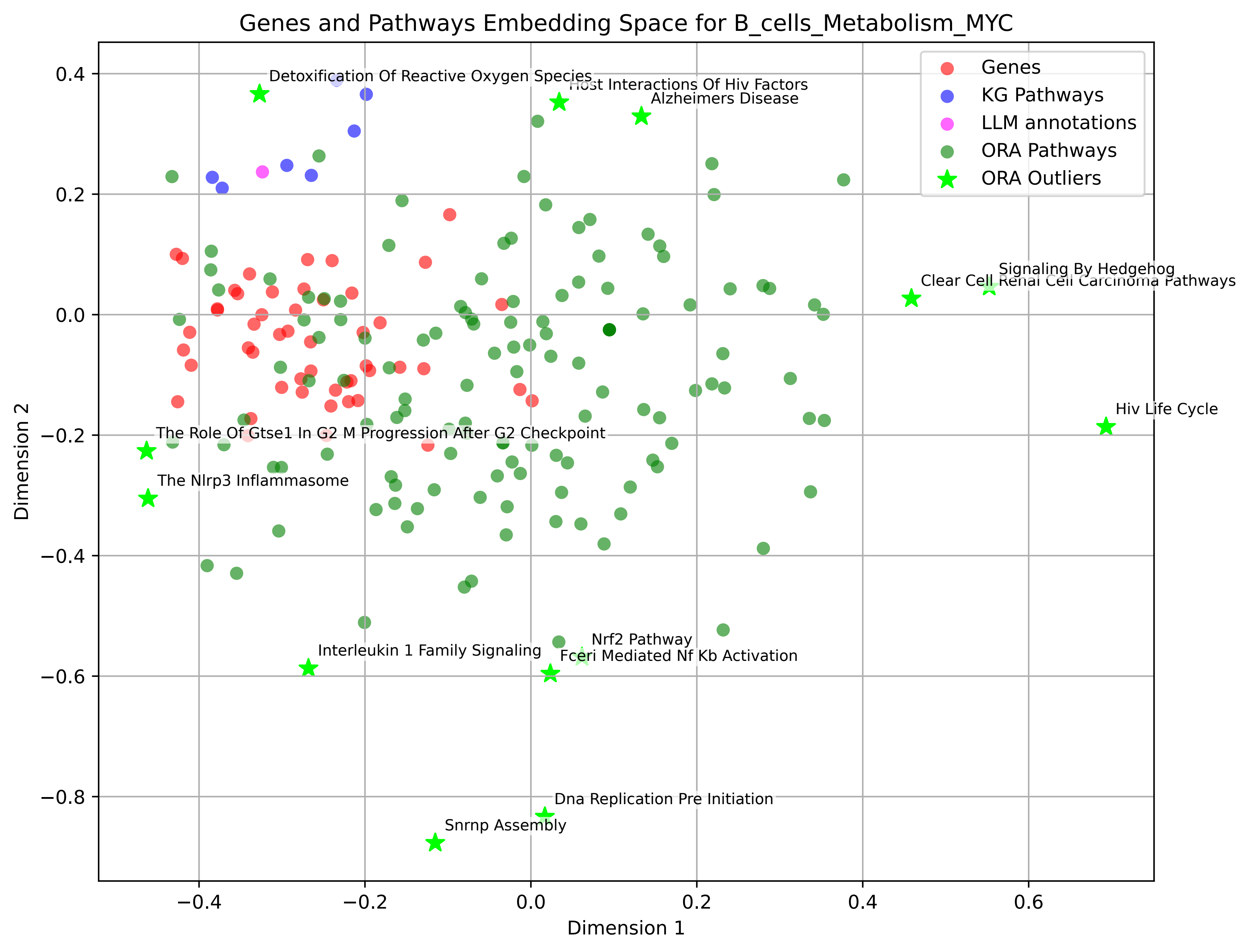

Genes and Pathways Embedding Space for B_cells_Metabolism_MYC
Genes
KG Pathways
LLM annotations
ORA Pathways
ORA Outliers
Detoxification Of Reactive Oxygen Species
Host Interactions Of Hiv Factors
Alzheimers Disease
Signaling By Hedgehog
Clear Cell Renal Cell Carcinoma Pathways
Hiv Life Cycle
The Role Of Gtse1 In G2 M Progression After G2 Checkpoint
The Nlrp3 Inflammasome
Interleukin 1 Family Signaling
Nrf2 Pathway
Fceri Mediated Nf Kb Activation
Dna Replication Pre Initiation
Snrnp Assembly
Dimension 1
Dimension 2
−0.4
−0.2
0.0
0.2
0.4
0.6
0.4
0.2
0.0
−0.2
−0.4
−0.6
−0.8

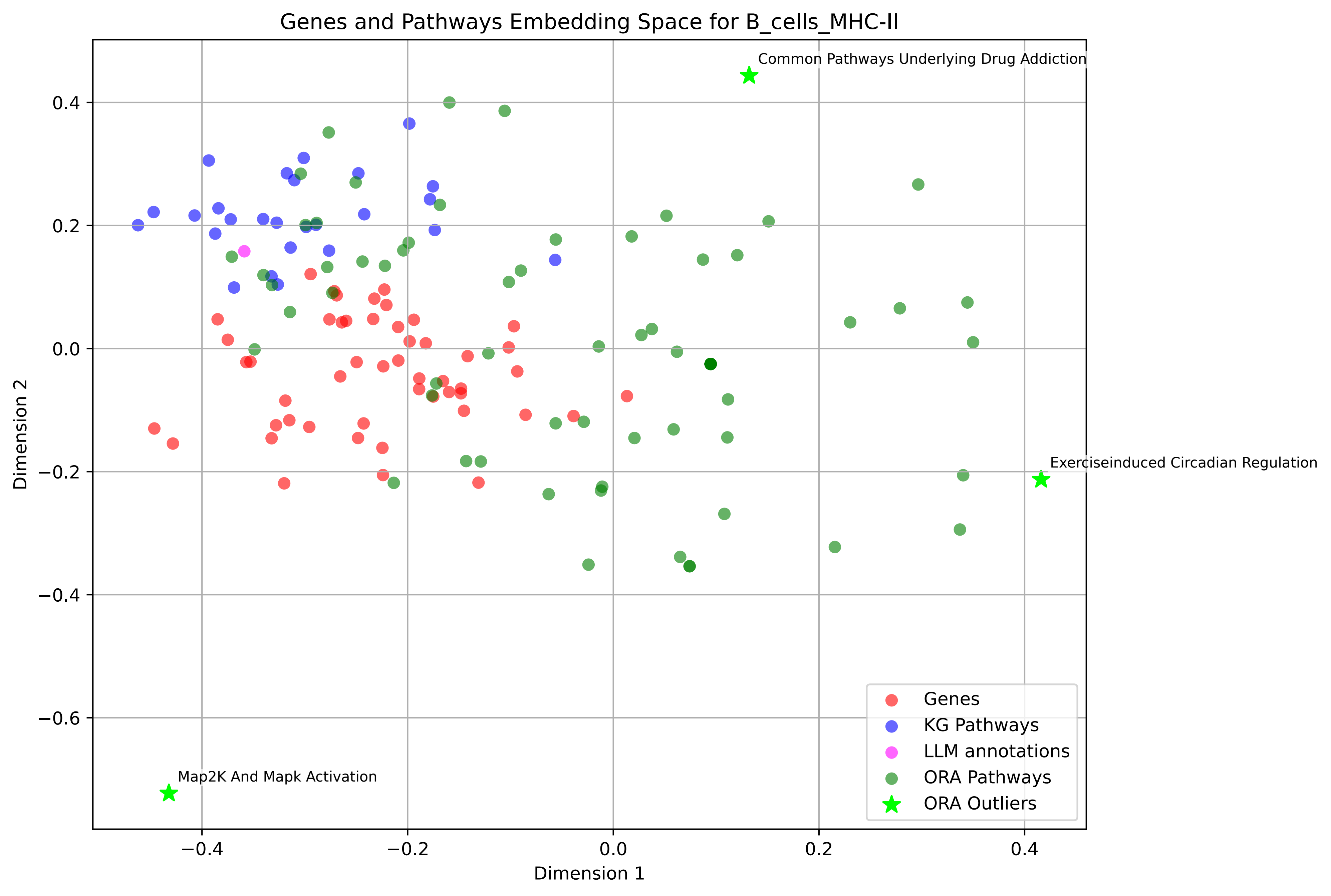

Genes and Pathways Embedding Space for B_cells_MHC-II
Common Pathways Underlying Drug Addiction
Exerciseinduced Circadian Regulation
Map2K And Mapk Activation
Dimension 1
Dimension 2
0.4
0.2
0.0
−0.2
−0.4
−0.6
−0.4
−0.2
0.0
0.2
0.4
Genes
KG Pathways
LLM annotations
ORA Pathways
ORA Outliers

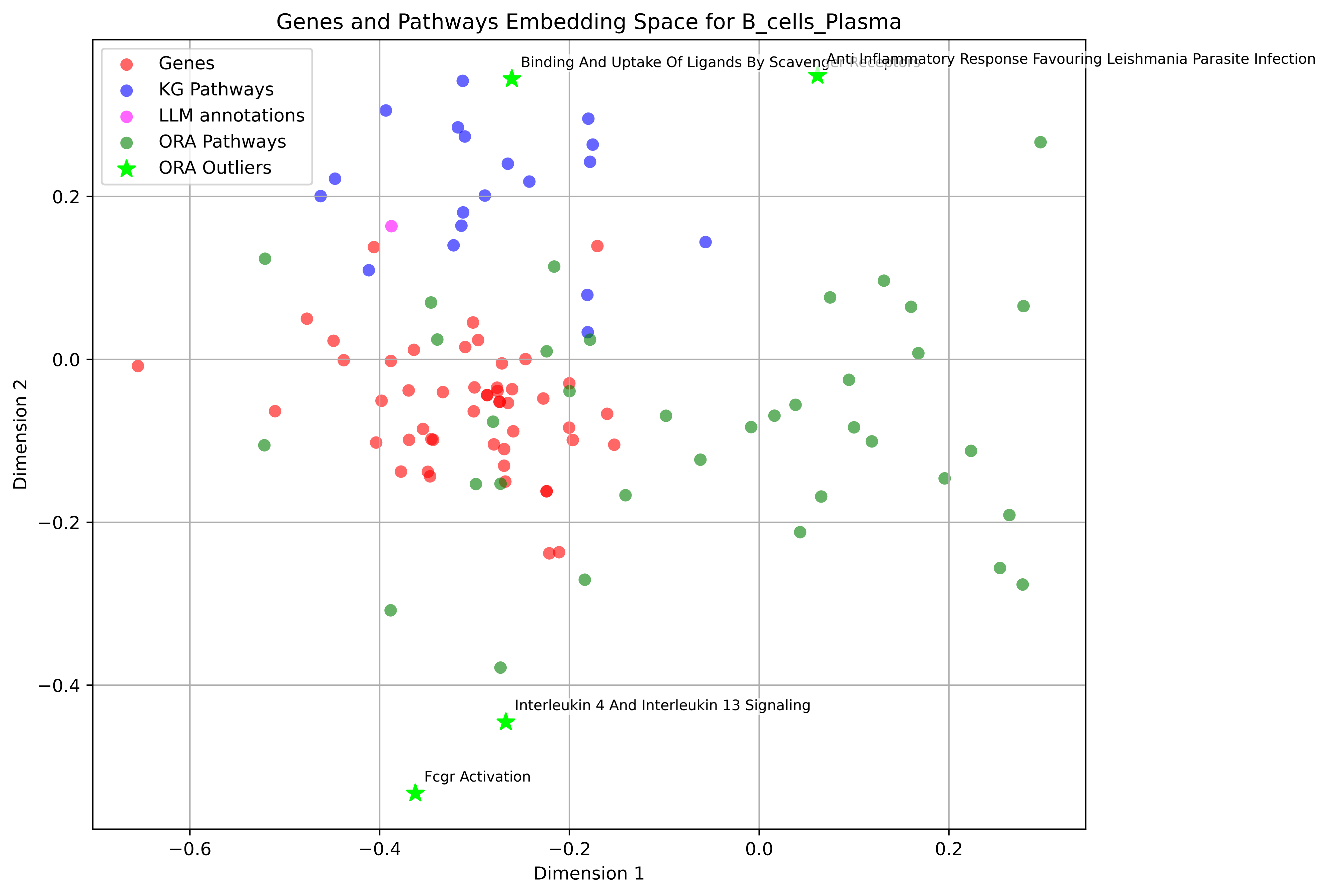

Genes and Pathways Embedding Space for B_cells_Plasma
Genes
KG Pathways
LLM annotations
ORA Pathways
ORA Outliers
Binding And Uptake Of Ligands By Scavenger Receptors
Anti Inflammatory Response Favouring Leishmania Parasite Infection
Interleukin 4 And Interleukin 13 Signaling
Fcgr Activation
0.2
0.0
−0.2
−0.4
−0.6
−0.4
−0.2
0.0
0.2
Dimension 1
Dimension 2

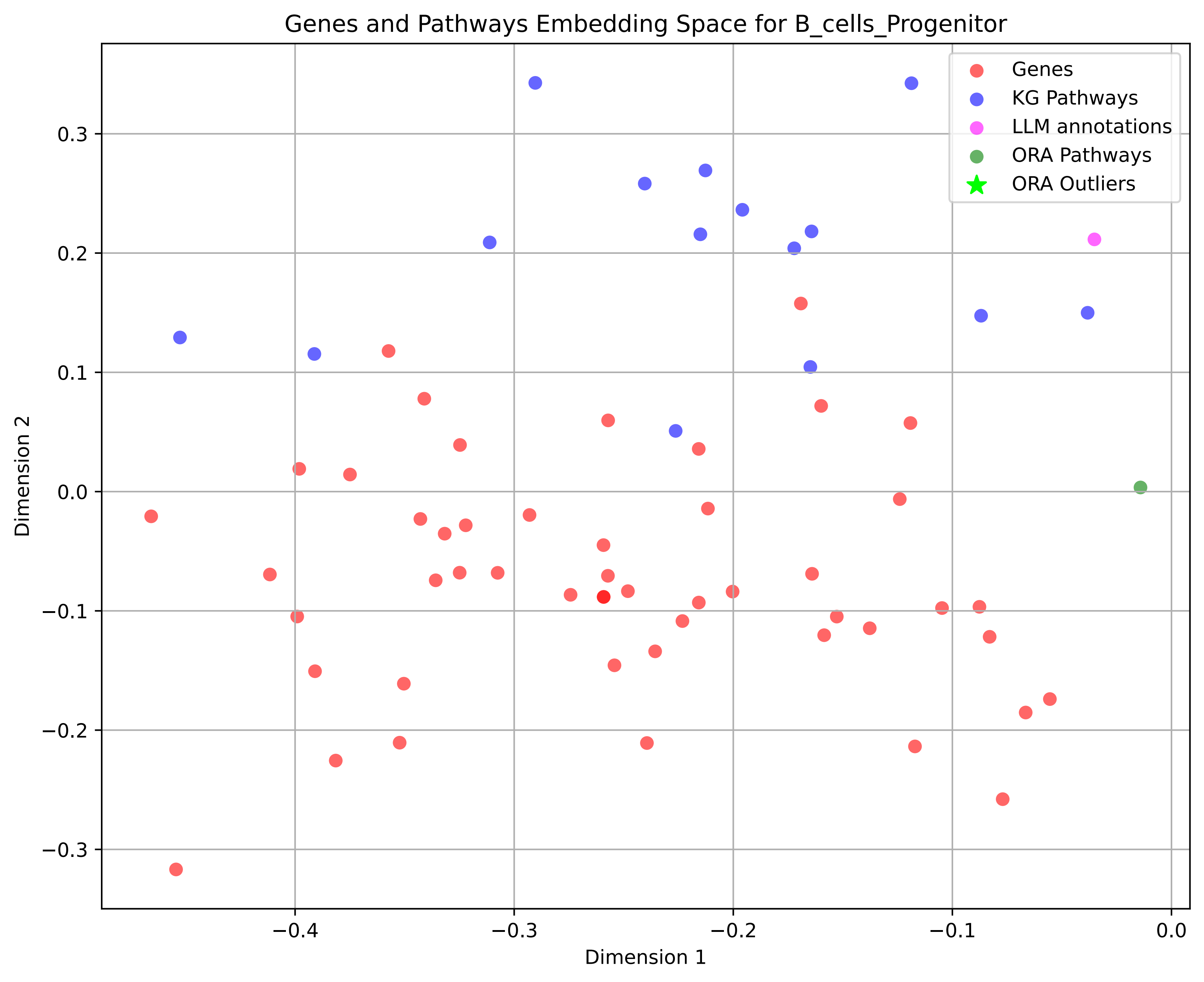

Genes and Pathways Embedding Space for B_cells_Progenitor
Genes
KG Pathways
LLM annotations
ORA Pathways
ORA Outliers
Dimension 1
Dimension 2
−0.4
−0.3
−0.2
−0.1
0.0
0.3
0.2
0.1
0.0
−0.1
−0.2
−0.3

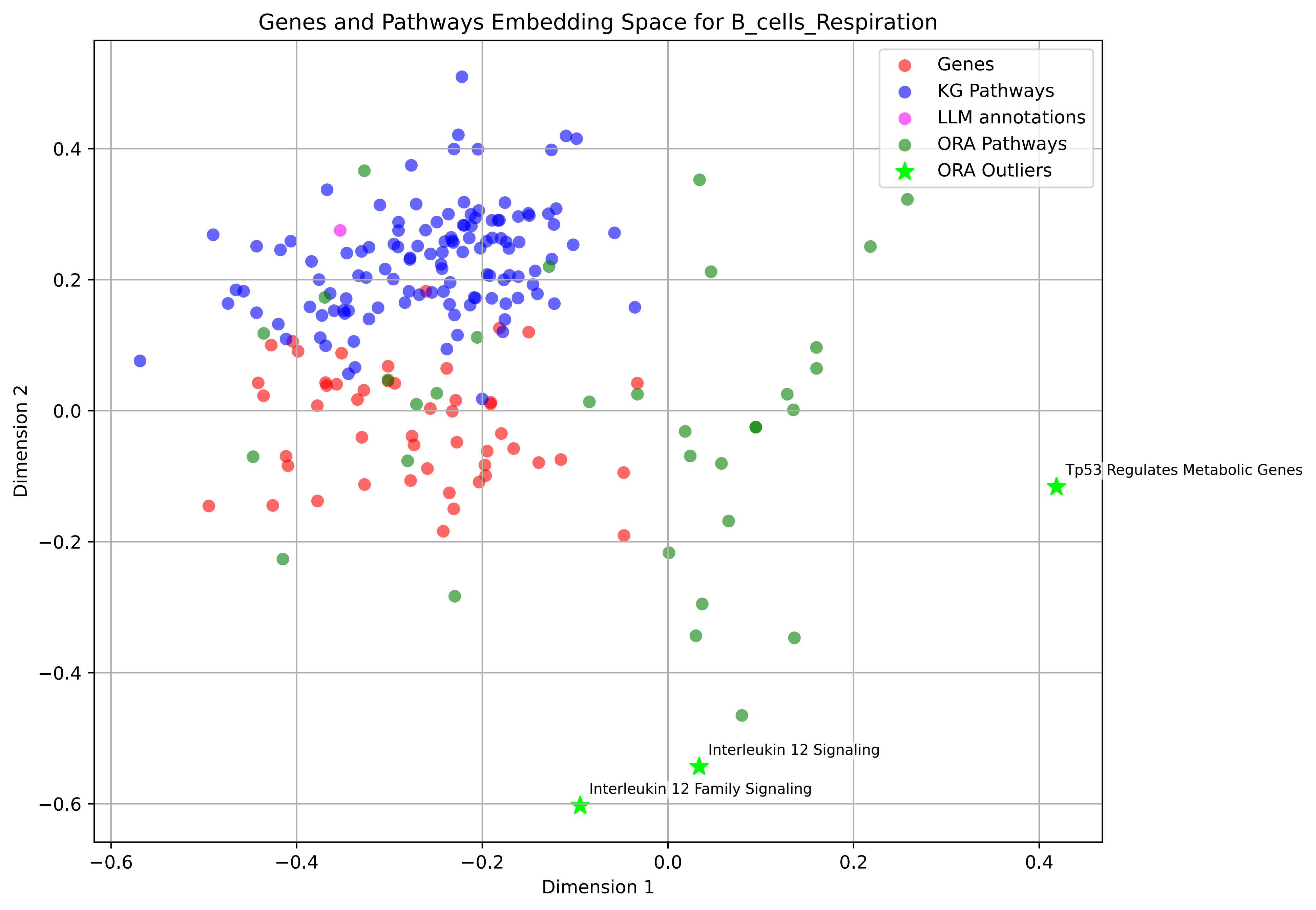

Genes and Pathways Embedding Space for B_cells_Respiration
Genes
KG Pathways
LLM annotations
ORA Pathways
ORA Outliers
Tp53 Regulates Metabolic Genes
Interleukin 12 Signaling
Interleukin 12 Family Signaling
Dimension 1
Dimension 2
0.4
0.2
0.0
−0.2
−0.4
−0.6
−0.6
−0.4
−0.2
0.0
0.2
0.4

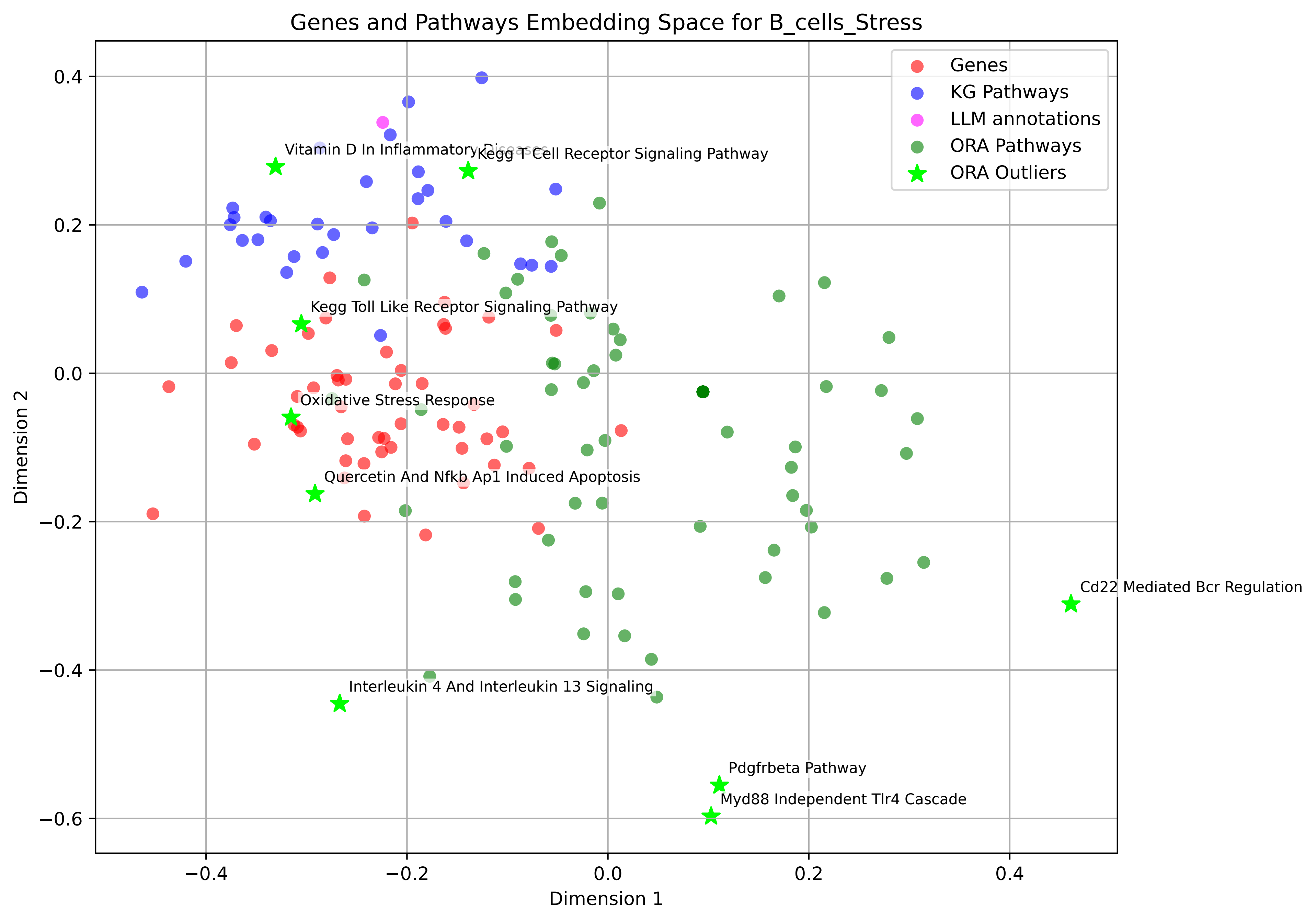
Genes and Pathways Embedding Space for B_cells_Stress
Dimension 1
Dimension 2
−0.4
−0.2
0.0
0.2
0.4
−0.6
Genes
KG Pathways
LLM annotations
ORA Pathways
ORA Outliers
Vitamin D In Inflammatory Diseases
Kegg T Cell Receptor Signaling Pathway
Kegg Toll Like Receptor Signaling Pathway
Oxidative Stress Response
Quercetin And Nfkb Ap1 Induced Apoptosis
Interleukin 4 And Interleukin 13 Signaling
Cd22 Mediated Bcr Regulation
Pdgfrbeta Pathway
Myd88 Independent Tlr4 Cascade

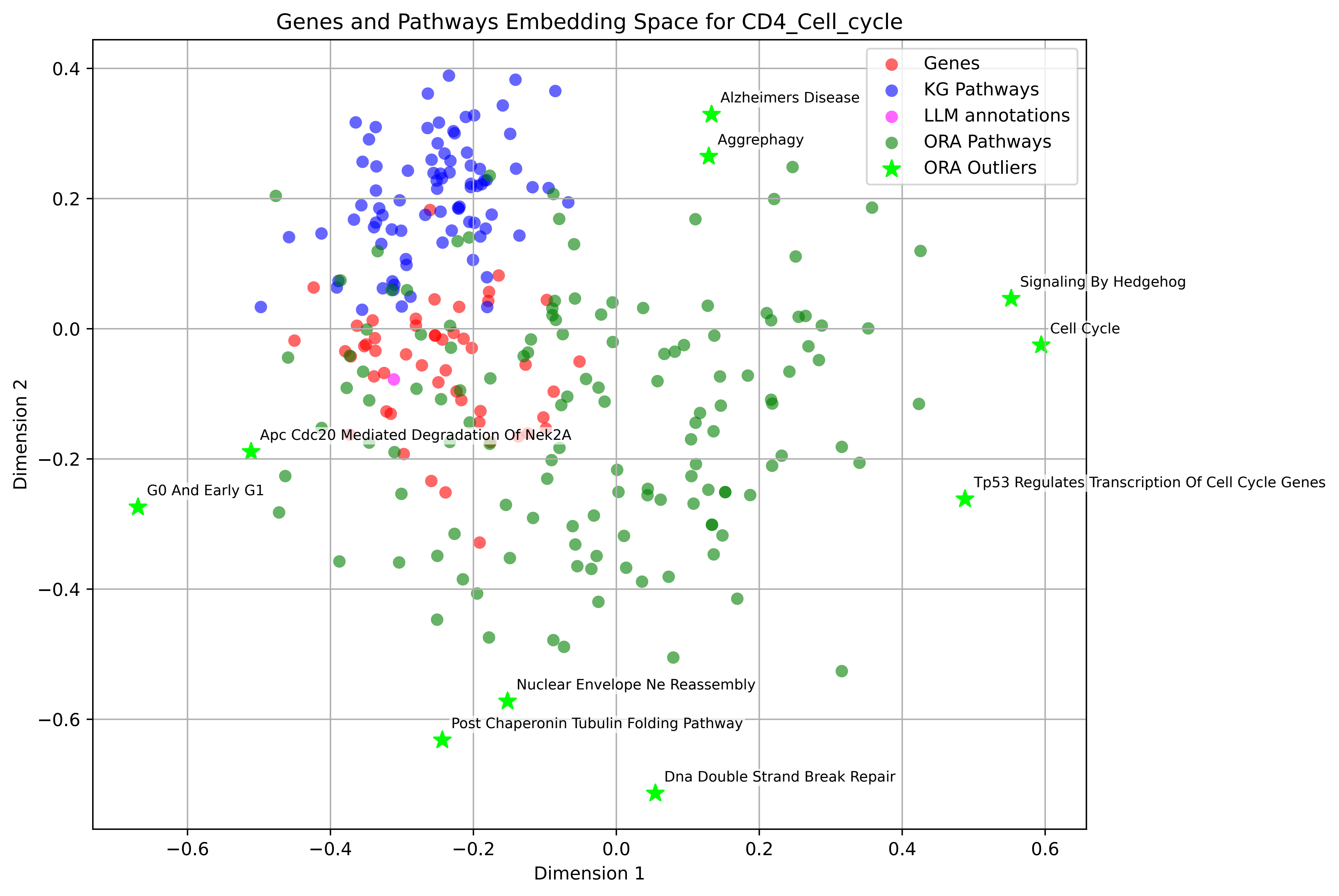
Genes and Pathways Embedding Space for CD4_Cell_cycle
Genes
KG Pathways
LLM annotations
ORA Pathways
ORA Outliers
Alzheimers Disease
Aggrephagy
Signaling By Hedgehog
Cell Cycle
Tp53 Regulates Transcription Of Cell Cycle Genes
Apc Cdc20 Mediated Degradation Of Nek2A
G0 And Early G1
Nuclear Envelope Ne Reassembly
Post Chaperonin Tubulin Folding Pathway
Dna Double Strand Break Repair
Dimension 1
Dimension 2
0.4
0.2
0.0
−0.2
−0.4
−0.6
−0.6
−0.4
−0.2
0.0
0.2
0.4
0.6

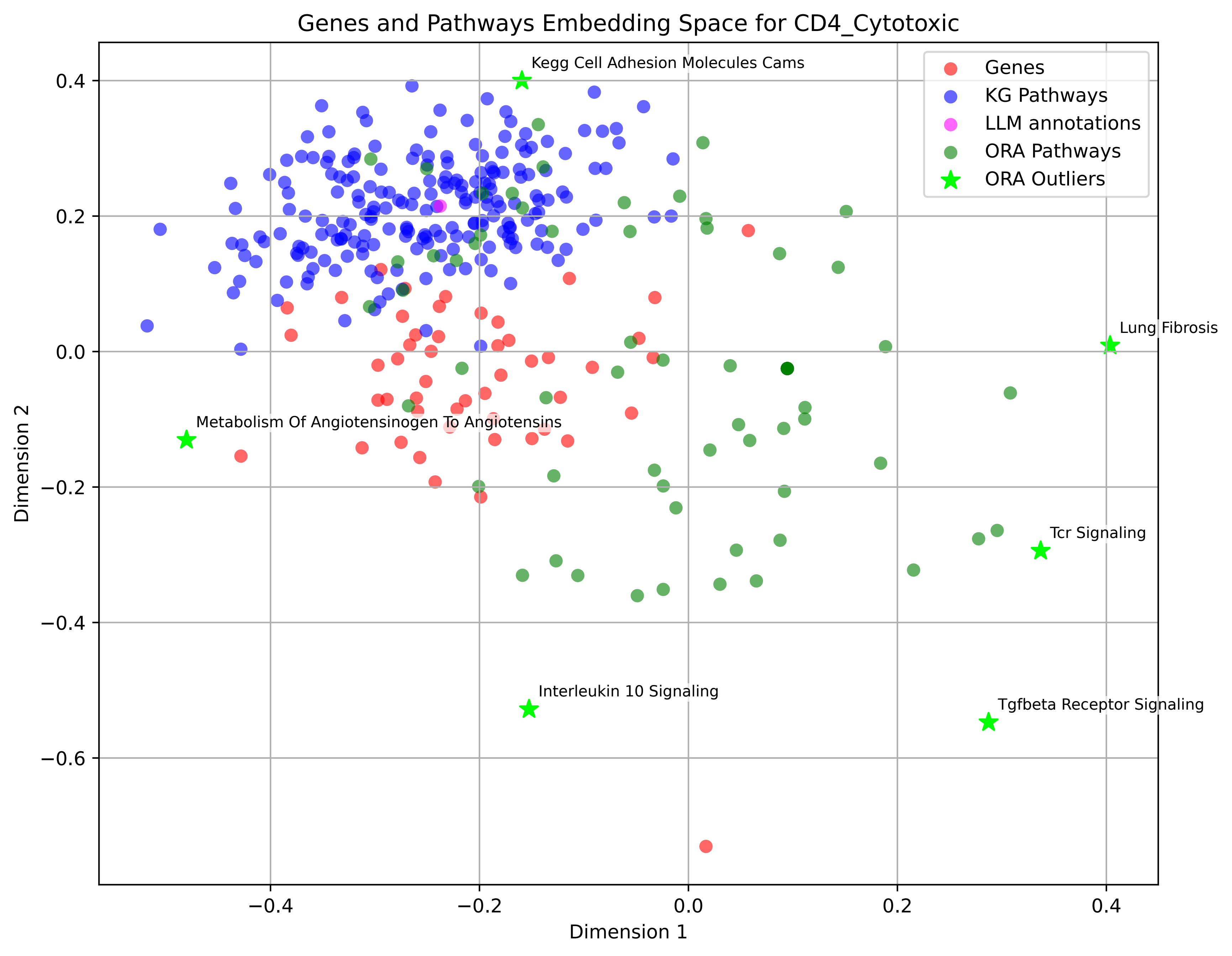
Genes and Pathways Embedding Space for CD4_Cytotoxic
Kegg Cell Adhesion Molecules Cams
Genes
KG Pathways
LLM annotations
ORA Pathways
ORA Outliers
Lung Fibrosis
Metabolism Of Angiotensinogen To Angiotensins
Tcr Signaling
Interleukin 10 Signaling
Tgfbeta Receptor Signaling
Dimension 1
Dimension 2
0.4
0.2
0.0
−0.2
−0.4
−0.6
−0.4
−0.2
0.0
0.2
0.4

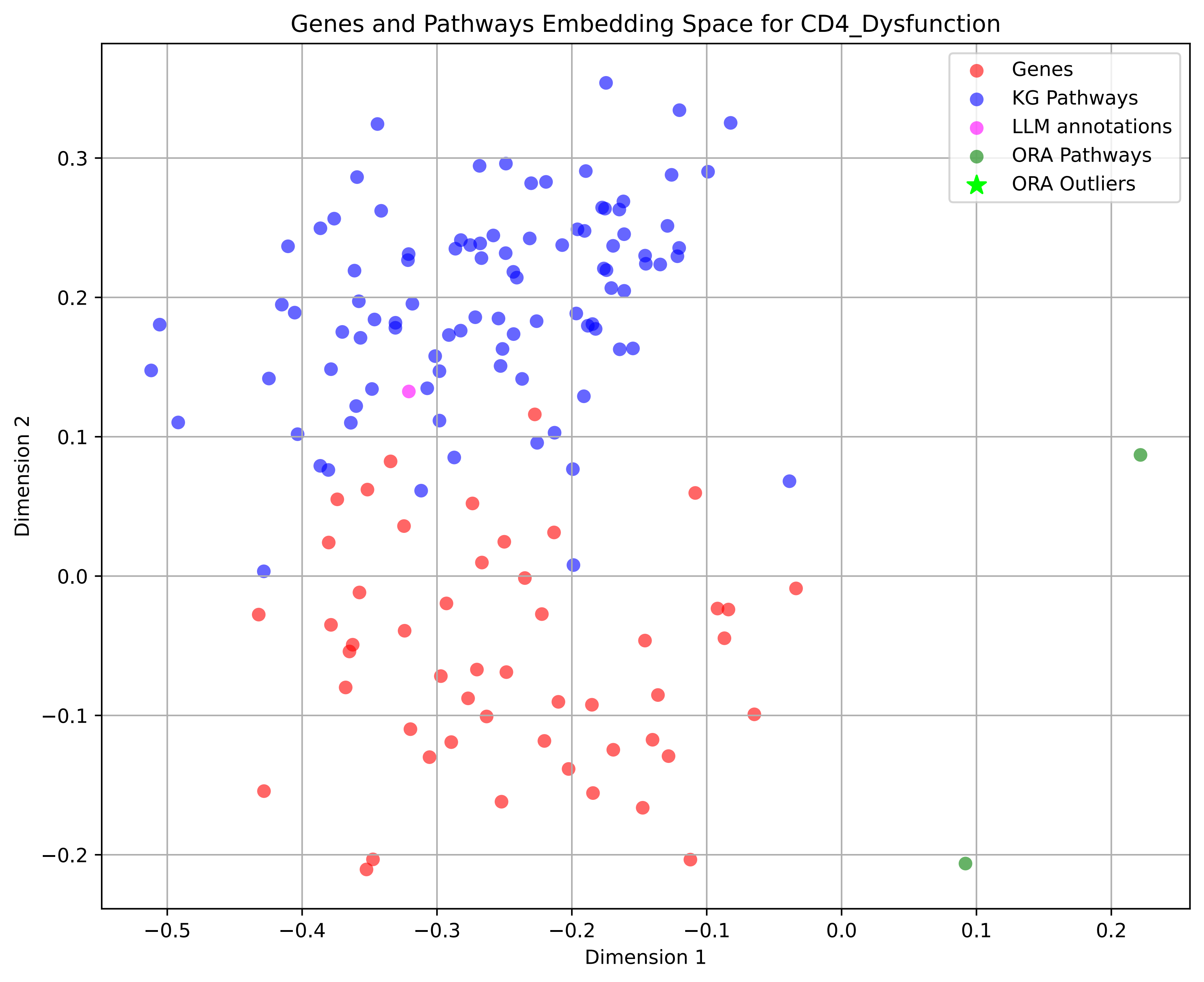
Genes and Pathways Embedding Space for CD4_Dysfunction
Genes
KG Pathways
LLM annotations
ORA Pathways
ORA Outliers
Dimension 1
Dimension 2
−0.5
−0.4
−0.3
−0.2
−0.1
0.0
0.1
0.2
0.3
−0.2
−0.1

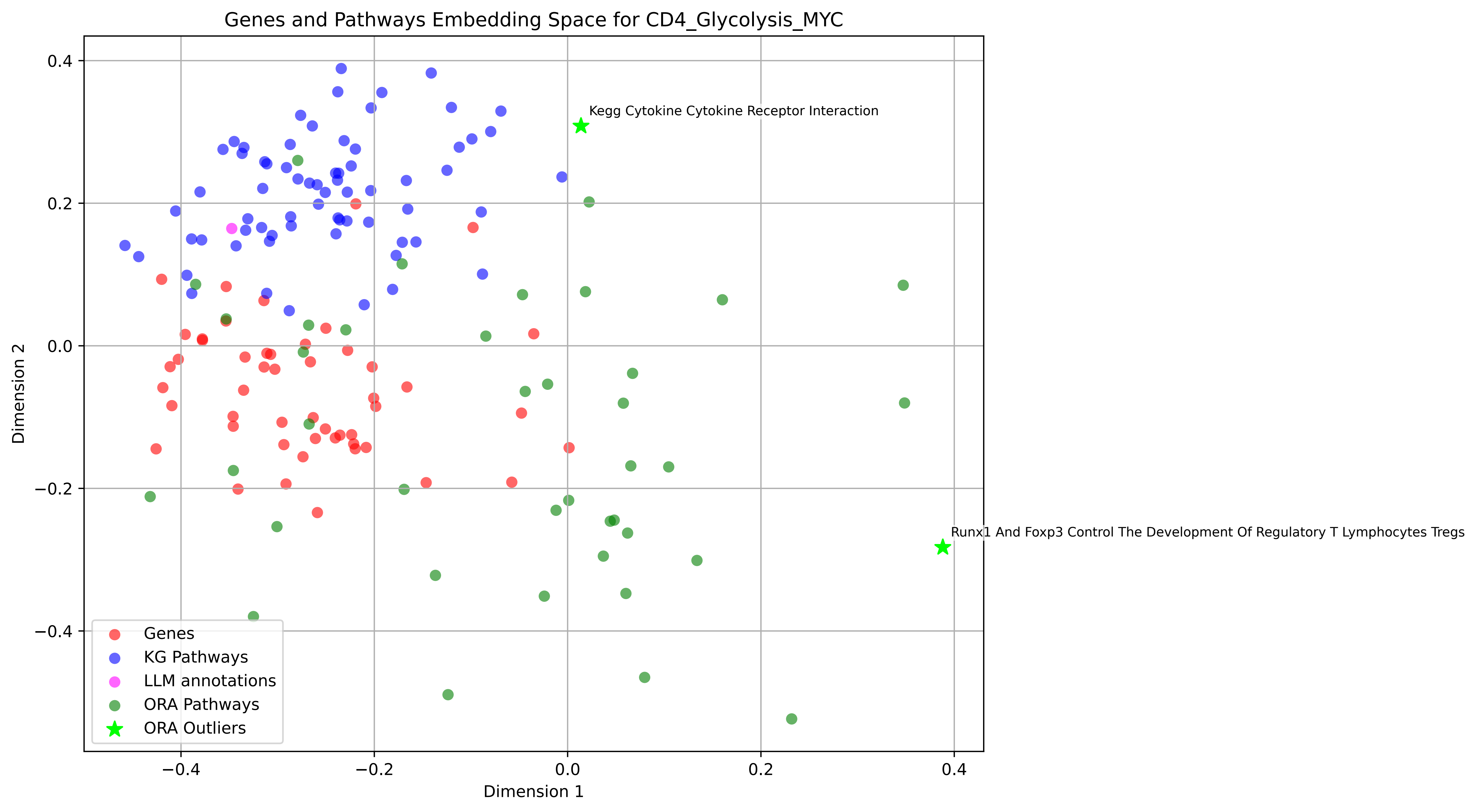
Genes and Pathways Embedding Space for CD4_Glycolysis_MYC
Kegg Cytokine Cytokine Receptor Interaction
Runx1 And Foxp3 Control The Development Of Regulatory T Lymphocytes Tregs
Dimension 1
Dimension 2
0.4
0.2
0.0
−0.2
−0.4
−0.4
−0.2
0.0
0.2
0.4
Genes
KG Pathways
LLM annotations
ORA Pathways
ORA Outliers

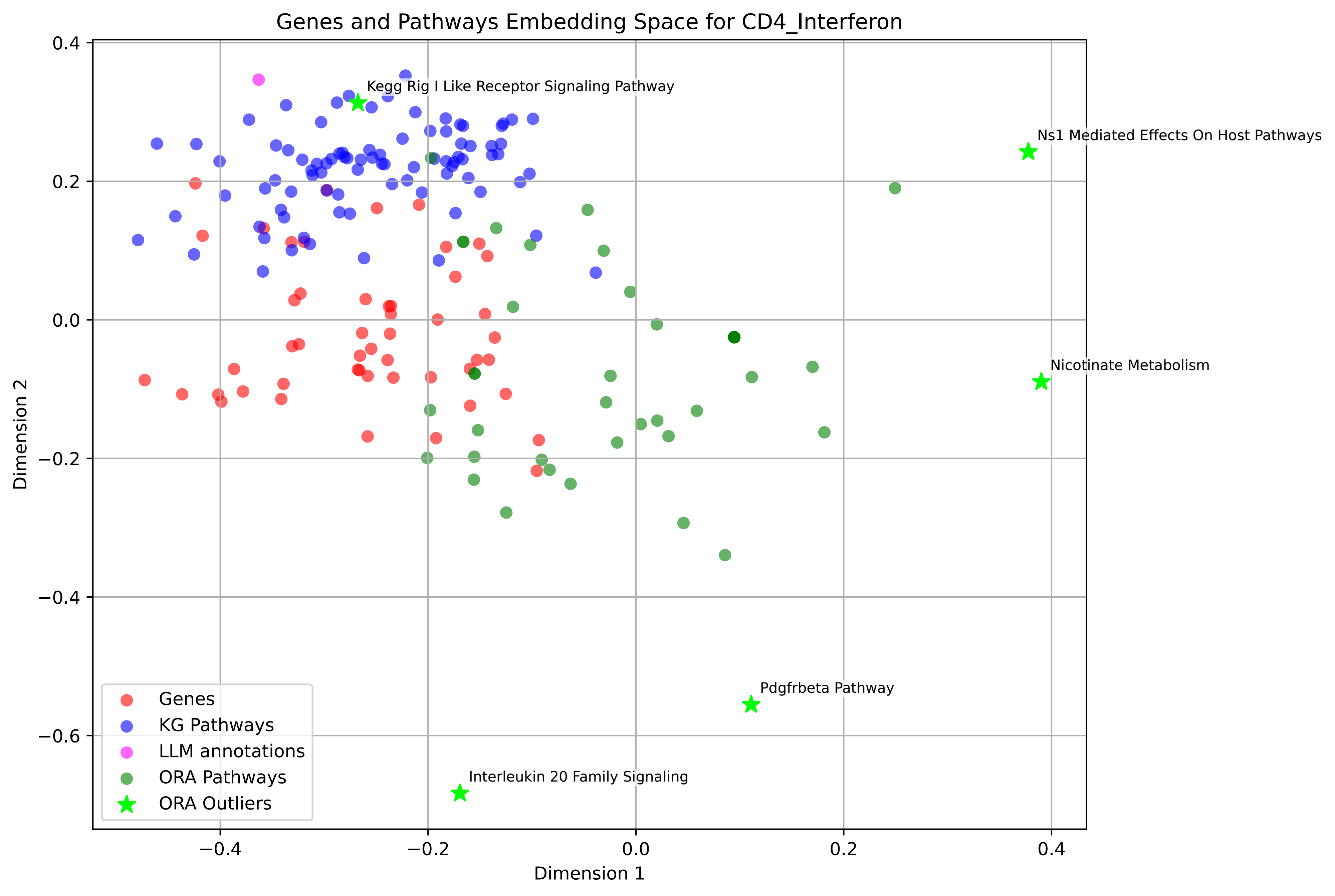

Genes and Pathways Embedding Space for CD4_Interferon
Kegg Rig I Like Receptor Signaling Pathway
Ns1 Mediated Effects On Host Pathways
Nicotinate Metabolism
Pdgfrbeta Pathway
Interleukin 20 Family Signaling
Genes
KG Pathways
LLM annotations
ORA Pathways
ORA Outliers
Dimension 1
Dimension 2
0.4
0.2
0.0
−0.2
−0.4
−0.6
−0.4
−0.2
0.0
0.2
0.4

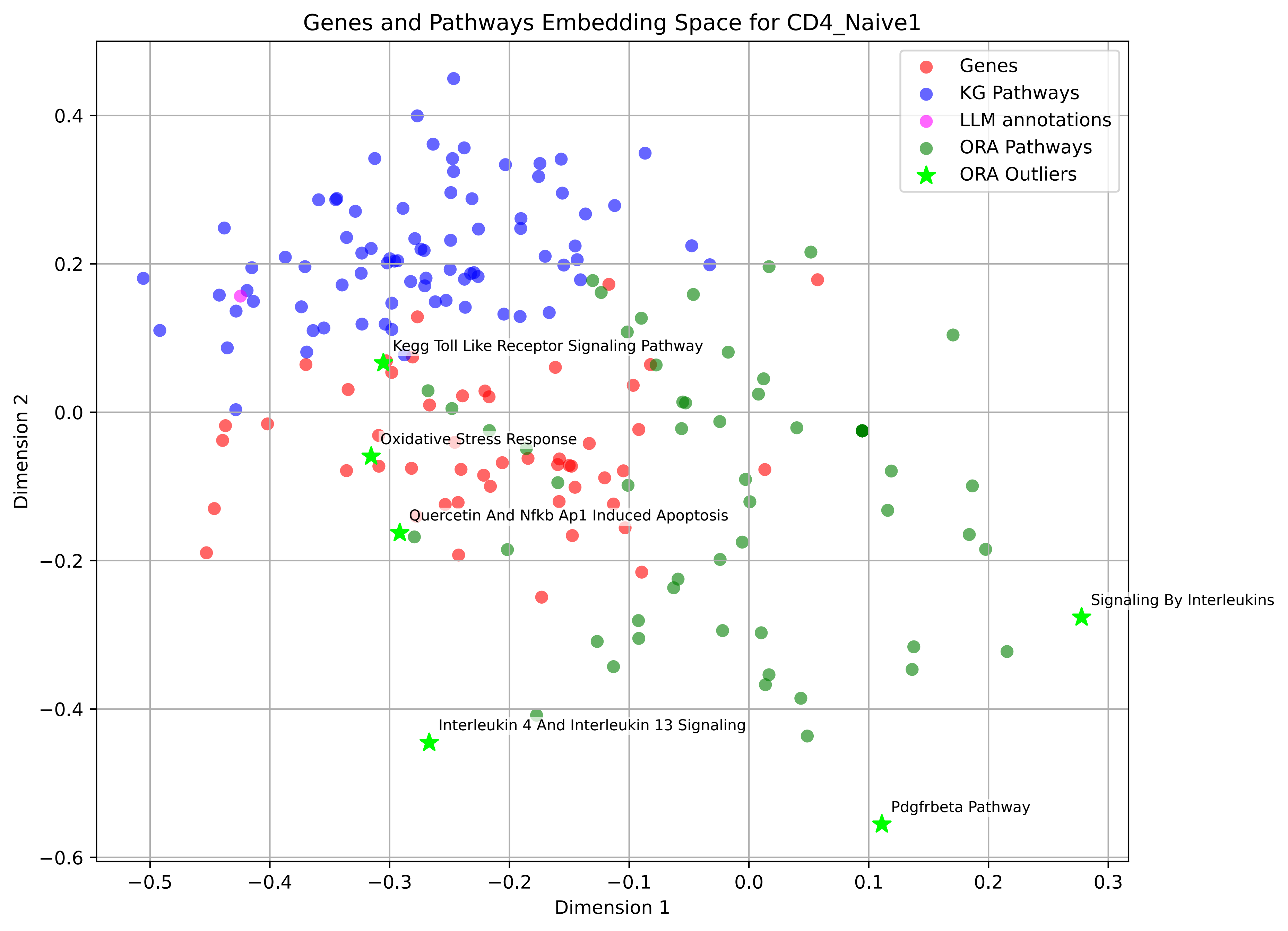
Genes and Pathways Embedding Space for CD4_Naive1
Genes
KG Pathways
LLM annotations
ORA Pathways
ORA Outliers
Kegg Toll Like Receptor Signaling Pathway
Oxidative Stress Response
Quercetin And Nfkb Ap1 Induced Apoptosis
Signaling By Interleukins
Interleukin 4 And Interleukin 13 Signaling
Pdgfrbeta Pathway
Dimension 1
Dimension 2
0.4
0.2
0.0
−0.2
−0.4
−0.6
−0.5
−0.4
−0.3
−0.2
−0.1
0.0
0.1
0.2
0.3

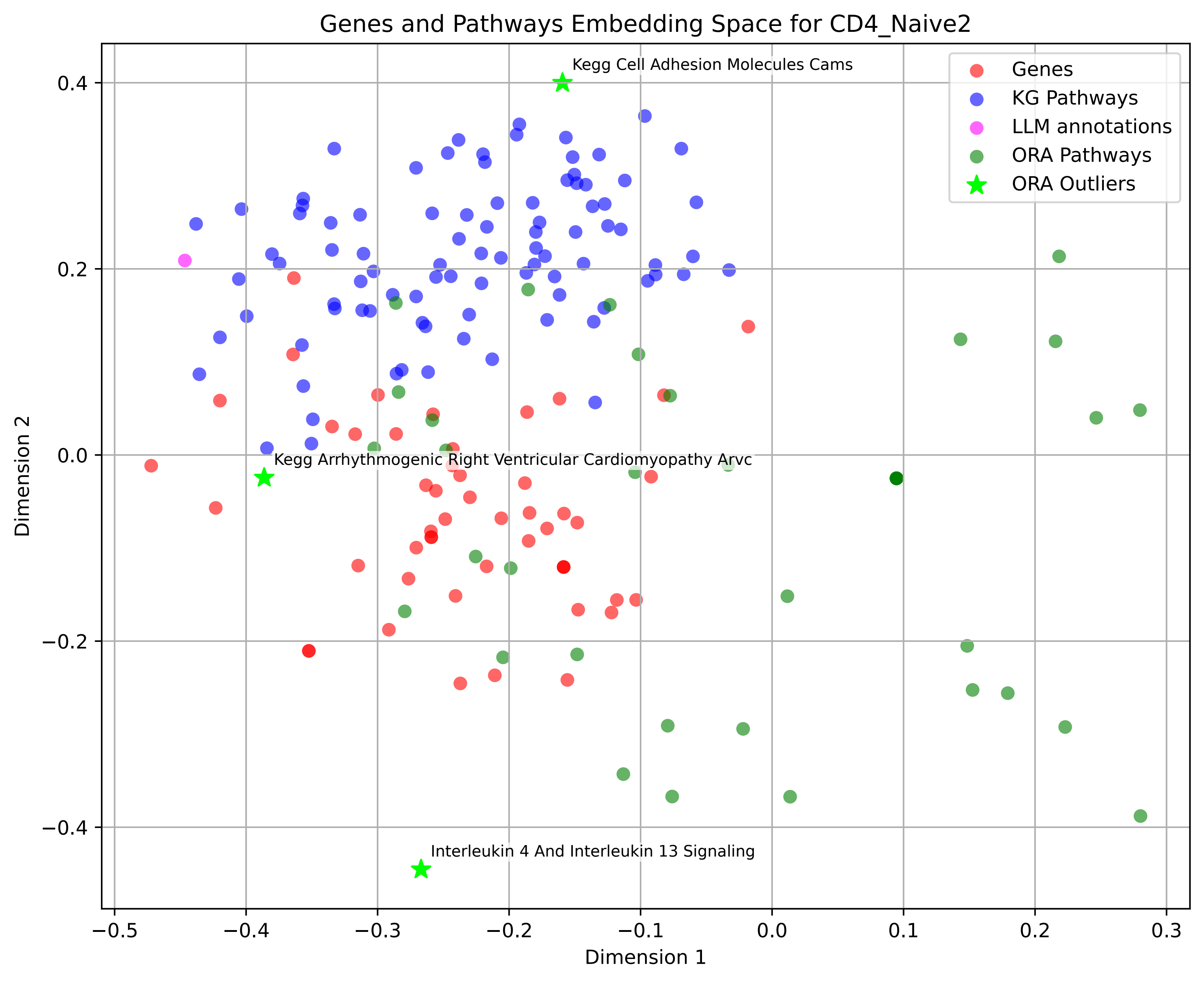

Genes and Pathways Embedding Space for CD4_Naive2
Kegg Cell Adhesion Molecules Cams
Kegg Arrhythmogenic Right Ventricular Cardiomyopathy Arvc
Interleukin 4 And Interleukin 13 Signaling
Genes
KG Pathways
LLM annotations
ORA Pathways
ORA Outliers
Dimension 1
Dimension 2
0.4
0.2
0.0
−0.2
−0.4
−0.5
−0.4
−0.3
−0.2
−0.1
0.0
0.1
0.2
0.3

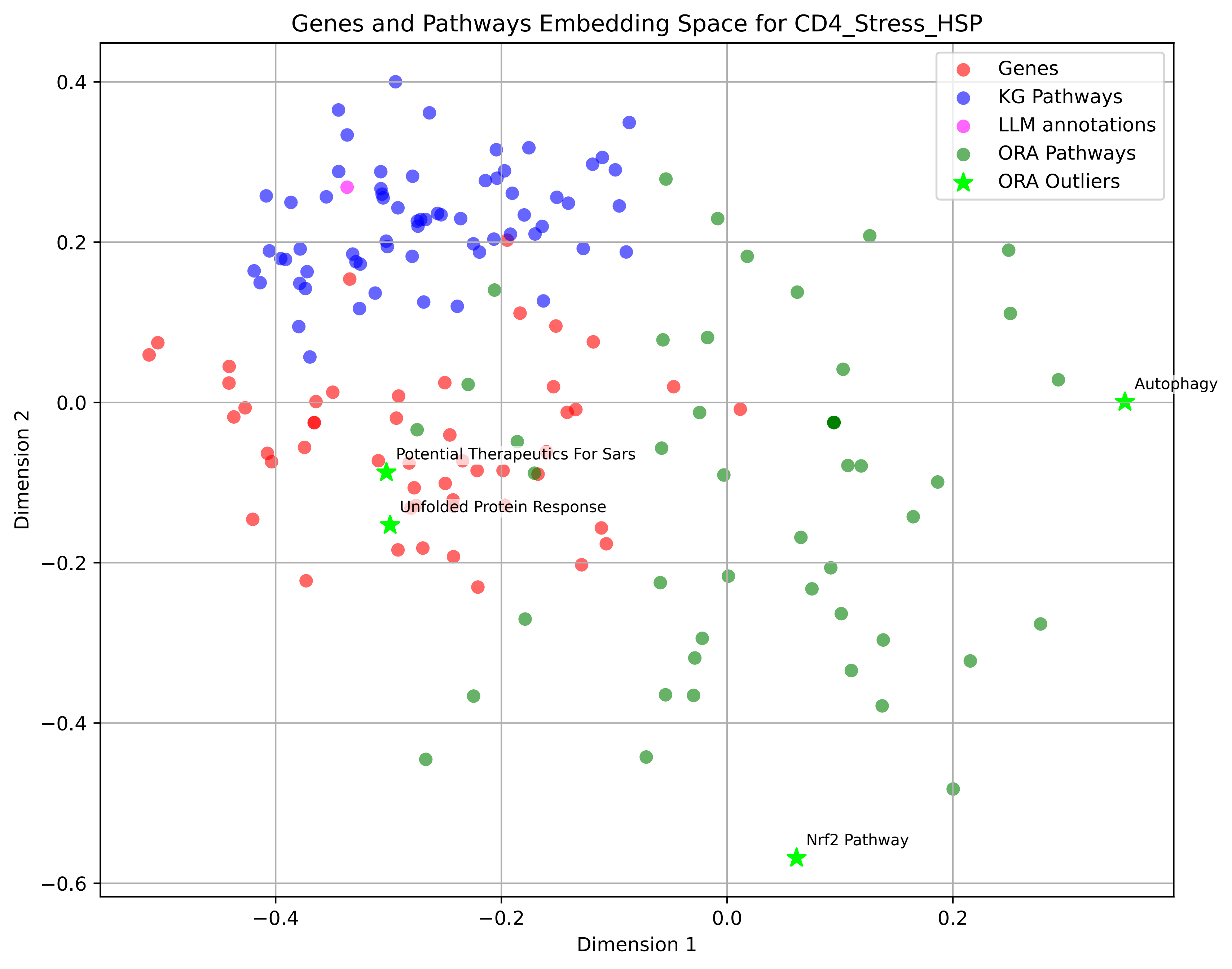

Genes and Pathways Embedding Space for CD4_Stress_HSP
Genes
KG Pathways
LLM annotations
ORA Pathways
ORA Outliers
Autophagy
Potential Therapeutics For Sars
Unfolded Protein Response
Nrf2 Pathway
Dimension 1
Dimension 2
0.4
0.2
0.0
−0.2
−0.4
−0.6
−0.4
−0.2
0.0
0.2

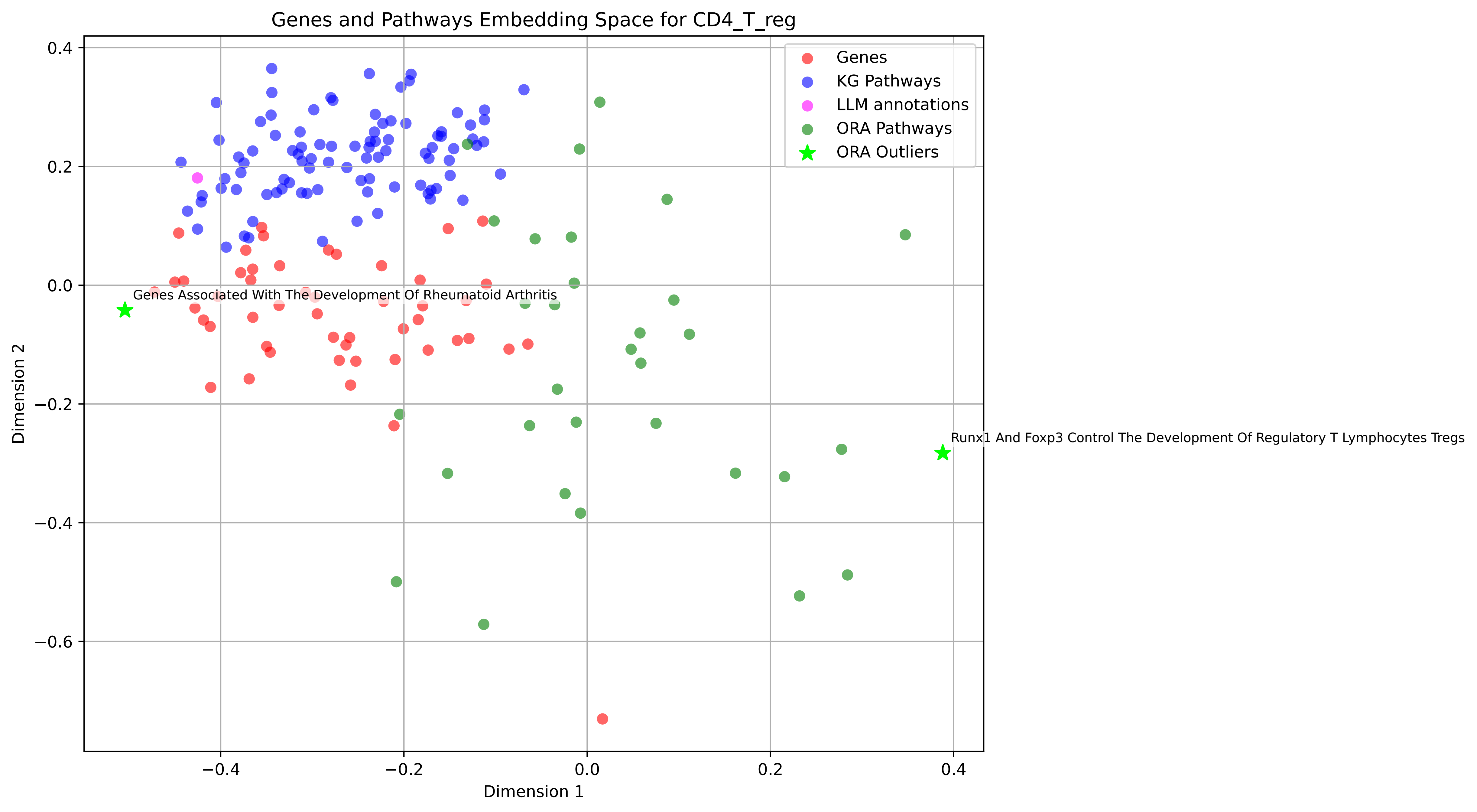
Genes and Pathways Embedding Space for CD4_T_reg
Genes
KG Pathways
LLM annotations
ORA Pathways
ORA Outliers
Genes Associated With The Development Of Rheumatoid Arthritis
Runx1 And Foxp3 Control The Development Of Regulatory T Lymphocytes Tregs
Dimension 1
Dimension 2
0.4
0.2
0.0
−0.2
−0.4
−0.6
−0.4
−0.2
0.0
0.2
0.4

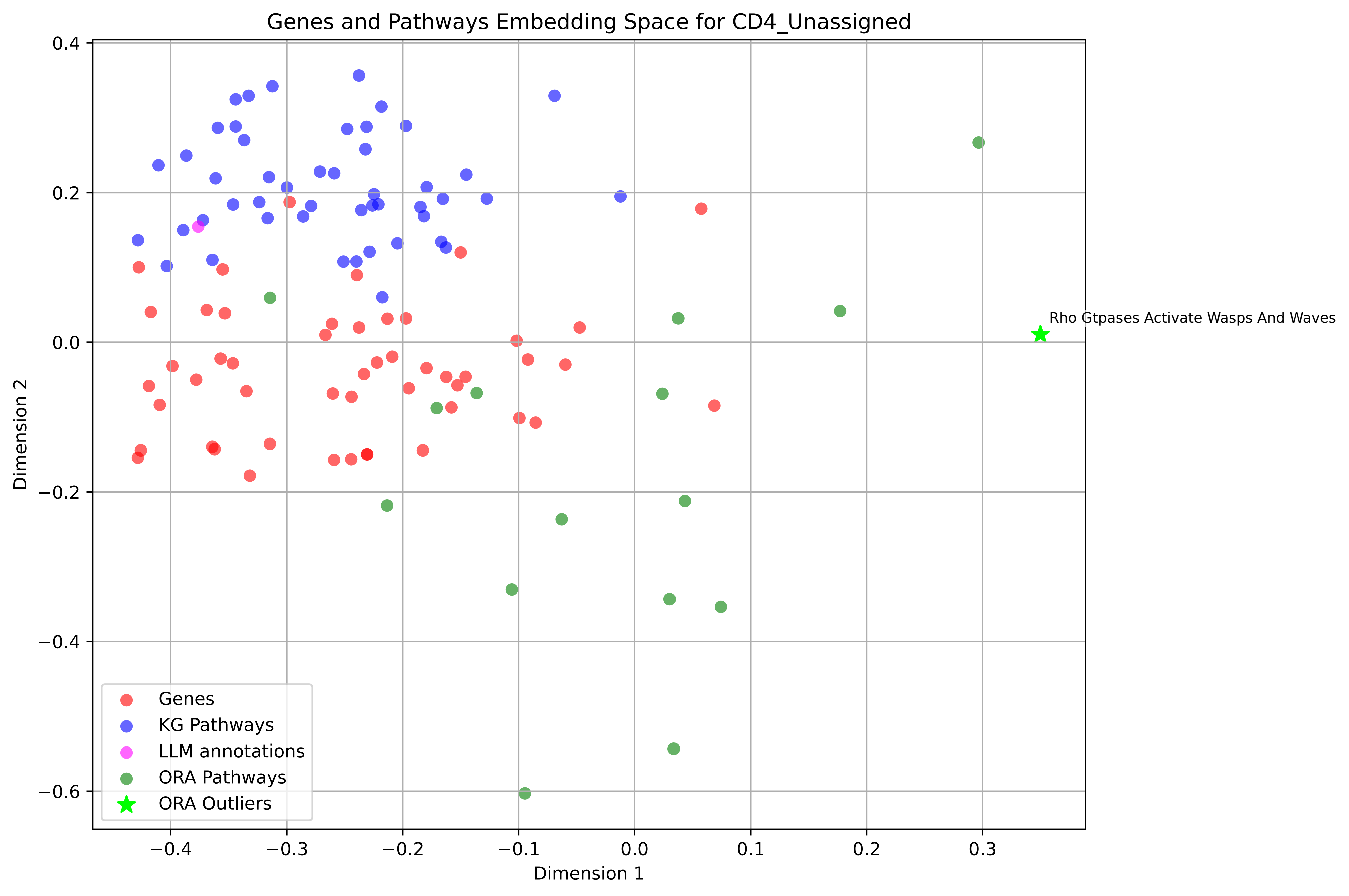

Genes and Pathways Embedding Space for CD4_Unassigned
Rho Gtpases Activate Wasps And Waves
Genes
KG Pathways
LLM annotations
ORA Pathways
ORA Outliers
Dimension 1
Dimension 2
0.4
0.2
0.0
−0.2
−0.4
−0.6
−0.4
−0.3
−0.2
−0.1
0.0
0.1
0.2
0.3

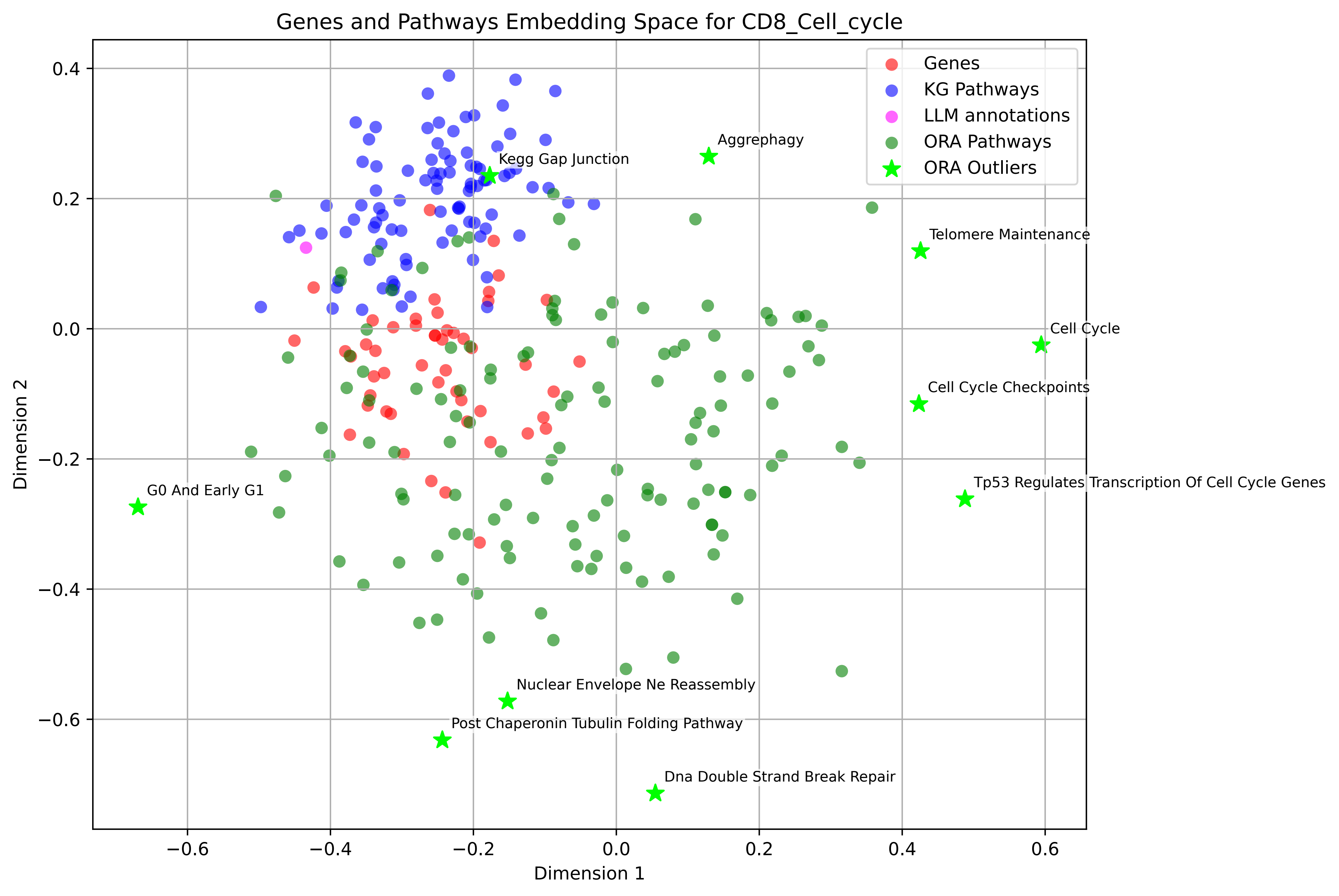

Genes and Pathways Embedding Space for CD8_Cell_cycle
Genes
KG Pathways
LLM annotations
ORA Pathways
ORA Outliers
Aggrephagy
Kegg Gap Junction
Telomere Maintenance
Cell Cycle
Cell Cycle Checkpoints
Tp53 Regulates Transcription Of Cell Cycle Genes
G0 And Early G1
Nuclear Envelope Ne Reassembly
Post Chaperonin Tubulin Folding Pathway
Dna Double Strand Break Repair
Dimension 1
Dimension 2
0.4
0.2
0.0
−0.2
−0.4
−0.6
−0.6
−0.4
−0.2
0.0
0.2
0.4
0.6

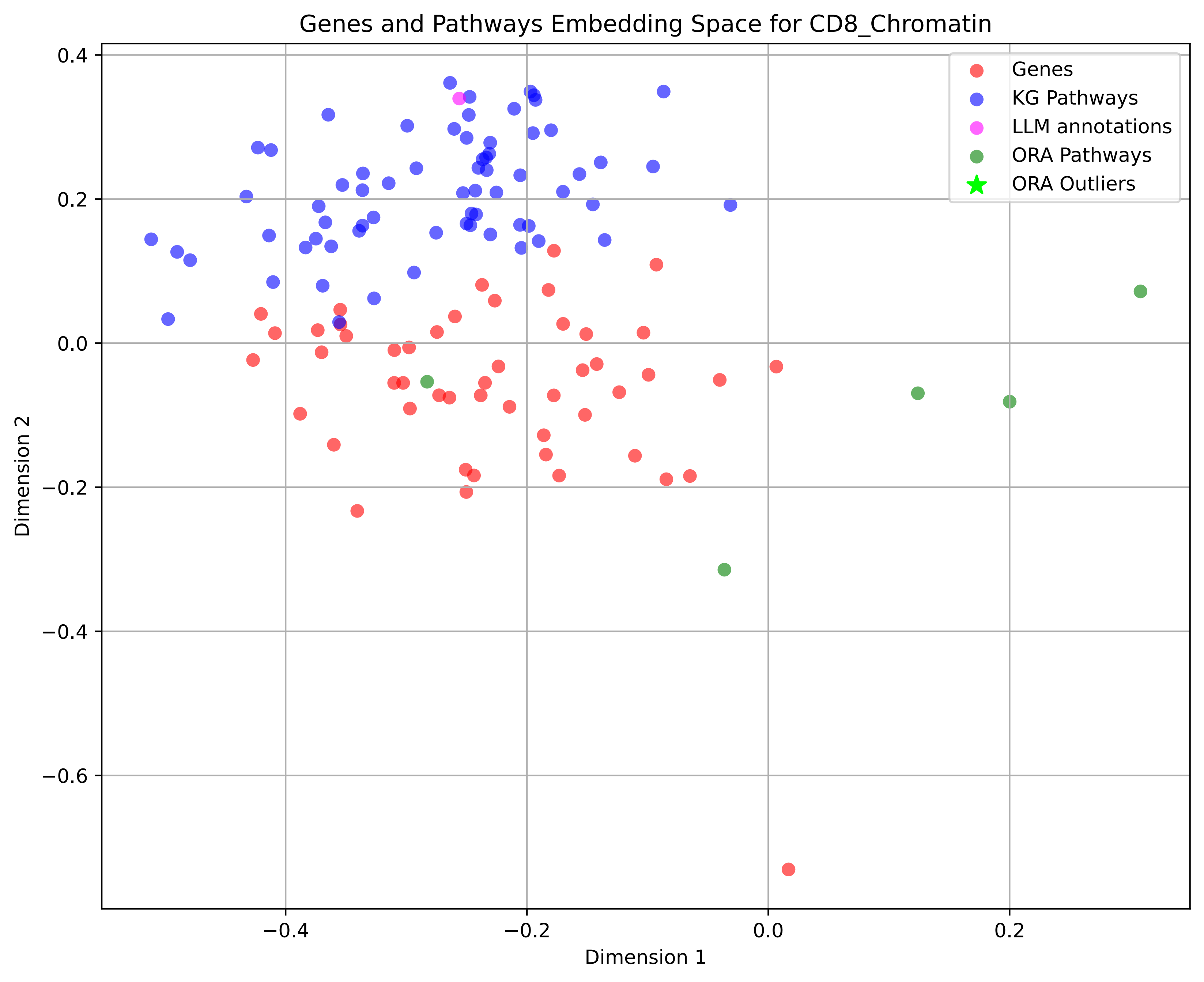

Genes and Pathways Embedding Space for CD8_Chromatin
Genes
KG Pathways
LLM annotations
ORA Pathways
ORA Outliers
Dimension 1
Dimension 2
0.4
0.2
0.0
−0.2
−0.4
−0.6
−0.4
−0.2
0.0
0.2

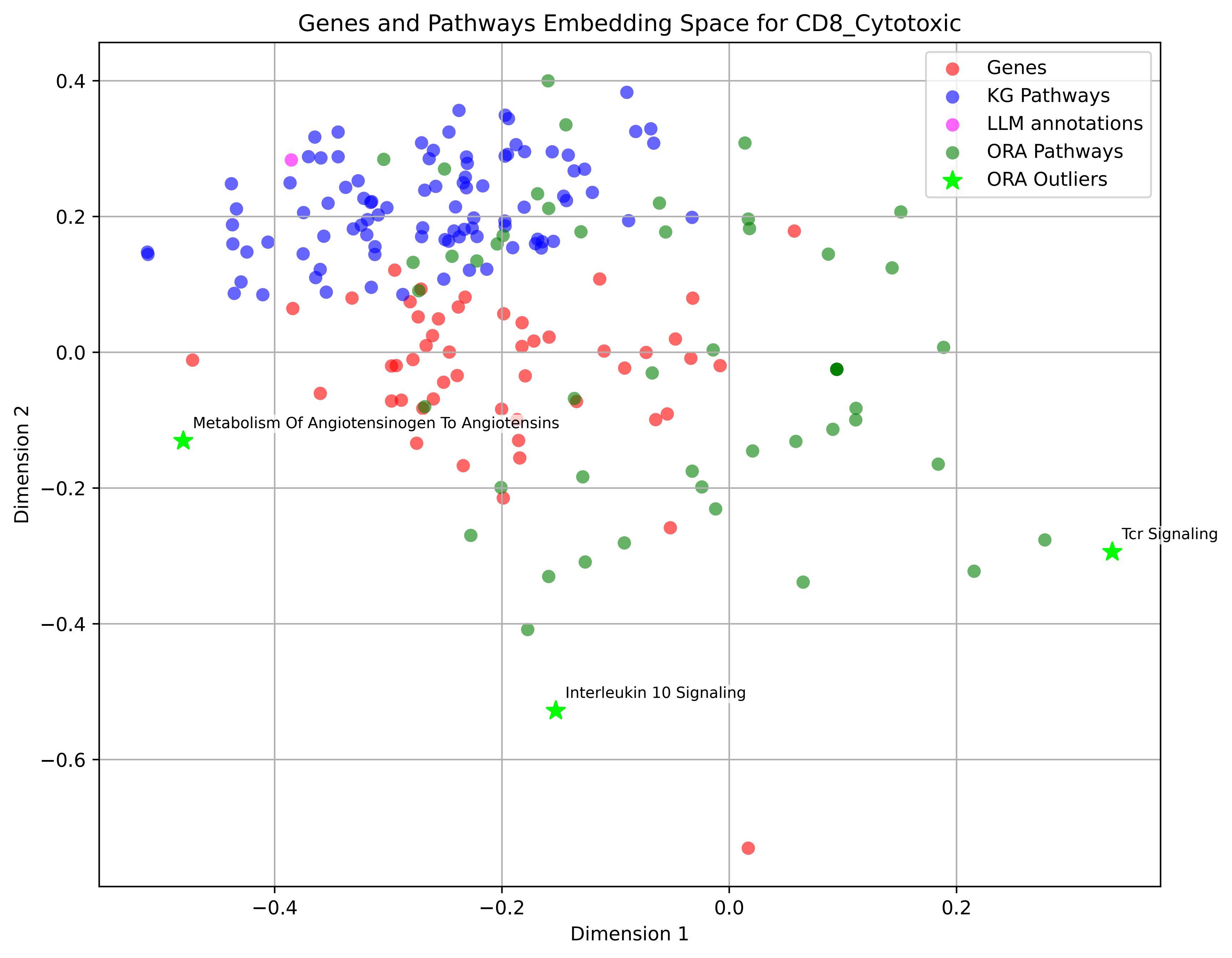

Genes and Pathways Embedding Space for CD8_Cytotoxic
Genes
KG Pathways
LLM annotations
ORA Pathways
ORA Outliers
Metabolism Of Angiotensinogen To Angiotensins
Tcr Signaling
Interleukin 10 Signaling
Dimension 1
Dimension 2
0.4
0.2
0.0
−0.2
−0.4
−0.6
−0.4
−0.2
0.0
0.2

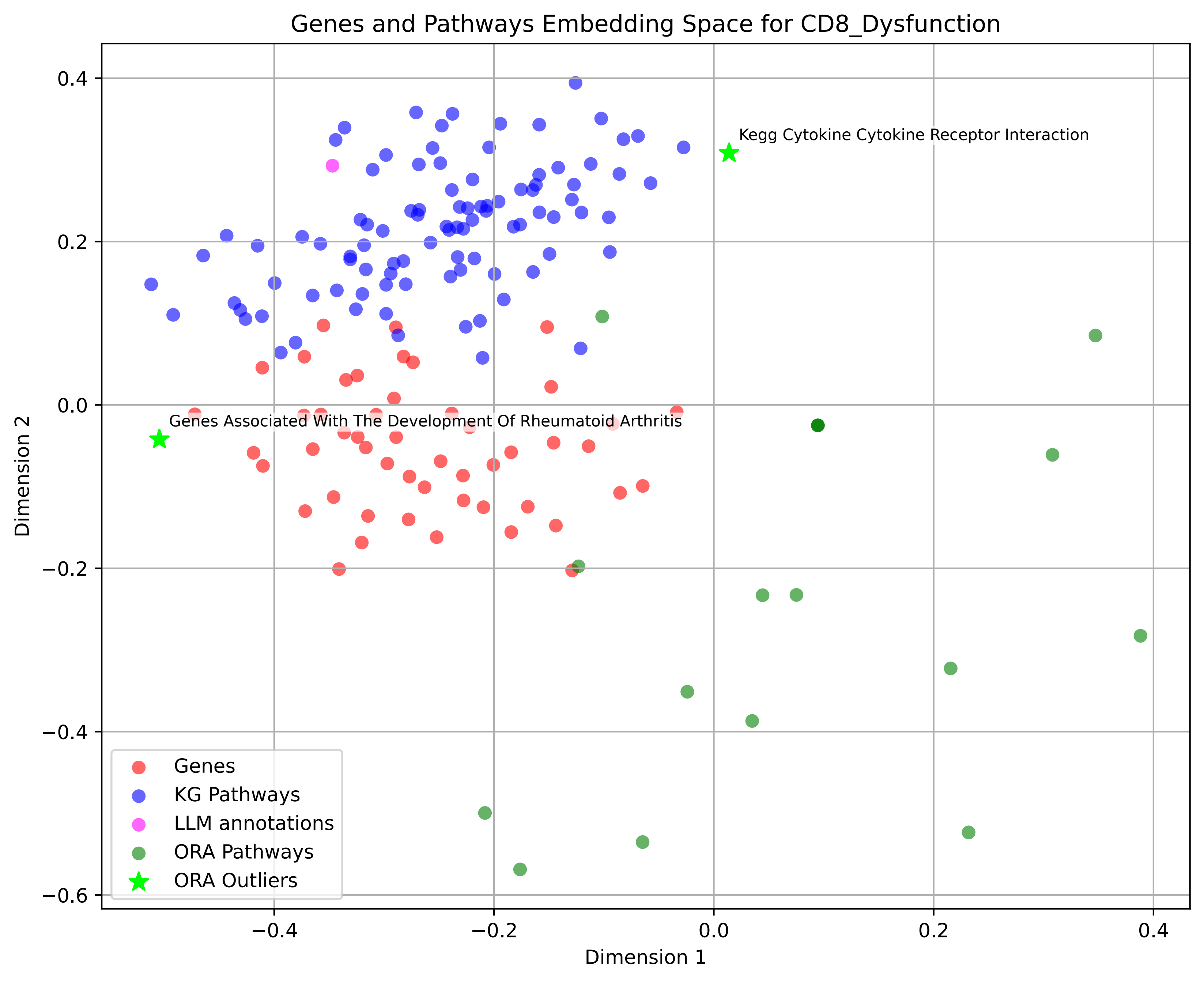

Genes and Pathways Embedding Space for CD8_Dysfunction
Kegg Cytokine Cytokine Receptor Interaction
Genes Associated With The Development Of Rheumatoid Arthritis
Genes
KG Pathways
LLM annotations
ORA Pathways
ORA Outliers
Dimension 1
Dimension 2
0.4
0.2
0.0
−0.2
−0.4
−0.6
−0.4
−0.2
0.0
0.2
0.4

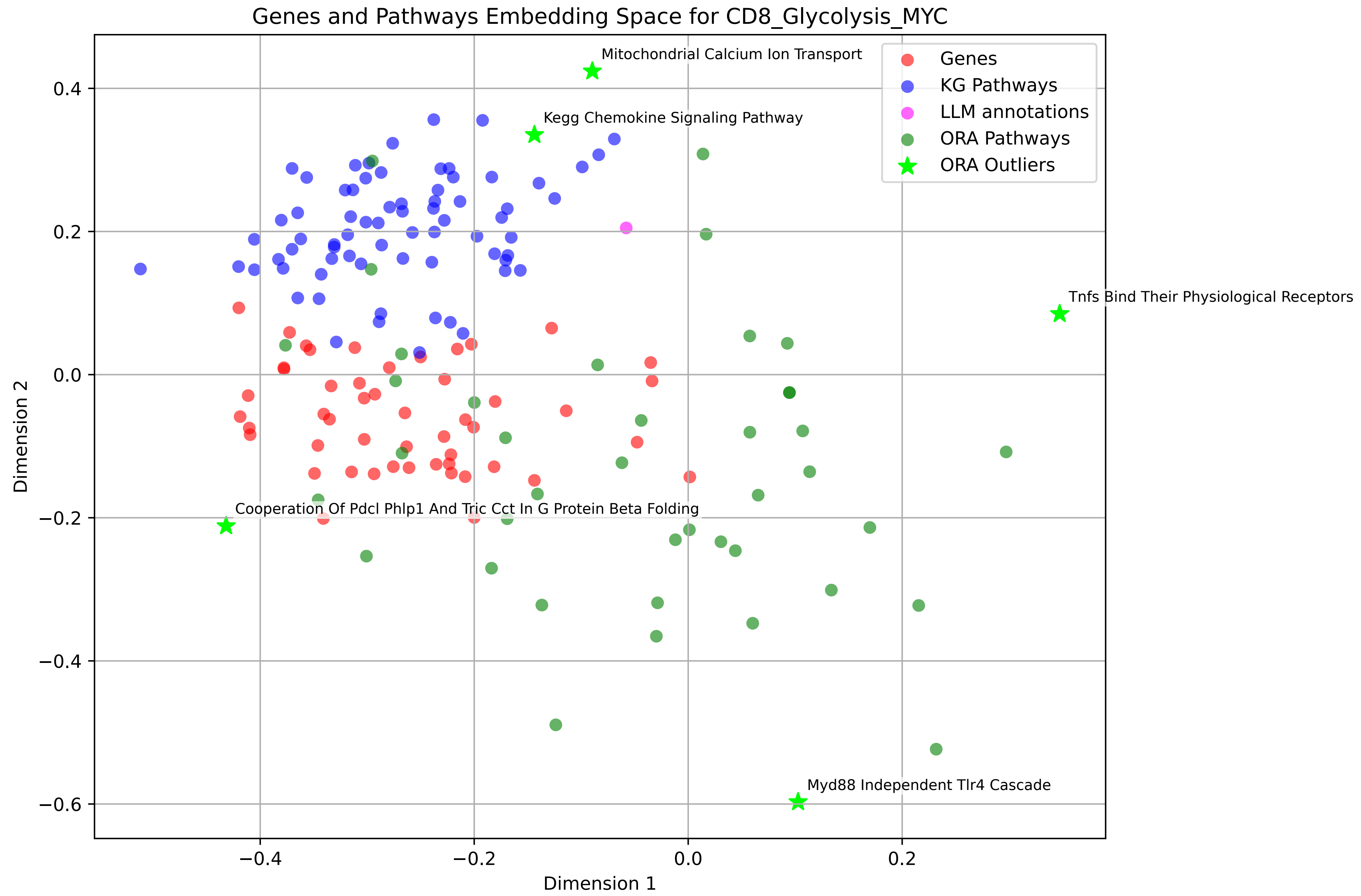
Genes and Pathways Embedding Space for CD8_Glycolysis_MYC
Mitochondrial Calcium Ion Transport
Kegg Chemokine Signaling Pathway
Tnfs Bind Their Physiological Receptors
Cooperation Of Pdcl Phlp1 And Tric Cct In G Protein Beta Folding
Myd88 Independent Tlr4 Cascade
Genes
KG Pathways
LLM annotations
ORA Pathways
ORA Outliers
Dimension 1
Dimension 2
0.4
0.2
0.0
−0.2
−0.4
−0.6
−0.4
−0.2
0.0
0.2

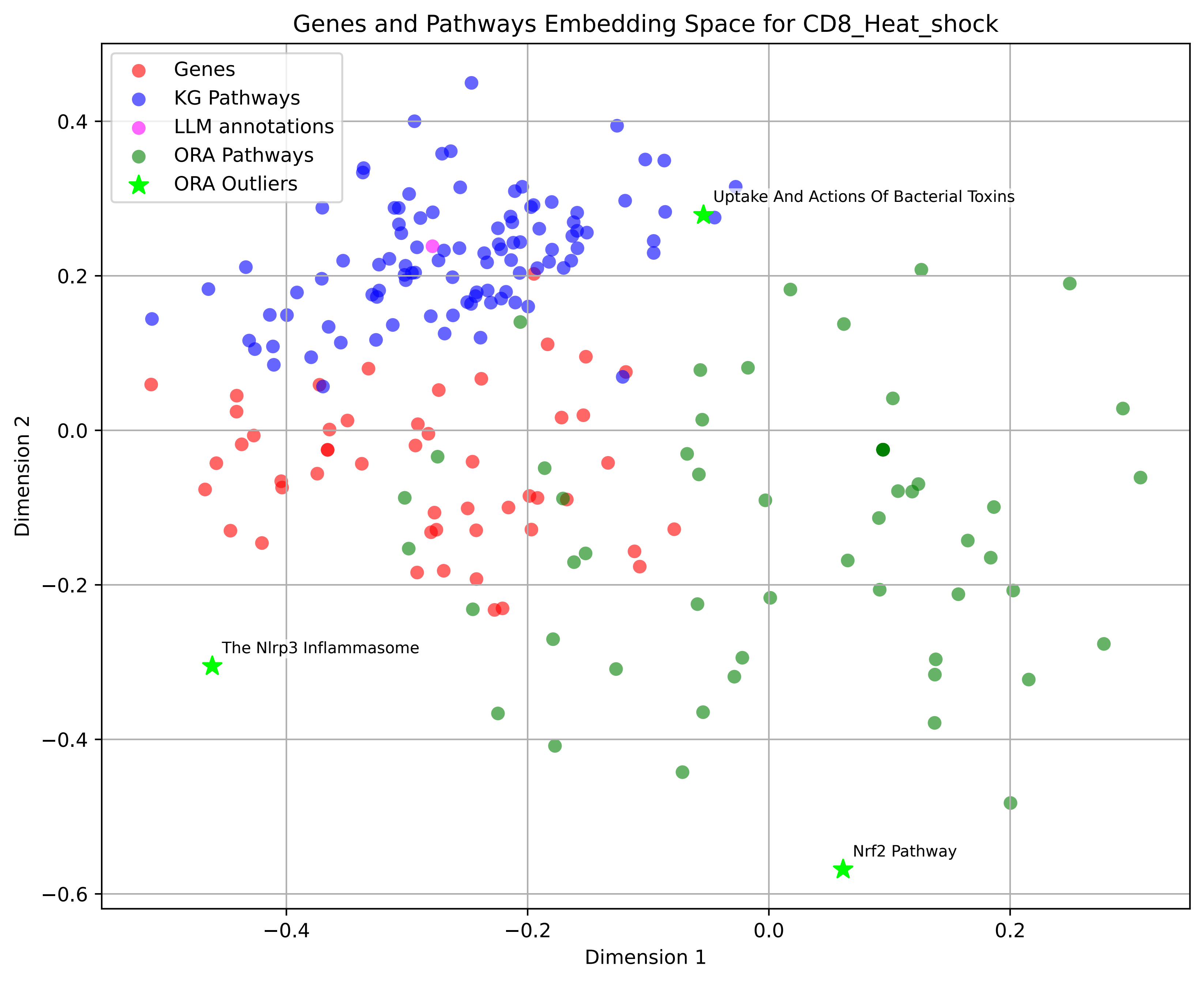

Genes and Pathways Embedding Space for CD8_Heat_shock
Genes
KG Pathways
LLM annotations
ORA Pathways
ORA Outliers
Uptake And Actions Of Bacterial Toxins
The Nlrp3 Inflammasome
Nrf2 Pathway
Dimension 1
Dimension 2
0.4
0.2
0.0
−0.2
−0.4
−0.6
−0.4
−0.2
0.0
0.2

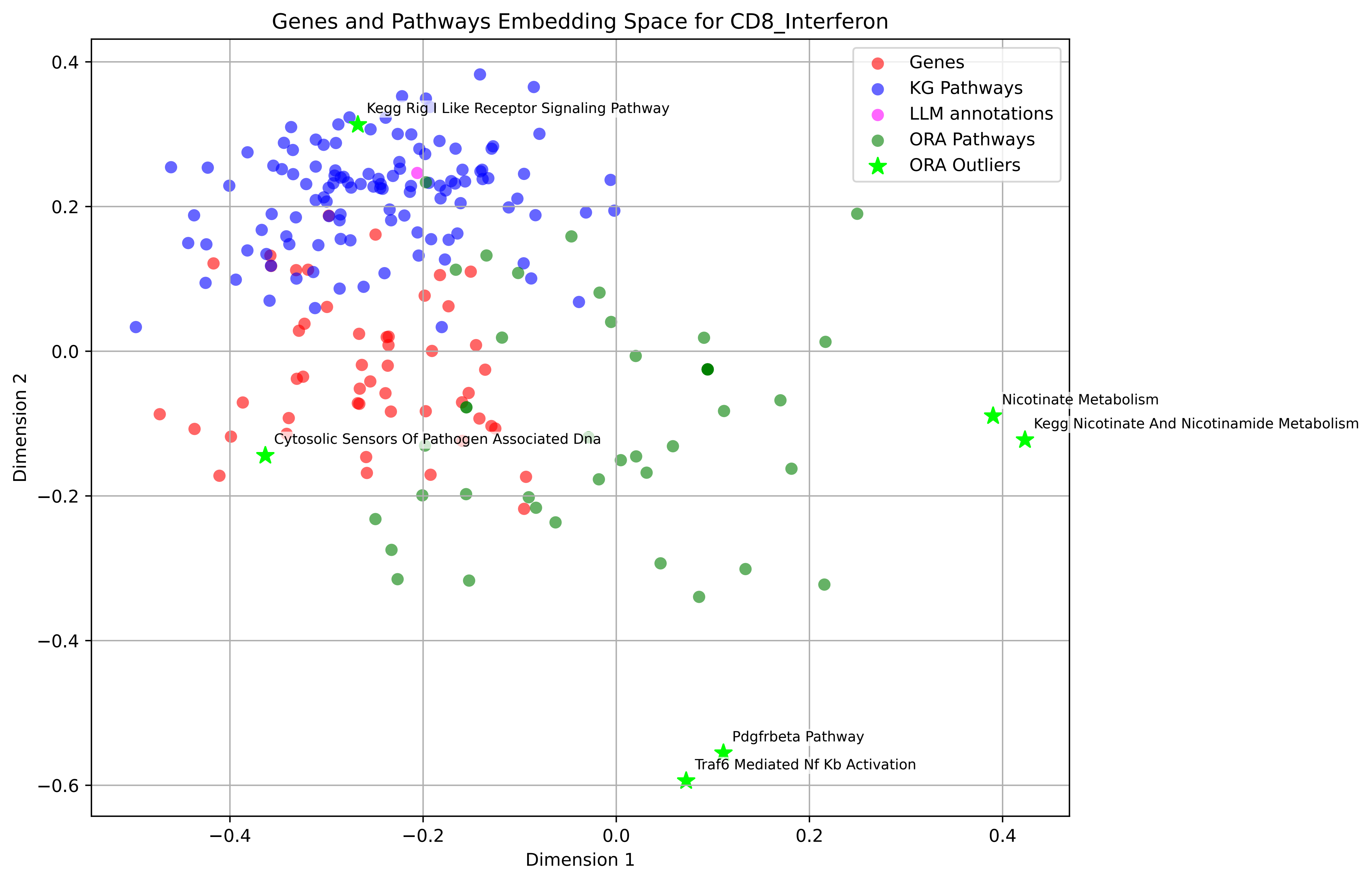
Genes and Pathways Embedding Space for CD8_Interferon
Genes
KG Pathways
LLM annotations
ORA Pathways
ORA Outliers
Kegg Rig I Like Receptor Signaling Pathway
Nicotinate Metabolism
Kegg Nicotinate And Nicotinamide Metabolism
Cytosolic Sensors Of Pathogen Associated Dna
Pdgfrbeta Pathway
Traf6 Mediated Nf Kb Activation
Dimension 1
Dimension 2
0.4
0.2
0.0
−0.2
−0.4
−0.6
−0.4
−0.2
0.0
0.2
0.4

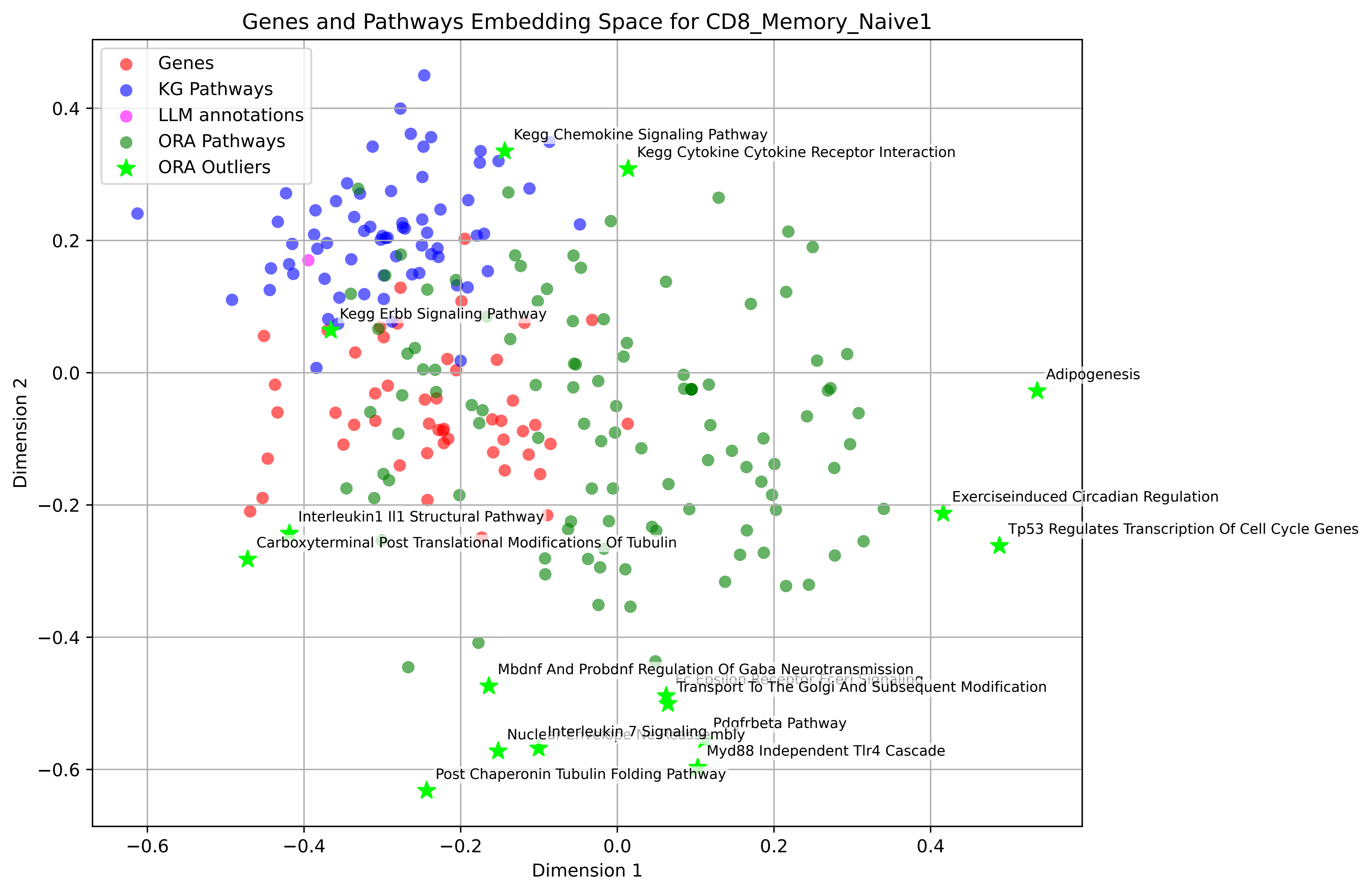
Genes and Pathways Embedding Space for CD8_Memory_Naive1
Genes
KG Pathways
LLM annotations
ORA Pathways
ORA Outliers
Kegg Chemokine Signaling Pathway
Kegg Cytokine Cytokine Receptor Interaction
Kegg Erbb Signaling Pathway
Adipogenesis
Exerciseinduced Circadian Regulation
Tp53 Regulates Transcription Of Cell Cycle Genes
Interleukin1 Il1 Structural Pathway
Carboxyterminal Post Translational Modifications Of Tubulin
Mbdnf And Probdnf Regulation Of Gaba Neurotransmission
Fc Epsilon Receptor Fceri Signaling
Transport To The Golgi And Subsequent Modification
Pdgfrbeta Pathway
Interleukin 7 Signaling
Nuclear Envelope Ne Reassembly
Myd88 Independent Tlr4 Cascade
Post Chaperonin Tubulin Folding Pathway
Dimension 1
Dimension 2
0.4
0.2
0.0
−0.2
−0.4
−0.6
−0.6
−0.4
−0.2
0.0
0.2
0.4

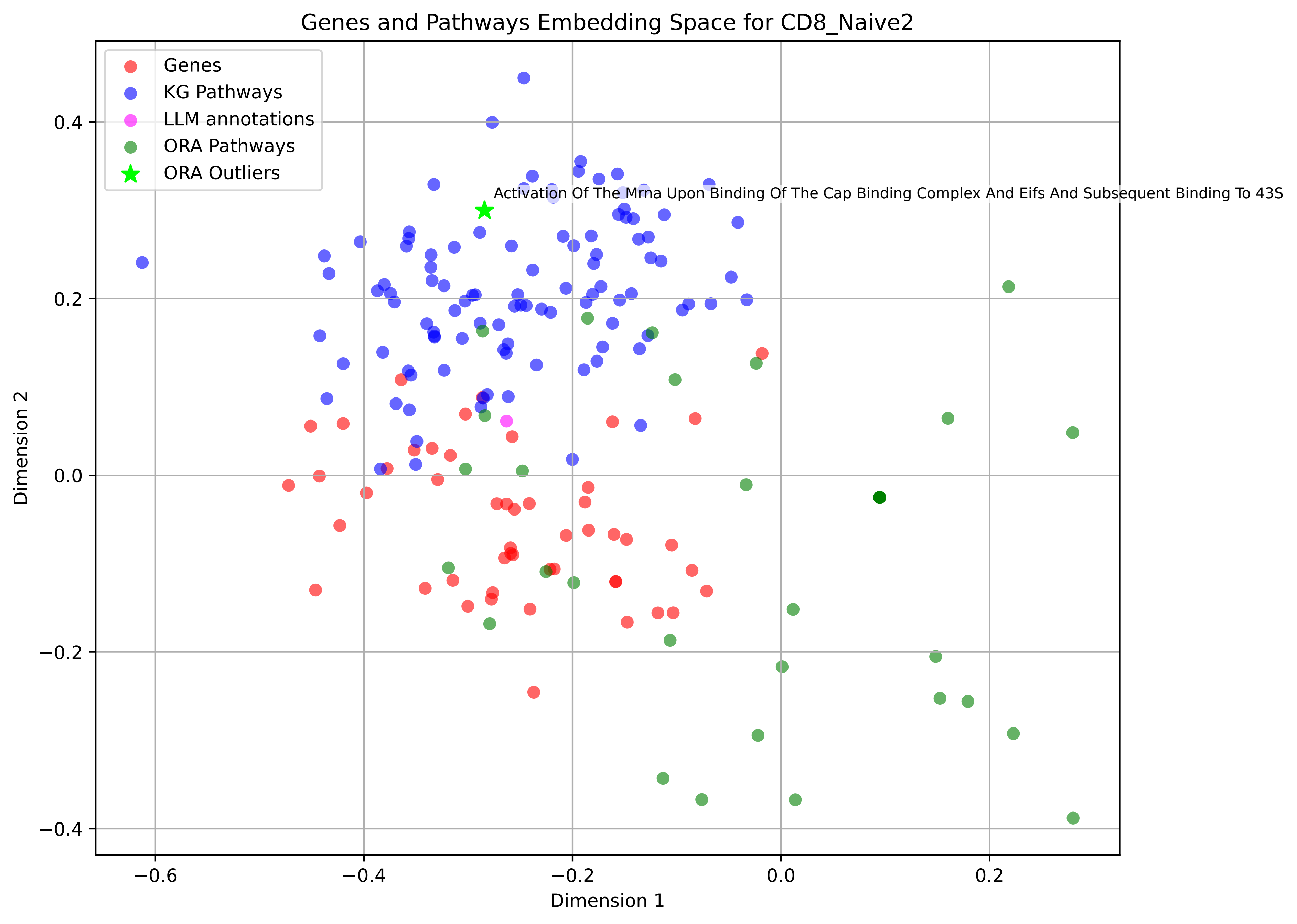

Genes and Pathways Embedding Space for CD8_Naive2
Genes
KG Pathways
LLM annotations
ORA Pathways
ORA Outliers
Activation Of The Mrna Upon Binding Of The Cap Binding Complex And Eifs And Subsequent Binding To 43S
Dimension 1
Dimension 2
−0.6
−0.4
−0.2
0.0
0.2
0.4

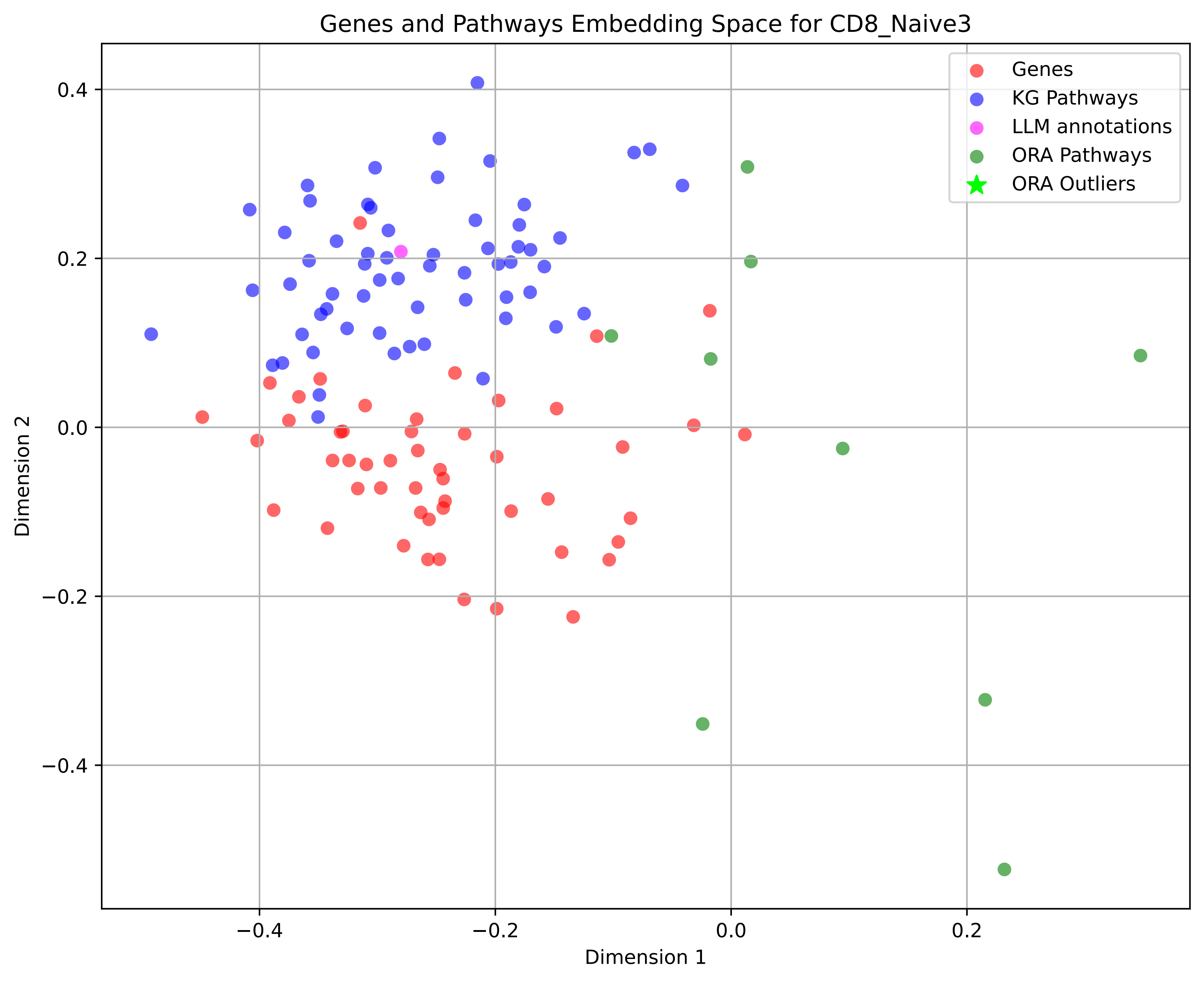

Genes and Pathways Embedding Space for CD8_Naive3
Genes
KG Pathways
LLM annotations
ORA Pathways
ORA Outliers
Dimension 1
Dimension 2
0.4
0.2
0.0
−0.2
−0.4
−0.4
−0.2
0.0
0.2

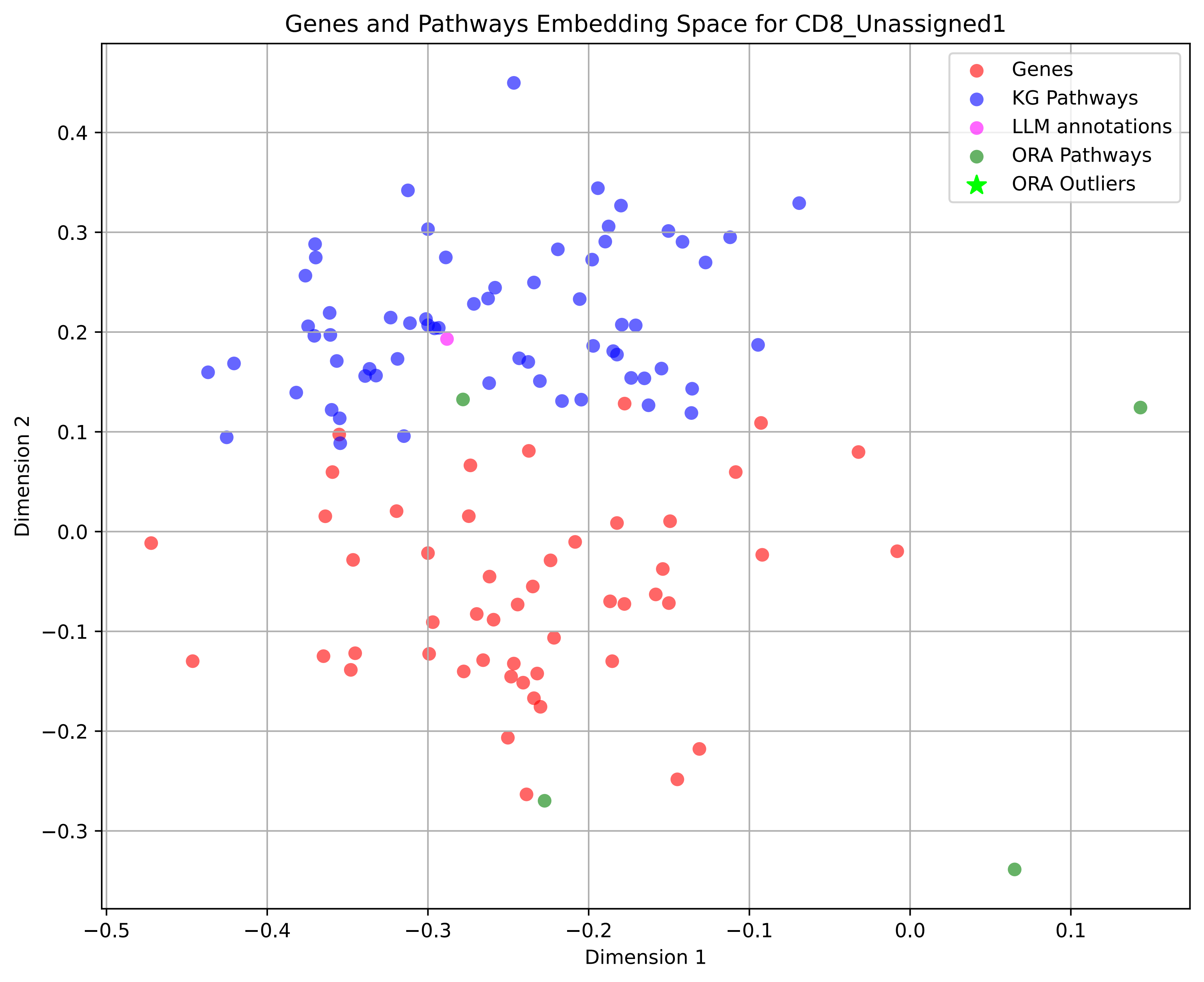

Genes and Pathways Embedding Space for CD8_Unassigned1
Genes
KG Pathways
LLM annotations
ORA Pathways
ORA Outliers
Dimension 1
Dimension 2
−0.5
−0.4
−0.3
−0.2
−0.1
0.0
0.1
0.4
0.3
0.2
0.1
0.0
−0.1
−0.2
−0.3

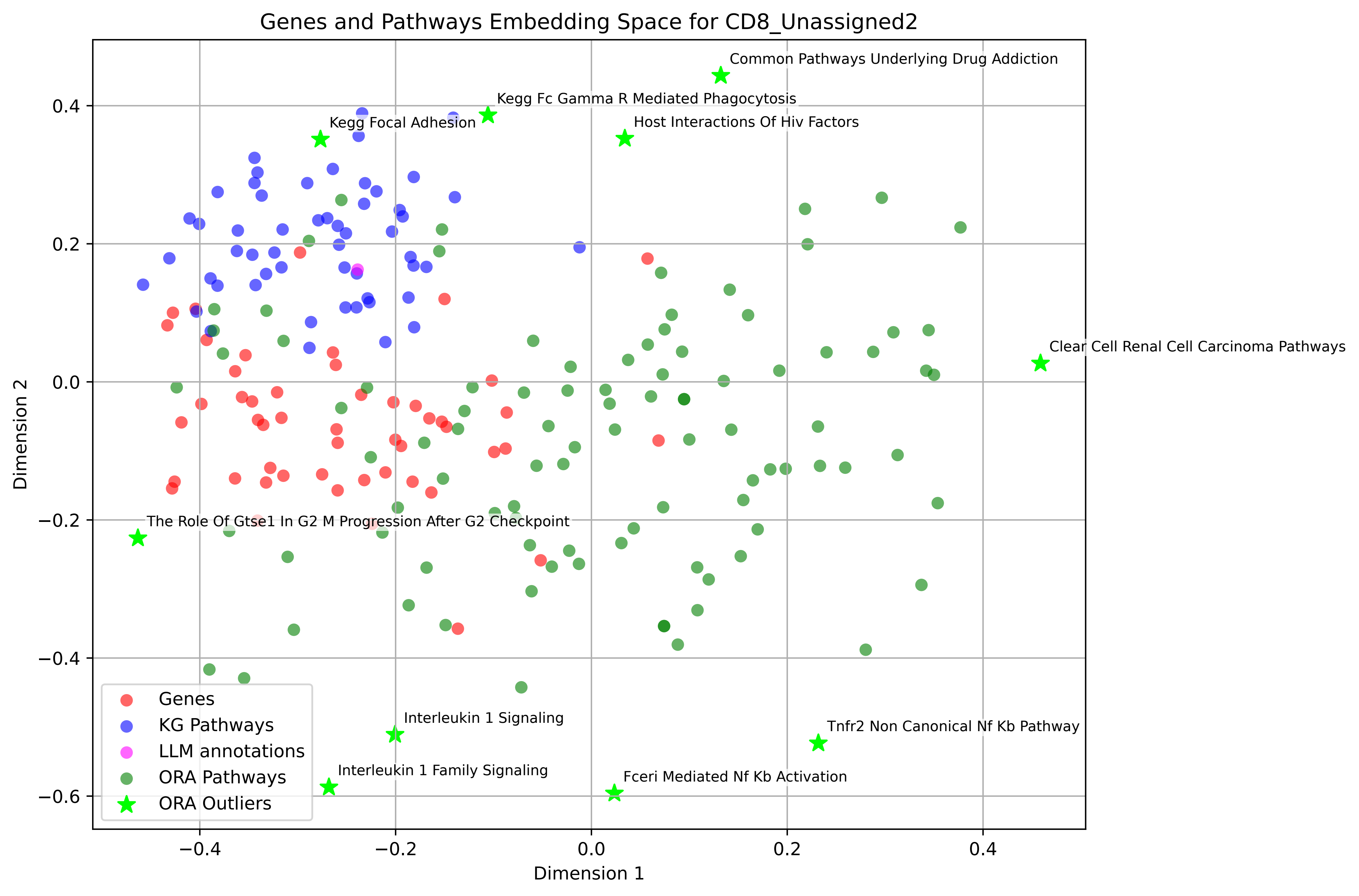

Genes and Pathways Embedding Space for CD8_Unassigned2
Common Pathways Underlying Drug Addiction
Kegg Fc Gamma R Mediated Phagocytosis
Kegg Focal Adhesion
Host Interactions Of Hiv Factors
Clear Cell Renal Cell Carcinoma Pathways
The Role Of Gtse1 In G2 M Progression After G2 Checkpoint
Interleukin 1 Signaling
Tnfr2 Non Canonical Nf Kb Pathway
Interleukin 1 Family Signaling
Fceri Mediated Nf Kb Activation
Genes
KG Pathways
LLM annotations
ORA Pathways
ORA Outliers
Dimension 1
Dimension 2
0.4
0.2
0.0
−0.2
−0.4
−0.6
−0.4
−0.2
0.0
0.2
0.4

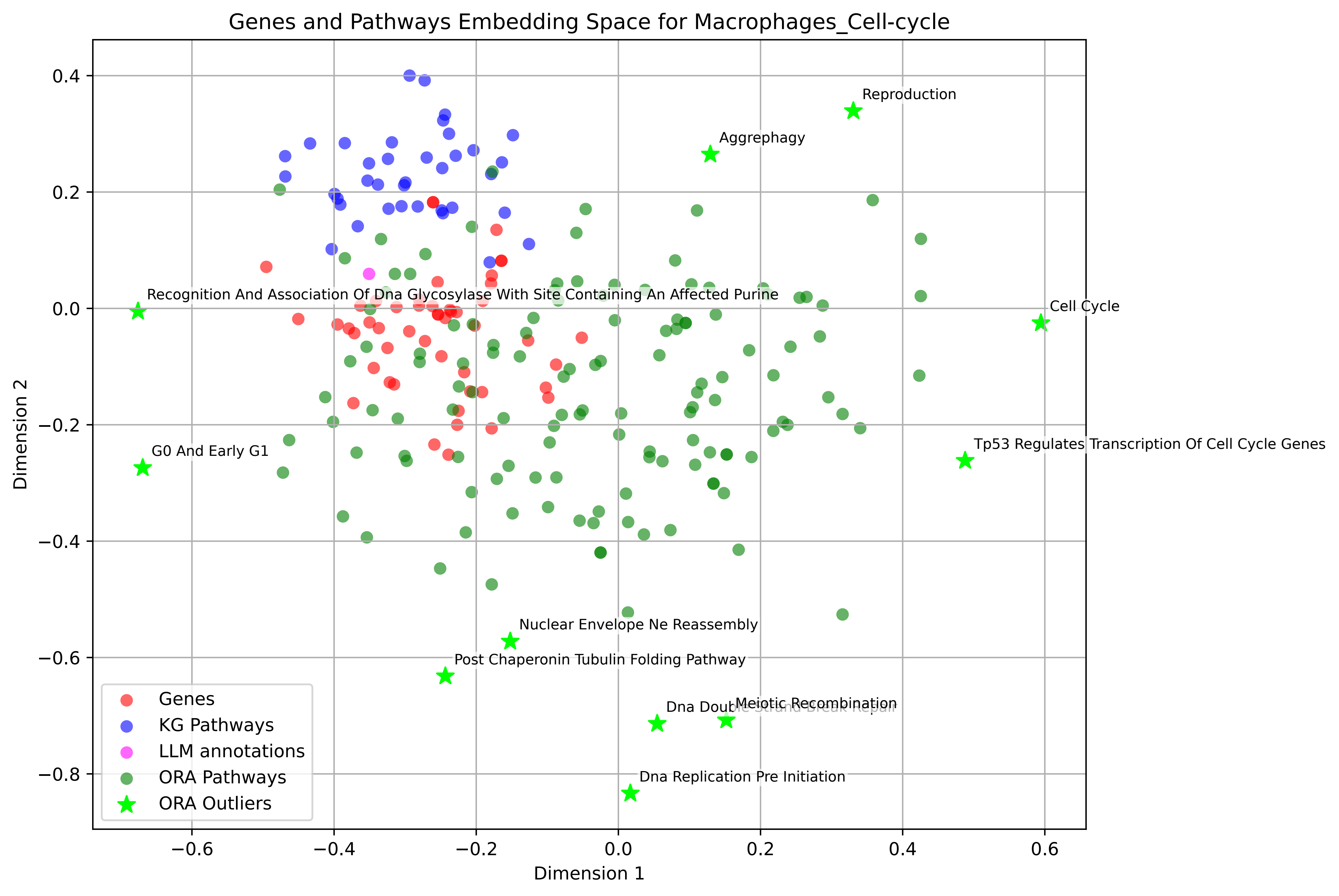

Genes and Pathways Embedding Space for Macrophages_Cell-cycle
Reproduction
Aggrephagy
Recognition And Association Of Dna Glycosylase With Site Containing An Affected Purine
Cell Cycle
G0 And Early G1
Tp53 Regulates Transcription Of Cell Cycle Genes
Nuclear Envelope Ne Reassembly
Post Chaperonin Tubulin Folding Pathway
Dna Double Strand Break Repair
Meiotic Recombination
Dna Replication Pre Initiation
Genes
KG Pathways
LLM annotations
ORA Pathways
ORA Outliers
Dimension 1
Dimension 2

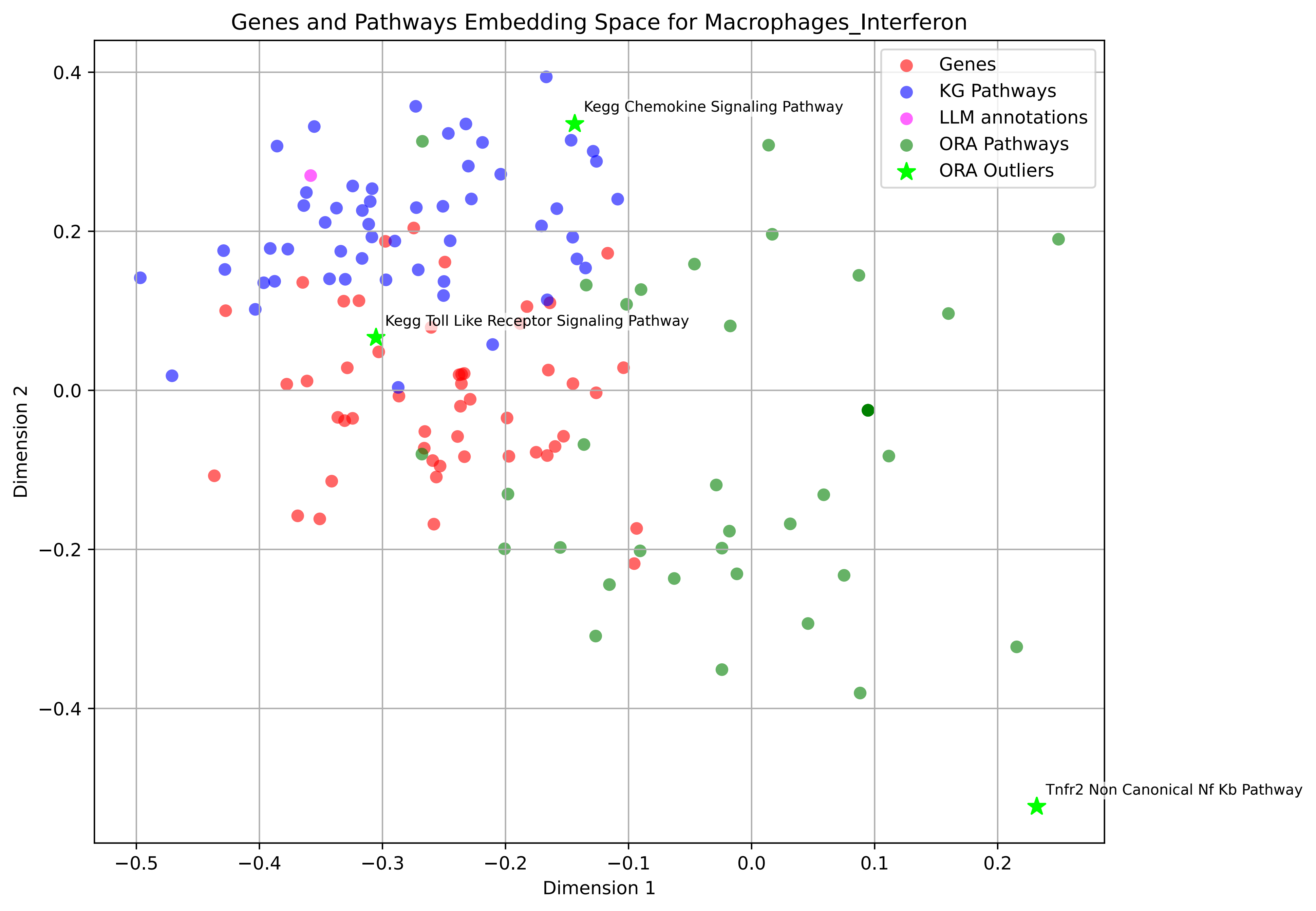

Genes and Pathways Embedding Space for Macrophages_Interferon
Genes
KG Pathways
LLM annotations
ORA Pathways
ORA Outliers
Kegg Chemokine Signaling Pathway
Kegg Toll Like Receptor Signaling Pathway
Tnfr2 Non Canonical Nf Kb Pathway
Dimension 1
Dimension 2
−0.5
−0.4
−0.3
−0.2
−0.1
0.0
0.1
0.2
0.4
0.2
0.0
−0.2
−0.4

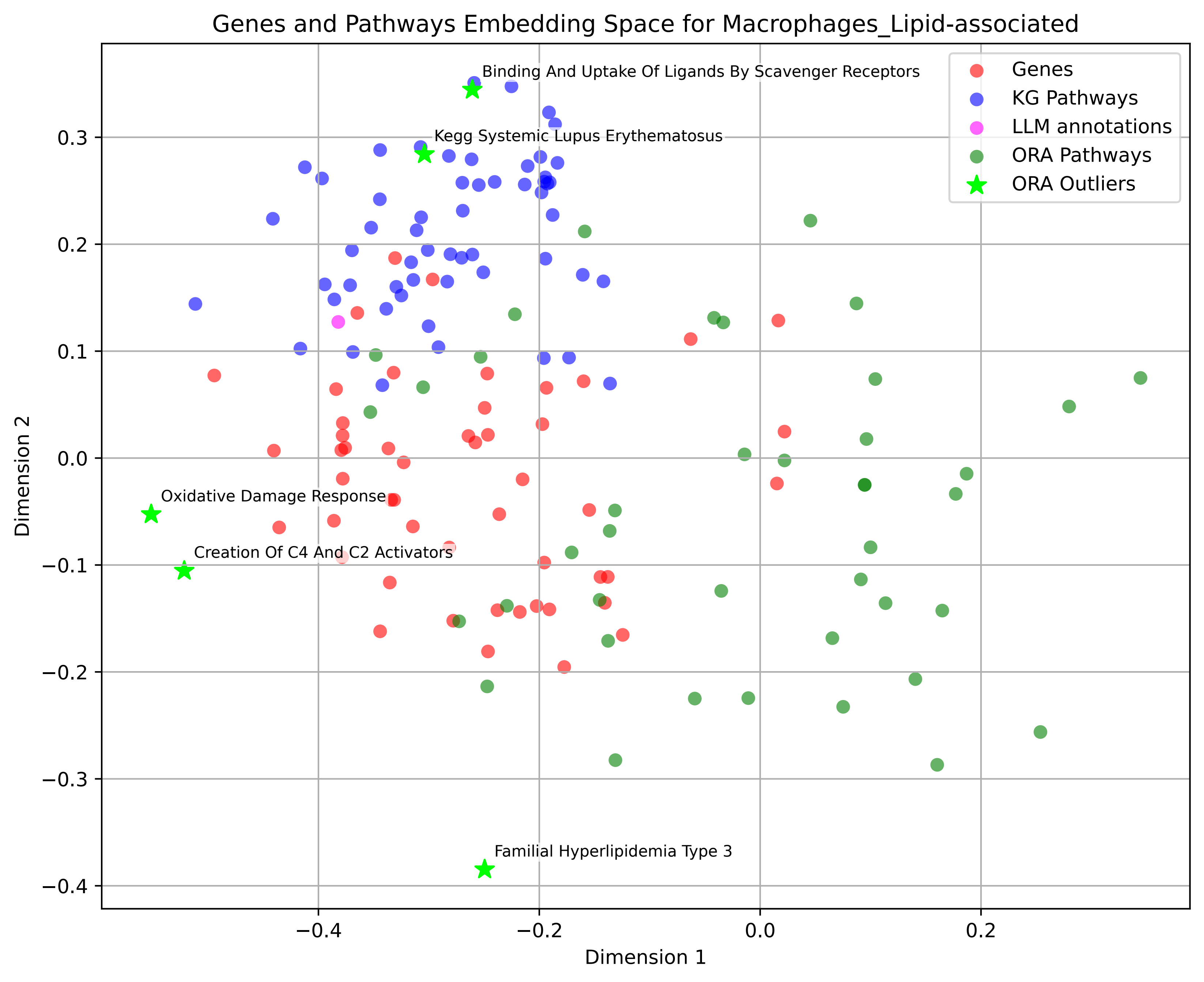

Genes and Pathways Embedding Space for Macrophages_Lipid-associated
Genes
KG Pathways
LLM annotations
ORA Pathways
ORA Outliers
Binding And Uptake Of Ligands By Scavenger Receptors
Kegg Systemic Lupus Erythematosus
Oxidative Damage Response
Creation Of C4 And C2 Activators
Familial Hyperlipidemia Type 3
Dimension 1
Dimension 2
−0.4
−0.2
0.0
0.2
0.3
0.2
0.1
0.0
−0.1
−0.2
−0.3
−0.4

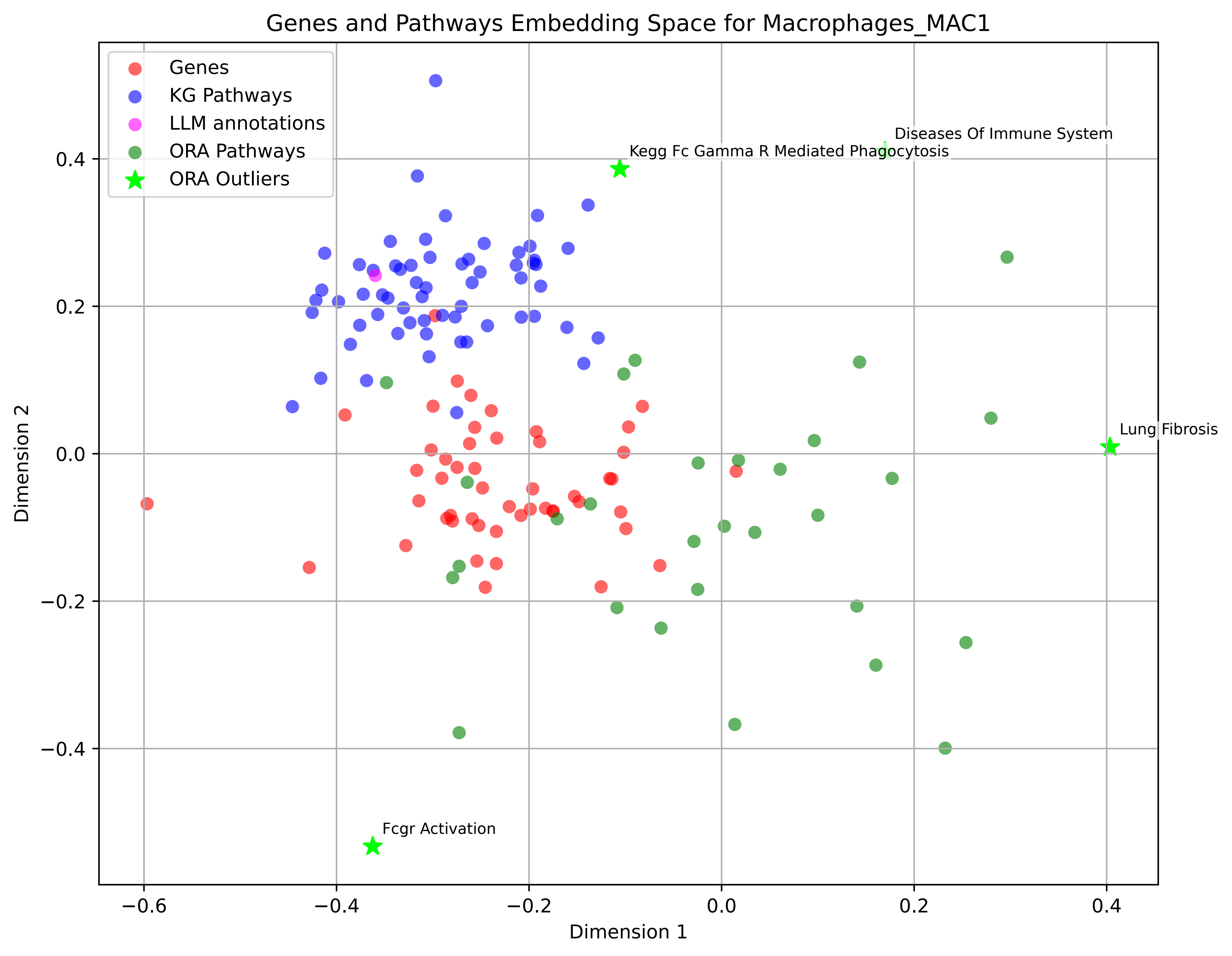
Genes and Pathways Embedding Space for Macrophages_MAC1
Genes
KG Pathways
LLM annotations
ORA Pathways
ORA Outliers
Diseases Of Immune System
Kegg Fc Gamma R Mediated Phagocytosis
Lung Fibrosis
Fcgr Activation
Dimension 1
Dimension 2
0.4
0.2
0.0
−0.2
−0.4
−0.6
0.0
0.2
0.4

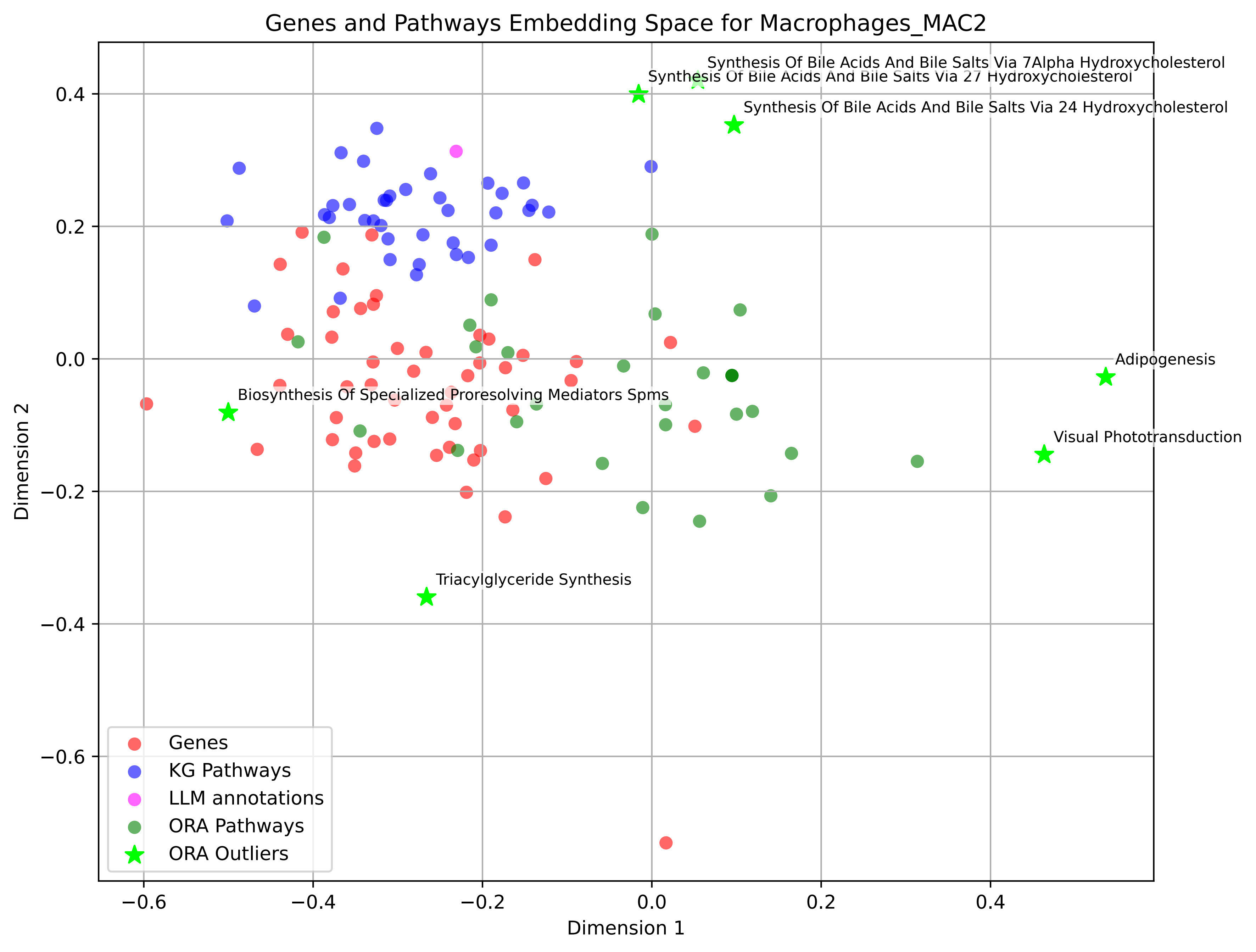
Genes and Pathways Embedding Space for Macrophages_MAC2
Synthesis Of Bile Acids And Bile Salts Via 7Alpha Hydroxycholesterol
Synthesis Of Bile Acids And Bile Salts Via 27 Hydroxycholesterol
Synthesis Of Bile Acids And Bile Salts Via 24 Hydroxycholesterol
Adipogenesis
Biosynthesis Of Specialized Proresolving Mediators Spms
Visual Phototransduction
Triacylglyceride Synthesis
0.4
0.2
0.0
−0.2
−0.4
−0.6
−0.6
−0.4
−0.2
0.0
0.2
0.4
Dimension 1
Dimension 2
Genes
KG Pathways
LLM annotations
ORA Pathways
ORA Outliers

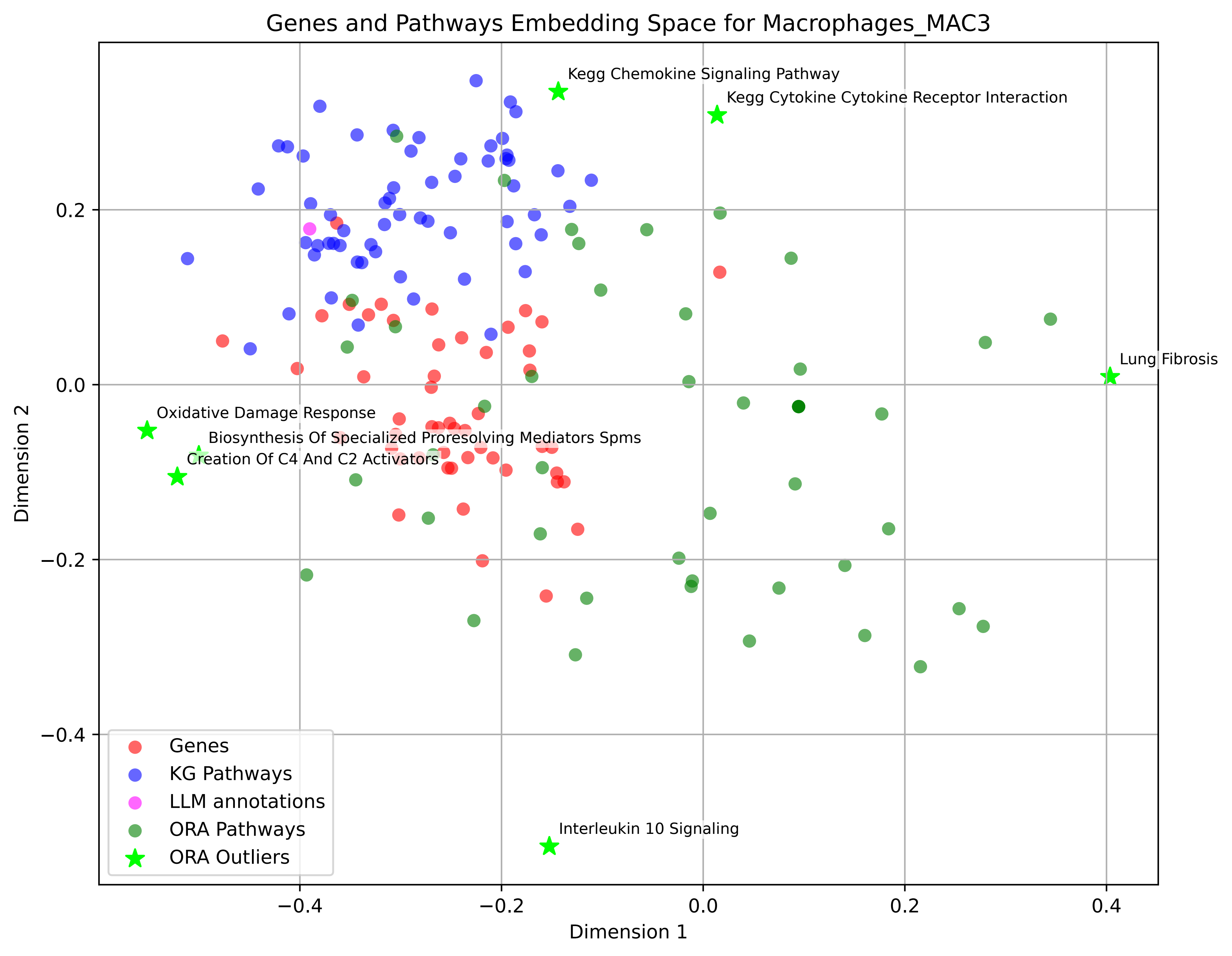

Genes and Pathways Embedding Space for Macrophages_MAC3
Kegg Chemokine Signaling Pathway
Kegg Cytokine Cytokine Receptor Interaction
Lung Fibrosis
Oxidative Damage Response
Biosynthesis Of Specialized Proresolving Mediators Spms
Creation Of C4 And C2 Activators
Interleukin 10 Signaling
Genes
KG Pathways
LLM annotations
ORA Pathways
ORA Outliers
Dimension 1
Dimension 2
0.2
0.0
−0.2
−0.4
−0.4
−0.2
0.0
0.2
0.4

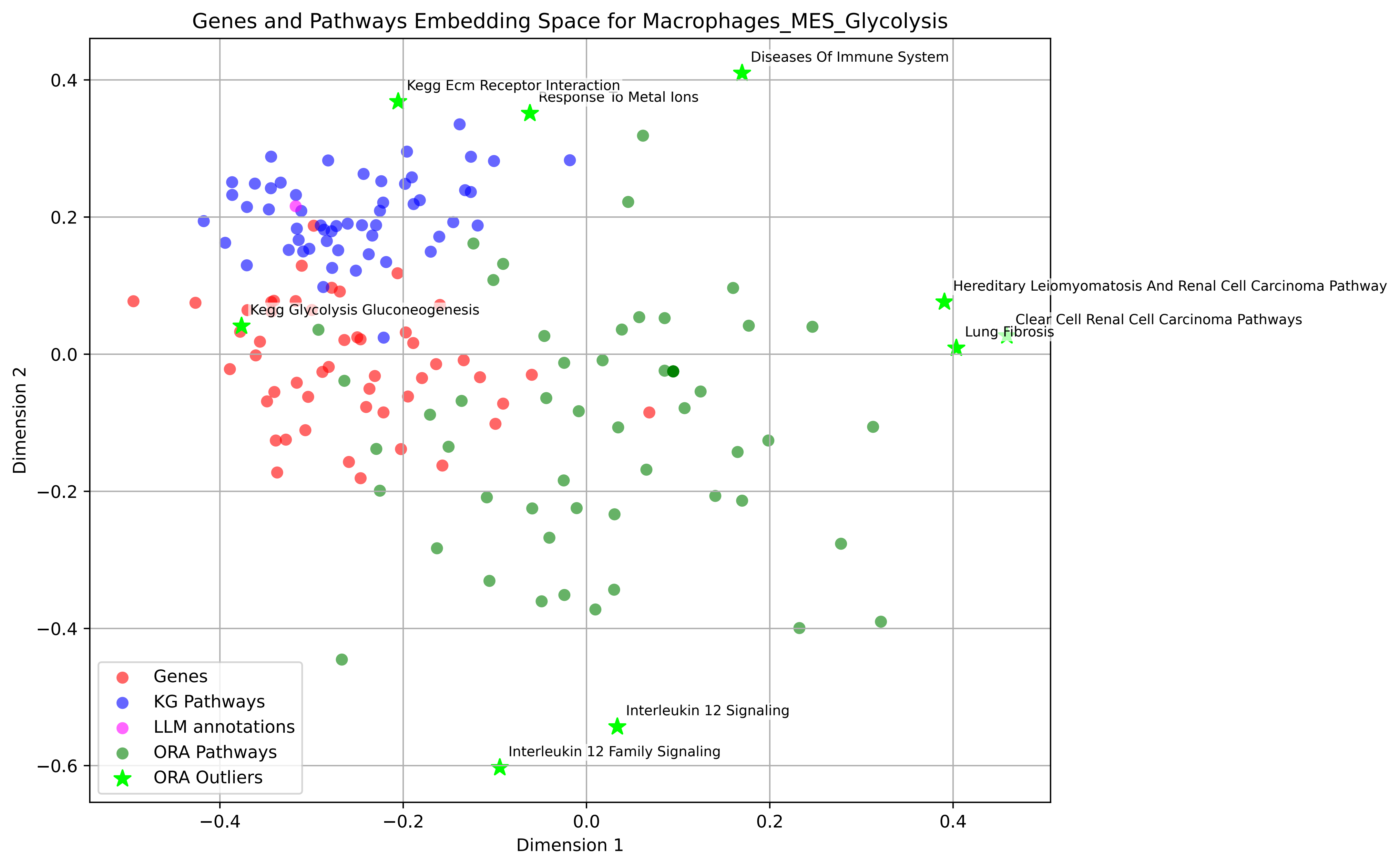

Genes and Pathways Embedding Space for Macrophages_MES_Glycolysis
Diseases Of Immune System
Kegg Ecm Receptor Interaction
Response To Metal Ions
Hereditary Leiomyomatosis And Renal Cell Carcinoma Pathway
Clear Cell Renal Cell Carcinoma Pathways
Lung Fibrosis
Kegg Glycolysis Gluconeogenesis
Interleukin 12 Signaling
Interleukin 12 Family Signaling
Genes
KG Pathways
LLM annotations
ORA Pathways
ORA Outliers
Dimension 1
Dimension 2
0.4
0.2
0.0
−0.2
−0.4
−0.6
−0.4
−0.2
0.0
0.2
0.4

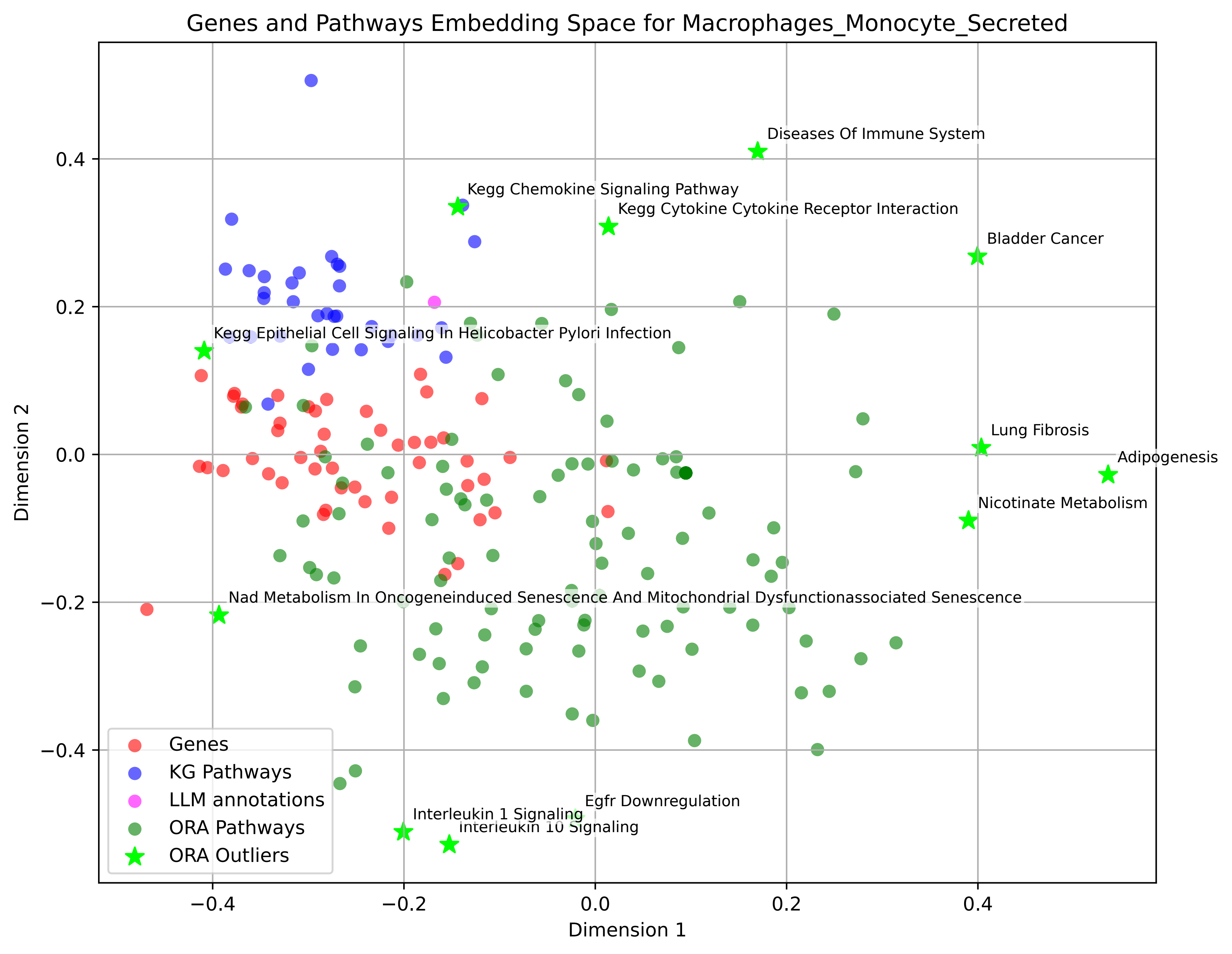

Genes and Pathways Embedding Space for Macrophages_Monocyte_Secreted
Diseases Of Immune System
Kegg Chemokine Signaling Pathway
Kegg Cytokine Cytokine Receptor Interaction
Bladder Cancer
Kegg Epithelial Cell Signaling In Helicobacter Pylori Infection
Lung Fibrosis
Adipogenesis
Nicotinate Metabolism
Nad Metabolism In Oncogeneinduced Senescence And Mitochondrial Dysfunctionassociated Senescence
Egfr Downregulation
Interleukin 1 Signaling
Interleukin 10 Signaling
Genes
KG Pathways
LLM annotations
ORA Pathways
ORA Outliers
Dimension 1
Dimension 2
−0.4
−0.2
0.0
0.2
0.4

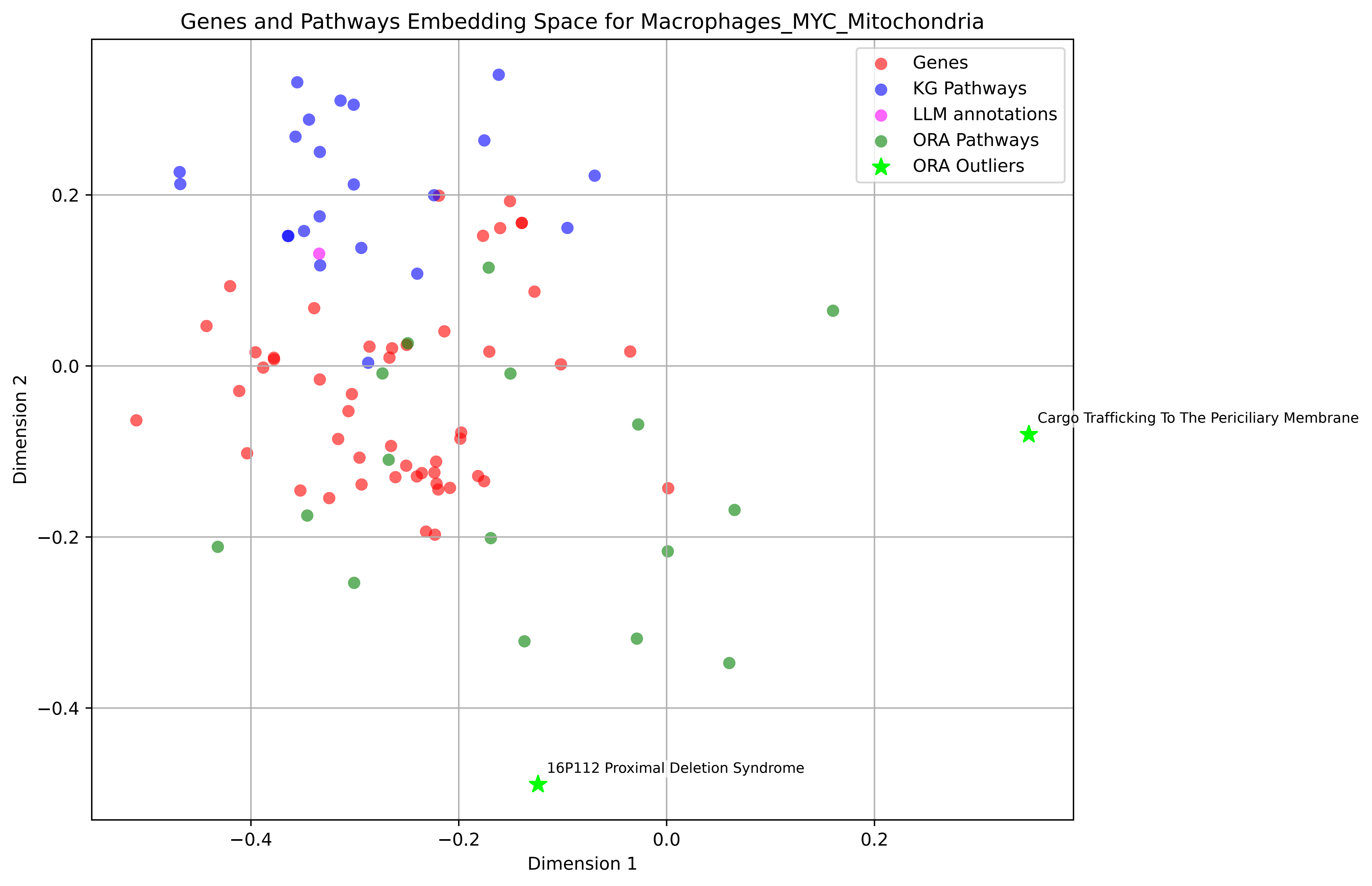

Genes and Pathways Embedding Space for Macrophages_MYC_Mitochondria
Genes
KG Pathways
LLM annotations
ORA Pathways
ORA Outliers
Cargo Trafficking To The Periciliary Membrane
16P112 Proximal Deletion Syndrome
Dimension 1
Dimension 2
0.2
0.0
−0.2
−0.4

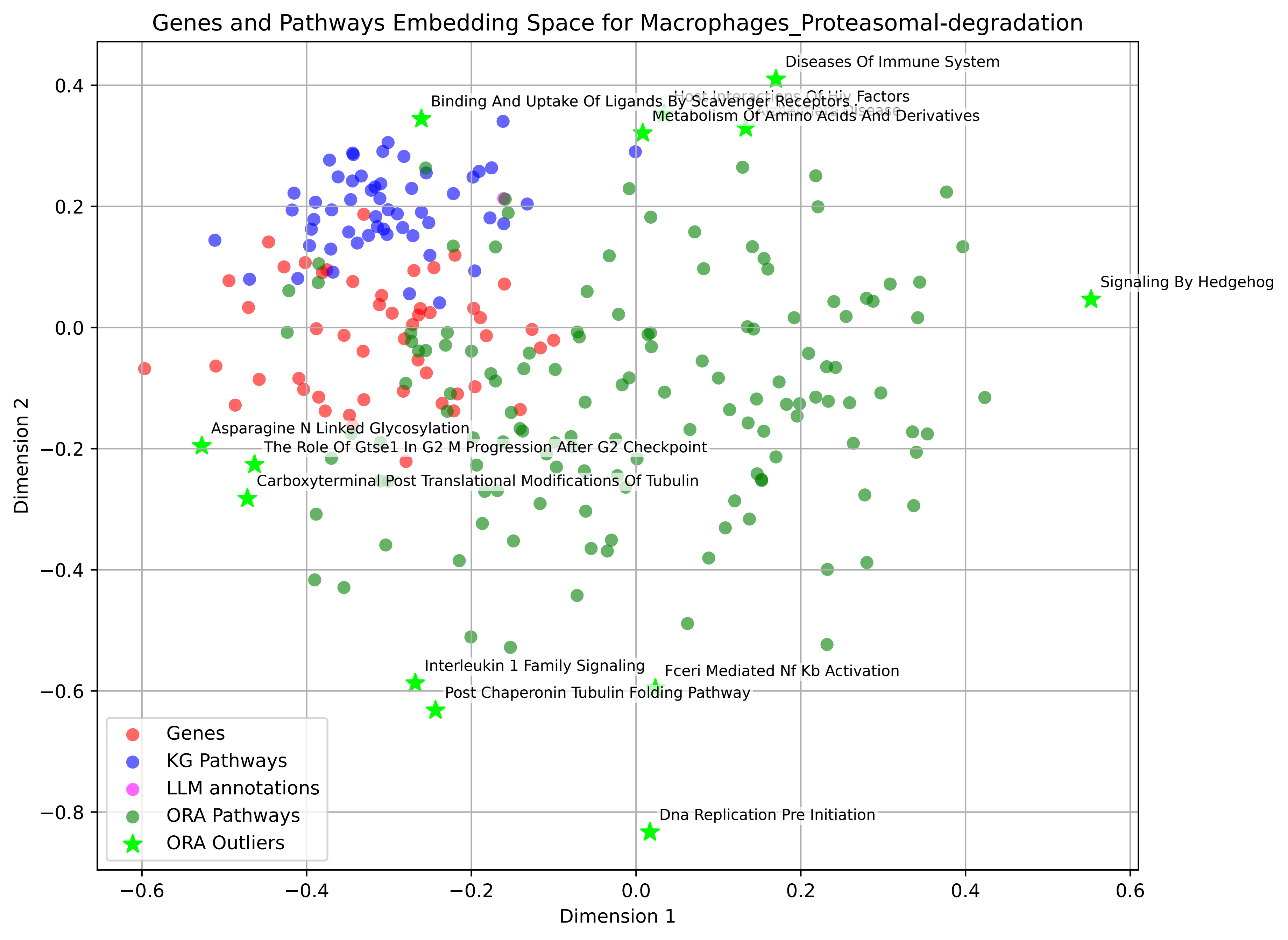
Genes and Pathways Embedding Space for Macrophages_Proteasomal-degradation
Diseases Of Immune System
Host Interactions Of Hiv Factors
Binding And Uptake Of Ligands By Scavenger Receptors
Alzheimers Disease
Metabolism Of Amino Acids And Derivatives
Signaling By Hedgehog
Asparagine N Linked Glycosylation
The Role Of Gtse1 In G2 M Progression After G2 Checkpoint
Carboxyterminal Post Translational Modifications Of Tubulin
Interleukin 1 Family Signaling
Fceri Mediated Nf Kb Activation
Post Chaperonin Tubulin Folding Pathway
Dna Replication Pre Initiation
Genes
KG Pathways
LLM annotations
ORA Pathways
ORA Outliers
Dimension 1
Dimension 2
−0.6
−0.4
−0.2
0.0
0.2
0.4
0.6
−0.8

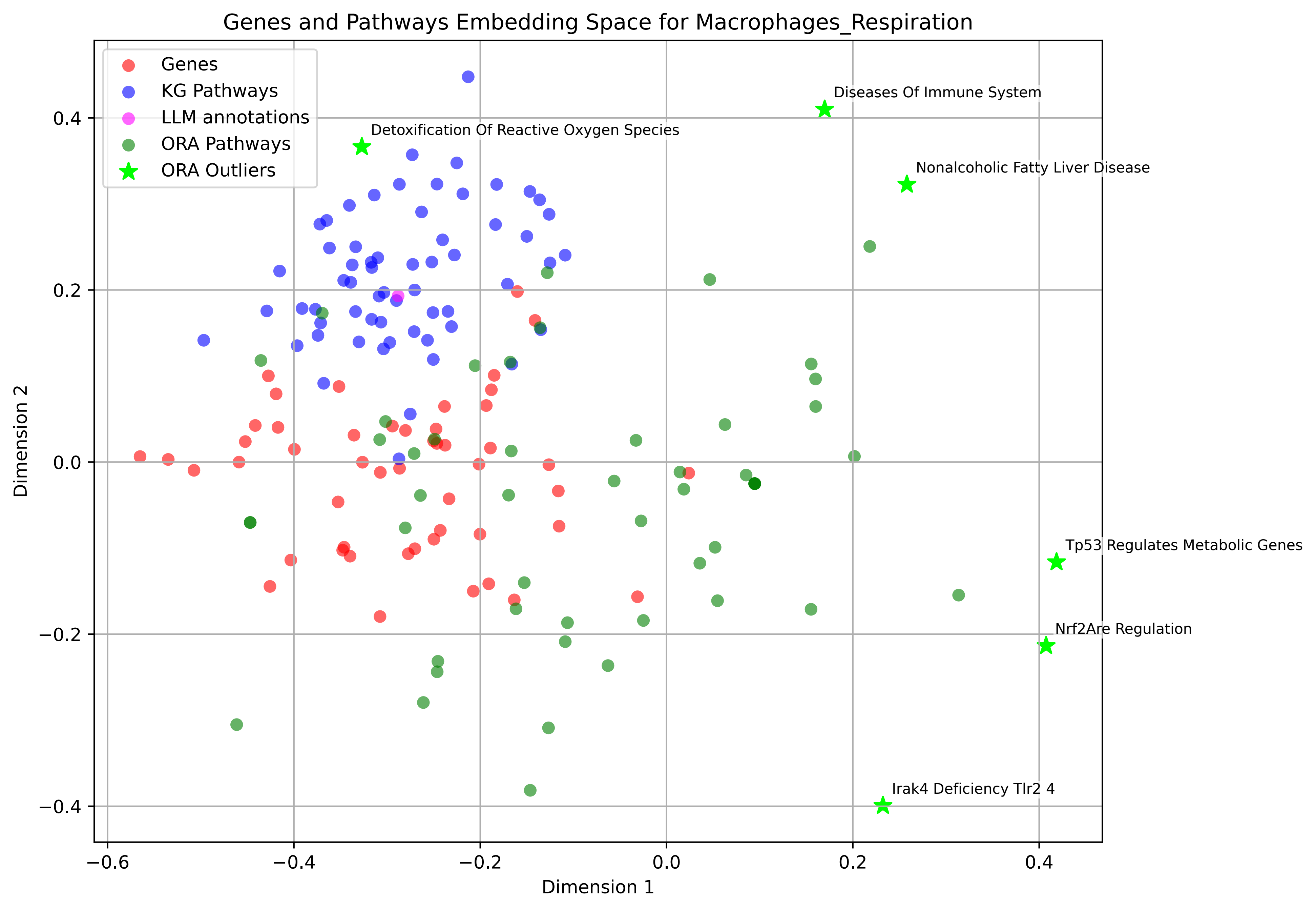
Genes and Pathways Embedding Space for Macrophages_Respiration
Genes
KG Pathways
LLM annotations
ORA Pathways
ORA Outliers
Diseases Of Immune System
Detoxification Of Reactive Oxygen Species
Nonalcoholic Fatty Liver Disease
Tp53 Regulates Metabolic Genes
Nrf2Are Regulation
Irak4 Deficiency Tlr2 4
Dimension 1
Dimension 2
−0.6
−0.4
−0.2
0.0
0.2
0.4
−0.4
−0.2
0.0
0.2
0.4

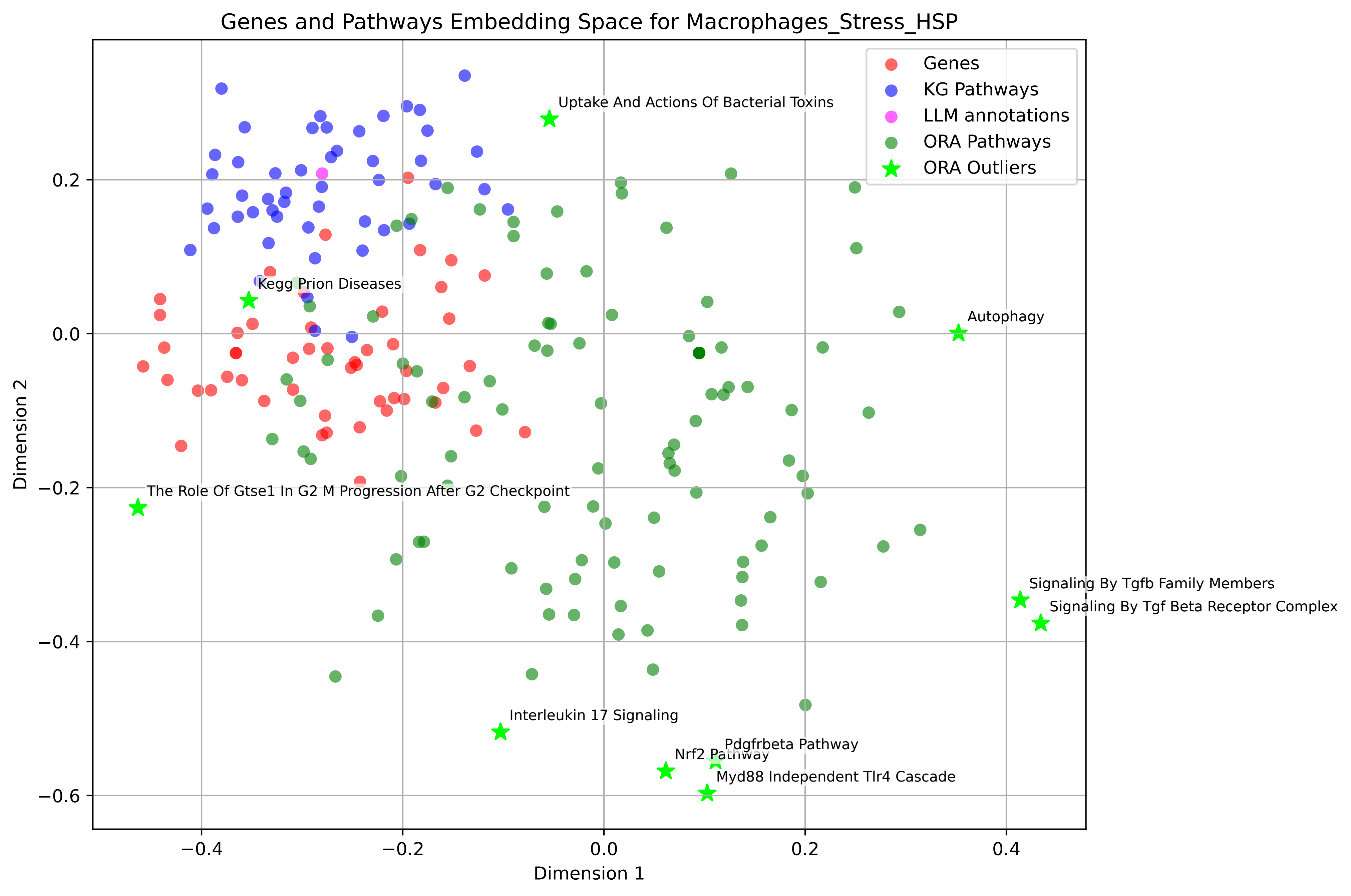
Genes and Pathways Embedding Space for Macrophages_Stress_HSP
Genes
KG Pathways
LLM annotations
ORA Pathways
ORA Outliers
Uptake And Actions Of Bacterial Toxins
Kegg Prion Diseases
Autophagy
The Role Of Gtse1 In G2 M Progression After G2 Checkpoint
Signaling By Tgfb Family Members
Signaling By Tgf Beta Receptor Complex
Interleukin 17 Signaling
Pdgfrbeta Pathway
Nrf2 Pathway
Myd88 Independent Tlr4 Cascade
Dimension 1
Dimension 2
0.2
0.0
−0.2
−0.4
−0.6
−0.4
−0.2
0.0
0.2
0.4

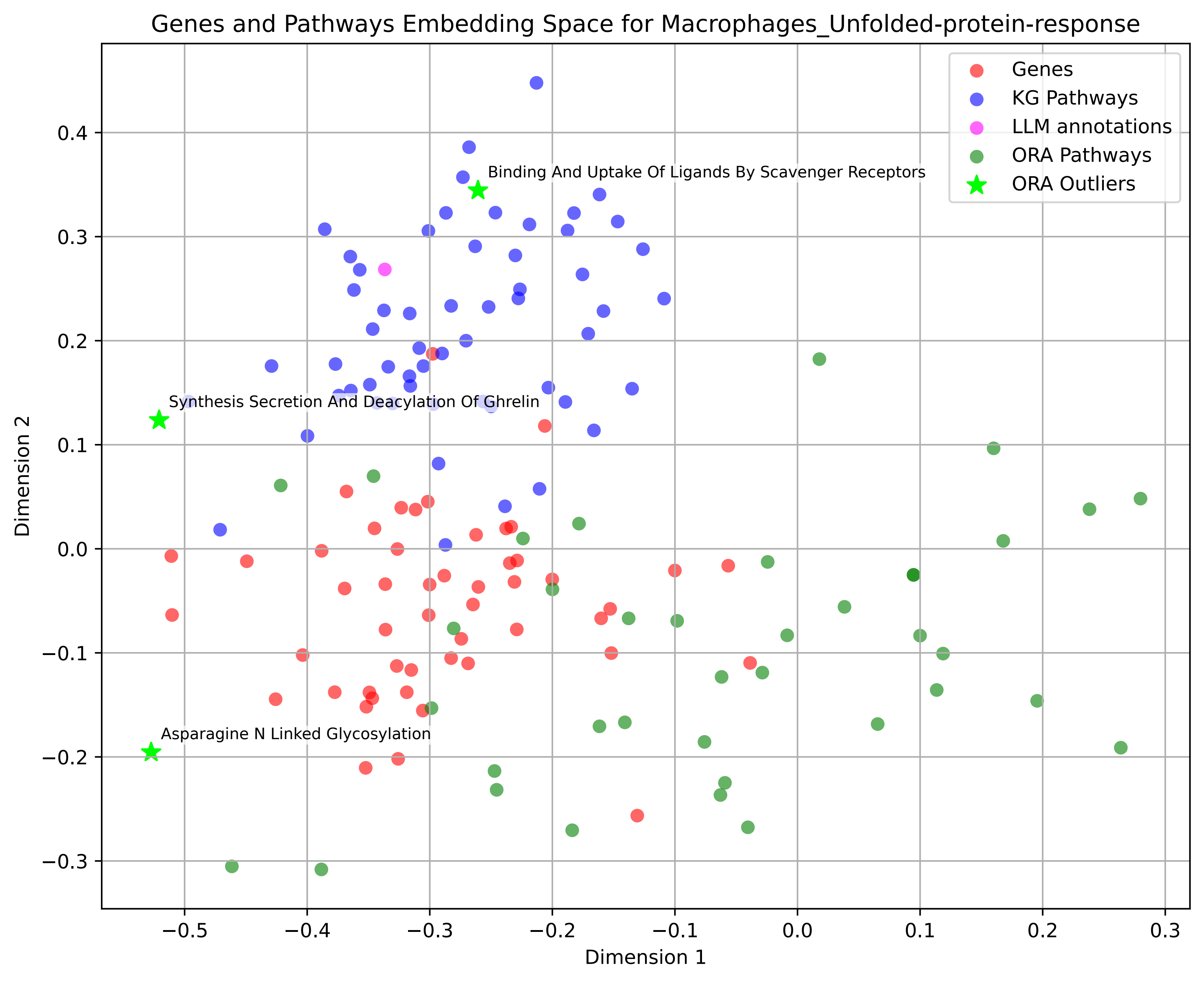

Genes and Pathways Embedding Space for Macrophages_Unfolded-protein-response
Genes
KG Pathways
LLM annotations
ORA Pathways
ORA Outliers
Binding And Uptake Of Ligands By Scavenger Receptors
Synthesis Secretion And Deacylation Of Ghrelin
Asparagine N Linked Glycosylation
Dimension 1
Dimension 2
0.4
0.3
0.2
0.1
0.0
−0.1
−0.2
−0.3
−0.5
−0.4
−0.3
−0.2
−0.1
0.0
0.1
0.2
0.3

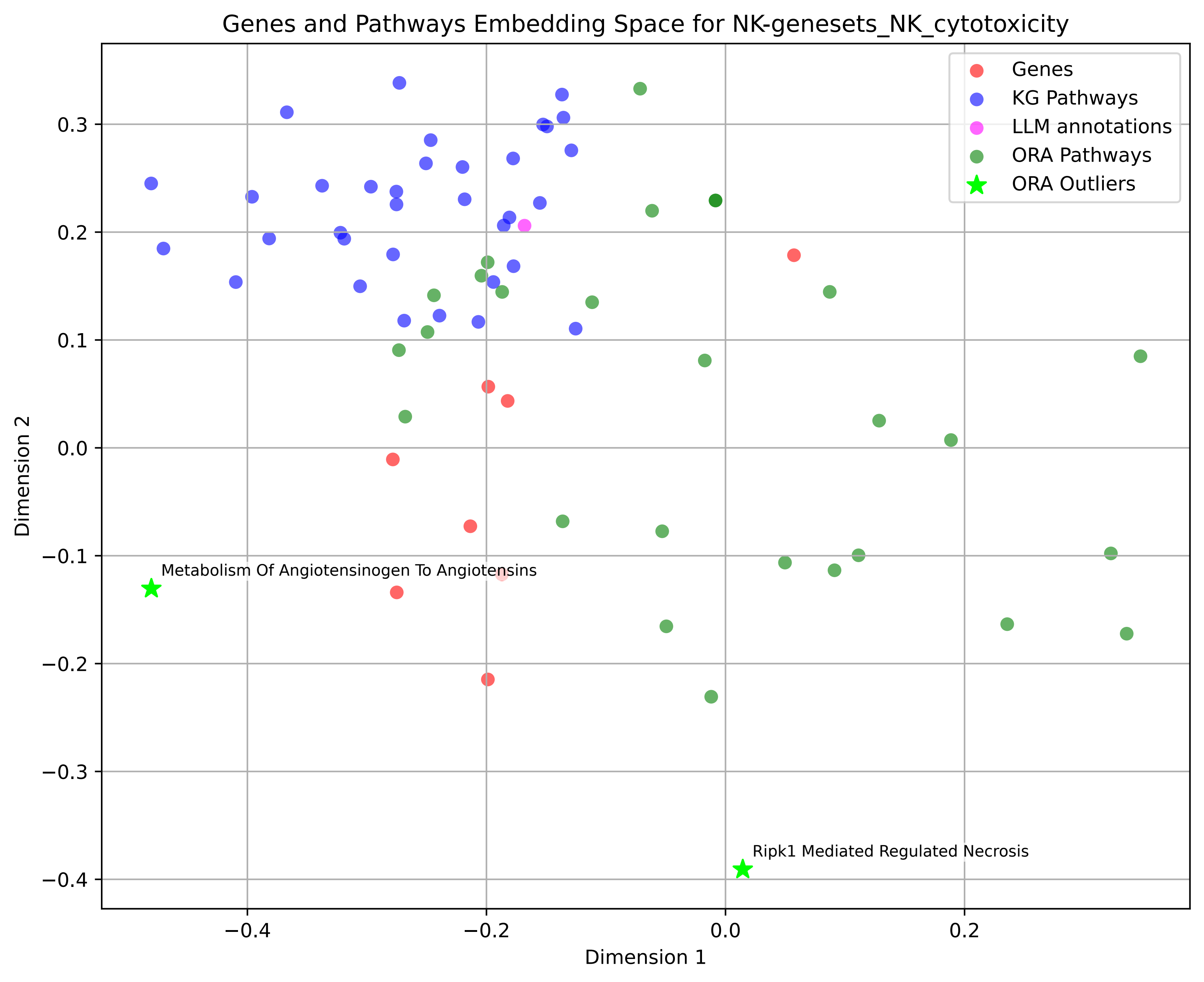
Genes and Pathways Embedding Space for NK-genesets_NK_cytotoxicity
Genes
KG Pathways
LLM annotations
ORA Pathways
ORA Outliers
Metabolism Of Angiotensinogen To Angiotensins
Ripk1 Mediated Regulated Necrosis
Dimension 1
Dimension 2
0.3
0.2
0.1
0.0
−0.1
−0.2
−0.3
−0.4
−0.4
−0.2
0.0
0.2

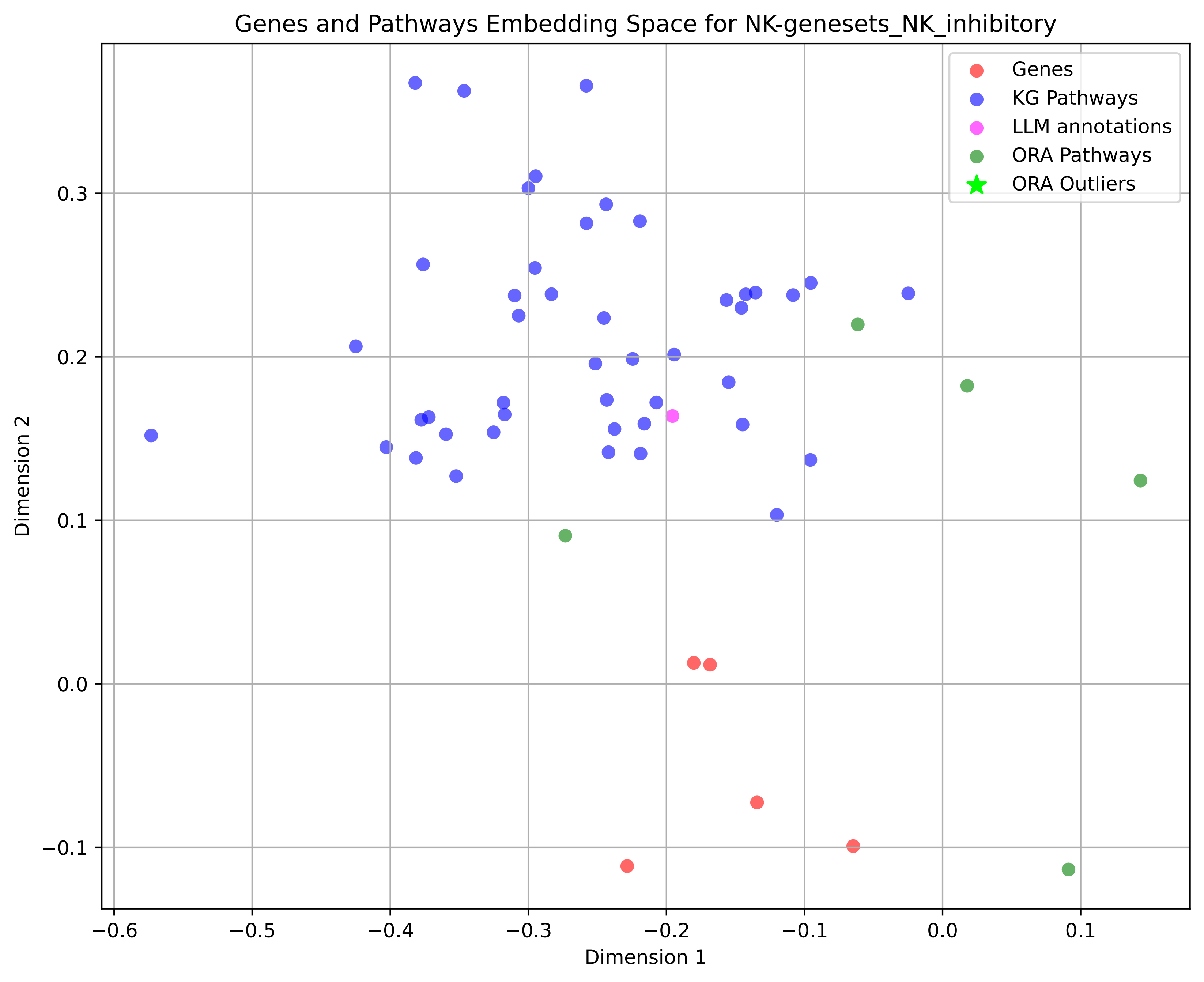

Genes and Pathways Embedding Space for NK-genesets_NK_inhibitory
Genes
KG Pathways
LLM annotations
ORA Pathways
ORA Outliers
Dimension 1
Dimension 2
−0.6
−0.5
−0.4
−0.3
−0.2
−0.1
0.0
0.1
−0.1
0.0
0.1
0.2
0.3

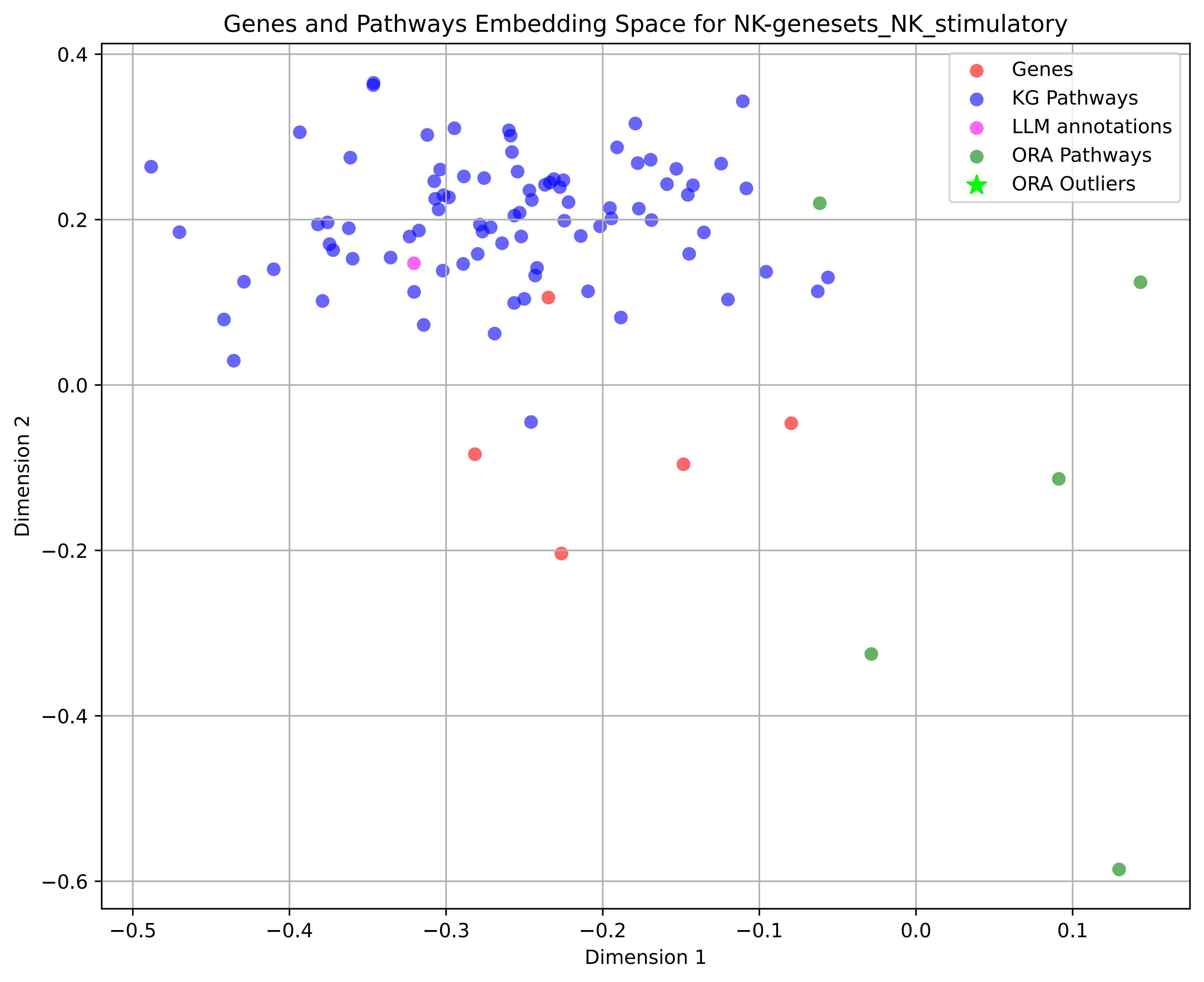

Genes and Pathways Embedding Space for NK-genesets_NK_stimulatory
Genes
KG Pathways
LLM annotations
ORA Pathways
ORA Outliers
Dimension 1
Dimension 2
0.4
0.2
0.0
−0.2
−0.4
−0.6
−0.5
−0.4
−0.3
−0.2
−0.1
0.0
0.1

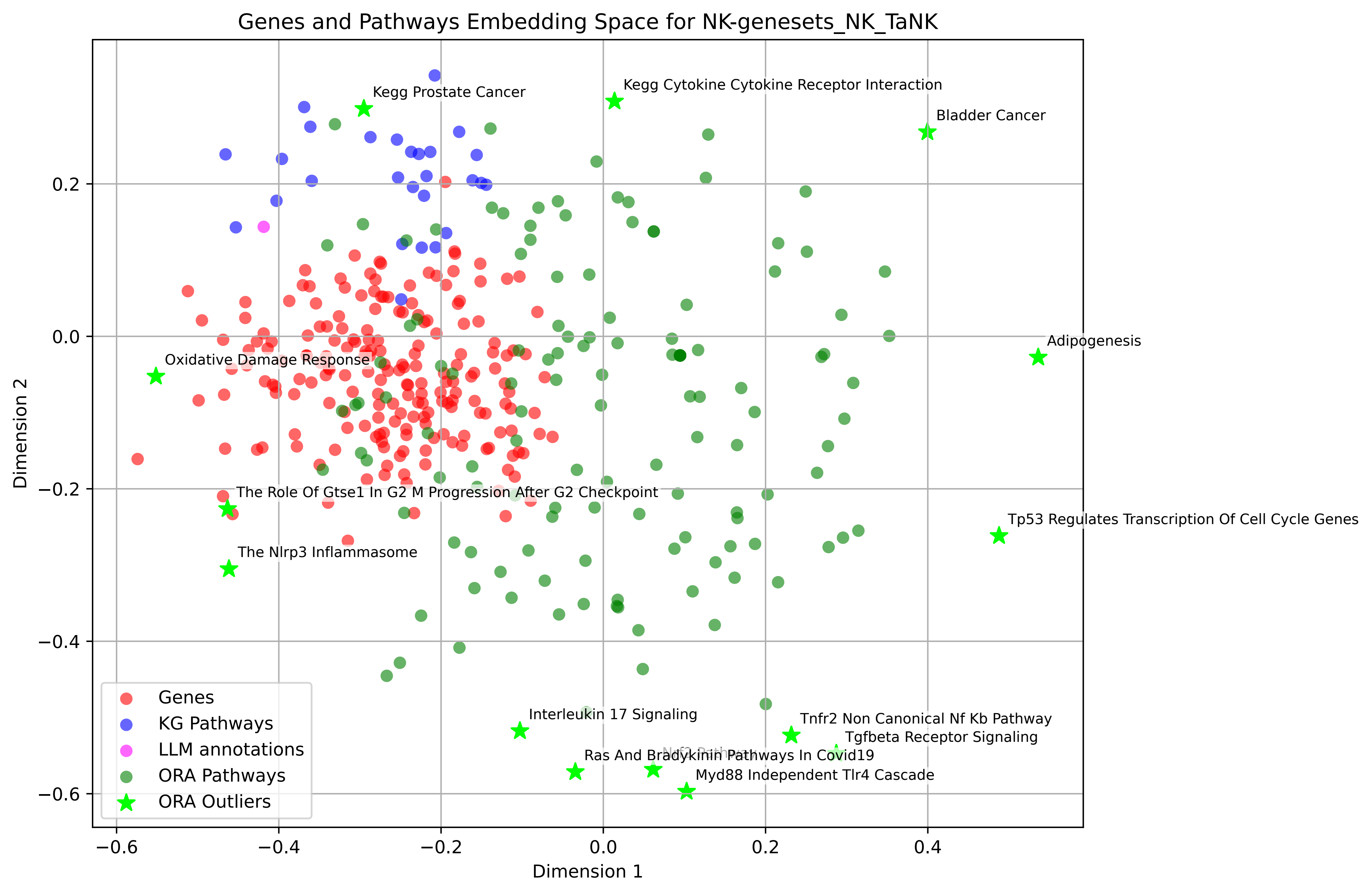
Genes and Pathways Embedding Space for NK-genesets_NK_TaNK
Dimension 1
Dimension 2
−0.6
−0.4
−0.2
0.0
0.2
0.4
Kegg Prostate Cancer
Kegg Cytokine Cytokine Receptor Interaction
Bladder Cancer
Adipogenesis
Oxidative Damage Response
The Role Of Gtse1 In G2 M Progression After G2 Checkpoint
Tp53 Regulates Transcription Of Cell Cycle Genes
The Nlrp3 Inflammasome
Interleukin 17 Signaling
Tnfr2 Non Canonical Nf Kb Pathway
Tgfbeta Receptor Signaling
Nrf2 Pathway
Ras And Bradykinin Pathways In Covid19
Myd88 Independent Tlr4 Cascade
Genes
KG Pathways
LLM annotations
ORA Pathways
ORA Outliers

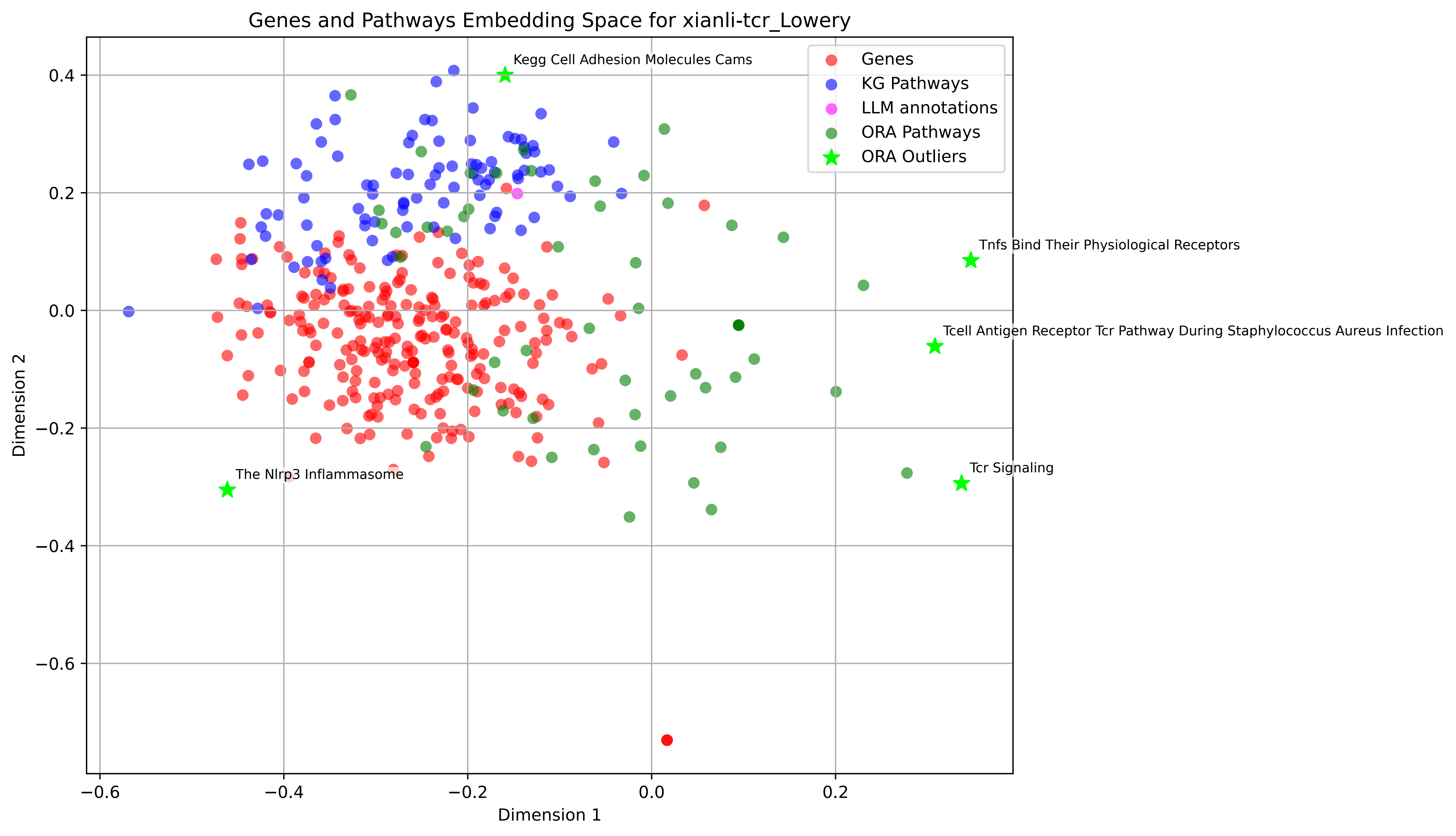

Genes and Pathways Embedding Space for xianli-tcr_Lowery
Kegg Cell Adhesion Molecules Cams
Tnfs Bind Their Physiological Receptors
Tcell Antigen Receptor Tcr Pathway During Staphylococcus Aureus Infection
Tcr Signaling
The Nlrp3 Inflammasome
Genes
KG Pathways
LLM annotations
ORA Pathways
ORA Outliers
Dimension 1
Dimension 2
−0.6
−0.4
−0.2
0.0
0.2
0.4

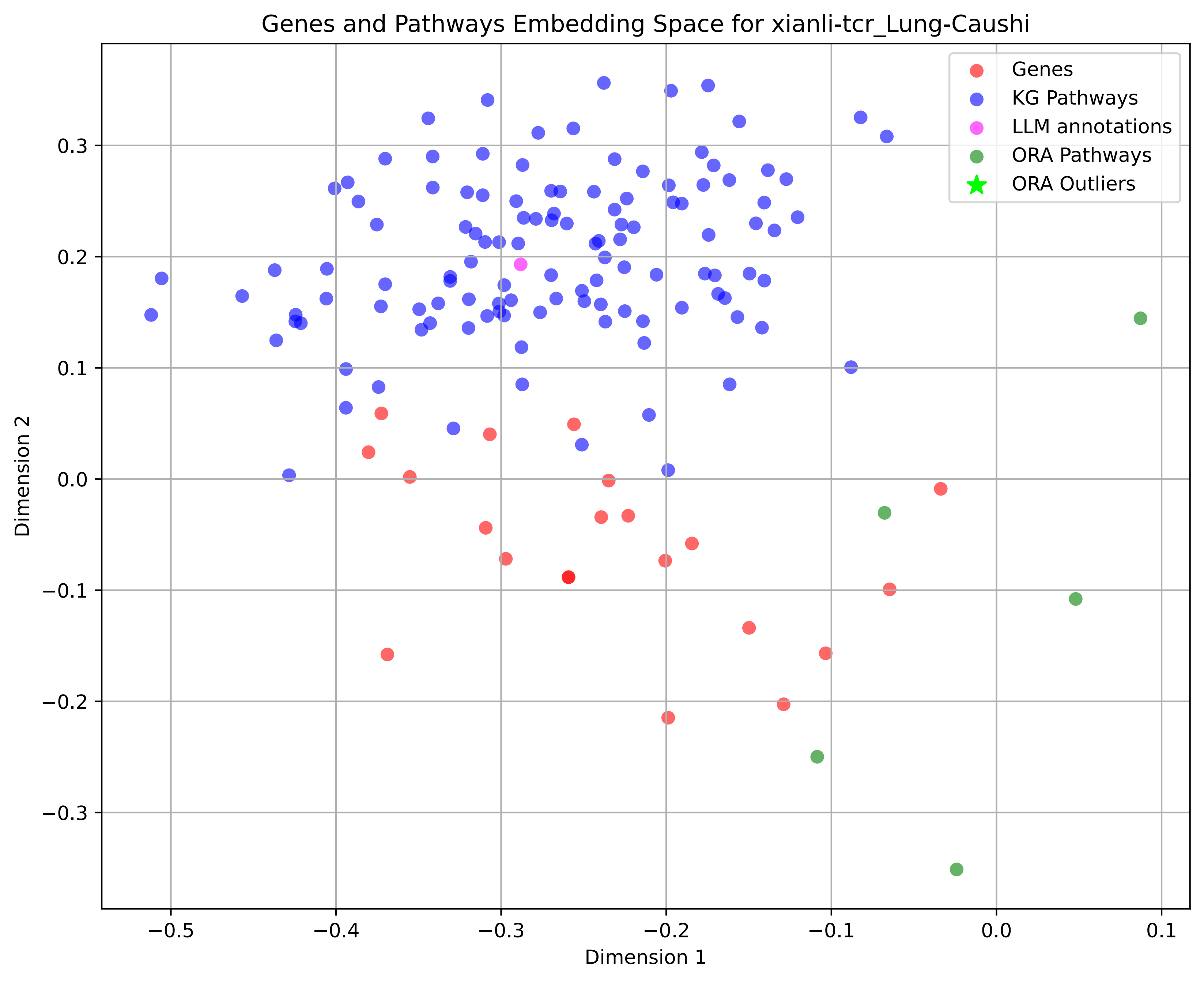
Genes and Pathways Embedding Space for xianli-tcr_Lung-Caushi
Genes
KG Pathways
LLM annotations
ORA Pathways
ORA Outliers
Dimension 1
Dimension 2
−0.5
−0.4
−0.3
−0.2
−0.1
0.0
0.1
0.3
0.2
0.1
0.0
−0.1
−0.2
−0.3

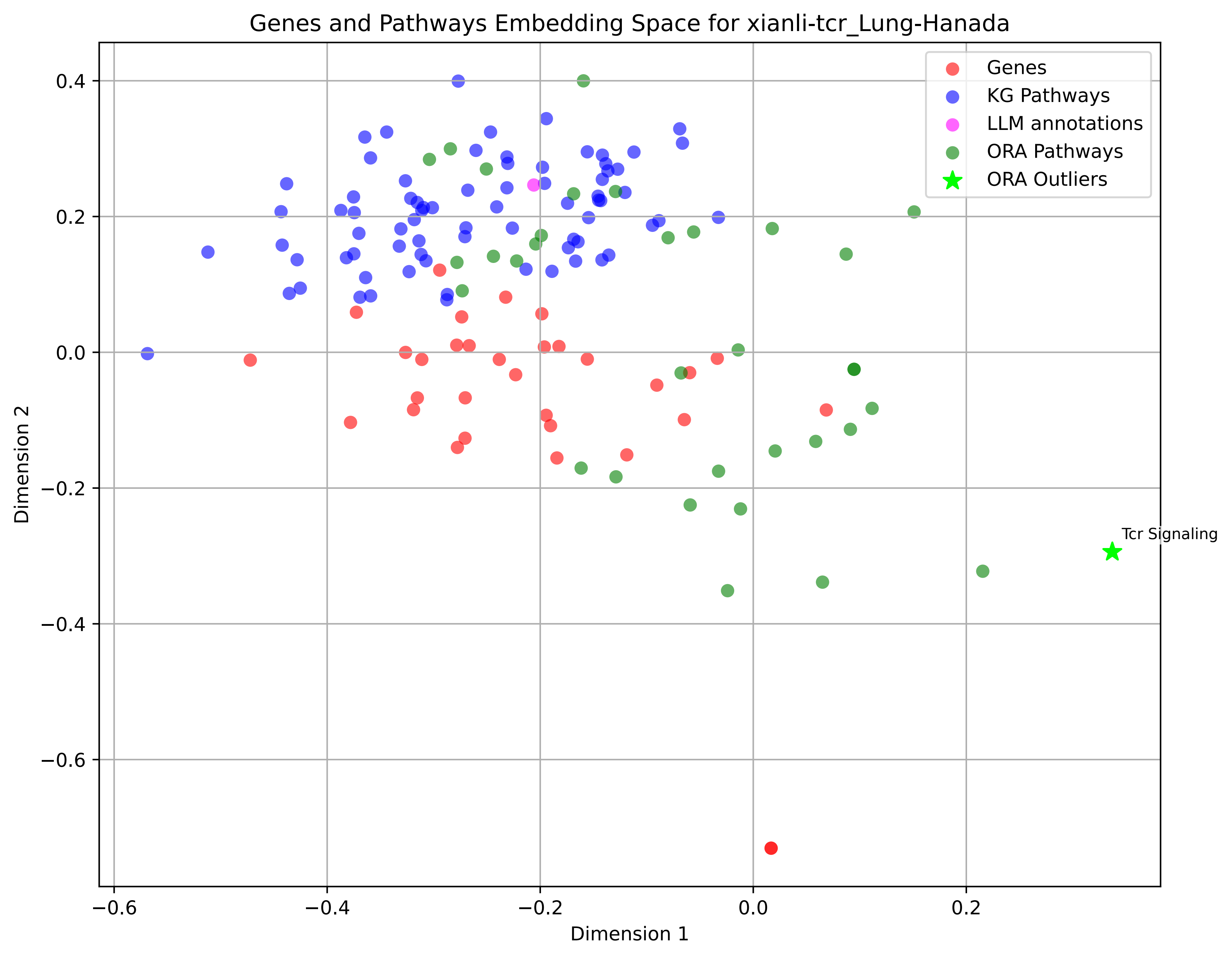

Genes and Pathways Embedding Space for xianli-tcr_Lung-Hanada
Genes
KG Pathways
LLM annotations
ORA Pathways
ORA Outliers
Tcr Signaling
Dimension 1
Dimension 2
0.4
0.2
0.0
−0.2
−0.4
−0.6
−0.6
−0.4
−0.2
0.0
0.2

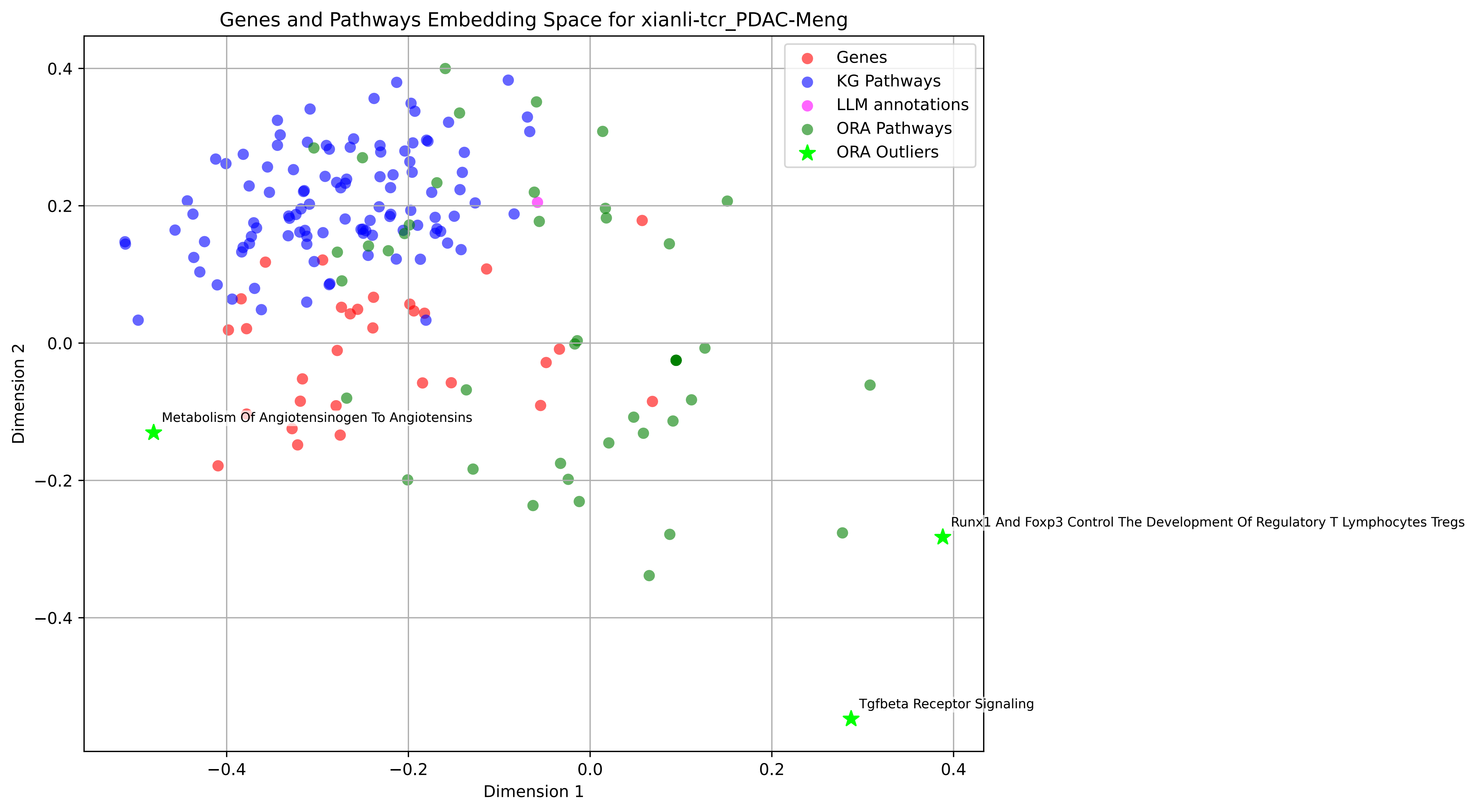
Genes and Pathways Embedding Space for xianli-tcr_PDAC-Meng
Genes
KG Pathways
LLM annotations
ORA Pathways
ORA Outliers
Metabolism Of Angiotensinogen To Angiotensins
Runx1 And Foxp3 Control The Development Of Regulatory T Lymphocytes Tregs
Tgfbeta Receptor Signaling
Dimension 1
Dimension 2
0.4
0.2
0.0
−0.2
−0.4
−0.4
−0.2
0.0
0.2
0.4

Figure S6. Two-dimensional representation of genes in each gene set and respective enriched pathways in the BERT embedding space. Individual genes (red dots) are shown alongside two types of pathways: ICKG Pathways (blue dots) and ORA Pathways (green dots). The proximity between dots suggests semantic or functional proximity.

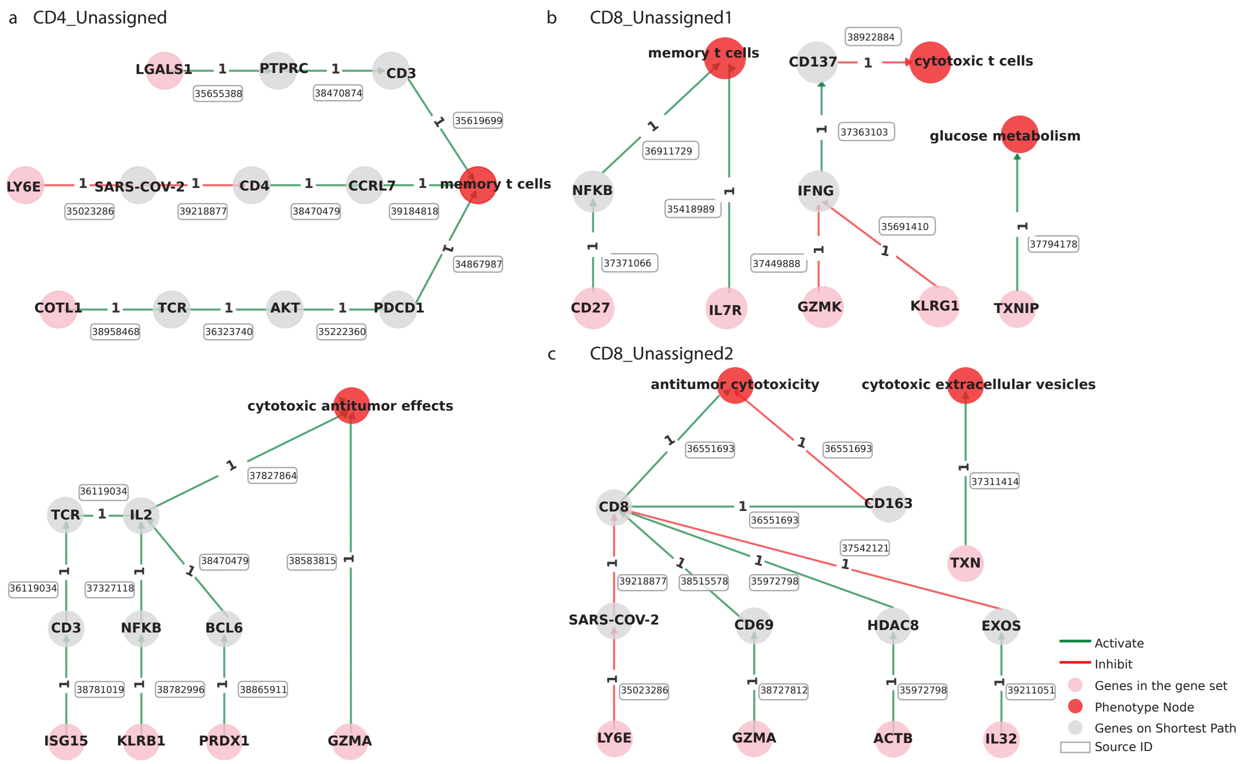

Figure S7. Subgraphs queried from the T cell ICKG for (a) CD4_Unassigned, (b) CD8_Unassigned1, and (c) CD8_Unassigned2. In all networks, pink nodes represent genes in the corresponding gene sets, red nodes indicate the phenotype that these pink genes are linked to, and gray nodes show genes on the shortest paths between gene set members and phenotypes. Green arrows represent activation relationships, while red arrows represent inhibition. Each edge is labeled by a PMID referencing the primary literature that inferred that relationship.

```
system_prompt = """
  You are a computational biologist who excels at carefully reading biomedical publication
  abstracts and inferring the relationship between biomedical terms based on the abstract
  """
user_prompt = f"""
  I will provide you with an Abstract and multiple pairs of Biomedical terms.
  Abstract: {abstract}

  For each pair of terms, determine if there is a directional relationship from the first term to the second term.
  The relationship should be one of the following three options:
  - 'Inhibit' if the first term directly or indirectly reduces, blocks, or inhibits the second term.
  - 'Activate' if the first term directly or indirectly promotes or leads to the second term.
  - 'no association' if there is no clear direct or indirect relationship from the first term to the
    second term, or if you are unsure about the relationship based on the provided information.

  Important rules to follow:
  - The relationship must be inferred in the direction from the first term to the second term only.
    Do not infer relationships in the opposite direction unless explicitly stated in the sentence.
  - If the relationship is ambiguous or unclear in the abstract, choose 'no association.'
  - If either term lacks a clear biological meaning in the context of the abstract, output 'no association.'
  - After determining the relationship, validate the answer by re-reading the abstract.
    If you cannot confidently validate the direction of the relationship, then change your output to 'no association'.

  Biomedical term pairs:
  {', '.join([f'({pair[0]}, {pair[1]})' for pair in entity_pairs])}
  **Remember:**
  - If term1 inhibits term2, the output should be (term1, term2, Inhibit).
  - If term1 activates term2, the output should be (term1, term2, Activate).
  - If term1 does not affect term2 or you cannot validate it, output (term1, term2, no association).
  - If term2 does not directly or indirectly affect term1, the output should always be (term2, term1, no association).
  Output your response in the following format: ('term1', 'term2', relationship) and this format only.
  Examples:
  - "TNF-α stimulates NF-κB activity, resulting in increased expression of proinflammatory genes."
    - If term1 is TNF-α and term2 is NF-κB, the output is (TNF-α, NF-κB, Activate).
    - If term1 is NF-κB and term2 is TNF-α, the output is (NF-κB, TNF-α, no association).
  - "PD-1 signaling inhibits T cell activation, leading to immune evasion by tumor cells."
    - If term1 is PD-1 and term2 is T cell activation, the output is (PD-1, T cell activation, Inhibit).
    - If term1 is T cell activation and term2 is PD-1, the output is (T cell activation, PD-1, no association).

  Output your response in the following format for each pair:
  (term1, term2, relationship)

  Only include pairs where the relationship is either 'Activate' or 'Inhibit'.
  """
```

The design of this AI-driven prompt is tailored to replicate the analytical rigor a computational biologist applies when interpreting biomedical abstracts.

Specify the input and question of interest as detailed as possible. Provide possible answers to avoid generating unwanted outputs.

The requirement to confirm the validity of these relationships before finalizing them emphasizes the importance of precision and mitigates the risk of misinterpretation, ensuring the AI's outputs are both accurate and scientifically reliable.

Including examples helps:
(1) Clarify the expected outcomes by demonstrating how the AI should interpret and categorize interactions between terms based on the context provided in biomedical abstracts. This is essential for setting clear expectations and standards for the analysis.
(2) Assist in training the AI to recognize and differentiate between activating and inhibiting relationships, by showcasing specific instances of how complex biological interactions are reported in literatures.

Stipulate the format of the output to ensure smooth downstream data analysis.

Figure S8. The figure shows a prompt designed to guide an AI in identifying activation or inhibition relationships between biomedical terms within abstracts. The left panel presents the full prompt text, while the right panel provides brief annotations explaining the purpose of each section, including instructions, rules, and output formatting.

Plasma	KG_annotation (ranked by pagerank scor	ORA_annotation	LLM_annotation
DERL3	nanoscale plasma membrane organizatio	KEGG_PROTEIN_EXPORT	Endoplasmic Reticulum Associated Protein Degradation (ERAD) and Immunoglobulin Assembly (0.85)
MZB1	immune membrane organization	HALLMARK_UNFOLDED_PROTEIN_RESPONSE	
JCHAIN	cell cycle	REACTOME_SRP_DEPENDENT_COTRANSLATIONAL_PROTEIN_TARGETING_TO_MEMBRANE	
PRDX4	disease-free survival	REACTOME_UNFOLDED_PROTEIN_RESPONSE_UPR	
XBP1	cell-derived lymphomas	WP_PHOTODYNAMIC_THERAPYINDUCED_UNFOLDED_PROTEIN_RESPONSE	
SEC11C	cancer immunosurveillance	REACTOME_SYNTHESIS_SECRETION_AND_INACTIVATION_OF_GLUCOSE_DEPENDENT_INSULINOTROPIC_POLYPEPTIDE_GIP	
FKBP11	hematopoietic cellular products	REACTOME_SYNTHESIS_SECRETION_AND_DEACYLATION_OF_GHRELIN	
FKBP2	standard allogeneic grafts	REACTOME_IRE1ALPHA_ACTIVATES_CHAPERONES	
IGHG3	anticancer-directed immune responses	REACTOME_SYNTHESIS_SECRETION_AND_INACTIVATION_OF_GLUCAGON_LIKE_PEPTIDE_1_GLP_1	
IGHG1	death receptor	REACTOME_BINDING_AND_UPTAKE_OF_LIGANDS_BY_SCAVENGER_RECEPTORS	
ITM2C	memory-like responses	REACTOME_INCRETIN_SYNTHESIS_SECRETION_AND_INACTIVATION	
IGKC	proinflammatory cytokine production	REACTOME_FCGR_ACTIVATION	
IGLC2	degranulation	REACTOME_SCAVENGING_OF_HEME_FROM_PLASMA	
IGLC3	response heterogeneity	REACTOME_CREATION_OF_C4_AND_C2_ACTIVATORS	
HSP90B1	neutrophil-mediated destruction	REACTOME_TRANSLATION	
IGHA1	complement-dependent cytotoxicity	REACTOME_ATF6_ATF6_ALPHA_ACTIVATES_CHAPERONE_GENES	
SSR4	gene rearrangement	REACTOME_INITIAL_TRIGGERING_OF_COMPLEMENT	
IGHG4	early-minted antibody secreting cells	REACTOME_ROLE_OF_PHOSPHOLIPIDS_IN_PHAGOCYTOSIS	
NUCB2	lymphoma pathogenesis	REACTOME_ATF6_ATF6_ALPHA_ACTIVATES_CHAPERONES	
SPCS3		REACTOME_FCGR3A_MEDIATED_IL10_SYNTHESIS	
MANF		REACTOME_CELL_SURFACE_INTERACTIONS_AT_THE_VASCULAR_WALL	
SSR3		REACTOME_COMPLEMENT_CASCADE	
SDF2L1		REACTOME_PARASITE_INFECTION	
MYDGF		WP_VEGFAVEGFR2_SIGNALING_PATHWAY	
IGHG2		REACTOME_FCGAMMA_RECEPTOR_FCGR_DEPENDENT_PHAGOCYTOSIS	
PPIB		REACTOME_PEPTIDE_HORMONE_METABOLISM	
SPCS1		HALLMARK_MTORC1_SIGNALING	
IGHA2		REACTOME_ANTI_INFLAMMATORY_RESPONSE_FAVOURING_LEISHMANIA_PARASITE_INFECTION	
HSPA5		REACTOME_LEISHMANIA_INFECTION	
CD63		WP_UNFOLDED_PROTEIN_RESPONSE	
LMAN1		HALLMARK_ANDROGEN_RESPONSE	
IGHGP		REACTOME_PERK_REGULATES_GENE_EXPRESSION	
PDIA4		WP_TYPE_I_COLLAGEN_SYNTHESIS_IN_THE_CONTEXT_OF_OSTEOGENESIS_IMPERFECTA	
HERPUD1		REACTOME_INTERLEUKIN_4_AND_INTERLEUKIN_13_SIGNALING	
DNAJB9		WP_PRION_DISEASE_PATHWAY	
ERLEC1		REACTOME_REGULATION_OF_INSULIN_LIKE_GROWTH_FACTOR_IGF_TRANSPORT_AND_UPTAKE_BY_INSULIN_LIKE_GROWTH_FACTOR_BINDING_PROTEINS_IGFBPS	
RPN2		REACTOME_RESPONSE_TO_ELEVATED_PLATELET_CYTOSOLIC_CA2	
SEC61B		WP_ENDOPLASMIC_RETICULUM_STRESS_RESPONSE_IN_CORONAVIRUS_INFECTION	
PDIA6		KEGG_VIBRIO_CHOLERAE_INFECTION	
SPCS2		REACTOME_SIGNALING_BY_INTERLEUKINS	
TMEM258			
CRELD2			
RABAC1			
SDC1			
KRTCAP2			
PIM2			
TNFRSF17			
TMEM59			
VIMP			
P4HB			

MHC-II	KG_annotation (ranked by pagerank scor	ORA_annotation	LLM_annotation
HLA-DPB1	antigen presentation	KEGG_VIRAL_MYOCARDITIS	B-cell Activation and Immune Response (0.95)
HLA-DRA	immune response	KEGG_ASTHMA	
HLA-DRB1	lymphocyte depletion	KEGG_INTESTINAL_IMMUNE_NETWORK_FOR_IGA_PRODUCTION	
LAPTM5	systemic inflammatory response syndrom	KEGG_ALLOGRAFT_REJECTION	
HLA-DPA1	immune signaling	KEGG_GRAFT_VERSUS_HOST_DISEASE	
LTB	nanoscale plasma membrane organizatio	WP_EBOLA_VIRUS_INFECTION_IN_HOST	
HLA-DQB1	immune membrane organization	KEGG_TYPE_I_DIABETES_MELLITUS	
HLA-DQA1	pro-survival pathways	KEGG_AUTOIMMUNE_THYROID_DISEASE	
MS4A1	extracellular signal-regulated kinase	KEGG_ANTIGEN_PROCESSING_AND_PRESENTATION	
CD52	cell-derived lymphomas	KEGG_CELL_ADHESION_MOLECULES_CAMS	
CD37	eukaryotic cell migration	KEGG_LEISHMANIA_INFECTION	
COTL1	disease-free survival	REACTOME_PD_1_SIGNALING	
CORO1A	hematopoietic cellular products	REACTOME_MHC_CLASS_II_ANTIGEN_PRESENTATION	
BANK1	standard allogeneic grafts	WP_ALLOGRAFT_REJECTION	
CD69	clonal tumor evolution	REACTOME_GENERATION_OF_SECOND_MESSENGER_MOLECULES	
HLA-DMB	tissue-specific splicing	KEGG_SYSTEMIC_LUPUS_ERYTHEMATOSUS	
CXCR4	plasmablastic lymphoma	REACTOME_COSTIMULATION_BY_THE_CD28_FAMILY	
SMIM14	cancer immunosurveillance	REACTOME_TCR_SIGNALING	
CD53	co-receptor function	HALLMARK_INTERFERON_GAMMA_RESPONSE	
LIMD2	extranodal lymphoma	REACTOME_INTERFERON_GAMMA_SIGNALING	
ARHGDIB	anticancer-directed immune responses	HALLMARK_ALLOGRAFT_REJECTION	
HLA-DMA	lymphoma biology	REACTOME_INTERFERON_SIGNALING	
BTG1	primary cutaneous follicle center lymphor	KEGG_REGULATION_OF_ACTIN_CYTOSKELETON	
CD48	follicular lymphoma	KEGG_HEMATOPOIETIC_CELL_LINEAGE	
SELL	neutrophil-mediated destruction	KEGG_LEUKOCYTE_TRANSENDOTHELIAL_MIGRATION	
TXNIP	complement-dependent cytotoxicity	WP_PATHOGENESIS_OF_SARSCOV2_MEDIATED_BY_NSP9NSP10_COMPLEX	
VPREB3	lymphoma pathogenesis	HALLMARK_TNFA_SIGNALING_VIA_NFKB	
CD79B		WP_NETWORK_MAP_OF_SARSCOV2_SIGNALING_PATHWAY	
CD83		WP_REGULATION_OF_ACTIN_CYTOSKELETON	
ACTB		WP_ACUTE_VIRAL_MYOCARDITIS	
HLA-DRB5		REACTOME_RHO_GTPASES_ACTIVATE_WASPS_AND_WAVES	
ZFAS1		REACTOME_EPH_EPHRIN_SIGNALING	
PTPRC		HALLMARK_INTERFERON_ALPHA_RESPONSE	
ACTG1		WP_B_CELL_RECEPTOR_SIGNALING_PATHWAY	
CD74		REACTOME_EPHB_MEDIATED_FORWARD_SIGNALING	
MYL12A		WP_FAS_LIGAND_PATHWAY_AND_STRESS_INDUCTION_OF_HEAT_SHOCK_PROTEINS	
REL		HALLMARK_IL2_STAT5_SIGNALING	
ARPC1B		HALLMARK_APICAL_JUNCTION	
SH3BGRL3		REACTOME_GAP_JUNCTION_DEGRADATION	
NAP1L1		WP_PATHOGENIC_ESCHERICHIA_COLI_INFECTION	
RAC2		KEGG_PATHOGENIC_ESCHERICHIA_COLI_INFECTION	
STK17A		WP_OVERLAP_BETWEEN_SIGNAL_TRANSDUCTION_PATHWAYS_CONTRIBUTING_TO_LMNA_LAMINOPATHIES	
GPSM3		REACTOME_CELL_EXTRACELLULAR_MATRIX_INTERACTIONS	
LY86		KEGG_ADHERENS_JUNCTION	
PFN1		WP_CYTOKINES_AND_INFLAMMATORY_RESPONSE	
GPR183		WP_VEGFAVEGFR2_SIGNALING_PATHWAY	
IFITM2		REACTOME_CELL_SURFACE_INTERACTIONS_AT_THE_VASCULAR_WALL	
JUNB		KEGG_FC_GAMMA_R_MEDIATED_PHAGOCYTOSIS	
LCP1		KEGG_FOCAL_ADHESION	
TMSB4X		REACTOME_RHO_GTPASE_EFFECTORS	
		HALLMARK_INFLAMMATORY_RESPONSE	
		HALLMARK_KRAS_SIGNALING_UP	
		WP_FOCAL_ADHESION	
		REACTOME_INTERACTION_BETWEEN_L1_AND_ANKYRINS	
		REACTOME_RHO_GTPASES_ACTIVATE_IQGAPS	
		REACTOME_ADHERENS_JUNCTIONS_INTERACTIONS	
		WP_HOSTPATHOGEN_INTERACTION_OF_HUMAN_CORONAVIRUSES_MAPK_SIGNALING	
		WP_NEOVASCULARISATION_PROCESSES	
		REACTOME_PARASITE_INFECTION	
		REACTOME_MAP2K_AND_MAPK_ACTIVATION	
		WP_G13_SIGNALING_PATHWAY	
		WP_MICROGLIA_PATHOGEN_PHAGOCYTOSIS_PATHWAY	
		WP_COMMON_PATHWAYS_UNDERLYING_DRUG_ADDICTION	
		KEGG_TIGHT_JUNCTION	
		REACTOME_SIGNALING_BY_MODERATE_KINASE_ACTIVITY_BRAF_MUTANTS	
		WP_MECHANOREGULATION_AND_PATHOLOGY_OF_YAPTAZ_VIA_HIPPO_AND_NONHIPPO_MECHANISMS	
		REACTOME_RHO_GTPASES_ACTIVATE_FORMINS	
		REACTOME_RECYCLING_PATHWAY_OF_L1	
		WP_EXERCISEINDUCED_CIRCADIAN_REGULATION	

Cell-cycle	KG_annotation (ranked by pagerank scor	ORA_annotation	LLM_annotation
HMGB2	proliferation markers	HALLMARK_E2F_TARGETS	Cell Cycle Regulation and Mitosis (0.95)
STMN1	cell survival	HALLMARK_G2M_CHECKPOINT	
TUBA1B	gene rearrangement	WP_RETINOBLASTOMA_GENE_IN_CANCER	
TUBB	complete remission	REACTOME_M_PHASE	
HMGN2	median duration	HALLMARK_MYC_TARGETS_V1	
PTTG1	early-minted antibody secreting cells	REACTOME_MITOTIC_METAPHASE_AND_ANAPHASE	
DUT	tumour-like effects	REACTOME_MITOTIC_PROMETAPHASE	
NUSAP1	adaptive immune function	REACTOME_MITOTIC_G1_PHASE_AND_G1_S_TRANSITION	
H2AFZ	plasma cell dyscrasias	WP_GLYCOLYSIS_IN_SENESCENCE	
PCNA	tumor beds	REACTOME_G1_S_SPECIFIC_TRANSCRIPTION	
HMGA1	epithelial ovarian cancer	WP_AEROBIC_GLYCOLYSIS	
PCLAF	immune checkpoint inhibitors	REACTOME_RESOLUTION_OF_SISTER_CHROMATID_COHESION	
CKS1B	antigen-specific long-term protection	REACTOME_SEPARATION_OF_SISTER_CHROMATIDS	
RANBP1	plasma cell removal	REACTOME_RHO_GTPASE_EFFECTORS	
SMC4	virus elimination	REACTOME_CONDENSATION_OF_PROPHASE_CHROMOSOMES	
HMGB1	clinical diagnosis	REACTOME_CELL_CYCLE_CHECKPOINTS	
TK1	unique genetic events	REACTOME_RHO_GTPASES_ACTIVATE_FORMINS	
MKI67	autophagy-related genes	WP_METABOLIC_REPROGRAMMING_IN_COLON_CANCER	
LDHA	plasma cell accumulation	WP_GLYCOLYSIS_AND_GLUCONEOGENESIS	
TYMS	heterogeneous lifespans	REACTOME_SUMOYLATION_OF_DNA_REPLICATION_PROTEINS	
TOP2A	immune microenvironment changes	REACTOME_MITOTIC_SPINDLE_CHECKPOINT	
H4C3	systemic mucinosis	REACTOME_DNA_REPLICATION	
HIST1H4C	dermato-neuro syndrome	KEGG_GLYCOLYSIS_GLUCONEOGENESIS	
RAN	hematopoietic stem cell transplantation	REACTOME_DEPOSITION_OF_NEW_CENPA_CONTAINING_NUCLEOSOMES_AT_THE_CENTROMERE	
TCL1A	lymphoma pathogenesis	REACTOME_CHROMOSOME_MAINTENANCE	
UBE2C		REACTOME_G0_AND_EARLY_G1	
ENO1		REACTOME_MITOTIC_PROPHASE	
H2AZ1		REACTOME_NUCLEAR_ENVELOPE_NE_REASSEMBLY	
CENPM		REACTOME_INTERCONVERSION_OF_NUCLEOTIDE_DI_AND_TRIPHOSPHATES	
SLC25A5		REACTOME_DNA_DAMAGE_TELOMERE_STRESS_INDUCED_SENESCENCE	
MCM7		WP_PYRIMIDINE_METABOLISM	
SNRPD1		WP_FLUOROPYRIMIDINE_ACTIVITY	
AURKB		WP_CLEAR_CELL_RENAL_CELL_CARCINOMA_PATHWAYS	
CDK1		REACTOME_APC_C_MEDIATED_DEGRADATION_OF_CELL_CYCLE_PROTEINS	
DEK		REACTOME_TRANSCRIPTIONAL_REGULATION_OF_GRANULOPOIESIS	
PKM		WP_COHESIN_COMPLEX_CORNELIA_DE_LANGE_SYNDROME	
TUBB4B		REACTOME_B_WICH_COMPLEX_POSITIVELY_REGULATES_RRNA_EXPRESSION	
ANP32B		REACTOME_BASE_EXCISION_REPAIR	
ANP32E		KEGG_GAP_JUNCTION	
BIRC5		WP_PHOTODYNAMIC_THERAPYINDUCED_HIF1_SURVIVAL_SIGNALING	
CDKN3		REACTOME_RECRUITMENT_OF_NUMA_TO_MITOTIC_CENTROSOMES	
CKS2		REACTOME_METABOLISM_OF_NUCLEOTIDES	
H2AFV		KEGG_PYRIMIDINE_METABOLISM	
KPNA2		REACTOME_SUMOYLATION	
LRMP		REACTOME_POSITIVE_EPIGENETIC_REGULATION_OF_RRNA_EXPRESSION	
RRM2		REACTOME_TRANSCRIPTIONAL_REGULATION_BY_SMALL_RNAS	
ZWINT		WP_7Q1123_COPY_NUMBER_VARIATION_SYNDROME	
GAPDH		REACTOME_CELLULAR_SENESCENCE	
SNRPF		REACTOME_SENESCENCE_ASSOCIATED_SECRETORY_PHENOTYPE_SASP	
H2AZ2		REACTOME_TELOMERE_MAINTENANCE	
		HALLMARK_MITOTIC_SPINDLE	
		HALLMARK_GLYCOLYSIS	
		WP_MICROTUBULE_CYTOSKELETON_REGULATION	
		REACTOME_CONDENSATION_OF_PROMETAPHASE_CHROMOSOMES	
		WP_CELL_CYCLE	
		REACTOME_DNA_REPAIR	
		REACTOME_RECOGNITION_AND_ASSOCIATION_OF_DNA_GLYCOSYLASE_WITH_SITE_CONTAINING_AN_AFFECTED_PURINE	
		KEGG_CELL_CYCLE	
		WP_PATHOGENIC_ESCHERICHIA_COLI_INFECTION	
		REACTOME_APOPTOSIS_INDUCED_DNA_FRAGMENTATION	
		KEGG_PATHOGENIC_ESCHERICHIA_COLI_INFECTION	
		REACTOME_HOST_INTERACTIONS_OF_HIV_FACTORS	
		REACTOME_BASE_EXCISION_REPAIR_AP_SITE_FORMATION	
		WP_BIOMARKERS_FOR_PYRIMIDINE_METABOLISM_DISORDERS	
		REACTOME_GENE_SILENCING_BY_RNA	
		KEGG_SYSTEMIC_LUPUS_ERYTHEMATOSUS	
		REACTOME_DNA_METHYLATION	
		WP_G1_TO_S_CELL_CYCLE_CONTROL	
		REACTOME_ACTIVATED_PKN1_STIMULATES_TRANSCRIPTION_OF_AR_ANDROGEN_RECEPTOR_REGULATED_GENES_KLK2_AND_KLK3	
		REACTOME_INHIBITION_OF_DNA_RECOMBINATION_AT_TELOMERE	
		REACTOME_SIRT1_NEGATIVELY_REGULATES_RRNA_EXPRESSION	
		REACTOME_DNA_REPLICATION_PRE_INITIATION	
		REACTOME_EPIGENETIC_REGULATION_OF_GENE_EXPRESSION	
		WP_CORI_CYCLE	
		WP_PYRIMIDINE_METABOLISM_AND_RELATED_DISEASES	
		REACTOME_ASSEMBLY_OF_THE_ORC_COMPLEX_AT_THE_ORIGIN_OF_REPLICATION	
		REACTOME_ESTROGEN_DEPENDENT_GENE_EXPRESSION	
		HALLMARK_DNA_REPAIR	
		REACTOME_TP53_REGULATES_TRANSCRIPTION_OF_GENES_INVOLVED_IN_G2_CELL_CYCLE_ARREST	
		WP_PARKINUBIQUITIN_PROTEASOMAL_SYSTEM_PATHWAY	
		REACTOME_PRC2_METHYLATES_HISTONES_AND_DNA	
		REACTOME_AURKA_ACTIVATION_BY_TPX2	
		REACTOME_GLYCOLYSIS	
		REACTOME_TRANSCRIPTION_OF_E2F_TARGETS_UNDER_NEGATIVE_CONTROL_BY_DREAM_COMPLEX	
		WP_ALZHEIMERS_DISEASE	
		REACTOME_APC_C_CDH1_MEDIATED_DEGRADATION_OF_CDC20_AND_OTHER_APC_C_CDH1_TARGETED_PROTEINS_IN_LATE_MITOSIS_EARLY_G1	
		REACTOME_ERCC6_CSB_AND_EHMT2_G9A_POSITIVELY_REGULATE_RRNA_EXPRESSION	
		REACTOME_PHOSPHORYLATION_OF_THE_APC_C	
		REACTOME_S_PHASE	
		REACTOME_MEIOTIC_SYNAPSIS	
		REACTOME_RMTS_METHYLATE_HISTONE_ARGININES	
		REACTOME_TRANSPORT_OF_CONNEXONS_TO_THE_PLASMA_MEMBRANE	
		REACTOME_THE_ROLE_OF_GTSE1_IN_G2_M_PROGRESSION_AFTER_G2_CHECKPOINT	
		REACTOME_RECRUITMENT_OF_MITOTIC_CENTROSOME_PROTEINS_AND_COMPLEXES	
		REACTOME_POST_CHAPERONIN_TUBULIN_FOLDING_PATHWAY	
		REACTOME_MEIOTIC_RECOMBINATION	
		REACTOME_APC_C_CDC20_MEDIATED_DEGRADATION_OF_CYCLIN_B	
		REACTOME_REGULATION_OF_PLK1_ACTIVITY_AT_G2_M_TRANSITION	
		REACTOME_RNA_POLYMERASE_I_PROMOTER_ESCAPE	
		REACTOME_FORMATION_OF_THE_BETA_CATENIN_TCF_TRANSACTIVATING_COMPLEX	
		REACTOME_FORMATION_OF_TUBULIN_FOLDING_INTERMEDIATES_BY_CCT_TRIC	
		REACTOME_GLUCOSE_METABOLISM	
		REACTOME_RHO_GTPASES_ACTIVATE_PKNS	
		REACTOME_ACTIVATION_OF_NMDA_RECEPTORS_AND_POSTSYNAPTIC_EVENTS	
		REACTOME_RUNX1_REGULATES_GENES_INVOLVED_IN_MEGAKARYOCYTE_DIFFERENTIATION_AND_PLATELET_FUNCTION	
		REACTOME_ANCHORING_OF_THE_BASAL_BODY_TO_THE_PLASMA_MEMBRANE	
		REACTOME_ACTIVATION_OF_AMPK_DOWNSTREAM_OF_NMDARS	
		WP_GASTRIC_CANCER_NETWORK_1	
		HALLMARK_MTORC1_SIGNALING	
		REACTOME_MITOTIC_G2_G2_M_PHASES	
		REACTOME_CILIUM_ASSEMBLY	
		REACTOME_DISEASES_OF_PROGRAMMED_CELL_DEATH	
		WP_BASE_EXCISION_REPAIR	
		WP_ALZHEIMERS_DISEASE_AND_MIRNA_EFFECTS	
		REACTOME_DNA_STRAND_ELONGATION	
		REACTOME_RHO_GTPASES_ACTIVATE_IQGAPS	
		REACTOME_SEALING_OF_THE_NUCLEAR_ENVELOPE_NE_BY_ESCRT_III	
		REACTOME_TERMINATION_OF_TRANSLESION_DNA_SYNTHESIS	
		REACTOME_COOPERATION_OF_PREFOLDIN_AND_TRIC_CCT_IN_ACTIN_AND_TUBULIN_FOLDING	
		WP_GASTRIC_CANCER_NETWORK_2	
		REACTOME_NEGATIVE_EPIGENETIC_REGULATION_OF_RRNA_EXPRESSION	
		REACTOME_GLUCONEOGENESIS	
		REACTOME_RNA_POLYMERASE_I_TRANSCRIPTION	
		REACTOME_ESR_MEDIATED_SIGNALING	
		KEGG_BASE_EXCISION_REPAIR	
		KEGG_DNA_REPLICATION	
		REACTOME_INTERACTIONS_OF_REV_WITH_HOST_CELLULAR_PROTEINS	
		REACTOME_INTERACTIONS_OF_VPR_WITH_HOST_CELLULAR_PROTEINS	
		REACTOME_MEIOSIS	
		REACTOME_GAP_JUNCTION_ASSEMBLY	
		WP_FBXL10_ENHANCEMENT_OF_MAPERK_SIGNALING_IN_DIFFUSE_LARGE_BCELL_LYMPHOMA	
		REACTOME_PRE_NOTCH_EXPRESSION_AND_PROCESSING	
		REACTOME_ACTIVATION_OF_ANTERIOR_HOX_GENES_IN_HINDBRAIN_DEVELOPMENT_DURING_EARLY_EMBRYOGENESIS	
		REACTOME_SYNTHESIS_OF_DNA	
		REACTOME_TRANSLESION_SYNTHESIS_BY_Y_FAMILY_DNA_POLYMERASES_BYPASSES_LESIONS_ON_DNA_TEMPLATE	
		REACTOME_HIV_INFECTION	
		KEGG_PYRUVATE_METABOLISM	
		REACTOME_OXIDATIVE_STRESS_INDUCED_SENESCENCE	
		WP_DNA_REPLICATION	
		REACTOME_RUNX1_REGULATES_TRANSCRIPTION_OF_GENES_INVOLVED_IN_DIFFERENTIATION_OF_HSCS	
		REACTOME_AGGREPHAGY	
		REACTOME_ASSEMBLY_AND_CELL_SURFACE_PRESENTATION_OF_NMDA_RECEPTORS	
		REACTOME_CARBOXYTERMINAL_POST_TRANSLATIONAL_MODIFICATIONS_OF_TUBULIN	
		WP_ATM_SIGNALING_IN_DEVELOPMENT_AND_DISEASE	
		REACTOME_HCMV_EARLY_EVENTS	
		REACTOME_DNA_DAMAGE_BYPASS	
		REACTOME_RECYCLING_PATHWAY_OF_L1	
		REACTOME_TP53_REGULATES_TRANSCRIPTION_OF_CELL_CYCLE_GENES	
		REACTOME_REPRODUCTION	
		WP_HEPATITIS_C_AND_HEPATOCELLULAR_CARCINOMA	
		REACTOME_GAP_JUNCTION_TRAFFICKING_AND_REGULATION	
		REACTOME_APOPTOTIC_EXECUTION_PHASE	
		REACTOME_MRNA_SPLICING_MINOR_PATHWAY	
		REACTOME_COPI_INDEPENDENT_GOLGI_TO_ER_RETROGRADE_TRAFFIC	
		REACTOME_INTRAFLAGELLAR_TRANSPORT	
		REACTOME_SNRNP_ASSEMBLY	
		REACTOME_HIV_LIFE_CYCLE	
		WP_OREXIN_RECEPTOR_PATHWAY	
		REACTOME_HSP90_CHAPERONE_CYCLE_FOR_STEROID_HORMONE_RECEPTORS_SHR_IN_THE_PRESENCE_OF_LIGAND	
		REACTOME_HCMV_INFECTION	
		REACTOME_SIGNALING_BY_NUCLEAR_RECEPTORS	
		REACTOME_ORGANELLE_BIOGENESIS_AND_MAINTENANCE	
		REACTOME_KINESINS	
		REACTOME_DNA_DOUBLE_STRAND_BREAK_REPAIR	
		REACTOME_G2_M_CHECKPOINTS	

Stress	KG_annotation (ranked by pagerank scor	ORA_annotation	LLM_annotation
CD69	stress response	HALLMARK_TNFA_SIGNALING_VIA_NFKB	Immune Response and Lymphocyte Activation (0.92)
FOS	immune response	HALLMARK_INFLAMMATORY_RESPONSE	
JUN	cell survival	WP_CORTICOTROPINRELEASING_HORMONE_SIGNALING_PATHWAY	
CD83	systemic inflammatory response syndron	WP_IL18_SIGNALING_PATHWAY	
NR4A2	immune signaling	HALLMARK_IL2_STAT5_SIGNALING	
FOSB	in vivo cell proliferation	HALLMARK_HYPOXIA	
IGHD	cell proliferation	KEGG_B_CELL_RECEPTOR_SIGNALING_PATHWAY	
IGHM	myeloid differentiation	WP_NEUROINFLAMMATION	
DUSP1	tumor microenvironments	HALLMARK_UV_RESPONSE_UP	
FCER2	tumor evolution	WP_QUERCETIN_AND_NFKB_AP1_INDUCED_APOPTOSIS	
FOXP1	macrophage differentiation	WP_TGFBETA_SIGNALING_PATHWAY	
TCL1A	basal cells	WP_GLUCOCORTICOID_RECEPTOR_PATHWAY	
ZNF331	human lung epithelial development	WP_OREXIN_RECEPTOR_PATHWAY	
ZFP36	cytokine release syndrome	WP_HOSTPATHOGEN_INTERACTION_OF_HUMAN_CORONAVIRUSES_INTERFERON_INDUCTION	
GPR183	cancer cell motility	HALLMARK_APOPTOSIS	
CD55	neuromyelitis optica	KEGG_HEMATOPOIETIC_CELL_LINEAGE	
FCMR	giant cell arteritis	WP_HOSTPATHOGEN_INTERACTION_OF_HUMAN_CORONAVIRUSES_MAPK_SIGNALING	
JUNB	immune microenvironment changes	WP_APOPTOSIS_MODULATION_AND_SIGNALING	
RGS2	cell turnover	WP_TCELL_RECEPTOR_TCR_SIGNALING_PATHWAY	
YPEL5	tumor suppression	KEGG_MAPK_SIGNALING_PATHWAY	
CCR7	peripheral angiogenesis	REACTOME_NGF_STIMULATED_TRANSCRIPTION	
LINC00926	intrinsic tumor-suppressive mechanisms	REACTOME_MYD88_INDEPENDENT_TLR4_CASCADE	
RHOH		WP_PDGF_PATHWAY	
IER2		WP_TNFRELATED_WEAK_INDUCER_OF_APOPTOSIS_TWEAK_SIGNALING_PATHWAY	
SELL		REACTOME_ACTIVATION_OF_THE_AP_1_FAMILY_OF_TRANSCRIPTION_FACTORS	
SLC2A3		REACTOME_INTERLEUKIN_4_AND_INTERLEUKIN_13_SIGNALING	
BIRC3		HALLMARK_ESTROGEN_RESPONSE_LATE	
BTG2		WP_GASTRIN_SIGNALING_PATHWAY	
LY9		WP_IL3_SIGNALING_PATHWAY	
IL4R		WP_PHOTODYNAMIC_THERAPYINDUCED_AP1_SURVIVAL_SIGNALING	
ZFP36L2		WP_SPINAL_CORD_INJURY	
CLEC2D		WP_NUCLEAR_RECEPTORS_METAPATHWAY	
HVCN1		WP_IL4_SIGNALING_PATHWAY	
PPP1R15A		WP_RANKLRANK_SIGNALING_PATHWAY	
SNX2		WP_PREIMPLANTATION_EMBRYO	
AREG		REACTOME_CD22_MEDIATED_BCR_REGULATION	
DUSP2		REACTOME_NUCLEAR_EVENTS_KINASE_AND_TRANSCRIPTION_FACTOR_ACTIVATION	
GADD45B		WP_HEMATOPOIETIC_STEM_CELL_DIFFERENTIATION	
KLF2		WP_TCELL_ANTIGEN_RECEPTOR_TCR_PATHWAY_DURING_STAPHYLOCOCCUS_AUREUS_INFECTION	
KLF6		WP_MAPK_PATHWAY_IN_CONGENITAL_THYROID_CANCER	
ADAM28		WP_ONCOSTATIN_M_SIGNALING_PATHWAY	
CD72		WP_MAPK_SIGNALING_PATHWAY	
NFKBIA		KEGG_LEISHMANIA_INFECTION	
VPREB3		REACTOME_TOLL_LIKE_RECEPTOR_CASCADES	
CD22		WP_CHROMOSOMAL_AND_MICROSATELLITE_INSTABILITY_IN_COLORECTAL_CANCER	
CREM		WP_PROLACTIN_SIGNALING_PATHWAY	
NR4A1		REACTOME_SIGNALING_BY_THE_B_CELL_RECEPTOR_BCR	
PHACTR1		WP_VITAMIN_D_IN_INFLAMMATORY_DISEASES	
TSPAN13		REACTOME_RAF_INDEPENDENT_MAPK1_3_ACTIVATION	
VPS37B		WP_ESTROGEN_SIGNALING_PATHWAY	
		WP_PHOTODYNAMIC_THERAPYINDUCED_NFE2L2_NRF2_SURVIVAL_SIGNALING	
		REACTOME_ESTROGEN_DEPENDENT_NUCLEAR_EVENTS_DOWNSTREAM_OF_ESR_MEMBRANE_SIGNALING	
		REACTOME_ANTIGEN_ACTIVATES_B_CELL_RECEPTOR_BCR_LEADING_TO_GENERATION_OF_SECOND_MESSENGERS	
		WP_APOPTOSIS	
		WP_TCELL_ACTIVATION_SARSCOV2	
		WP_PHYSIOLOGICAL_AND_PATHOLOGICAL_HYPERTROPHY_OF_THE_HEART	
		WP_HAIR_FOLLICLE_DEVELOPMENT_CYTODIFFERENTIATION_PART_3_OF_3	
		REACTOME_TOLL_LIKE_RECEPTOR_9_TLR9_CASCADE	
		WP_TNFALPHA_SIGNALING_PATHWAY	
		WP_PDGFRBETA_PATHWAY	
		WP_SELECTIVE_EXPRESSION_OF_CHEMOKINE_RECEPTORS_DURING_TCELL_POLARIZATION	
		WP_B_CELL_RECEPTOR_SIGNALING_PATHWAY	
		WP_SMALL_CELL_LUNG_CANCER	
		WP_COMPLEMENT_SYSTEM	
		REACTOME_SIGNALING_BY_INTERLEUKINS	
		HALLMARK_P53_PATHWAY	
		REACTOME_MAPK_TARGETS_NUCLEAR_EVENTS_MEDIATED_BY_MAP_KINASES	
		KEGG_TOLL_LIKE_RECEPTOR_SIGNALING_PATHWAY	
		REACTOME_TOLL_LIKE_RECEPTOR_TLR1_TLR2_CASCADE	
		WP_TOLLLIKE_RECEPTOR_SIGNALING_PATHWAY	
		KEGG_T_CELL_RECEPTOR_SIGNALING_PATHWAY	
		WP_HEPATOCYTE_GROWTH_FACTOR_RECEPTOR_SIGNALING	
		WP_OXIDATIVE_STRESS_RESPONSE	
		WP_SIGNAL_TRANSDUCTION_THROUGH_IL1R	
		REACTOME_ESR_MEDIATED_SIGNALING	
		WP_NETWORK_MAP_OF_SARSCOV2_SIGNALING_PATHWAY	
		WP_NUCLEAR_RECEPTORS	
		WP_ATM_SIGNALING_PATHWAY	
		WP_IL5_SIGNALING_PATHWAY	
		WP_NEURAL_CREST_CELL_MIGRATION_DURING_DEVELOPMENT	
		WP_IL2_SIGNALING_PATHWAY	
		REACTOME_NEGATIVE_REGULATION_OF_MAPK_PATHWAY	
		WP_NEURAL_CREST_CELL_MIGRATION_IN_CANCER	
		REACTOME_SIGNALING_BY_NTRKS	

Memory	KG_annotation (ranked by pagerank scor	ORA_annotation	LLM_annotation
CRIP1	cell adhesion	HALLMARK_ALLOGRAFT_REJECTION	Immune Response and Cell Adhesion (0.85)
TNFRSF13B	autoantibody production	HALLMARK_EPITHELIAL_MESENCHYMAL_TRANSITION	
CAPG	favourable patient prognosis	HALLMARK_P53_PATHWAY	
ITGB1	epithelial ovarian cancer	WP_PROSTAGLANDIN_SYNTHESIS_AND_REGULATION	
CD99	immune effector mechanisms	REACTOME_DISSOLUTION_OF_FIBRIN_CLOT	
S100A4	virus elimination	WP_TYROBP_CAUSAL_NETWORK_IN_MICROGLIA	
ACP5	allergic sensitization		
CD82	persistent respiratory immune alterations		
CLECL1	cytotoxic immune responses		
HCST	long-term pulmonary sequelae		
SCIMP			
ANXA2			
COTL1			
CTSH			
VIM			
CD27			
LGALS1			
PLP2			
CCDC50			
FCRL5			
NEAT1			
S100A11			
S100A10			
UBE2N			
AIM2			
EMP3			
S100A6			
ARPC1B			
LSP1			
PTPN1			
RGS1			
EVI2B			
GRN			
IFI30			
RGCC			
SRGN			
SUB1			
CAPZB			
ENTPD1			
GPR183			
IGHA1			
CD86			
GSTK1			
HOPX			
IGFLR1			
PLEK			
BLK			
BHLHE41			
ISCU			
CXCR3			

Metabolism_MY(	KG_annotation (ranked by pagerank scor	ORA_annotation	LLM_annotation
PSME2	immune response	HALLMARK_MYC_TARGETS_V1	Glycolysis and Protein Synthesis Regulation (0.85)
ENO1	systemic inflammatory response syndron	HALLMARK_MTORC1_SIGNALING	
LDHA	immune signaling	WP_GLYCOLYSIS_AND_GLUCONEOGENESIS	
NME1	possible ongoing antigenic stimulation	WP_AEROBIC_GLYCOLYSIS	
RANBP1	antitumor response	KEGG_GLYCOLYSIS_GLUCONEOGENESIS	
TUBB	virus elimination	REACTOME_NEUTROPHIL_DEGRANULATION	
C1QBP	immune-related gene signatures	HALLMARK_GLYCOLYSIS	
EIF5A		REACTOME_HOST_INTERACTIONS_OF_HIV_FACTORS	
NHP2		WP_GLYCOLYSIS_IN_SENESCENCE	
PKM		REACTOME_THE_ROLE_OF_GTSE1_IN_G2_M_PROGRESSION_AFTER_G2_CHECKPOINT	
PRDX1		WP_CLEAR_CELL_RENAL_CELL_CARCINOMA_PATHWAYS	
RAN		WP_METABOLIC_REPROGRAMMING_IN_COLON_CANCER	
FABP5		REACTOME_METABOLISM_OF_NUCLEOTIDES	
SNRPD1		WP_CORI_CYCLE	
VDAC1		HALLMARK_E2F_TARGETS	
HSPD1		REACTOME_MITOTIC_G2_G2_M_PHASES	
PPA1		REACTOME_FORMATION_OF_TUBULIN_FOLDING_INTERMEDIATES_BY_CCT_TRIC	
SNRPE		WP_MRNA_PROCESSING	
PA2G4		REACTOME_GLYCOLYSIS	
RPL22L1		REACTOME_INTERCONVERSION_OF_NUCLEOTIDE_DI_AND_TRIPHOSPHATES	
SNRPF		REACTOME_HIV_INFECTION	
TUBA1B		REACTOME_PYRUVATE_METABOLISM	
CCT3		REACTOME_COOPERATION_OF_PREFOLDIN_AND_TRIC_CCT_IN_ACTIN_AND_TUBULIN_FOLDING	
DDX21		REACTOME_GLUCONEOGENESIS	
EIF4A1		KEGG_PURINE_METABOLISM	
HMGA1		REACTOME_CELLULAR_RESPONSE_TO_CHEMICAL_STRESS	
IMPDH2		REACTOME_GLUCOSE_METABOLISM	
LDHB		KEGG_PYRUVATE_METABOLISM	
PRMT1		REACTOME_ANTIGEN_PROCESSING_CROSS_PRESENTATION	
PSMB3		REACTOME_FOLDING_OF_ACTIN_BY_CCT_TRIC	
CALR		KEGG_PROTEASOME	
HSP90AB1		REACTOME_SLBP_DEPENDENT_PROCESSING_OF_REPLICATION_DEPENDENT_HISTONE_PRE_MRNAS	
HSPE1		REACTOME_HEDGEHOG_OFF_STATE	
NME2		HALLMARK_OXIDATIVE_PHOSPHORYLATION	
PGAM1		REACTOME_CROSS_PRESENTATION_OF_SOLUBLE_EXOGENOUS_ANTIGENS_ENDOSOMES	
TPI1		REACTOME_MRNA_SPLICING_MINOR_PATHWAY	
TXN		REACTOME_NEGATIVE_REGULATION_OF_NOTCH4_SIGNALING	
ABRACL		REACTOME_SNRNP_ASSEMBLY	
ATP5G1		REACTOME_AUF1_HNRNP_D0_BINDS_AND_DESTABILIZES_MRNA	
NDUFAB1		REACTOME_DEGRADATION_OF_AXIN	
PSMA4		REACTOME_PYRUVATE_METABOLISM_AND_CITRIC_ACID_TCA_CYCLE	
SYNGR2		REACTOME_REGULATION_OF_RUNX3_EXPRESSION_AND_ACTIVITY	
TIMM13		REACTOME_DEGRADATION_OF_DVL	
APRT		REACTOME_STABILIZATION_OF_P53	
CCT6A		HALLMARK_MYC_TARGETS_V2	
CD83		REACTOME_CDT1_ASSOCIATION_WITH_THE_CDC6_ORC_ORIGIN_COMPLEX	
ERH		REACTOME_METABOLISM_OF_POLYAMINES	
MIF		REACTOME_DEGRADATION_OF_GLI1_BY_THE_PROTEASOME	
PRELID1		REACTOME_SCF_SKP2_MEDIATED_DEGRADATION_OF_P27_P21	
HNRNPAB		REACTOME_DEFECTIVE_CFTR_CAUSES_CYSTIC_FIBROSIS	
		REACTOME_DECTIN_1_MEDIATED_NONCANONICAL_NF_KB_SIGNALING	
		REACTOME_THE_NLRP3_INFLAMMASOME	
		REACTOME_MITOTIC_METAPHASE_AND_ANAPHASE	
		REACTOME_ASYMMETRIC_LOCALIZATION_OF_PCP_PROTEINS	
		WP_PROTEASOME_DEGRADATION	
		REACTOME_HEDGEHOG_LIGAND_BIOGENESIS	
		REACTOME_MITOCHONDRIAL_PROTEIN_IMPORT	
		REACTOME_REGULATION_OF_HMOX1_EXPRESSION_AND_ACTIVITY	
		REACTOME_G1_S_DNA_DAMAGE_CHECKPOINTS	
		REACTOME_REGULATION_OF_RAS_BY_GAPS	
		REACTOME_REGULATION_OF_PTEN_STABILITY_AND_ACTIVITY	
		REACTOME_SIGNALING_BY_HEDGEHOG	
		REACTOME_ORC1_REMOVAL_FROM_CHROMATIN	
		REACTOME_REGULATION_OF_RUNX2_EXPRESSION_AND_ACTIVITY	
		REACTOME_SARS_COV_INFECTIONS	
		REACTOME_APC_C_CDH1_MEDIATED_DEGRADATION_OF_CDC20_AND_OTHER_APC_C_CDH1_TARGETED_PROTEINS_IN_LATE_MITOSIS_EARLY_G1	
		REACTOME_CELLULAR_RESPONSE_TO_HYPOXIA	
		WP_ALZHEIMERS_DISEASE	
		REACTOME_INFLAMMASOMES	
		REACTOME_ABC_TRANSPORTER_DISORDERS	
		REACTOME_DOWNSTREAM_SIGNALING_EVENTS_OF_B_CELL_RECEPTOR_BCR	
		REACTOME_RHOBTB2_GTPASE_CYCLE	
		REACTOME_SIGNALING_BY_NOTCH4	
		REACTOME_REGULATION_OF_EXPRESSION_OF_SLITS_AND_ROBOS	
		REACTOME_M_PHASE	
		REACTOME_CYCLIN_A_CDK2_ASSOCIATED_EVENTS_AT_S_PHASE_ENTRY	
		REACTOME_DEGRADATION_OF_BETA_CATENIN_BY_THE_DESTRUCTION_COMPLEX	
		REACTOME_HEDGEHOG_ON_STATE	
		REACTOME_DEFECTIVE_INTRINSIC_PATHWAY_FOR_APOPTOSIS	
		REACTOME_POTENTIAL_THERAPEUTICS_FOR_SARS	
		REACTOME_REGULATION_OF_MRNA_STABILITY_BY_PROTEINS_THAT_BIND_AU_RICH_ELEMENTS	
		REACTOME_THE_CITRIC_ACID_TCA_CYCLE_AND_RESPIRATORY_ELECTRON_TRANSPORT	
		REACTOME_APOPTOSIS	
		KEGG_ANTIGEN_PROCESSING_AND_PRESENTATION	
		REACTOME_APC_C_MEDIATED_DEGRADATION_OF_CELL_CYCLE_PROTEINS	
		REACTOME_PURINERGIC_SIGNALING_IN_LEISHMANIASIS_INFECTION	
		REACTOME_SWITCHING_OF_ORIGINS_TO_A_POST_REPLICATIVE_STATE	
		WP_ANDROGEN_RECEPTOR_SIGNALING_PATHWAY	
		REACTOME_PCP_CE_PATHWAY	
		REACTOME_MAPK6_MAPK4_SIGNALING	
		REACTOME_PROCESSING_OF_CAPPED_INTRONLESS_PRE_MRNA	
		REACTOME_SEPARATION_OF_SISTER_CHROMATIDS	
		REACTOME_TRANSCRIPTIONAL_REGULATION_BY_RUNX3	
		REACTOME_PROTEIN_FOLDING	
		WP_COMPLEMENT_SYSTEM	
		REACTOME_CLEC7A_DECTIN_1_SIGNALING	
		REACTOME_UCH_PROTEINASES	
		HALLMARK_HYPOXIA	
		REACTOME_TNFR2_NON_CANONICAL_NF_KB_PATHWAY	
		REACTOME_INTERLEUKIN_1_SIGNALING	
		REACTOME_ABC_FAMILY_PROTEINS_MEDIATED_TRANSPORT	
		KEGG_PROPANOATE_METABOLISM	
		REACTOME_PROGRAMMED_CELL_DEATH	
		KEGG_CYSTEINE_AND_METHIONINE_METABOLISM	
		WP_ALZHEIMERS_DISEASE_AND_MIRNA_EFFECTS	
		REACTOME_RHOBTB_GTPASE_CYCLE	
		REACTOME_SIGNALING_BY_ROBO_RECEPTORS	
		HALLMARK_UNFOLDED_PROTEIN_RESPONSE	
		REACTOME_UB_SPECIFIC_PROCESSING_PROTEASES	
		REACTOME_DETOXIFICATION_OF_REACTIVE_OXYGEN_SPECIES	
		REACTOME_INTERACTIONS_OF_REV_WITH_HOST_CELLULAR_PROTEINS	
		WP_PHOTODYNAMIC_THERAPYINDUCED_HIF1_SURVIVAL_SIGNALING	
		REACTOME_COOPERATION_OF_PDCL_PHLP1_AND_TRIC_CCT_IN_G_PROTEIN_BETA_FOLDING	
		REACTOME_GENE_AND_PROTEIN_EXPRESSION_BY_JAK_STAT_SIGNALING_AFTER_INTERLEUKIN_12_STIMULATION	
		REACTOME_ASSOCIATION_OF_TRIC_CCT_WITH_TARGET_PROTEINS_DURING_BIOSYNTHESIS	
		REACTOME_SYNTHESIS_OF_DNA	
		REACTOME_TRANSCRIPTIONAL_REGULATION_BY_RUNX2	
		REACTOME_CYTOPROTECTION_BY_HMOX1	
		REACTOME_TCR_SIGNALING	
		REACTOME_TRANSCRIPTIONAL_REGULATION_BY_RUNX1	
		REACTOME_METABOLISM_OF_AMINO_ACIDS_AND_DERIVATIVES	
		KEGG_SPLICEOSOME	
		REACTOME_RUNX1_REGULATES_TRANSCRIPTION_OF_GENES_INVOLVED_IN_DIFFERENTIATION_OF_HSCS	
		REACTOME_INTERLEUKIN_12_SIGNALING	
		REACTOME_FCERI_MEDIATED_NF_KB_ACTIVATION	
		HALLMARK_REACTIVE_OXYGEN_SPECIES_PATHWAY	
		REACTOME_PTEN_REGULATION	
		REACTOME_C_TYPE_LECTIN_RECEPTORS_CLRS	
		REACTOME_INTERLEUKIN_1_FAMILY_SIGNALING	
		WP_NRF2_PATHWAY	
		REACTOME_BETA_CATENIN_INDEPENDENT_WNT_SIGNALING	
		REACTOME_DNA_REPLICATION_PRE_INITIATION	
		WP_TRANSLATION_FACTORS	
		REACTOME_MITOTIC_G1_PHASE_AND_G1_S_TRANSITION	
		HALLMARK_DNA_REPAIR	
		WP_INTERFERON_TYPE_I_SIGNALING_PATHWAYS	
		WP_PATHOGENIC_ESCHERICHIA_COLI_INFECTION	
		REACTOME_AUTOPHAGY	
		REACTOME_HIV_LIFE_CYCLE	
		KEGG_PATHOGENIC_ESCHERICHIA_COLI_INFECTION	
		REACTOME_NUCLEOTIDE_BINDING_DOMAIN_LEUCINE_RICH_REPEAT_CONTAINING_RECEPTOR_NLR_SIGNALING_PATHWAYS	

Germinal_Center	ICKG_annotation (ranked by pagerank score)	ORA_annotation	LLM_annotation
LRMP	eukaryotic cell migration		Lymphocyte Activation and Signaling (0.85)
NEIL1	antigen presentation		
TCL1A	immune checkpoint upregulation		
MARCKSL1	complete remission		
RGS13	median duration		
RFTN1	tissue-specific splicing		
BASP1	tumor regression		
HMCES	tumor immunopathology		
BCL7A	antibody-secreting cells		
LCP1	multiple immune checkpoints		
LPP	developing combination immunotherapeutic strategies		
UBE2J1	hematopoietic cellular products		
PRDX6	standard allogeneic grafts		
SYNE2	stem cells		
CD22	multiple subset-dependent entry mechanisms		
FAM3C	viral glycoproteins		
HOPX	cytokine secretion		
CCDC144A	anti-viral vaccines		
KLHL6	antibody-coated cell phagocytosis		
PARP1	lymphocyte activation		
PRPSAP2	relapse prevention		
ABRACL	transient peripheral transcriptomic alterations		
CD40	tertiary lymphoid structure-like lymphocytic aggregates		
CD79B			
DBI			
GCHFR			
LAT2			
LMO2			
POU2AF1			
RGS1			
TMEM123			
BCAS4			
DAAM1			
GGA2			
HMGA1			
HMGN1			
LCK			
MTF2			
P2RX5			
RNGTT			
STAG3			
UCP2			
JCHAIN			
SERPINA9			
CCDC88A			
SUSD3			
GRHPR			
ACTG1			
PPP2CA			
CPNE3			

Interferon	CKG_annotation (ranked by pagerank score	ORA_annotation	LLM_annotation
MX1	interferon secretion	HALLMARK_INTERFERON_GAMMA_RESPONSE	Antiviral Defense and Interferon Response (0.92)
PLSCR1	virus reactivation	HALLMARK_INTERFERON_ALPHA_RESPONSE	
SAMD9L	autoimmune hepatitis	REACTOME_INTERFERON_SIGNALING	
GBP1	immunological disease indications	REACTOME_INTERFERON_ALPHA_BETA_SIGNALING	
GBP4	cancer occurrence	WP_IMMUNE_RESPONSE_TO_TUBERCULOSIS	
IFI6	therapeutic antibody discovery	REACTOME_ANTIVIRAL_MECHANISM_BY_IFN_STIMULATED_GENES	
IFIT2	cancer heterogeneity	WP_TYPE_II_INTERFERON_SIGNALING_IFNG	
IFIT3	non-invasive serum/plasma immunobiomar	REACTOME_INTERFERON_GAMMA_SIGNALING	
ISG15	tumor particulate antigens	WP_TYPE_I_INTERFERON_INDUCTION_AND_SIGNALING_DURING_SARSCOV2_INFECTION	
MX2	long-term protective humoral responses	WP_NETWORK_MAP_OF_SARSCOV2_SIGNALING_PATHWAY	
OAS1	cancer genesis	WP_HOSTPATHOGEN_INTERACTION_OF_HUMAN_CORONAVIRUSES_INTERFERON_INDUCTION	
PARP9	extracellular matrix remodeling	WP_MEASLES_VIRUS_INFECTION	
TNFSF10	stromal cell heterogeneity	WP_SARSCOV2_INNATE_IMMUNITY_EVASION_AND_CELLSPECIFIC_IMMUNE_RESPONSE	
CMPK2	transcription factors	WP_NONGENOMIC_ACTIONS_OF_125_DIHYDROXYVITAMIN_D3	
IFI35	cancer immunology	HALLMARK_INFLAMMATORY_RESPONSE	
IFIT1	memory cell differentiation	REACTOME_REGULATION_OF_IFNA_SIGNALING	
IRF7	systemic autoimmune diseases	REACTOME_DDX58_IFIH1_MEDIATED_INDUCTION_OF_INTERFERON_ALPHA_BETA	
PARP14	breast cancer cell growth	REACTOME_NEGATIVE_REGULATORS_OF_DDX58_IFIH1_SIGNALING	
SAMD9	resistant tumor variants	REACTOME_MATURATION_OF_SARS_COV_1_NUCLEOPROTEIN	
STAT1	immune cell-based immunotherapy	HALLMARK_IL2_STAT5_SIGNALING	
TRIM22	immune-excluded squamous cell carcinomas	REACTOME_REGULATION_OF_IFNG_SIGNALING	
UBE2L6	cell-based therapeutics	WP_EBOLA_VIRUS_INFECTION_IN_HOST	
USP18	colorectal carcinoma	REACTOME_MATURATION_OF_SARS_COV_2_NUCLEOPROTEIN	
XAF1	liver cancer tissues	REACTOME_NICOTINAMIDE_SALVAGING	
APOL6	peripheral immune macroenvironment	REACTOME_GROWTH_HORMONE_RECEPTOR_SIGNALING	
BST2	alternative polyadenylation	REACTOME_INACTIVATION_OF_CSF3_G_CSF_SIGNALING	
HERC5	acidic cancer microenvironment	WP_EPO_RECEPTOR_SIGNALING	
IFI44	tumor-derived extracellular vesicles	REACTOME_TRANSLATION_OF_SARS_COV_1_STRUCTURAL_PROTEINS	
IFI44L	radiation-induced lymphopenia	WP_PDGFRBETA_PATHWAY	
NT5C3A	cancer-associated immune dysfunction	REACTOME_SIGNALING_BY_CSF3_G_CSF	
OAS3	metabolic hyperactivation	REACTOME_NICOTINATE_METABOLISM	
RABGAP1L	cancer immune evasion mechanisms	HALLMARK_ALLOGRAFT_REJECTION	
RNF213	unwanted immune reactions	REACTOME_TERMINATION_OF_TRANSLESION_DNA_SYNTHESIS	
RSAD2	lymphocyte infiltration		
CHST12	excessive complement activation		
EIF2AK2	adaptive immune functions		
LY6E	immunological correlation		
MNDA	cancer-related immune disorder		
NMI	glioma progression		
IFITM1	long-lasting inflammatory conditions		
ISG20	immune dynamics		
LAP3	modest expansion		
MT2A	anti-tumor immune responses		
OAS2	type-2 immune responses		
PPM1K	oxidative phosphorylation		
SLFN5	metabolic regulation		
SOCS1			
EPSTI1			
TAP1			
DYNLT1			

Progenitor	ICKG_annotation (ranked by pagerank score)	ORA_annotation	LLM_annotation
SOX4	complete remission	KEGG_HEMATOPOIETIC_CELL_LINE	B-cell Development and Differentiation (0.85)
VPREB1	median duration		
CD9	early plasmablasts differentiation		
IGLL1	humoral immune responses		
CD24	long-term immune memory		
MZB1	tumor immunopathology		
SNHG7	multiple immune checkpoints		
STMN1	tumor infiltrating immune cells		
NEIL1	aggrephagy-related gene expression		
VPREB3	aggrephagy-related genes		
CD38	tumor microenvironment support		
PCDH9	immune checkpoint blockade immunotherapeutic response		
CCDC191	tumor immunosurveillance		
CMTM7	lymphocyte activation		
DNTT	tumorigenic responses		
GAPDH			
H1FX			
MME			
NSMCE1			
SSBP2			
TCL1A			
TOP2B			
YBX3			
ARPP21			
BACH2			
BCL7A			
CDC25B			
EBF1			
GLRX			
HMGB2			
MLXIP			
MT1X			
NIBAN3			
RCSD1			
S1PR4			
TKT			
VDAC1			
LDLRAD4			
CCDC112			
CD99			
RGS2			
ITM2C			
TRAM1			
IGLL5			
UBE2J1			
PTPRE			
CD72			
TP53INP1			
TCF4			
TMEM243			

B-cells1	ICKG_annotation (ranked by pagerank score)	ORA_annotation	LLM_annotation
NKTR	enhanced immune cell recruitment	WP_HISTONE_MODIFICATIC	Chromatin Remodeling and Transcriptional Regulation (0.85)
ARGLU1	clinical remission		
ATM	severe neurotoxicity		
RBM6	co-receptor function		
ARID1B	ectopic accumulation		
ATRX	cytokine release syndrome		
BDP1	neuromyelitis optica		
BPTF	giant cell arteritis		
MDM4	hematopoietic cellular products		
NEAT1	tertiary lymphoid structure-like lymphocytic aggregates		
PARP14	standard allogeneic grafts		
SEL1L3	immunoglobulin deficiency		
SYNE2	stem cells		
ZNF292	successful solid tumor cell therapy		
ASH1L	successful cellular therapy		
ATF7IP	tumour-promoting mediators		
BIRC3	multiple subset-dependent entry mechanisms		
CD22	viral glycoproteins		
CLEC2D	antigen recognition		
CTNNB1	anti-viral vaccines		
FTX	antibody-coated cell phagocytosis		
HNRNPH1	relapse prevention		
ITSN2	poor tumour differentiation		
KMT2A	transient peripheral transcriptomic alterations		
LNPEP			
MBNL1			
PCSK7			
PSMA3-AS1			
RSRP1			
SFPQ			
STX7			
TNRC6B			
XIST			
TTN			
C1ORF56			
RASGEF1B			
ADAM19			
B4GALT1			
FAM214A			
PRDM2			
PPP3CA			
GOLGB1			
CDC42SE1			
TUT4			
IKZF3			
CDK13			
CCNL1			
OGA			
SET			
ANKRD11			

Respiration	ICKG_annotation (ranked by pagerank score)	ORA_annotation	LLM_annotation
PPIB	cancer progression	KEGG_HUNTINGTONS_DISEASE	Protein Folding and Cellular Energy Production (0.85)
H2AFZ	metastasis formation	KEGG_PARKINSONS_DISEASE	
JCHAIN	tumor biology	KEGG_OXIDATIVE_PHOSPHORYLATION	
ATP5G3	tumor progression	REACTOME_RESPIRATORY_ELECTRON_TRANSPORT	
COX7B	tumor growth	WP_NONALCOHOLIC_FATTY_LIVER_DISEASE	
HMGB1	immune evasion	WP_ELECTRON_TRANSPORT_CHAIN_OXPHOS_SYSTEM_IN_MITOCHONDRIA	
RAN	tumor immunology	KEGG_ALZHEIMERS_DISEASE	
CYCS	immunogenic cell death	REACTOME_RESPIRATORY_ELECTRON_TRANSPORT_ATP_SYNTHESIS_BY_CHEMIOSMOTIC_COUPLING_AND_HEAT_PRODUCTION_BY_UNCOUPLING_PROTEINS	
HSPE1	subsequent inadequate antigen presentation	REACTOME_TP53_REGULATES_METABOLIC_GENES	
LGALS1	cell cycle progression	REACTOME_CELLULAR_RESPONSE_TO_CHEMICAL_STRESS	
PDIA6	autophagy-dependent mechanisms	REACTOME_THE_CITRIC_ACID_TCA_CYCLE_AND_RESPIRATORY_ELECTRON_TRANSPORT	
PPIA	locally advanced adenocarcinoma	HALLMARK_MYC_TARGETS_V1	
SNRPD2	designated minor histocompatibility antigens	HALLMARK_OXIDATIVE_PHOSPHORYLATION	
SNRPG	cancer cell motility	KEGG_CARDIAC_MUSCLE_CONTRACTION	
ATP5I	tumor-associated tertiary lymphoid structures	WP_MITOCHONDRIAL_COMPLEX_IV_ASSEMBLY	
ATP5J2	vivo cell proliferation	REACTOME_TRANSCRIPTIONAL_REGULATION_BY_TP53	
CHCHD2	tumor escape	REACTOME_CYTOPROTECTION_BY_HMOX1	
COX6C	immunosuppressive tumor microenvironment	WP_VEGFAVEGFR2_SIGNALING_PATHWAY	
DBI	epithelial-mesenchymal transition	REACTOME_INTERCONVERSION_OF_NUCLEOTIDE_DI_AND_TRIPHOSPHATES	
GLRX	precancerous lesions	REACTOME_DETOXIFICATION_OF_REACTIVE_OXYGEN_SPECIES	
IGHA1	immune checkpoint ligands	REACTOME_GENE_AND_PROTEIN_EXPRESSION_BY_JAK_STAT_SIGNALING_AFTER_INTERLEUKIN_12_STIMULATION	
LSM3	cancer prognosis	HALLMARK_MTORC1_SIGNALING	
NDUFS5	immune detection	REACTOME_SRP_DEPENDENT_COTRANSLATIONAL_PROTEIN_TARGETING_TO_MEMBRANE	
NOP10	neo-adjuvant chemotherapy	REACTOME_INTERLEUKIN_12_SIGNALING	
NPM1	cancer treatment	HALLMARK_REACTIVE_OXYGEN_SPECIES_PATHWAY	
POLR2L	modern rectal cancer treatment	REACTOME_MRNA_SPLICING_MINOR_PATHWAY	
PRDX1	organ-preserving strategies	REACTOME_HOST_INTERACTIONS_OF_HIV_FACTORS	
PSME2	neo-adjuvant radiotherapy	REACTOME_INTERLEUKIN_12_FAMILY_SIGNALING	
SEC61B	cancer diagnosis	REACTOME_HIV_INFECTION	
SSR3	antitumor immune response	REACTOME_TRANSLATION	
COX6B1	cardiovascular disease	REACTOME_MRNA_SPLICING	
POMP	gastric carcinogenesis	REACTOME_MICRORNA_MIRNA_BIOGENESIS	
ATP5O	early gastric cancer	REACTOME_PYROPTOSIS	
COX7A2	immunological disease indications	REACTOME_METABOLISM_OF_NUCLEOTIDES	
PTMA	tumor microenvironments	REACTOME_ANTIGEN_PROCESSING_CROSS_PRESENTATION	
CLIC1	tumor-directed immune responses	REACTOME_NUCLEAR_IMPORT_OF_REV_PROTEIN	
BTF3	immune-based approaches		
SSR2	cancer occurrence		
ANXA2	therapeutic antibody discovery		
TMSB10	intratumoral tertiary lymphoid structures		
PPA1	cancer heterogeneity		
TMA7	tumor-associated antigens		
NME1	non-invasive serum/plasma immunobiomarkers		
TXN	tumor-infiltrating lymphocytes		
ATP5J	follicular lymphoma		
UQCRH	neutrophil extracellular traps		
NDUFB3	cellular senescence		
SUB1	cancer risk		
RPS7	inhibitory immune checkpoints		
HN1	chronic low-grade inflammation		
	successful solid tumor cell therapy		
	successful cellular therapy		
	leucine-rich repeat-containing protein		
	tumor particulate antigens		
	myocardial infarction		
	inflammatory-related genes		
	extracellular matrix remodeling		
	cell-based cancer immunotherapy		
	tumor immunosuppression		
	substantial tissue specificity		
	stromal cell heterogeneity		
	gene therapy		
	immune-regulatory networks		
	immune challenges		
	proinflammatory cytokine production		
	degranulation		
	immune cell fate		
	tumour progression		
	chemotherapy-induced cachexia		
	transcription factors		
	cancer immunology		
	cancer genesis		
	inflammasome receptor nucleotide-binding oligomerization domain		
	breast cancer cell growth		
	resistant tumor variants		
	immune cell-based immunotherapy		
	immune-excluded squamous cell carcinomas		
	cell-based therapeutics		
	colorectal carcinoma		
	liver cancer tissues		
	peripheral immune macroenvironment		
	alternative polyadenylation		
	acidic cancer microenvironment		
	tumor-derived extracellular vesicles		
	radiation-induced lymphopenia		
	cancer-associated immune dysfunction		
	metabolic hyperactivation		
	cancer immune evasion mechanisms		
	unwanted immune reactions		
	lymphocyte infiltration		
	excessive complement activation		
	adaptive immune functions		
	immunological correlation		
	cancer-related immune disorder		
	glioma progression		
	long-lasting inflammatory conditions		
	convergent antibody responses		
	highly aggressive tumors		
	sepsis-induced loss		
	cell turnover		
	immune dynamics		
	immune-mediated tissue damage		
	tumor-restricted glycoepitopes		
	post-translational modifications		
	phage peptide antigen presentation		
	bacterial infections		
	tissue-specific expression differences		
	pathogenesis research		
	intrinsic tumor-suppressive mechanisms		
	adaptive immune function		
	new exosome-based immunotherapeutic approaches		
	immune signaling		
	lymphocyte activation		
	cellular immunity		
	anti-tumor immune responses		
	basal cells		
	human lung epithelial development		
	type-2 immune responses		
	chronic fur mite infestation		
	binary cell fate decisions		
	oxidative phosphorylation		
	metabolic regulation		

HSP_Stres	KG_annotation (ranked by pagerank scor	ORA_annotation	LLM_annotation
CACYBP	immune response	REACTOME_ATTENUATION_PHASE	Heat Shock Response and Cellular Stress Management (0.85)
HSPA1B	systemic inflammatory response syndrom	REACTOME_HSF1_DEPENDENT_TRANSACTIVATION	
CD69	immune signaling	REACTOME_HSF1_ACTIVATION	
DNAJB1	vivo cell proliferation	REACTOME_CELLULAR_RESPONSE_TO_HEAT_STRESS	
HSPB1	myeloid differentiation	HALLMARK_TNFA_SIGNALING_VIA_NFKB	
NR4A1	complete remission	REACTOME_REGULATION_OF_HSF1_MEDIATED_HEAT_SHOCK_RESPONSE	
DEDD2	median duration	REACTOME_HSP90_CHAPERONE_CYCLE_FOR_STEROID_HORMONE_RECEPTORS_SHR_IN_THE_PRESENCE_OF_LIGAND	
FKBP4	cell proliferation	WP_MAPK_SIGNALING_PATHWAY	
HSPA1A	tumor microenvironments	REACTOME_CHAPERONE_MEDIATED_AUTOPHAGY	
HSPA6	tumor evolution	KEGG_MAPK_SIGNALING_PATHWAY	
HSPD1	macrophage differentiation	WP_VEGFAVEGFR2_SIGNALING_PATHWAY	
CCR7	basal cells	WP_MEASLES_VIRUS_INFECTION	
CHORDC1	human lung epithelial development	KEGG_ANTIGEN_PROCESSING_AND_PRESENTATION	
DNAJA1	severe neurotoxicity	WP_APOPTOSIS_MODULATION_AND_SIGNALING	
EGR1	cytokine release syndrome	REACTOME_ESTROGEN_DEPENDENT_GENE_EXPRESSION	
EGR3	cancer cell motility	REACTOME_ESR_MEDIATED_SIGNALING	
HSP90AA1	neuromyelitis optica	REACTOME_MAPK6_MAPK4_SIGNALING	
HSP90AB1	giant cell arteritis	HALLMARK_UV_RESPONSE_UP	
HSPE1	immune escape	REACTOME_AUF1_HNRNP_D0_BINDS_AND_DESTABILIZES_MRNA	
HSPH1	co-receptor function	REACTOME_SIGNALING_BY_INTERLEUKINS	
JUN	immune microenvironment changes	REACTOME_SIGNALING_BY_NUCLEAR_RECEPTORS	
KLF6	hematopoietic cellular products	REACTOME_DDX58_IFIH1_MEDIATED_INDUCTION_OF_INTERFERON_ALPHA_BETA	
MRPL18	standard allogeneic grafts	WP_NRF2_PATHWAY	
MYC	cell turnover	WP_NUCLEAR_RECEPTORS_METAPATHWAY	
NFKBID	peripheral angiogenesis	REACTOME_NF_KB_IS_ACTIVATED_AND_SIGNALS_SURVIVAL	
SLBP	tumor suppression	WP_NEUROINFLAMMATION	
STIP1	stem cells	REACTOME_REGULATION_OF_MRNA_STABILITY_BY_PROTEINS_THAT_BIND_AU_RICH_ELEMENTS	
TAF7	intrinsic tumor-suppressive mechanisms	REACTOME_SARS_COV_INFECTIONS	
UBC	relapse prevention	REACTOME_TOLL_LIKE_RECEPTOR_9_TLR9_CASCADE	
ZFAND2A	lymphocyte activation	HALLMARK_APOPTOSIS	
PMAIP1		REACTOME_MYD88_INDEPENDENT_TLR4_CASCADE	
CCL4		REACTOME_P75NTR_SIGNALS_VIA_NF_KB	
UBB		WP_MAPK_PATHWAY_IN_CONGENITAL_THYROID_CANCER	
NFKBIA		WP_QUERCETIN_AND_NFKB_AP1_INDUCED_APOPTOSIS	
NUDC		REACTOME_TOLL_LIKE_RECEPTOR_TLR1_TLR2_CASCADE	
SLC5A3		REACTOME_CONSTITUTIVE_SIGNALING_BY_LIGAND_RESPONSIVE_EGFR_CANCER_VARIANTS	
AHSA1		REACTOME_INTERLEUKIN_4_AND_INTERLEUKIN_13_SIGNALING	
IGHA1		WP_IL18_SIGNALING_PATHWAY	
ANKRD12		WP_GASTRIN_SIGNALING_PATHWAY	
IER2		HALLMARK_MYC_TARGETS_V2	
SMIM14		WP_TCELL_ANTIGEN_RECEPTOR_TCR_PATHWAY_DURING_STAPHYLOCOCCUS_AUREUS_INFECTION	
HSPA8		KEGG_SPLICEOSOME	
DUSP2		WP_THYROID_STIMULATING_HORMONE_TSH_SIGNALING_PATHWAY	
FOS		REACTOME_SIGNALING_BY_EGFR_IN_CANCER	
MIR155HG		WP_PARKINUBIQUITIN_PROTEASOMAL_SYSTEM_PATHWAY	
BCL2A1		REACTOME_INTERLEUKIN_17_SIGNALING	
CD83		REACTOME_MAPK_FAMILY_SIGNALING_CASCADES	
DDX5		WP_CHROMOSOMAL_AND_MICROSATELLITE_INSTABILITY_IN_COLORECTAL_CANCER	
DNAJB4		WP_PROLACTIN_SIGNALING_PATHWAY	
IGHG1		REACTOME_DOWNREGULATION_OF_ERBB2_SIGNALING	
		REACTOME_RIPK1_MEDIATED_REGULATED_NECROSIS	
		REACTOME_THE_ROLE_OF_GTSE1_IN_G2_M_PROGRESSION_AFTER_G2_CHECKPOINT	
		REACTOME_AUTOPHAGY	
		WP_OREXIN_RECEPTOR_PATHWAY	
		REACTOME_SELECTIVE_AUTOPHAGY	
		REACTOME_TOLL_LIKE_RECEPTOR_CASCADES	
		REACTOME_SMAD2_SMAD3_SMAD4_HETEROTRIMER_REGULATES_TRANSCRIPTION	
		REACTOME_TAK1_ACTIVATES_NFKB_BY_PHOSPHORYLATION_AND_ACTIVATION_OF_IKKS_COMPLEX	
		WP_HOSTPATHOGEN_INTERACTION_OF_HUMAN_CORONAVIRUSES_INTERFERON_INDUCTION	
		WP_APOPTOSIS	
		REACTOME_LATE_ENDOSOMAL_MICROAUTOPHAGY	
		KEGG_PRION_DISEASES	
		WP_TCELL_RECEPTOR_TCR_SIGNALING_PATHWAY	
		WP_CORTICOTROPINRELEASING_HORMONE_SIGNALING_PATHWAY	
		REACTOME_NGF_STIMULATED_TRANSCRIPTION	
		WP_IL5_SIGNALING_PATHWAY	
		WP_PDGF_PATHWAY	
		KEGG_TOLL_LIKE_RECEPTOR_SIGNALING_PATHWAY	
		WP_IL2_SIGNALING_PATHWAY	
		REACTOME_FC_EPSILON_RECEPTOR_FCERI_SIGNALING	
		REACTOME_NEGATIVE_REGULATION_OF_MAPK_PATHWAY	
		WP_TOLLLIKE_RECEPTOR_SIGNALING_PATHWAY	
		REACTOME_AGGREPHAGY	
		REACTOME_TRANSCRIPTIONAL_ACTIVITY_OF_SMAD2_SMAD3_SMAD4_HETEROTRIMER	
		REACTOME_ACTIVATION_OF_THE_AP_1_FAMILY_OF_TRANSCRIPTION_FACTORS	
		REACTOME_IRAK2_MEDIATED_ACTIVATION_OF_TAK1_COMPLEX	
		REACTOME_SENESCENCE_ASSOCIATED_SECRETORY_PHENOTYPE_SASP	
		HALLMARK_ESTROGEN_RESPONSE_EARLY	
		HALLMARK_INFLAMMATORY_RESPONSE	
		REACTOME_NOTCH1_INTRACELLULAR_DOMAIN_REGULATES_TRANSCRIPTION	
		WP_ARYL_HYDROCARBON_RECEPTOR_PATHWAY_WP2586	
		WP_IL3_SIGNALING_PATHWAY	
		REACTOME_ALPHA_PROTEIN_KINASE_1_SIGNALING_PATHWAY	
		REACTOME_PEXOPHAGY	
		REACTOME_REGULATION_OF_BACH1_ACTIVITY	
		REACTOME_TICAM1_TRAF6_DEPENDENT_INDUCTION_OF_TAK1_COMPLEX	
		WP_CIRCADIAN_RHYTHM_GENES	
		REACTOME_SARS_COV_1_INFECTION	
		REACTOME_SIGNALING_BY_ERBB2	
		WP_INTERLEUKIN1_IL1_STRUCTURAL_PATHWAY	
		WP_PHOTODYNAMIC_THERAPYINDUCED_AP1_SURVIVAL_SIGNALING	
		WP_SPINAL_CORD_INJURY	
		KEGG_PATHWAYS_IN_CANCER	
		REACTOME_JOSEPHIN_DOMAIN_DUBS	
		REACTOME_TICAM1_DEPENDENT_ACTIVATION_OF_IRF3_IRF7	
		REACTOME_OXIDATIVE_STRESS_INDUCED_SENESCENCE	
		WP_RANKLRANK_SIGNALING_PATHWAY	
		WP_NETWORK_MAP_OF_SARSCOV2_SIGNALING_PATHWAY	
		REACTOME_DOWNREGULATION_OF_ERBB2_ERBB3_SIGNALING	
		REACTOME_P75NTR_RECRUITS_SIGNALLING_COMPLEXES	
		REACTOME_SUPPRESSION_OF_PHAGOSOMAL_MATURATION	
		REACTOME_TRAF6_MEDIATED_IRF7_ACTIVATION_IN_TLR7_8_OR_9_SIGNALING	
		REACTOME_NUCLEOTIDE_BINDING_DOMAIN_LEUCINE_RICH_REPEAT_CONTAINING_RECEPTOR_NLR_SIGNALING_PATHWAYS	
		REACTOME_REGULATED_NECROSIS	
		REACTOME_SIGNALING_BY_NOTCH1_PEST_DOMAIN_MUTANTS_IN_CANCER	
		REACTOME_IRAK1_RECRUITS_IKK_COMPLEX	
		WP_TGFBETA_SIGNALING_PATHWAY	
		REACTOME_NUCLEAR_EVENTS_KINASE_AND_TRANSCRIPTION_FACTOR_ACTIVATION	
		REACTOME_INLB_MEDIATED_ENTRY_OF_LISTERIA_MONOCYTOGENES_INTO_HOST_CELL	
		REACTOME_NEGATIVE_REGULATION_OF_FLT3	
		REACTOME_REGULATION_OF_INNATE_IMMUNE_RESPONSES_TO_CYTOSOLIC_DNA	
		REACTOME_SIGNALING_BY_NOTCH1_HD_DOMAIN_MUTANTS_IN_CANCER	
		WP_LET7_INHIBITION_OF_ES_CELL_REPROGRAMMING	
		KEGG_COLORECTAL_CANCER	
		KEGG_NOD_LIKE_RECEPTOR_SIGNALING_PATHWAY	
		REACTOME_PTEN_REGULATION	
		WP_MIRNAS_INVOLVEMENT_IN_THE_IMMUNE_RESPONSE_IN_SEPSIS	
		REACTOME_CYTOSOLIC_SENSORS_OF_PATHOGEN_ASSOCIATED_DNA	
		REACTOME_ASSEMBLY_OF_THE_HIV_VIRION	
		REACTOME_GLYCOGEN_STORAGE_DISEASES	
		REACTOME_GLYCOGEN_SYNTHESIS	
		REACTOME_MAP3K8_TPL2_DEPENDENT_MAPK1_3_ACTIVATION	
		REACTOME_NRIF_SIGNALS_CELL_DEATH_FROM_THE_NUCLEUS	
		REACTOME_SEMA3A_PAK_DEPENDENT_AXON_REPULSION	
		REACTOME_SPRY_REGULATION_OF_FGF_SIGNALING	
		REACTOME_TGF_BETA_RECEPTOR_SIGNALING_IN_EMT_EPITHELIAL_TO_MESENCHYMAL_TRANSITION	
		REACTOME_TRAF6_MEDIATED_INDUCTION_OF_TAK1_COMPLEX_WITHIN_TLR4_COMPLEX	
		REACTOME_TRANSCRIPTIONAL_REGULATION_BY_TP53	
		WP_BRAINDERIVED_NEUROTROPHIC_FACTOR_BDNF_SIGNALING_PATHWAY	
		WP_ONCOSTATIN_M_SIGNALING_PATHWAY	
		REACTOME_ACTIVATION_OF_IRF3_IRF7_MEDIATED_BY_TBK1_IKK_EPSILON	
		REACTOME_TRANSLESION_SYNTHESIS_BY_POLK	
		REACTOME_SARS_COV_2_INFECTION	
		WP_HEPATITIS_B_INFECTION	
		KEGG_LEISHMANIA_INFECTION	
		REACTOME_REGULATION_OF_TP53_ACTIVITY_THROUGH_METHYLATION	
		REACTOME_TICAM1_RIP1_MEDIATED_IKK_COMPLEX_RECRUITMENT	
		REACTOME_TRANSLESION_SYNTHESIS_BY_POLH	
		REACTOME_SIGNALING_BY_NOTCH1	
		KEGG_B_CELL_RECEPTOR_SIGNALING_PATHWAY	
		REACTOME_EXTRA_NUCLEAR_ESTROGEN_SIGNALING	
		REACTOME_SIGNALING_BY_TGF_BETA_RECEPTOR_COMPLEX	
		REACTOME_LISTERIA_MONOCYTOGENES_ENTRY_INTO_HOST_CELLS	
		WP_TP53_NETWORK	
		REACTOME_ER_QUALITY_CONTROL_COMPARTMENT_ERQC	
		REACTOME_NEGATIVE_REGULATION_OF_MET_ACTIVITY	
		REACTOME_REGULATION_OF_FZD_BY_UBIQUITINATION	
		REACTOME_JNK_C_JUN_KINASES_PHOSPHORYLATION_AND_ACTIVATION_MEDIATED_BY_ACTIVATED_HUMAN_TAK1	
		REACTOME_NOTCH2_ACTIVATION_AND_TRANSMISSION_OF_SIGNAL_TO_THE_NUCLEUS	
		REACTOME_PINK1_PRKN_MEDIATED_MITOPHAGY	
		REACTOME_REGULATION_OF_SIGNALING_BY_CBL	
		WP_REGULATION_OF_APOPTOSIS_BY_PARATHYROID_HORMONERELATED_PROTEIN	
		REACTOME_DOWNSTREAM_SIGNALING_EVENTS_OF_B_CELL_RECEPTOR_BCR	
		REACTOME_ACTIVATED_TAK1_MEDIATES_P38_MAPK_ACTIVATION	
		REACTOME_IKK_COMPLEX_RECRUITMENT_MEDIATED_BY_RIP1	
		REACTOME_RESPONSE_OF_MTB_TO_PHAGOCYTOSIS	
		REACTOME_RHOBTB2_GTPASE_CYCLE	
		WP_ESTROGEN_SIGNALING_PATHWAY	
		WP_PHOTODYNAMIC_THERAPYINDUCED_NFE2L2_NRF2_SURVIVAL_SIGNALING	
		REACTOME_CYCLIN_A_CDK2_ASSOCIATED_EVENTS_AT_S_PHASE_ENTRY	
		REACTOME_DEGRADATION_OF_BETA_CATENIN_BY_THE_DESTRUCTION_COMPLEX	
		REACTOME_APC_C_CDC20_MEDIATED_DEGRADATION_OF_CYCLIN_B	
		REACTOME_RAS_PROCESSING	
		WP_EBSTEINBARR_VIRUS_LMP1_SIGNALING	
		REACTOME_DISEASES_OF_SIGNAL_TRANSDUCTION_BY_GROWTH_FACTOR_RECEPTORS_AND_SECOND_MESSENGERS	
		REACTOME_POTENTIAL_THERAPEUTICS_FOR_SARS	
		REACTOME_REGULATION_OF_PLK1_ACTIVITY_AT_G2_M_TRANSITION	
		WP_TCELL_ACTIVATION_SARSCOV2	
		KEGG_ENDOCYTOSIS	
		REACTOME_GAP_FILLING_DNA_REPAIR_SYNTHESIS_AND_LIGATION_IN_GG_NER	
		REACTOME_INACTIVATION_OF_CSF3_G_CSF_SIGNALING	
		REACTOME_NOTCH3_ACTIVATION_AND_TRANSMISSION_OF_SIGNAL_TO_THE_NUCLEUS	
		WP_PHYSIOLOGICAL_AND_PATHOLOGICAL_HYPERTROPHY_OF_THE_HEART	
		KEGG_PROSTATE_CANCER	
		REACTOME_APC_CDC20_MEDIATED_DEGRADATION_OF_NEK2A	
		REACTOME_CALNEXIN_CALRETICULIN_CYCLE	
		REACTOME_DOWNREGULATION_OF_TGF_BETA_RECEPTOR_SIGNALING	
		REACTOME_INTRACELLULAR_SIGNALING_BY_SECOND_MESSENGERS	
		REACTOME_REGULATION_OF_TP53_ACTIVITY_THROUGH_PHOSPHORYLATION	
		REACTOME_GLYCOGEN_METABOLISM	
		REACTOME_INFECTION_WITH_MYCOBACTERIUM_TUBERCULOSIS	
		WP_TNFALPHA_SIGNALING_PATHWAY	
		REACTOME_BUDDING_AND_MATURATION_OF_HIV_VIRION	
		REACTOME_DOWNREGULATION_OF_SMAD2_3_SMAD4_TRANSCRIPTIONAL_ACTIVITY	
		REACTOME_FLT3_SIGNALING_IN_DISEASE	
		WP_INTERACTIONS_OF_NATURAL_KILLER_CELLS_IN_PANCREATIC_CANCER	
		REACTOME_TRANSCRIPTIONAL_REGULATION_BY_RUNX3	
		REACTOME_CELLULAR_SENESCENCE	
		REACTOME_P75_NTR_RECEPTOR_MEDIATED_SIGNALLING	
		REACTOME_MITOPHAGY	
		REACTOME_NEGATIVE_REGULATION_OF_FGFR3_SIGNALING	
		REACTOME_TRANSLATION_OF_SARS_COV_1_STRUCTURAL_PROTEINS	
		REACTOME_UPTAKE_AND_ACTIONS_OF_BACTERIAL_TOXINS	
		WP_PDGFRBETA_PATHWAY	
		WP_SELECTIVE_EXPRESSION_OF_CHEMOKINE_RECEPTORS_DURING_TCELL_POLARIZATION	
		REACTOME_BINDING_AND_UPTAKE_OF_LIGANDS_BY_SCAVENGER_RECEPTORS	
		WP_B_CELL_RECEPTOR_SIGNALING_PATHWAY	
		HALLMARK_ESTROGEN_RESPONSE_LATE	
		HALLMARK_MTORC1_SIGNALING	
		HALLMARK_MYC_TARGETS_V1	
		REACTOME_MITOTIC_G2_G2_M_PHASES	
		REACTOME_RECOGNITION_OF_DNA_DAMAGE_BY_PCNA_CONTAINING_REPLICATION_COMPLEX	
		REACTOME_SIGNALING_BY_CSF3_G_CSF	
		REACTOME_SYNTHESIS_OF_ACTIVE_UBIQUITIN_ROLES_OF_E1_AND_E2_ENZYMES	
		REACTOME_TNFR1_INDUCED_NFKAPPAB_SIGNALING_PATHWAY	
		REACTOME_CLEC7A_DECTIN_1_SIGNALING	
		REACTOME_INTERLEUKIN_1_SIGNALING	
		REACTOME_ACTIVATED_NOTCH1_TRANSMITS_SIGNAL_TO_THE_NUCLEUS	
		REACTOME_EGFR_DOWNREGULATION	
		REACTOME_ENDOSOMAL_SORTING_COMPLEX_REQUIRED_FOR_TRANSPORT_ESCRT	
		REACTOME_MAPK_TARGETS_NUCLEAR_EVENTS_MEDIATED_BY_MAP_KINASES	
		REACTOME_NEGATIVE_REGULATION_OF_FGFR4_SIGNALING	
		REACTOME_NEUTROPHIL_DEGRANULATION	
		REACTOME_SIGNALING_BY_TGFB_FAMILY_MEMBERS	
		REACTOME_PROGRAMMED_CELL_DEATH	
		REACTOME_TERMINATION_OF_TRANSLESION_DNA_SYNTHESIS	
		REACTOME_TGF_BETA_RECEPTOR_SIGNALING_ACTIVATES_SMADS	
		REACTOME_NEGATIVE_REGULATION_OF_FGFR1_SIGNALING	
		REACTOME_SIGNALING_BY_NOTCH2	
		WP_GASTRIC_CANCER_NETWORK_2	
		KEGG_T_CELL_RECEPTOR_SIGNALING_PATHWAY	
		REACTOME_DISEASES_OF_CARBOHYDRATE_METABOLISM	
		REACTOME_NEGATIVE_REGULATION_OF_FGFR2_SIGNALING	
		WP_HEPATOCYTE_GROWTH_FACTOR_RECEPTOR_SIGNALING	
		WP_P38_MAPK_SIGNALING_PATHWAY	
		REACTOME_UB_SPECIFIC_PROCESSING_PROTEASES	
		REACTOME_METALLOPROTEASE_DUBS	
		REACTOME_N_GLYCAN_TRIMMING_IN_THE_ER_AND_CALNEXIN_CALRETICULIN_CYCLE	
		REACTOME_NEGATIVE_REGULATORS_OF_DDX58_IFIH1_SIGNALING	
		REACTOME_ONCOGENE_INDUCED_SENESCENCE	
		REACTOME_REGULATION_OF_TNFR1_SIGNALING	
		REACTOME_RHOBTB_GTPASE_CYCLE	
		WP_SIGNAL_TRANSDUCTION_THROUGH_IL1R	
		REACTOME_NOD1_2_SIGNALING_PATHWAY	
		WP_HOSTPATHOGEN_INTERACTION_OF_HUMAN_CORONAVIRUSES_MAPK_SIGNALING	
		WP_DNA_DAMAGE_RESPONSE_ONLY_ATM_DEPENDENT	
		REACTOME_REGULATION_OF_TP53_EXPRESSION_AND_DEGRADATION	
		WP_PHOTODYNAMIC_THERAPYINDUCED_HIF1_SURVIVAL_SIGNALING	
		REACTOME_DNA_DAMAGE_RECOGNITION_IN_GG_NER	
		REACTOME_FLT3_SIGNALING	
		REACTOME_TRANSCRIPTIONAL_REGULATION_BY_THE_AP_2_TFAP2_FAMILY_OF_TRANSCRIPTION_FACTORS	
		REACTOME_FANCONI_ANEMIA_PATHWAY	
		REACTOME_OVARIAN_TUMOR_DOMAIN_PROTEASES	
		REACTOME_TRANSLESION_SYNTHESIS_BY_Y_FAMILY_DNA_POLYMERASES_BYPASSES_LESIONS_ON_DNA_TEMPLATE	
		REACTOME_SIGNALING_BY_FGFR3	
		WP_ATM_SIGNALING_PATHWAY	
		REACTOME_TCR_SIGNALING	
		REACTOME_DUAL_INCISION_IN_GG_NER	
		REACTOME_SIGNALING_BY_FGFR4	
		WP_NEURAL_CREST_CELL_MIGRATION_DURING_DEVELOPMENT	
		WP_NUCLEOTIDEBINDING_OLIGOMERIZATION_DOMAIN_NOD_PATHWAY	
		REACTOME_DEACTIVATION_OF_THE_BETA_CATENIN_TRANSACTIVATING_COMPLEX	
		WP_TNFRELATED_WEAK_INDUCER_OF_APOPTOSIS_TWEAK_SIGNALING_PATHWAY	
		REACTOME_SIGNALING_BY_NOTCH	
		REACTOME_FORMATION_OF_INCISION_COMPLEX_IN_GG_NER	
		REACTOME_TNF_SIGNALING	
		WP_FAS_LIGAND_PATHWAY_AND_STRESS_INDUCTION_OF_HEAT_SHOCK_PROTEINS	
		WP_NEURAL_CREST_CELL_MIGRATION_IN_CANCER	
		REACTOME_SIGNALING_BY_NTRKS	
		REACTOME_FCERI_MEDIATED_NF_KB_ACTIVATION	
		REACTOME_CYCLIN_D_ASSOCIATED_EVENTS_IN_G1	
		WP_THYMIC_STROMAL_LYMPHOPOIETIN_TSLP_SIGNALING_PATHWAY	
		REACTOME_DNA_DAMAGE_BYPASS	
		REACTOME_INTERLEUKIN_3_INTERLEUKIN_5_AND_GM_CSF_SIGNALING	
		WP_ARYL_HYDROCARBON_RECEPTOR_PATHWAY_WP2873	
		REACTOME_C_TYPE_LECTIN_RECEPTORS_CLRS	
		REACTOME_INTERLEUKIN_1_FAMILY_SIGNALING	
		REACTOME_DEATH_RECEPTOR_SIGNALLING	
		REACTOME_SIGNALING_BY_NOTCH3	
		REACTOME_TRANSLATION_OF_SARS_COV_2_STRUCTURAL_PROTEINS	
		WP_EXERCISEINDUCED_CIRCADIAN_REGULATION	
		REACTOME_FCGAMMA_RECEPTOR_FCGR_DEPENDENT_PHAGOCYTOSIS	
		REACTOME_SIGNALING_BY_EGFR	
		REACTOME_SIGNALING_BY_FGFR1	
		WP_HEPATITIS_C_AND_HEPATOCELLULAR_CARCINOMA	
		REACTOME_CLATHRIN_MEDIATED_ENDOCYTOSIS	
		REACTOME_BETA_CATENIN_INDEPENDENT_WNT_SIGNALING	
		REACTOME_MITOTIC_G1_PHASE_AND_G1_S_TRANSITION	
		REACTOME_FORMATION_OF_TC_NER_PRE_INCISION_COMPLEX	
		KEGG_WNT_SIGNALING_PATHWAY	
		REACTOME_NEGATIVE_REGULATION_OF_NOTCH4_SIGNALING	
		REACTOME_SIGNALING_BY_PTK6	
		WP_APOPTOSISRELATED_NETWORK_DUE_TO_ALTERED_NOTCH3_IN_OVARIAN_CANCER	
		REACTOME_HIV_LIFE_CYCLE	
		KEGG_CYTOSOLIC_DNA_SENSING_PATHWAY	
		REACTOME_DEGRADATION_OF_AXIN	
		REACTOME_REGULATION_OF_RUNX3_EXPRESSION_AND_ACTIVITY	
		WP_IL4_SIGNALING_PATHWAY	
		WP_BREAST_CANCER_PATHWAY	
		REACTOME_DEGRADATION_OF_DVL	
		REACTOME_STABILIZATION_OF_P53	
		WP_IL1_SIGNALING_PATHWAY	
		REACTOME_CHEMOKINE_RECEPTORS_BIND_CHEMOKINES	
		REACTOME_IRON_UPTAKE_AND_TRANSPORT	
		REACTOME_SIGNALING_BY_ERBB4	
		WP_TGFBETA_RECEPTOR_SIGNALING	
		REACTOME_REGULATION_OF_TP53_ACTIVITY	
		WP_INSULIN_SIGNALING	
		REACTOME_CDT1_ASSOCIATION_WITH_THE_CDC6_ORC_ORIGIN_COMPLEX	
		REACTOME_E3_UBIQUITIN_LIGASES_UBIQUITINATE_TARGET_PROTEINS	
		REACTOME_RHO_GTPASE_CYCLE	
		REACTOME_S_PHASE	
		WP_SUDDEN_INFANT_DEATH_SYNDROME_SIDS_SUSCEPTIBILITY_PATHWAYS	
		REACTOME_DEUBIQUITINATION	
		REACTOME_DEGRADATION_OF_GLI1_BY_THE_PROTEASOME	
		REACTOME_SCF_SKP2_MEDIATED_DEGRADATION_OF_P27_P21	
		REACTOME_PROTEIN_LOCALIZATION	
		WP_CHEMOKINE_SIGNALING_PATHWAY	
		REACTOME_DEFECTIVE_CFTR_CAUSES_CYSTIC_FIBROSIS	
		REACTOME_REGULATION_OF_PTEN_GENE_TRANSCRIPTION	
		REACTOME_SIGNALING_BY_THE_B_CELL_RECEPTOR_BCR	
		REACTOME_DECTIN_1_MEDIATED_NONCANONICAL_NF_KB_SIGNALING	
		WP_TGFBETA_RECEPTOR_SIGNALING_IN_SKELETAL_DYSPLASIAS	
		REACTOME_PEROXISOMAL_PROTEIN_IMPORT	
		WP_ENDOMETRIAL_CANCER	
		REACTOME_ASYMMETRIC_LOCALIZATION_OF_PCP_PROTEINS	
		REACTOME_SEMAPHORIN_INTERACTIONS	
		WP_PROTEASOME_DEGRADATION	
		REACTOME_DUAL_INCISION_IN_TC_NER	
		REACTOME_HEDGEHOG_LIGAND_BIOGENESIS	
		REACTOME_REGULATION_OF_HMOX1_EXPRESSION_AND_ACTIVITY	
		REACTOME_TP53_REGULATES_TRANSCRIPTION_OF_DNA_REPAIR_GENES	
		WP_AGERAGE_PATHWAY	
		REACTOME_HDR_THROUGH_HOMOLOGOUS_RECOMBINATION_HRR	
		KEGG_EPITHELIAL_CELL_SIGNALING_IN_HELICOBACTER_PYLORI_INFECTION	
		REACTOME_G1_S_DNA_DAMAGE_CHECKPOINTS	
		REACTOME_REGULATION_OF_RAS_BY_GAPS	
		WP_PHYSICOCHEMICAL_FEATURES_AND_TOXICITYASSOCIATED_PATHWAYS	
		REACTOME_APOPTOSIS	
		REACTOME_REGULATION_OF_PTEN_STABILITY_AND_ACTIVITY	
		WP_DNA_DAMAGE_RESPONSE	
		WP_RAC1PAK1P38MMP2_PATHWAY	
		REACTOME_CIRCADIAN_CLOCK	
		WP_3Q29_COPY_NUMBER_VARIATION_SYNDROME	
		REACTOME_ORC1_REMOVAL_FROM_CHROMATIN	
		WP_GLUCOCORTICOID_RECEPTOR_PATHWAY	
		KEGG_CHRONIC_MYELOID_LEUKEMIA	
		REACTOME_REGULATION_OF_RUNX2_EXPRESSION_AND_ACTIVITY	
		REACTOME_SIGNALING_BY_FGFR2	
		KEGG_CHEMOKINE_SIGNALING_PATHWAY	
		REACTOME_APC_C_CDH1_MEDIATED_DEGRADATION_OF_CDC20_AND_OTHER_APC_C_CDH1_TARGETED_PROTEINS_IN_LATE_MITOSIS_EARLY_G1	
		WP_CYTOSOLIC_DNASENSING_PATHWAY	
		REACTOME_SEPARATION_OF_SISTER_CHROMATIDS	
		REACTOME_CELLULAR_RESPONSE_TO_HYPOXIA	

Lipid-associated	ICKG_annotation (ranked by pagerank score)	ORA_annotation	LLM_annotation
APOE	gastric adenocarcinoma	HALLMARK_COMPLEMENT	Immune response and lipid metabolism (0.85)
C1QB	tumor microenvironment	KEGG_COMPLEMENT_AND_COAGULATION_CASCADES	
LGMN	extracellular matrix	KEGG_LYSOSOME	
APOC1	tumour progression	WP_COMPLEMENT_ACTIVATION	
C1QC	lymphatic invasion	WP_COMPLEMENT_SYSTEM	
C1QA	breast cancer brain metastasis	HALLMARK_COAGULATION	
GPNMB	interferon-related pathways	WP_COMPLEMENT_AND_COAGULATION_CASCADES	
RNASE1	secondary brain tumours	WP_COMPLEMENT_SYSTEM_IN_NEURONAL_DEVELOPMENT_AND_PLASTICITY	
PLD3	diverse brain tumours	REACTOME_COMPLEMENT_CASCADE	
LIPA	innate immune defense	REACTOME_PLASMA_LIPOPROTEIN_ASSEMBLY_REMODELING_AND_CLEARANCE	
PLTP	immune cell trafficking regulation	REACTOME_NEUTROPHIL_DEGRANULATION	
FOLR2	pro-tumor behavior	REACTOME_INITIAL_TRIGGERING_OF_COMPLEMENT	
NUPR1	perihilar large duct type	WP_MICROGLIA_PATHOGEN_PHAGOCYTOSIS_PATHWAY	
CTSD	disease responses	WP_NETWORK_MAP_OF_SARSCOV2_SIGNALING_PATHWAY	
SLC40A1	tumor promotion	WP_OXIDATIVE_DAMAGE_RESPONSE	
ACP5	insufficient antigen presentation	REACTOME_PLASMA_LIPOPROTEIN_ASSEMBLY	
A2M	compartment-specific immunological responses	KEGG_SYSTEMIC_LUPUS_ERYTHEMATOSUS	
MS4A4A	lymphocyte activation	REACTOME_PLASMA_LIPOPROTEIN_CLEARANCE	
TREM2	effective tumor control	WP_STATIN_INHIBITION_OF_CHOLESTEROL_PRODUCTION	
DAB2	antibody-mediated phagocytosis	KEGG_PRION_DISEASES	
SLCO2B1	macrophage-mediated phagocytosis	WP_ALLOGRAFT_REJECTION	
CTSL	tumor infiltration	REACTOME_NR1H3_NR1H2_REGULATE_GENE_EXPRESSION_LINKED_TO_CHOLESTEROL_TRANSPORT_AND_EFFLUX	
CCL18	cross-talk pathways	REACTOME_BINDING_AND_UPTAKE_OF_LIGANDS_BY_SCAVENGER_RECEPTORS	
SEPP1	macrophage phagocytosis	REACTOME_HDL_REMODELING	
CFD	anti-inflammatory responses	REACTOME_NR1H2_AND_NR1H3_MEDIATED_SIGNALING	
FUCA1	systemic bacterial infection	HALLMARK_KRAS_SIGNALING_UP	
SPP1	profound microbiome depletion	REACTOME_TRAFFICKING_AND_PROCESSING_OF_ENDOSOMAL_TLR	
TMEM176B	tumor-immune interactions	REACTOME_MHC_CLASS_II_ANTIGEN_PRESENTATION	
PLA2G7	enhanced bacterial capture ability	WP_FAMILIAL_HYPERLIPIDEMIA_TYPE_3	
CD163	follicular lymphoma	REACTOME_RESPONSE_TO_ELEVATED_PLATELET_CYTOSOLIC_CA2	
FCGR3A	proinflammatory polarization	WP_OSTEOCLAST_SIGNALING	
CD59	transient peripheral transcriptomic alterations	REACTOME_DEGRADATION_OF_THE_EXTRACELLULAR_MATRIX	
C2	improved long-term systemic antitumor immuni	REACTOME_SCAVENGING_BY_CLASS_A_RECEPTORS	
CYP27A1	murine peritoneal macrophage differentiation	REACTOME_CREATION_OF_C4_AND_C2_ACTIVATORS	
GM2A	dependent cell phagocytosis	HALLMARK_CHOLESTEROL_HOMEOSTASIS	
IFI27	protective anti-tumor immune responses	HALLMARK_IL6_JAK_STAT3_SIGNALING	
MSR1	tumor mass	KEGG_HEMATOPOIETIC_CELL_LINEAGE	
CD14	leukocyte migration	WP_INTERACTIONS_OF_NATURAL_KILLER_CELLS_IN_PANCREATIC_CANCER	
GPR34	foster metastasis formation	REACTOME_METABOLISM_OF_VITAMINS_AND_COFACTORS	
NPL	distant metastasis/cell migration	HALLMARK_EPITHELIAL_MESENCHYMAL_TRANSITION	
CTSC	hypoxia-mediated resistance	HALLMARK_MTORC1_SIGNALING	
CREG1	mediate tumor progression	HALLMARK_P53_PATHWAY	
VSIG4	tumor progress	KEGG_TOLL_LIKE_RECEPTOR_SIGNALING_PATHWAY	
LGALS3	strong tumor angiogenesis	WP_TOLLLIKE_RECEPTOR_SIGNALING_PATHWAY	
STAB1	disseminated tumor cells	REACTOME_CARGO_CONCENTRATION_IN_THE_ER	
CD9	immunosuppressive macrophage-like characteri	REACTOME_PLASMA_LIPOPROTEIN_REMODELING	
GCHFR	tumor-promoting microenvironments		
ASAH1	immunosuppressive tumor-associated macrophages		
CCL3	tumor immune microenvironment		
F13A1	somatic hypermutation		
	pro-inflammatory switch		
	increased onco-toxicity		
	pathogenic envelope viruses		
	genetic variation		
	macrophage-induced phagocytosis		
	inflammatory macrophage polarization		

Monocyte_Secreted	KG_annotation (ranked by pagerank scor	ORA_annotation	LLM_annotation
IL1B	tumor initiation	HALLMARK_TNFA_SIGNALING_VIA_NFKB	Inflammatory Response and Oxidative Stress Management (0.92)
CXCL2	cancer inflammation	REACTOME_INTERLEUKIN_10_SIGNALING	
G0S2	tumor microenvironment	WP_IL18_SIGNALING_PATHWAY	
CXCL8	antitumor immune responses	HALLMARK_INFLAMMATORY_RESPONSE	
CXCL3	cancer-related genetic expression	WP_OVERVIEW_OF_PROINFLAMMATORY_AND_PROFIBROTIC_MEDIATORS	
S100A8	early lymph node metastasis	REACTOME_CHEMOKINE_RECEPTORS_BIND_CHEMOKINES	
BCL2A1	aggressive invasion	REACTOME_SIGNALING_BY_INTERLEUKINS	
FCN1	tumor associated macrophages	KEGG_CHEMOKINE_SIGNALING_PATHWAY	
VCAN	immune checkpoint inhibitors	WP_IL1_AND_MEGAKARYOCYTES_IN_OBESITY	
IER3	established metastatic disease	HALLMARK_KRAS_SIGNALING_UP	
CCL3	phagocyte infiltration	WP_NETWORK_MAP_OF_SARSCOV2_SIGNALING_PATHWAY	
EREG	leukocyte migration	KEGG_NOD_LIKE_RECEPTOR_SIGNALING_PATHWAY	
TIMP1	immune cell trafficking regulation	WP_LUNG_FIBROSIS	
CCL20	pro-tumor behavior	WP_PROSTAGLANDIN_SIGNALING	
S100A9	epithelial-mesenchymal transition	WP_BURN_WOUND_HEALING	
TNFAIP3	immune microenvironment changes	WP_PHOTODYNAMIC_THERAPYINDUCED_NFKB_SURVIVAL_SIGNALING	
AREG	greater pathogenesis	KEGG_CYTOKINE_CYTOKINE_RECEPTOR_INTERACTION	
CCL4	chronic low-grade inflammation	WP_SARSCOV2_INNATE_IMMUNITY_EVASION_AND_CELLSPECIFIC_IMMUNE_RESPONSE	
SLC2A3	subclinical atherosclerosis	REACTOME_PEPTIDE_LIGAND_BINDING_RECEPTORS	
SOD2	tumor mass	HALLMARK_COMPLEMENT	
IL1RN	viral pathogenesis	HALLMARK_EPITHELIAL_MESENCHYMAL_TRANSITION	
PLAUR	tumor outgrowth	HALLMARK_HYPOXIA	
NFKBIA	immune suppressor cells	REACTOME_SHC1_EVENTS_IN_EGFR_SIGNALING	
DUSP2	tumor cell expansion	WP_COVID19_ADVERSE_OUTCOME_PATHWAY	
GPR183	macrophage-ferroptosis crosstalk	REACTOME_CLASS_A_1_RHODOPSIN_LIKE_RECEPTORS	
THBS1	immune checkpoint expression	HALLMARK_APOPTOSIS	
CCL3L3	tumor progress	REACTOME_GAB1_SIGNALOSOME	
OLR1	inflammation-driven cancer	KEGG_TOLL_LIKE_RECEPTOR_SIGNALING_PATHWAY	
NAMPT	glioma prognosis	WP_TOLLLIKE_RECEPTOR_SIGNALING_PATHWAY	
CCL4L2	unfavourable prognosis	REACTOME_GPCR_LIGAND_BINDING	
C15ORF48	checkpoint inhibitor pneumonitis	KEGG_CYTOSOLIC_DNA_SENSING_PATHWAY	
NLRP3	immune-related neutropenia	REACTOME_NEUTROPHIL_DEGRANULATION	
PTGS2	local cellular clustering	WP_LTF_DANGER_SIGNAL_RESPONSE_PATHWAY	
TREM1		REACTOME_INTERLEUKIN_4_AND_INTERLEUKIN_13_SIGNALING	
CD83		WP_STING_PATHWAY_IN_KAWASAKILIKE_DISEASE_AND_COVID19	
HBEGF		WP_SPINAL_CORD_INJURY	
NFKBIZ		WP_MIRNAS_INVOLVEMENT_IN_THE_IMMUNE_RESPONSE_IN_SEPSIS	
NR4A2		HALLMARK_INTERFERON_GAMMA_RESPONSE	
CD55		REACTOME_ESTROGEN_DEPENDENT_NUCLEAR_EVENTS_DOWNSTREAM_OF_ESR_MEMBRANE_SIGNALING	
LYZ		WP_EBSTEINBARR_VIRUS_LMP1_SIGNALING	
SLC11A1		REACTOME_SIGNALING_BY_EGFR_IN_CANCER	
ATF3		WP_GLUCOCORTICOID_RECEPTOR_PATHWAY	
FOSB		REACTOME_G_ALPHA_I_SIGNALLING_EVENTS	
SAMSN1		WP_CYTOSOLIC_DNASENSING_PATHWAY	
SOCS3		WP_NUCLEAR_RECEPTORS_METAPATHWAY	
PLIN2		REACTOME_DISEASES_OF_IMMUNE_SYSTEM	
SLC25A37		REACTOME_EGFR_DOWNREGULATION	
RGS2		WP_ANTIVIRAL_AND_ANTIINFLAMMATORY_EFFECTS_OF_NRF2_ON_SARSCOV2_PATHWAY	
AQP9		WP_OREXIN_RECEPTOR_PATHWAY	
PPP1R15A		HALLMARK_UV_RESPONSE_UP	
		WP_SIGNAL_TRANSDUCTION_THROUGH_IL1R	
		HALLMARK_ANGIOGENESIS	
		WP_CHEMOKINE_SIGNALING_PATHWAY	
		WP_PHOTODYNAMIC_THERAPYINDUCED_HIF1_SURVIVAL_SIGNALING	
		WP_CORTICOTROPINRELEASING_HORMONE_SIGNALING_PATHWAY	
		REACTOME_ANTIMICROBIAL_PEPTIDES	
		WP_BLADDER_CANCER	
		WP_NUCLEOTIDEBINDING_OLIGOMERIZATION_DOMAIN_NOD_PATHWAY	
		WP_COMPLEMENT_SYSTEM	
		WP_VITAMIN_D_RECEPTOR_PATHWAY	
		REACTOME_PI3K_EVENTS_IN_ERBB4_SIGNALING	
		REACTOME_NEGATIVE_REGULATION_OF_THE_PI3K_AKT_NETWORK	
		HALLMARK_ALLOGRAFT_REJECTION	
		REACTOME_SIGNALING_BY_EGFR	
		WP_VEGFAVEGFR2_SIGNALING_PATHWAY	
		REACTOME_ERBB2_ACTIVATES_PTK6_SIGNALING	
		WP_MAMMARY_GLAND_DEVELOPMENT_PATHWAY_PUBERTY_STAGE_2_OF_4	
		REACTOME_SIGNALING_BY_PTK6	
		WP_APOPTOSISRELATED_NETWORK_DUE_TO_ALTERED_NOTCH3_IN_OVARIAN_CANCER	
		REACTOME_SHC1_EVENTS_IN_ERBB4_SIGNALING	
		REACTOME_ERBB2_REGULATES_CELL_MOTILITY	
		REACTOME_RESPONSE_OF_EIF2AK1_HRI_TO_HEME_DEFICIENCY	
		REACTOME_GRB2_EVENTS_IN_ERBB2_SIGNALING	
		REACTOME_PI3K_EVENTS_IN_ERBB2_SIGNALING	
		WP_QUERCETIN_AND_NFKB_AP1_INDUCED_APOPTOSIS	
		REACTOME_IRAK4_DEFICIENCY_TLR2_4	
		KEGG_EPITHELIAL_CELL_SIGNALING_IN_HELICOBACTER_PYLORI_INFECTION	
		REACTOME_NICOTINAMIDE_SALVAGING	
		WP_OVERVIEW_OF_NANOPARTICLE_EFFECTS	
		KEGG_LEISHMANIA_INFECTION	
		WP_HYPERTROPHY_MODEL	
		REACTOME_EXTRA_NUCLEAR_ESTROGEN_SIGNALING	
		REACTOME_REGULATION_OF_TLR_BY_ENDOGENOUS_LIGAND	
		WP_LEPTIN_SIGNALING_PATHWAY	
		REACTOME_CONSTITUTIVE_SIGNALING_BY_ABERRANT_PI3K_IN_CANCER	
		REACTOME_SHC1_EVENTS_IN_ERBB2_SIGNALING	
		REACTOME_RHO_GTPASES_ACTIVATE_NADPH_OXIDASES	
		WP_NAD_METABOLISM_IN_ONCOGENEINDUCED_SENESCENCE_AND_MITOCHONDRIAL_DYSFUNCTIONASSOCIATED_SENESCENCE	
		WP_UNFOLDED_PROTEIN_RESPONSE	
		HALLMARK_IL6_JAK_STAT3_SIGNALING	
		KEGG_ERBB_SIGNALING_PATHWAY	
		REACTOME_PURINERGIC_SIGNALING_IN_LEISHMANIASIS_INFECTION	
		REACTOME_SIGNALING_BY_ERBB2_IN_CANCER	
		WP_CYTOKINES_AND_INFLAMMATORY_RESPONSE	
		WP_ALLOGRAFT_REJECTION	
		REACTOME_ATF4_ACTIVATES_GENES_IN_RESPONSE_TO_ENDOPLASMIC_RETICULUM_STRESS	
		WP_ERBB_SIGNALING_PATHWAY	
		WP_SELENIUM_MICRONUTRIENT_NETWORK	
		WP_TCELL_RECEPTOR_TCR_SIGNALING_PATHWAY	
		WP_INTERACTIONS_OF_NATURAL_KILLER_CELLS_IN_PANCREATIC_CANCER	
		REACTOME_DOWNREGULATION_OF_ERBB2_SIGNALING	
		WP_PHOTODYNAMIC_THERAPYINDUCED_UNFOLDED_PROTEIN_RESPONSE	
		WP_SELECTIVE_EXPRESSION_OF_CHEMOKINE_RECEPTORS_DURING_TCELL_POLARIZATION	
		WP_TLR4_SIGNALING_AND_TOLERANCE	
		WP_MALIGNANT_PLEURAL_MESOTHELIOMA	
		REACTOME_NICOTINATE_METABOLISM	
		WP_OLIGODENDROCYTE_SPECIFICATION_AND_DIFFERENTIATION_LEADING_TO_MYELIN_COMPONENTS_FOR_CNS	
		HALLMARK_IL2_STAT5_SIGNALING	
		HALLMARK_P53_PATHWAY	
		REACTOME_NUCLEAR_SIGNALING_BY_ERBB4	
		REACTOME_PERK_REGULATES_GENE_EXPRESSION	
		WP_DEVELOPMENT_AND_HETEROGENEITY_OF_THE_ILC_FAMILY	
		REACTOME_INTERLEUKIN_1_SIGNALING	
		REACTOME_TOLL_LIKE_RECEPTOR_TLR1_TLR2_CASCADE	
		WP_RESISTIN_AS_A_REGULATOR_OF_INFLAMMATION	
		REACTOME_CARGO_RECOGNITION_FOR_CLATHRIN_MEDIATED_ENDOCYTOSIS	
		REACTOME_PI3K_AKT_SIGNALING_IN_CANCER	
		WP_TYPE_II_INTERFERON_SIGNALING_IFNG	
		REACTOME_ESR_MEDIATED_SIGNALING	
		WP_GASTRIN_SIGNALING_PATHWAY	
		REACTOME_METABOLISM_OF_WATER_SOLUBLE_VITAMINS_AND_COFACTORS	
		KEGG_BLADDER_CANCER	
		WP_IL6_SIGNALING_PATHWAY	
		WP_ADIPOGENESIS	
		WP_TGFBETA_SIGNALING_PATHWAY	
		WP_THYMIC_STROMAL_LYMPHOPOIETIN_TSLP_SIGNALING_PATHWAY	
		WP_NOCGMPPKG_MEDIATED_NEUROPROTECTION	
		HALLMARK_COAGULATION	
		WP_MEASLES_VIRUS_INFECTION	

Cell-cycle	ICKG_annotation (ranked by pagerank score)	ORA_annotation	LLM_annotation
STMN1	cell cycle	HALLMARK_E2F_TARGETS	Cell Cycle Regulation and DNA Replication (0.95)
CKS1B	immune responses	HALLMARK_G2M_CHECKPOINT	
HMGB2	cancer development/progression	WP_RETINOBLASTOMA_GENE_IN_CANCER	
TUBB	gastric tumorigenesis	REACTOME_M_PHASE	
TK1	enhanced cancer immunogenicity	REACTOME_MITOTIC_PROMETAPHASE	
PTTG1	inflammatory periodontal tissue loss	REACTOME_MITOTIC_METAPHASE_AND_ANAPHASE	
PCNA	macrophage inflammatory proteins	REACTOME_RESOLUTION_OF_SISTER_CHROMATID_COHESION	
H2AFZ	cancer-induced muscle wasting	REACTOME_SEPARATION_OF_SISTER_CHROMATIDS	
HMGN2	periodontal disease	REACTOME_MITOTIC_G1_PHASE_AND_G1_S_TRANSITION	
TUBA1B	aging-related inflammation	REACTOME_CELL_CYCLE_CHECKPOINTS	
TYMS	chronic infection	HALLMARK_MYC_TARGETS_V1	
DUT	bacterial infection-induced pulmonary inflammat	REACTOME_RHO_GTPASES_ACTIVATE_FORMINS	
SMC4	myeloid cell development	REACTOME_RHO_GTPASE_EFFECTORS	
HIST1H4C	portal inflammation	REACTOME_MITOTIC_SPINDLE_CHECKPOINT	
CDK1	parasite infection control	REACTOME_G1_S_SPECIFIC_TRANSCRIPTION	
CKS2	severe inflammation	REACTOME_CONDENSATION_OF_PROPHASE_CHROMOSOMES	
NUSAP1	induced experimental periodontitis	REACTOME_CHROMOSOME_MAINTENANCE	
UBE2C	periodontal tissue destruction	REACTOME_CONDENSATION_OF_PROMETAPHASE_CHROMOSOMES	
BIRC5	periodontitis gingival tissue	REACTOME_MITOTIC_PROPHASE	
DTYMK	chronic pain	REACTOME_DNA_REPLICATION	
KIAA0101	cellular antitumor immunity	HALLMARK_MITOTIC_SPINDLE	
ZWINT	endogenous antitumor immunity	WP_G1_TO_S_CELL_CYCLE_CONTROL	
DEK	high plasma cell proportion	REACTOME_DEPOSITION_OF_NEW_CENPA_CONTAINING_NUCLEOSOMES_AT_THE_CENTROMERE	
RANBP1	macrophage heterogeneity	REACTOME_G0_AND_EARLY_G1	
HMGB1	castration-resistant metastatic disease	REACTOME_NUCLEAR_ENVELOPE_NE_REASSEMBLY	
SMC2	cell communication	REACTOME_INTERCONVERSION_OF_NUCLEOTIDE_DI_AND_TRIPHOSPHATES	
TMEM106C	germinal center formation	WP_GASTRIC_CANCER_NETWORK_1	
CENPW	primary biliary cholangitis	HALLMARK_DNA_REPAIR	
RPA3	dysregulated immune reactions	REACTOME_DNA_STRAND_ELONGATION	
IDH2	marked inflammation	REACTOME_TERMINATION_OF_TRANSLESION_DNA_SYNTHESIS	
KPNA2	cancer immune equilibrium	REACTOME_S_PHASE	
MKI67	phagocyte infiltration	WP_PYRIMIDINE_METABOLISM	
TOP2A	tumor-supportive inflammation	WP_FLUOROPYRIMIDINE_ACTIVITY	
GGH	chemokine receptors	REACTOME_BASE_EXCISION_REPAIR	
HMGB3	antitumor effectiveness	KEGG_GAP_JUNCTION	
CENPF	myocardial necrosis	KEGG_DNA_REPLICATION	
MCM7	persistent viral replication	REACTOME_RECRUITMENT_OF_NUMA_TO_MITOTIC_CENTROSOMES	
PCLAF		REACTOME_METABOLISM_OF_NUCLEOTIDES	
NUCKS1		REACTOME_TRANSLESION_SYNTHESIS_BY_Y_FAMILY_DNA_POLYMERASES_BYPASSES_LESIONS_ON_DNA_TEMPLATE	
CDKN3		KEGG_PYRIMIDINE_METABOLISM	
CARHSP1		REACTOME_SUMOYLATION	
TMPO		WP_DNA_REPLICATION	
SKA2		REACTOME_SUMOYLATION_OF_DNA_REPLICATION_PROTEINS	
H2AFV		REACTOME_MITOTIC_G2_G2_M_PHASES	
H2AZ1		REACTOME_TELOMERE_MAINTENANCE	
ANP32E		REACTOME_DNA_DAMAGE_BYPASS	
CENPM		REACTOME_SYNTHESIS_OF_DNA	
DNMT1		REACTOME_DNA_REPAIR	
TUBB4B		WP_CELL_CYCLE	
H4C3		KEGG_CELL_CYCLE	
		WP_PATHOGENIC_ESCHERICHIA_COLI_INFECTION	
		KEGG_PATHOGENIC_ESCHERICHIA_COLI_INFECTION	
		REACTOME_APOPTOSIS_INDUCED_DNA_FRAGMENTATION	
		REACTOME_DEPOLYMERISATION_OF_THE_NUCLEAR_LAMINA	
		REACTOME_MISMATCH_REPAIR	
		REACTOME_PROCESSIVE_SYNTHESIS_ON_THE_LAGGING_STRAND	
		REACTOME_DNA_METHYLATION	
		REACTOME_DNA_REPLICATION_PRE_INITIATION	
		REACTOME_EPIGENETIC_REGULATION_OF_GENE_EXPRESSION	
		REACTOME_TRANSLESION_SYNTHESIS_BY_POLK	
		WP_PARKINUBIQUITIN_PROTEASOMAL_SYSTEM_PATHWAY	
		REACTOME_TP53_REGULATES_TRANSCRIPTION_OF_GENES_INVOLVED_IN_G2_CELL_CYCLE_ARREST	
		REACTOME_PRC2_METHYLATES_HISTONES_AND_DNA	
		REACTOME_AURKA_ACTIVATION_BY_TPX2	
		REACTOME_INITIATION_OF_NUCLEAR_ENVELOPE_NE_REFORMATION	
		REACTOME_PROCESSIVE_SYNTHESIS_ON_THE_C_STRAND_OF_THE_TELOMERE	
		REACTOME_TRANSCRIPTION_OF_E2F_TARGETS_UNDER_NEGATIVE_CONTROL_BY_DREAM_COMPLEX	
		REACTOME_TRANSLESION_SYNTHESIS_BY_POLH	
		WP_NUCLEOTIDE_EXCISION_REPAIR_IN_XERODERMA_PIGMENTOSUM	
		REACTOME_LAGGING_STRAND_SYNTHESIS	
		REACTOME_PHOSPHORYLATION_OF_THE_APC_C	
		REACTOME_DNA_DOUBLE_STRAND_BREAK_REPAIR	
		REACTOME_G2_M_CHECKPOINTS	
		REACTOME_PCNA_DEPENDENT_LONG_PATCH_BASE_EXCISION_REPAIR	
		REACTOME_TRANSPORT_OF_CONNEXONS_TO_THE_PLASMA_MEMBRANE	
		REACTOME_THE_ROLE_OF_GTSE1_IN_G2_M_PROGRESSION_AFTER_G2_CHECKPOINT	
		REACTOME_RECRUITMENT_OF_MITOTIC_CENTROSOME_PROTEINS_AND_COMPLEXES	
		KEGG_MISMATCH_REPAIR	
		REACTOME_POST_CHAPERONIN_TUBULIN_FOLDING_PATHWAY	
		WP_DNA_MISMATCH_REPAIR	
		REACTOME_ORGANELLE_BIOGENESIS_AND_MAINTENANCE	
		REACTOME_MEIOTIC_RECOMBINATION	
		REACTOME_APC_C_CDC20_MEDIATED_DEGRADATION_OF_CYCLIN_B	
		REACTOME_REGULATION_OF_PLK1_ACTIVITY_AT_G2_M_TRANSITION	
		REACTOME_APC_C_MEDIATED_DEGRADATION_OF_CELL_CYCLE_PROTEINS	
		REACTOME_TRANSCRIPTIONAL_REGULATION_OF_GRANULOPOIESIS	
		REACTOME_B_WICH_COMPLEX_POSITIVELY_REGULATES_RRNA_EXPRESSION	
		REACTOME_GAP_FILLING_DNA_REPAIR_SYNTHESIS_AND_LIGATION_IN_GG_NER	
		REACTOME_RESOLUTION_OF_AP_SITES_VIA_THE_MULTIPLE_NUCLEOTIDE_PATCH_REPLACEMENT_PATHWAY	
		REACTOME_FORMATION_OF_TUBULIN_FOLDING_INTERMEDIATES_BY_CCT_TRIC	
		REACTOME_ACTIVATION_OF_NMDA_RECEPTORS_AND_POSTSYNAPTIC_EVENTS	
		REACTOME_G2_M_DNA_DAMAGE_CHECKPOINT	
		REACTOME_ANCHORING_OF_THE_BASAL_BODY_TO_THE_PLASMA_MEMBRANE	
		REACTOME_ACTIVATION_OF_AMPK_DOWNSTREAM_OF_NMDARS	
		REACTOME_CILIUM_ASSEMBLY	
		REACTOME_DISEASES_OF_PROGRAMMED_CELL_DEATH	
		REACTOME_RECOGNITION_OF_DNA_DAMAGE_BY_PCNA_CONTAINING_REPLICATION_COMPLEX	
		WP_BASE_EXCISION_REPAIR	
		WP_ONECARBON_METABOLISM	
		REACTOME_POSITIVE_EPIGENETIC_REGULATION_OF_RRNA_EXPRESSION	
		REACTOME_RHO_GTPASES_ACTIVATE_IQGAPS	
		REACTOME_SEALING_OF_THE_NUCLEAR_ENVELOPE_NE_BY_ESCRT_III	
		WP_TRANSSULFURATION_AND_ONECARBON_METABOLISM	
		REACTOME_NEGATIVE_EPIGENETIC_REGULATION_OF_RRNA_EXPRESSION	
		REACTOME_ACTIVATION_OF_THE_PRE_REPLICATIVE_COMPLEX	
		REACTOME_COOPERATION_OF_PREFOLDIN_AND_TRIC_CCT_IN_ACTIN_AND_TUBULIN_FOLDING	
		WP_GASTRIC_CANCER_NETWORK_2	
		REACTOME_SENESCENCE_ASSOCIATED_SECRETORY_PHENOTYPE_SASP	
		REACTOME_TELOMERE_C_STRAND_LAGGING_STRAND_SYNTHESIS	
		KEGG_BASE_EXCISION_REPAIR	
		WP_COHESIN_COMPLEX_CORNELIA_DE_LANGE_SYNDROME	
		REACTOME_MEIOSIS	
		REACTOME_ACTIVATION_OF_ATR_IN_RESPONSE_TO_REPLICATION_STRESS	
		REACTOME_GAP_JUNCTION_ASSEMBLY	
		REACTOME_RESOLUTION_OF_ABASIC_SITES_AP_SITES	
		WP_DNA_REPAIR_PATHWAYS_FULL_NETWORK	
		REACTOME_DUAL_INCISION_IN_GG_NER	
		KEGG_NUCLEOTIDE_EXCISION_REPAIR	
		REACTOME_AGGREPHAGY	
		REACTOME_ASSEMBLY_AND_CELL_SURFACE_PRESENTATION_OF_NMDA_RECEPTORS	
		WP_NUCLEOTIDE_EXCISION_REPAIR	
		REACTOME_HCMV_EARLY_EVENTS	
		REACTOME_CARBOXYTERMINAL_POST_TRANSLATIONAL_MODIFICATIONS_OF_TUBULIN	
		REACTOME_HOMOLOGY_DIRECTED_REPAIR	
		REACTOME_REPRODUCTION	
		REACTOME_RECYCLING_PATHWAY_OF_L1	
		REACTOME_TP53_REGULATES_TRANSCRIPTION_OF_CELL_CYCLE_GENES	
		WP_MICROTUBULE_CYTOSKELETON_REGULATION	
		REACTOME_EXTENSION_OF_TELOMERES	
		REACTOME_GAP_JUNCTION_TRAFFICKING_AND_REGULATION	
		REACTOME_ESTROGEN_DEPENDENT_GENE_EXPRESSION	
		REACTOME_APOPTOTIC_EXECUTION_PHASE	
		REACTOME_COPI_INDEPENDENT_GOLGI_TO_ER_RETROGRADE_TRAFFIC	
		REACTOME_NUCLEAR_ENVELOPE_BREAKDOWN	
		REACTOME_INTRAFLAGELLAR_TRANSPORT	
		REACTOME_RECOGNITION_AND_ASSOCIATION_OF_DNA_GLYCOSYLASE_WITH_SITE_CONTAINING_AN_AFFECTED_PURINE	
		REACTOME_HSP90_CHAPERONE_CYCLE_FOR_STEROID_HORMONE_RECEPTORS_SHR_IN_THE_PRESENCE_OF_LIGAND	
		REACTOME_HCMV_INFECTION	
		REACTOME_BASE_EXCISION_REPAIR_AP_SITE_FORMATION	
		REACTOME_KINESINS	

Interferon	ICKG_annotation (ranked by pagerank score)	ORA_annotation	LLM_annotation
CXCL10	inducible nitric oxide synthetase	HALLMARK_INTERFERON_GAMMA_RESPONSE	Antiviral Immune Response (0.95)
ISG15	parasite infection control	HALLMARK_INTERFERON_ALPHA_RESPONSE	
GBP1	epithelial ovarian cancer	REACTOME_INTERFERON_SIGNALING	
IFIT3	anticancer therapy	REACTOME_INTERFERON_ALPHA_BETA_SIGNALING	
MX1	anticancer cellular immunotherapy	WP_TYPE_II_INTERFERON_SIGNALING_IFNG	
TNFSF10	macrophage-directed therapy	WP_IMMUNE_RESPONSE_TO_TUBERCULOSIS	
IFIT2	antitumor administration	WP_NETWORK_MAP_OF_SARSCOV2_SIGNALING_PATHWAY	
ISG20	liver disease	WP_SARSCOV2_INNATE_IMMUNITY_EVASION_AND_CELLSPECIFIC_IMMUNE_RESPONSE	
EPSTI1	immune checkpoint inhibitors	REACTOME_INTERFERON_GAMMA_SIGNALING	
GBP5	antitumor immune responses	REACTOME_ANTIVIRAL_MECHANISM_BY_IFN_STIMULATED_GENES	
IFI35	recurrence prediction potential	WP_NONGENOMIC_ACTIONS_OF_125_DIHYDROXYVITAMIN_D3	
IFIT1	enhanced cancer-directed phagocytosis	HALLMARK_INFLAMMATORY_RESPONSE	
IFI44L	anti-tumor immunomodulation	WP_TYPE_I_INTERFERON_INDUCTION_AND_SIGNALING_DURING_SARSCOV2_INFECTION	
STAT1	tolerogenic myeloid cells	WP_OVERVIEW_OF_PROINFLAMMATORY_AND_PROFIBROTIC_MEDIATORS	
CXCL11	tumor sites	WP_PROSTAGLANDIN_SIGNALING	
GBP4	pro-apoptosis effect	KEGG_TOLL_LIKE_RECEPTOR_SIGNALING_PATHWAY	
IL4I1	tumor immunosuppression	WP_TOLLLIKE_RECEPTOR_SIGNALING_PATHWAY	
RSAD2	activated natural killer cells	KEGG_CYTOKINE_CYTOKINE_RECEPTOR_INTERACTION	
IFI6	spontaneous intestinal tumorigenesis	KEGG_CHEMOKINE_SIGNALING_PATHWAY	
IFITM1	tumor necrosis factor	REACTOME_CHEMOKINE_RECEPTORS_BIND_CHEMOKINES	
SAMD9L	tumoricidal factors	HALLMARK_ALLOGRAFT_REJECTION	
UBE2L6	clinical melanoma metastasis suppression	WP_MEASLES_VIRUS_INFECTION	
CXCL9	melanoma cell growth	WP_HOSTPATHOGEN_INTERACTION_OF_HUMAN_CORONAVIRUSES_INTERFERON_INDUCTION	
IFITM3	extracellular matrix remodeling	HALLMARK_IL6_JAK_STAT3_SIGNALING	
OAS1	immune stimulation ability	WP_HOSTPATHOGEN_INTERACTION_OF_HUMAN_CORONAVIRUSES_MAPK_SIGNALING	
TAP1	multiple immune checkpoints	HALLMARK_TNFA_SIGNALING_VIA_NFKB	
VAMP5	tumor immune microenvironment	WP_COVID19_ADVERSE_OUTCOME_PATHWAY	
APOBEC3A	cancer cell survival	WP_NOVEL_INTRACELLULAR_COMPONENTS_OF_RIGILIKE_RECEPTOR_RLR_PATHWAY	
LAP3	acute tubulointerstitial nephritis	KEGG_RIG_I_LIKE_RECEPTOR_SIGNALING_PATHWAY	
WARS	immunotherapy regulation	REACTOME_REGULATION_OF_RUNX2_EXPRESSION_AND_ACTIVITY	
IRF7	aberrant cell proliferation	WP_CYTOSOLIC_DNASENSING_PATHWAY	
PARP14	tumor escape	HALLMARK_APOPTOSIS	
MX2	engineered immune cell-based cancer immunothera	WP_CHEMOKINE_SIGNALING_PATHWAY	
PPA1	efficient cancer immunotherapy	REACTOME_DDX58_IFIH1_MEDIATED_INDUCTION_OF_INTERFERON_ALPHA_BETA	
IDO1	anti-tumor effects	WP_ALLOGRAFT_REJECTION	
LY6E	checkpoint inhibitor pneumonitis	REACTOME_PEPTIDE_LIGAND_BINDING_RECEPTORS	
MT2A	immune-related neutropenia	HALLMARK_IL2_STAT5_SIGNALING	
ANKRD22	longer progression-free survival	HALLMARK_COMPLEMENT	
IFI27	low-risk subgroups	REACTOME_TNFR2_NON_CANONICAL_NF_KB_PATHWAY	
CCL2	cancer burden	REACTOME_TERMINATION_OF_TRANSLESION_DNA_SYNTHESIS	
NT5C3A	myeloid-related inflammatory changes	REACTOME_ANTIGEN_PROCESSING_CROSS_PRESENTATION	
PSME2	endothelial dysfunction		
CCL8	solid tumour cancers		
FAM26F	macrophage-dependent manner		
PSMB9	chemokine receptors		
SERPING1	immune recruitment		
GBP2	antitumor effectiveness		
OAS2	myocardial necrosis		
TNFSF13B	persistent viral replication		
RARRES3	abnormal immune responses		
	poor organ recovery post		
	lung macrophage gene expression		
	macrophage signature genes		

MES_Glycolysi	ICKG_annotation (ranked by pagerank score)	ORA_annotation	LLM_annotation
SPP1	cancer inflammation	HALLMARK_HYPOXIA	Tumor Progression and Metabolic Reprogramming (0.92)
GPNMB	mammary fibrosis	HALLMARK_MTORC1_SIGNALING	
S100A10	antitumor immune responses	WP_GLYCOLYSIS_AND_GLUCONEOGENESIS	
FABP5	tumor microenvironment	REACTOME_METALLOTHIONEINS_BIND_METALS	
CSTB	tumor immunity	REACTOME_RESPONSE_TO_METAL_IONS	
LGALS1	immune checkpoint inhibitors	HALLMARK_GLYCOLYSIS	
S100A6	established metastatic disease	REACTOME_REGULATION_OF_INSULIN_LIKE_GROWTH_FACTOR_IGF_TRANSPORT_AND_UPTAKE_BY_INSULIN_LIKE_GROWTH_FACTOR_BINDING_PROTEINS_IGFBPS	
C15ORF48	tumour progression	HALLMARK_EPITHELIAL_MESENCHYMAL_TRANSITION	
FN1	tolerogenic myeloid cells	HALLMARK_ANGIOGENESIS	
ANXA2	breast cancer brain metastasis	WP_PHOTODYNAMIC_THERAPYINDUCED_HIF1_SURVIVAL_SIGNALING	
PLIN2	interferon-related pathways	WP_ZINC_HOMEOSTASIS	
FBP1	secondary brain tumours	WP_AEROBIC_GLYCOLYSIS	
S100A9	diverse brain tumours	REACTOME_EXTRACELLULAR_MATRIX_ORGANIZATION	
S100A8	extracellular vesicles	WP_MAMMARY_GLAND_DEVELOPMENT_PATHWAY_PUBERTY_STAGE_2_OF_4	
NUPR1	rapamycin complex 1 pathway	WP_PROSTAGLANDIN_SYNTHESIS_AND_REGULATION	
HK2	perihilar large duct type	WP_COPPER_HOMEOSTASIS	
LGALS3	tumour initiation	WP_BURN_WOUND_HEALING	
MIF	early stage tumour development	REACTOME_NEUTROPHIL_DEGRANULATION	
MT1X	multiple immune checkpoints	WP_CCL18_SIGNALING_PATHWAY	
MT2A	durable immune control	WP_VITAMIN_D_RECEPTOR_PATHWAY	
RGCC	phagocyte infiltration	REACTOME_CHAPERONE_MEDIATED_AUTOPHAGY	
S100A4	nonresolving cancer-related inflammation	WP_SPINAL_CORD_INJURY	
ADM	increased tumor survival	KEGG_GLYCOLYSIS_GLUCONEOGENESIS	
BNIP3	tumor heterogeneity	HALLMARK_IL2_STAT5_SIGNALING	
VCAN	immune checkpoint proteins	HALLMARK_COMPLEMENT	
VIM	tumor neutrophils	HALLMARK_COAGULATION	
MT1G	tumor-immune interactions	HALLMARK_CHOLESTEROL_HOMEOSTASIS	
APOC1	fundamental multicellular units	REACTOME_DEGRADATION_OF_THE_EXTRACELLULAR_MATRIX	
MARCO	tumor formation processes	REACTOME_LATE_ENDOSOMAL_MICROAUTOPHAGY	
EMP3	distant metastasis/cell migration	WP_CLEAR_CELL_RENAL_CELL_CARCINOMA_PATHWAYS	
TIMP1	hypoxia-mediated resistance	REACTOME_GENE_AND_PROTEIN_EXPRESSION_BY_JAK_STAT_SIGNALING_AFTER_INTERLEUKIN_12_STIMULATION	
CD9	mediate tumor progression	WP_METABOLIC_REPROGRAMMING_IN_COLON_CANCER	
NDRG1	foster metastasis formation	REACTOME_METABOLISM_OF_CARBOHYDRATES	
ENO1	tumor immunosuppression	REACTOME_INTERLEUKIN_12_SIGNALING	
LDHA	cancer-triggered immune suppression	REACTOME_INTERLEUKIN_4_AND_INTERLEUKIN_13_SIGNALING	
RNASE1	cancer-related death	WP_GLYCOLYSIS_IN_SENESCENCE	
LSP1	cancer-associated fibroblasts	HALLMARK_P53_PATHWAY	
UPP1	ongoing long-term remodelling	REACTOME_DISSOLUTION_OF_FIBRIN_CLOT	
SDC2	conserved wound healing movements	REACTOME_INTERLEUKIN_12_FAMILY_SIGNALING	
SLC2A3	fibroblast activation	REACTOME_SIGNALING_BY_INTERLEUKINS	
CD52	immune microenvironment changes	WP_LUNG_FIBROSIS	
CTSL	efficient efferocytosis	WP_CORI_CYCLE	
SCD	anti-tumor effects	REACTOME_IRAK4_DEFICIENCY_TLR2_4	
ERO1A	critical auto-inflammatory adverse events	WP_TGFBETA_SIGNALING_IN_THYROID_CELLS_FOR_EPITHELIALMESENCHYMAL_TRANSITION	
P4HA1	checkpoint inhibitor pneumonitis	REACTOME_DEFECTIVE_B4GALT7_CAUSES_EDS_PROGEROID_TYPE	
ADAM8	immune-related neutropenia	WP_HEREDITARY_LEIOMYOMATOSIS_AND_RENAL_CELL_CARCINOMA_PATHWAY	
ANXA1	tumor-derived chemokines/cytokines	HALLMARK_FATTY_ACID_METABOLISM	
MT1E	tumor-derived cytokines	REACTOME_CELLULAR_HEXOSE_TRANSPORT	
RALA	heightened infiltration	REACTOME_REGULATION_OF_TLR_BY_ENDOGENOUS_LIGAND	
SLC2A1	cancer-associated inflammation	WP_PI3KAKTMTOR_VITD3_SIGNALING	
	decipher hypoxia-induced pro-tumor immune suppres	REACTOME_RHO_GTPASES_ACTIVATE_NADPH_OXIDASES	
	cancer cell epithelial-mesenchymal transition	KEGG_ECM_RECEPTOR_INTERACTION	
	facilitated tumor metastasis	WP_IL1_AND_MEGAKARYOCYTES_IN_OBESITY	
	long-term immunologic memory	REACTOME_A_TETRASACCHARIDE_LINKER_SEQUENCE_IS_REQUIRED_FOR_GAG_SYNTHESIS	
		REACTOME_SYNDECAN_INTERACTIONS	
		REACTOME_GLUCOSE_METABOLISM	
		REACTOME_DISEASES_OF_IMMUNE_SYSTEM	
		HALLMARK_INFLAMMATORY_RESPONSE	
		HALLMARK_KRAS_SIGNALING_UP	
		REACTOME_ANTIGEN_PROCESSING_CROSS_PRESENTATION	
		KEGG_FRUCTOSE_AND_MANNOSE_METABOLISM	
		REACTOME_GLUCONEOGENESIS	
		REACTOME_SMOOTH_MUSCLE_CONTRACTION	

MAC1	ICKG_annotation (ranked by pagerank score)	ORA_annotation	LLM_annotation
FCN1	tumor microenvironment	REACTOME_NEUTROPHIL_DEGRANULATION	Innate Immune Response and Leukocyte Activation (0.85)
CD52	immune checkpoint inhibitors	WP_COMPLEMENT_SYSTEM	
FCGR3A	established metastatic disease	HALLMARK_COMPLEMENT	
IFITM2	anti-helminth responses	WP_COMPLEMENT_ACTIVATION	
CFP	effective tumor control	REACTOME_RHO_GTPASES_ACTIVATE_NADPH_OXIDASES	
LILRB2	antibody-mediated phagocytosis	WP_COMPLEMENT_SYSTEM_IN_NEURONAL_DEVELOPMENT_AND_PLASTICITY	
LINC01272	antimyeloma activity	HALLMARK_INTERFERON_GAMMA_RESPONSE	
LILRA5	myeloma clearance	REACTOME_COMPLEMENT_CASCADE	
S100A4	cross-talk pathways	REACTOME_RESPONSE_TO_ELEVATED_PLATELET_CYTOSOLIC_CA2	
S100A9	macrophage-mediated phagocytosis	WP_COMPLEMENT_AND_COAGULATION_CASCADES	
SERPINA1	tumor infiltration	HALLMARK_COAGULATION	
TIMP1	follicular lymphoma	WP_LUNG_FIBROSIS	
WARS	macrophage phagocytosis	REACTOME_PLATELET_ACTIVATION_SIGNALING_AND_AGGREGATION	
IFITM3	innate immune defense	WP_AMPLIFICATION_AND_EXPANSION_OF_ONCOGENIC_PATHWAYS_AS_METASTATIC_TRAITS	
S100A8	transient peripheral transcriptomic alterations	KEGG_COMPLEMENT_AND_COAGULATION_CASCADES	
CORO1A	improved long-term systemic antitumor immunit	REACTOME_FCGR_ACTIVATION	
PLAC8	murine peritoneal macrophage differentiation	REACTOME_IRAK4_DEFICIENCY_TLR2_4	
STXBP2	dependent cell phagocytosis	REACTOME_REGULATION_OF_TLR_BY_ENDOGENOUS_LIGAND	
CFD	protective anti-tumor immune responses	REACTOME_INITIAL_TRIGGERING_OF_COMPLEMENT	
LST1	coronavirus-induced lung inflammation	WP_VITAMIN_D_RECEPTOR_PATHWAY	
CYTIP	immune resistance	WP_IL1_AND_MEGAKARYOCYTES_IN_OBESITY	
RHOC	virus reactivation	REACTOME_IMMUNOREGULATORY_INTERACTIONS_BETWEEN_A_LYMPHOID_AND_A_NON_LYMPHOID_CELL	
FGR	tumor progress	REACTOME_FCGR3A_MEDIATED_IL10_SYNTHESIS	
TCF7L2	greater pathogenesis	KEGG_FC_GAMMA_R_MEDIATED_PHAGOCYTOSIS	
CLEC12A	cutaneous melanoma	HALLMARK_HYPOXIA	
COTL1	cancer-related inflammation	HALLMARK_INFLAMMATORY_RESPONSE	
NAAA	anti-tumor effects	HALLMARK_INTERFERON_ALPHA_RESPONSE	
POU2F2	optimal combination regimens	REACTOME_DISEASES_OF_IMMUNE_SYSTEM	
ABI3	critical auto-inflammatory adverse events	HALLMARK_NOTCH_SIGNALING	
BCL2A1	checkpoint inhibitor pneumonitis	WP_GASTRIC_CANCER_NETWORK_2	
CD48	immune-related neutropenia	REACTOME_PARASITE_INFECTION	
CDKN1C	lymphovascular invasion	HALLMARK_ANGIOGENESIS	
LILRA1	pancreatic cancer burden	WP_HOSTPATHOGEN_INTERACTION_OF_HUMAN_CORONAVIRUSES_MAPK_SIGNALING	
LRRC25	viral pathogenesis		
LYST	somatic hypermutation		
MYO1G	cancer-induced immunosuppression		
RAC2	delayed tumor growth		
SPN	reduced drug accumulation		
CD55	combination chemotherapy		
CRIP1	intratumoral dynamics		
APOBEC3A	brain tumor outgrowth		
HES1	colitis-induced colon tumors		
LTA4H	pro-inflammatory switch		
LY6E	increased onco-toxicity		
MAFB	tumorigenesis capabilities		
MS4A7	myeloid reconstitution		
MT2A	improved reconstitution		
MTSS1	pathogenic envelope viruses		
HCK	gastric cancer pathogenesis		
VAMP5	gastric cancer immunotherapy		
	perihilar large duct type		
	macrophage-driven mechanisms		
	intratumoral redistribution		
	immune system homeostasis		
	antibody-dependent cellular cytotoxicity		
	phagocytosis enabling escape		
	additional antibody-dependent cellular phagocytosis		
	strong antitumor activity		
	increased leukocyte infiltration		
	co-mutated lung adenocarcinoma		
	tumoral chemoresistance		
	immune deregulation		

Stress_HSP	ICKG_annotation (ranked by pagerank score)	ORA_annotation	LLM_annotation
JUN	epithelial ovarian cancer	HALLMARK_TNFA_SIGNALING_VIA_NFKB	Stress Response and Protein Folding (0.92)
DNAJB1	cancer progression	REACTOME_ATTENUATION_PHASE	
HSPA1B	cancer-related genetic expression	REACTOME_HSF1_DEPENDENT_TRANSACTIVATION	
HSPA1A	tumour progression	REACTOME_HSF1_ACTIVATION	
FOS	antigen presentation	REACTOME_CELLULAR_RESPONSE_TO_HEAT_STRESS	
GADD45B	tumour initiation	REACTOME_HSP90_CHAPERONE_CYCLE_FOR_STEROID_HORMONE_RECEPTORS_SHR_IN_THE_PRESENCE_OF_LIGAND	
HSPH1	recurrence prediction potential	REACTOME_REGULATION_OF_HSF1_MEDIATED_HEAT_SHOCK_RESPONSE	
HSPA6	distant metastasis/cell migration	HALLMARK_UV_RESPONSE_UP	
RGS2	hypoxia-mediated resistance	KEGG_MAPK_SIGNALING_PATHWAY	
HSPB1	mediate tumor progression	HALLMARK_HYPOXIA	
IER2	anti-phagocytic receptors	WP_OREXIN_RECEPTOR_PATHWAY	
RHOB	immune cell trafficking regulation	WP_NETWORK_MAP_OF_SARSCOV2_SIGNALING_PATHWAY	
ATF3	pro-tumor behavior	HALLMARK_APOPTOSIS	
BAG3	cancer immunosurveillance	WP_NUCLEAR_RECEPTORS_METAPATHWAY	
EGR1	autochthonous immune response	WP_MAPK_SIGNALING_PATHWAY	
HSPD1	cell-cycle control	REACTOME_NGF_STIMULATED_TRANSCRIPTION	
HSP90AA1	foster metastasis formation	HALLMARK_P53_PATHWAY	
DNAJA4	tumor destruction	WP_IL18_SIGNALING_PATHWAY	
FOSB	tumor mass	KEGG_ANTIGEN_PROCESSING_AND_PRESENTATION	
NR4A1	enhanced immune cell recruitment	WP_CORTICOTROPINRELEASING_HORMONE_SIGNALING_PATHWAY	
JUNB	cancer-triggered immune suppression	REACTOME_CHAPERONE_MEDIATED_AUTOPHAGY	
DNAJA1	cancer-related death	WP_PREIMPLANTATION_EMBRYO	
KLF6	myeloid cell metabolism	REACTOME_NUCLEAR_EVENTS_KINASE_AND_TRANSCRIPTION_FACTOR_ACTIVATION	
SGK1	myeloid cell function	WP_SPINAL_CORD_INJURY	
DUSP1	efficient efferocytosis	WP_GLUCOCORTICOID_RECEPTOR_PATHWAY	
IER5	anti-tumor phenotype	HALLMARK_MTORC1_SIGNALING	
MAFB	intestinal adaptation	WP_TGFBETA_SIGNALING_PATHWAY	
RGS1	decipher hypoxia-induced pro-tumor immune suppres	WP_MEASLES_VIRUS_INFECTION	
ZFP36L2	clonal expansion	REACTOME_ESR_MEDIATED_SIGNALING	
KLF2	tumor-derived chemokines/cytokines	REACTOME_ESTROGEN_DEPENDENT_GENE_EXPRESSION	
HSP90AB1	tumor-derived cytokines	REACTOME_REGULATION_OF_MRNA_STABILITY_BY_PROTEINS_THAT_BIND_AU_RICH_ELEMENTS	
HSPE1	human myeloma cell growth	WP_APOPTOSIS_MODULATION_AND_SIGNALING	
KLF4	cognitive function	WP_VEGFAVEGFR2_SIGNALING_PATHWAY	
TSC22D3	cancer immunology	WP_PHOTODYNAMIC_THERAPYINDUCED_AP1_SURVIVAL_SIGNALING	
ZFAND2A	aberrant angiogenesis	REACTOME_AUF1_HNRNP_D0_BINDS_AND_DESTABILIZES_MRNA	
CCL3	onco-fetal ecosystem	WP_GASTRIN_SIGNALING_PATHWAY	
HSPA8	thrombus formation	REACTOME_SIGNALING_BY_NUCLEAR_RECEPTORS	
CCL4	local cellular clustering	WP_PARKINUBIQUITIN_PROTEASOMAL_SYSTEM_PATHWAY	
CLK1	fibroblast activation	REACTOME_SIGNALING_BY_NTRKS	
DDIT4	tumor regulatory mechanisms	WP_CHROMOSOMAL_AND_MICROSATELLITE_INSTABILITY_IN_COLORECTAL_CANCER	
NR4A2	cancer cell epithelial-mesenchymal transition	WP_SELECTIVE_EXPRESSION_OF_CHEMOKINE_RECEPTORS_DURING_TCELL_POLARIZATION	
PPP1R15A	facilitated tumor metastasis	WP_NRF2_PATHWAY	
UBC	pro-fibrotic activities	REACTOME_SIGNALING_BY_INTERLEUKINS	
BTG2	liver regeneration	WP_MYOMETRIAL_RELAXATION_AND_CONTRACTION_PATHWAYS	
CACYBP	high-grade serous ovarian cancer	WP_HOSTPATHOGEN_INTERACTION_OF_HUMAN_CORONAVIRUSES_MAPK_SIGNALING	
CXCR4	anti-glioma activity	REACTOME_TRANSCRIPTIONAL_REGULATION_BY_TP53	
ZFP36	advanced high-grade serous ovarian cancer	REACTOME_MAPK6_MAPK4_SIGNALING	
MALAT1	local valvular inflammation	KEGG_ENDOCYTOSIS	
FKBP4	melanoma cell growth	KEGG_TOLL_LIKE_RECEPTOR_SIGNALING_PATHWAY	
PMAIP1		WP_TOLLLIKE_RECEPTOR_SIGNALING_PATHWAY	
		REACTOME_ACTIVATION_OF_THE_AP_1_FAMILY_OF_TRANSCRIPTION_FACTORS	
		REACTOME_INTERLEUKIN_4_AND_INTERLEUKIN_13_SIGNALING	
		WP_ARYL_HYDROCARBON_RECEPTOR_PATHWAY_WP2873	
		HALLMARK_UNFOLDED_PROTEIN_RESPONSE	
		HALLMARK_ESTROGEN_RESPONSE_LATE	
		WP_NEUROINFLAMMATION	
		KEGG_SPLICEOSOME	
		REACTOME_CHEMOKINE_RECEPTORS_BIND_CHEMOKINES	
		REACTOME_RESPONSE_OF_EIF2AK1_HRI_TO_HEME_DEFICIENCY	
		WP_LET7_INHIBITION_OF_ES_CELL_REPROGRAMMING	
		WP_HEMATOPOIETIC_STEM_CELL_DIFFERENTIATION	
		REACTOME_SEMA3A_PAK_DEPENDENT_AXON_REPULSION	
		WP_MAPK_PATHWAY_IN_CONGENITAL_THYROID_CANCER	
		WP_QUERCETIN_AND_NFKB_AP1_INDUCED_APOPTOSIS	
		REACTOME_SEMAPHORIN_INTERACTIONS	
		WP_ONCOSTATIN_M_SIGNALING_PATHWAY	
		WP_THYROID_STIMULATING_HORMONE_TSH_SIGNALING_PATHWAY	
		REACTOME_AUTOPHAGY	
		REACTOME_CONSTITUTIVE_SIGNALING_BY_LIGAND_RESPONSIVE_EGFR_CANCER_VARIANTS	
		REACTOME_INTERLEUKIN_17_SIGNALING	
		REACTOME_SARS_COV_INFECTIONS	
		REACTOME_EXTRA_NUCLEAR_ESTROGEN_SIGNALING	
		REACTOME_SIGNALING_BY_TGF_BETA_RECEPTOR_COMPLEX	
		WP_INSULIN_SIGNALING	
		REACTOME_THE_ROLE_OF_GTSE1_IN_G2_M_PROGRESSION_AFTER_G2_CHECKPOINT	
		REACTOME_RHOBTB2_GTPASE_CYCLE	
		WP_ESTROGEN_SIGNALING_PATHWAY	
		WP_PHOTODYNAMIC_THERAPYINDUCED_NFE2L2_NRF2_SURVIVAL_SIGNALING	
		REACTOME_DDX58_IFIH1_MEDIATED_INDUCTION_OF_INTERFERON_ALPHA_BETA	
		REACTOME_SELECTIVE_AUTOPHAGY	
		REACTOME_SIGNALING_BY_EGFR_IN_CANCER	
		WP_PHYSIOLOGICAL_AND_PATHOLOGICAL_HYPERTROPHY_OF_THE_HEART	
		WP_UNFOLDED_PROTEIN_RESPONSE	
		REACTOME_POTENTIAL_THERAPEUTICS_FOR_SARS	
		REACTOME_DOWNREGULATION_OF_TGF_BETA_RECEPTOR_SIGNALING	
		WP_HAIR_FOLLICLE_DEVELOPMENT_CYTODIFFERENTIATION_PART_3_OF_3	
		WP_ANDROGEN_RECEPTOR_SIGNALING_PATHWAY	
		WP_INTERACTIONS_OF_NATURAL_KILLER_CELLS_IN_PANCREATIC_CANCER	
		REACTOME_TOLL_LIKE_RECEPTOR_9_TLR9_CASCADE	
		REACTOME_DOWNREGULATION_OF_ERBB2_SIGNALING	
		REACTOME_RIPK1_MEDIATED_REGULATED_NECROSIS	
		REACTOME_UPTAKE_AND_ACTIONS_OF_BACTERIAL_TOXINS	
		WP_PDGFRBETA_PATHWAY	
		WP_PHOTODYNAMIC_THERAPYINDUCED_UNFOLDED_PROTEIN_RESPONSE	
		REACTOME_MYD88_INDEPENDENT_TLR4_CASCADE	
		REACTOME_MAPK_TARGETS_NUCLEAR_EVENTS_MEDIATED_BY_MAP_KINASES	
		HALLMARK_ESTROGEN_RESPONSE_EARLY	
		HALLMARK_KRAS_SIGNALING_UP	
		REACTOME_SMAD2_SMAD3_SMAD4_HETEROTRIMER_REGULATES_TRANSCRIPTION	
		REACTOME_TGF_BETA_RECEPTOR_SIGNALING_ACTIVATES_SMADS	
		REACTOME_TOLL_LIKE_RECEPTOR_TLR1_TLR2_CASCADE	
		REACTOME_MAPK_FAMILY_SIGNALING_CASCADES	
		REACTOME_SIGNALING_BY_TGFB_FAMILY_MEMBERS	
		WP_HOSTPATHOGEN_INTERACTION_OF_HUMAN_CORONAVIRUSES_INTERFERON_INDUCTION	
		WP_WHITE_FAT_CELL_DIFFERENTIATION	
		REACTOME_STIMULI_SENSING_CHANNELS	
		REACTOME_LATE_ENDOSOMAL_MICROAUTOPHAGY	
		WP_HEPATOCYTE_GROWTH_FACTOR_RECEPTOR_SIGNALING	
		WP_OXIDATIVE_STRESS_RESPONSE	
		REACTOME_NEUTROPHIL_DEGRANULATION	
		KEGG_PRION_DISEASES	
		REACTOME_RHOBTB_GTPASE_CYCLE	
		REACTOME_SENESCENCE_ASSOCIATED_SECRETORY_PHENOTYPE_SASP	
		REACTOME_REGULATION_OF_TP53_EXPRESSION_AND_DEGRADATION	
		WP_NUCLEAR_RECEPTORS	
		WP_IL5_SIGNALING_PATHWAY	
		WP_PDGF_PATHWAY	

MAC2	CKG_annotation (ranked by pagerank score	ORA_annotation	LLM_annotation
FABP4	tumor growth	WP_PPAR_SIGNALING_PATHWAY	Inflammatory Response and Lipid Metabolism (0.85)
MARCO	tumor initiation	KEGG_PPAR_SIGNALING_PATHWAY	
RBP4	disease progression	HALLMARK_XENOBIOTIC_METABOLISM	
PCOLCE2	autoimmune destruction	REACTOME_RESPONSE_TO_ELEVATED_PLATELET_CYTOSOLIC_CA2	
INHBA	pro-inflammatory properties	HALLMARK_COAGULATION	
CES1	cell autoimmunity	WP_STEROL_REGULATORY_ELEMENTBINDING_PROTEINS_SREBP_SIGNALING	
CCL18	inflammatory bowel disease	WP_FAMILIAL_PARTIAL_LIPODYSTROPHY_FPLD	
CD52	obesity-associated breast cancer developme	REACTOME_FATTY_ACID_METABOLISM	
FN1	multiple reactive microglial states	HALLMARK_ADIPOGENESIS	
GCHFR	discrete myeloid cell phenotypes	HALLMARK_COMPLEMENT	
LPL	brain development	HALLMARK_EPITHELIAL_MESENCHYMAL_TRANSITION	
MCEMP1	brain homeostasis	REACTOME_METABOLISM_OF_FAT_SOLUBLE_VITAMINS	
PHLDA3	rapamycin complex 1 pathway	WP_ADIPOGENESIS	
PPARG	tumor-induced immunosuppression	REACTOME_SYNTHESIS_OF_BILE_ACIDS_AND_BILE_SALTS_VIA_24_HYDROXYCHOLESTEROL	
IFI27	inflammation-related cancer	REACTOME_ARACHIDONIC_ACID_METABOLISM	
NUPR1	tumor expansion	REACTOME_SYNTHESIS_OF_BILE_ACIDS_AND_BILE_SALTS_VIA_27_HYDROXYCHOLESTEROL	
S100A13	ulcerative colitis	REACTOME_PLATELET_ACTIVATION_SIGNALING_AND_AGGREGATION	
SERPING1	stromal-myeloid interactions	REACTOME_BIOSYNTHESIS_OF_SPECIALIZED_PRORESOLVING_MEDIATORS_SPMS	
ALDH2	cellular antiviral status	HALLMARK_CHOLESTEROL_HOMEOSTASIS	
GYPC	critical pro-tumoral roles	REACTOME_SYNTHESIS_OF_LEUKOTRIENES_LT_AND_EOXINS_EX	
LGALS3BP	compartment-specific immunological respon	KEGG_BIOSYNTHESIS_OF_UNSATURATED_FATTY_ACIDS	
SCD	spatial heterogeneity	REACTOME_TRANSCRIPTIONAL_REGULATION_OF_WHITE_ADIPOCYTE_DIFFERENTIATION	
ACOT7	scavenger receptors	REACTOME_SYNTHESIS_OF_BILE_ACIDS_AND_BILE_SALTS_VIA_7ALPHA_HYDROXYCHOLESTEROL	
CYP27A1	recruited myeloid cells	WP_EICOSANOID_SYNTHESIS	
LTA4H	lung cancer cell proliferation	WP_TRIACYLGLYCERIDE_SYNTHESIS	
NMB	intestinal inflammation	REACTOME_PEPTIDE_HORMONE_METABOLISM	
PDLIM1	tumor cell invasion	REACTOME_METABOLISM_OF_VITAMINS_AND_COFACTORS	
AQP3	anti-tumoral immunity	WP_7OXOC_AND_7BETAHC_PATHWAYS	
FBP1	bone-wasting diseases	WP_NUCLEAR_RECEPTORS_METAPATHWAY	
MGST1	cancer-promoting inflammation	KEGG_THYROID_CANCER	
PPIC	adaptive anti-tumor immunity	HALLMARK_KRAS_SIGNALING_UP	
RETN	br-met outgrowth	REACTOME_VISUAL_PHOTOTRANSDUCTION	
RP5-839B4.8	myeloid cell polarization	REACTOME_FOXO_MEDIATED_TRANSCRIPTION_OF_OXIDATIVE_STRESS_METABOLIC_AND_NEURONAL_GENES	
VMO1	adaptive/acquired resistance	WP_PROSTAGLANDIN_AND_LEUKOTRIENE_METABOLISM_IN_SENESCENCE	
ALOX5AP	macrophage-ferroptosis crosstalk	REACTOME_PHASE_I_FUNCTIONALIZATION_OF_COMPOUNDS	
GPD1	immune checkpoint expression		
SERPINA1	cancer cell biology		
TCF7L2	leukocyte migration		
FAM89A			
FHL1			
IGFBP2			
PLA2G16			
RND3			
STXBP2			
TGM2			
TREM1			
AGRP			
AKR1C3			
UBASH3B			
HDDC2			

Proteasomal-degradatio	KG_annotation (ranked by pagerank scor	ORA_annotation	LLM_annotation
NME1	tumor immunity	REACTOME_ANTIGEN_PROCESSING_CROSS_PRESENTATION	Immune Response and Protein Folding (0.85)
FABP5	cancer immunosurveillance	REACTOME_CROSS_PRESENTATION_OF_SOLUBLE_EXOGENOUS_ANTIGENS_ENDOSOMES	
HSPA5	autochthonous immune response	REACTOME_THE_ROLE_OF_GTSE1_IN_G2_M_PROGRESSION_AFTER_G2_CHECKPOINT	
CCT5	immune checkpoint inhibitors	REACTOME_REGULATION_OF_HMOX1_EXPRESSION_AND_ACTIVITY	
MANF	established metastatic disease	KEGG_PROTEASOME	
SDF2L1	tumor microenvironment	REACTOME_HEDGEHOG_OFF_STATE	
PRDX1	tumour progression	REACTOME_NEGATIVE_REGULATION_OF_NOTCH4_SIGNALING	
PSMA4	breast cancer brain metastasis	REACTOME_AUF1_HNRNP_D0_BINDS_AND_DESTABILIZES_MRNA	
SPP1	primary myelofibrosis	REACTOME_DEGRADATION_OF_AXIN	
ATP6V0D1	interferon-related pathways	REACTOME_REGULATION_OF_RUNX3_EXPRESSION_AND_ACTIVITY	
FBP1	secondary brain tumours	REACTOME_DEGRADATION_OF_DVL	
MDH1	diverse brain tumours	REACTOME_STABILIZATION_OF_P53	
VSIG4	perihilar large duct type	REACTOME_CDT1_ASSOCIATION_WITH_THE_CDC6_ORC_ORIGIN_COMPLEX	
CALR	liver disease	REACTOME_METABOLISM_OF_POLYAMINES	
MYDGF	tumor promotion	REACTOME_DEGRADATION_OF_GLI1_BY_THE_PROTEASOME	
PDIA6	cancer-microenvironment interactions	REACTOME_SCF_SKP2_MEDIATED_DEGRADATION_OF_P27_P21	
PSMD8	multiple reactive microglial states	REACTOME_DEFECTIVE_CFTR_CAUSES_CYSTIC_FIBROSIS	
SLC3A2	discrete myeloid cell phenotypes	REACTOME_DECTIN_1_MEDIATED_NONCANONICAL_NF_KB_SIGNALING	
TREM2	cognitive function	REACTOME_ASYMMETRIC_LOCALIZATION_OF_PCP_PROTEINS	
LGALS3	anti-inflammatory responses	WP_PROTEASOME_DEGRADATION	
LRPAP1	proinflammatory polarization	REACTOME_TRANSCRIPTIONAL_REGULATION_BY_RUNX3	
MARCO	immune cell recruitment	REACTOME_HEDGEHOG_LIGAND_BIOGENESIS	
PSMA3	tumor-immune interactions	REACTOME_G1_S_DNA_DAMAGE_CHECKPOINTS	
PSMD7	tumor sites	REACTOME_REGULATION_OF_RAS_BY_GAPS	
PSME2	enhanced bacterial capture ability	REACTOME_REGULATION_OF_PTEN_STABILITY_AND_ACTIVITY	
S100A8	spontaneous intestinal tumorigenesis	REACTOME_CLASS_I_MHC_MEDIATED_ANTIGEN_PROCESSING_PRESENTATION	
CTSL	cancer cell survival	REACTOME_ORC1_REMOVAL_FROM_CHROMATIN	
CYP27A1	distant metastasis/cell migration	REACTOME_REGULATION_OF_RUNX2_EXPRESSION_AND_ACTIVITY	
GCHFR	hypoxia-mediated resistance	REACTOME_APC_C_CDH1_MEDIATED_DEGRADATION_OF_CDC20_AND_OTHER_APC_C_CDH1_TARGETED_PROTEINS_IN_LATE_MITOSIS_EARLY_G1	
MNDA	mediate tumor progression	REACTOME_SIGNALING_BY_HEDGEHOG	
PSMB6	direct cytolytic infection	REACTOME_CELLULAR_RESPONSE_TO_HYPOXIA	
TUBA1B	foster metastasis formation	REACTOME_ABC_TRANSPORTER_DISORDERS	
DNASE2	kinase activity	REACTOME_CELLULAR_RESPONSE_TO_CHEMICAL_STRESS	
FCGR1A	br-met outgrowth	REACTOME_DOWNSTREAM_SIGNALING_EVENTS_OF_B_CELL_RECEPTOR_BCR	
HM13	optimal combination regimens	REACTOME_SIGNALING_BY_NOTCH4	
LTA4H	antibody-mediated phagocytosis	REACTOME_TRANSCRIPTIONAL_REGULATION_BY_RUNX2	
MGST3	anti-tumor effects	REACTOME_CYCLIN_A_CDK2_ASSOCIATED_EVENTS_AT_S_PHASE_ENTRY	
PSMA5	critical auto-inflammatory adverse events	REACTOME_DEGRADATION_OF_BETA_CATENIN_BY_THE_DESTRUCTION_COMPLEX	
PSMC2	cutaneous melanoma	REACTOME_CYTOPROTECTION_BY_HMOX1	
PSMC3	glioma development	REACTOME_HEDGEHOG_ON_STATE	
RENBP	checkpoint inhibitor pneumonitis	REACTOME_REGULATION_OF_MRNA_STABILITY_BY_PROTEINS_THAT_BIND_AU_RICH_ELEMENTS	
PLA2G7	immune-related neutropenia	REACTOME_APC_C_MEDIATED_DEGRADATION_OF_CELL_CYCLE_PROTEINS	
CCL2	strong tumor angiogenesis	REACTOME_SWITCHING_OF_ORIGINS_TO_A_POST_REPLICATIVE_STATE	
FPR1	disseminated tumor cells	REACTOME_PCP_CE_PATHWAY	
S100A9	immunotherapy-insensitive tumors	REACTOME_MAPK6_MAPK4_SIGNALING	
GLIPR2	dependent cell phagocytosis	REACTOME_SEPARATION_OF_SISTER_CHROMATIDS	
NAGK	lesional efferocytosis	REACTOME_CLEC7A_DECTIN_1_SIGNALING	
TUBA1A	chemokine receptors	REACTOME_UCH_PROTEINASES	
HSP90B1	disease-associated denervation	REACTOME_TNFR2_NON_CANONICAL_NF_KB_PATHWAY	
CD9	nonresolving cancer-related inflammatior	REACTOME_INTERLEUKIN_1_SIGNALING	
	increased tumor survival	REACTOME_ABC_FAMILY_PROTEINS_MEDIATED_TRANSPORT	
	immune cell-associated diseases	REACTOME_MITOTIC_G2_G2_M_PHASES	
	increased leukocyte infiltration	REACTOME_SYNTHESIS_OF_DNA	
	heightened infiltration	REACTOME_TCR_SIGNALING	
		REACTOME_MITOTIC_METAPHASE_AND_ANAPHASE	
		REACTOME_DISORDERS_OF_TRANSMEMBRANE_TRANSPORTERS	
		REACTOME_TRANSCRIPTIONAL_REGULATION_BY_RUNX1	
		REACTOME_RUNX1_REGULATES_TRANSCRIPTION_OF_GENES_INVOLVED_IN_DIFFERENTIATION_OF_HSCS	
		REACTOME_HOST_INTERACTIONS_OF_HIV_FACTORS	
		REACTOME_FCERI_MEDIATED_NF_KB_ACTIVATION	
		REACTOME_PTEN_REGULATION	
		REACTOME_C_TYPE_LECTIN_RECEPTORS_CLRS	
		REACTOME_INTERLEUKIN_1_FAMILY_SIGNALING	
		HALLMARK_MTORC1_SIGNALING	
		REACTOME_BETA_CATENIN_INDEPENDENT_WNT_SIGNALING	
		REACTOME_DNA_REPLICATION_PRE_INITIATION	
		REACTOME_MITOTIC_G1_PHASE_AND_G1_S_TRANSITION	
		WP_PARKINUBIQUITIN_PROTEASOMAL_SYSTEM_PATHWAY	
		REACTOME_S_PHASE	
		REACTOME_SIGNALING_BY_THE_B_CELL_RECEPTOR_BCR	
		REACTOME_G2_M_CHECKPOINTS	
		REACTOME_REGULATION_OF_EXPRESSION_OF_SLITS_AND_ROBOS	
		REACTOME_APOPTOSIS	
		REACTOME_FC_EPSILON_RECEPTOR_FCERI_SIGNALING	
		REACTOME_DNA_REPLICATION	
		WP_ALZHEIMERS_DISEASE	
		REACTOME_UNFOLDED_PROTEIN_RESPONSE_UPR	
		REACTOME_PROGRAMMED_CELL_DEATH	
		REACTOME_SIGNALING_BY_INTERLEUKINS	
		REACTOME_SIGNALING_BY_ROBO_RECEPTORS	
		REACTOME_UB_SPECIFIC_PROCESSING_PROTEASES	
		REACTOME_TCF_DEPENDENT_SIGNALING_IN_RESPONSE_TO_WNT	
		REACTOME_HIV_INFECTION	
		REACTOME_NEDDYLATION	
		REACTOME_SIGNALING_BY_NOTCH	
		REACTOME_M_PHASE	
		WP_ALZHEIMERS_DISEASE_AND_MIRNA_EFFECTS	
		REACTOME_METABOLISM_OF_AMINO_ACIDS_AND_DERIVATIVES	
		REACTOME_CELL_CYCLE_CHECKPOINTS	
		REACTOME_NEUTROPHIL_DEGRANULATION	
		REACTOME_DEUBIQUITINATION	
		REACTOME_INTRACELLULAR_SIGNALING_BY_SECOND_MESSENGERS	
		REACTOME_ANTIGEN_PROCESSING_UBIQUITINATION_PROTEASOME_DEGRADATION	
		REACTOME_MAPK_FAMILY_SIGNALING_CASCADES	
		REACTOME_SIGNALING_BY_WNT	
		HALLMARK_UNFOLDED_PROTEIN_RESPONSE	
		WP_PHOTODYNAMIC_THERAPYINDUCED_UNFOLDED_PROTEIN_RESPONSE	
		REACTOME_ATF6_ATF6_ALPHA_ACTIVATES_CHAPERONE_GENES	
		REACTOME_ATF6_ATF6_ALPHA_ACTIVATES_CHAPERONES	
		REACTOME_DISEASES_OF_SIGNAL_TRANSDUCTION_BY_GROWTH_FACTOR_RECEPTORS_AND_SECOND_MESSENGERS	
		REACTOME_IRE1ALPHA_ACTIVATES_CHAPERONES	
		REACTOME_SCAVENGING_BY_CLASS_A_RECEPTORS	
		HALLMARK_COMPLEMENT	
		WP_IL1_AND_MEGAKARYOCYTES_IN_OBESITY	
		REACTOME_FORMATION_OF_TUBULIN_FOLDING_INTERMEDIATES_BY_CCT_TRIC	
		REACTOME_COOPERATION_OF_PREFOLDIN_AND_TRIC_CCT_IN_ACTIN_AND_TUBULIN_FOLDING	
		KEGG_ANTIGEN_PROCESSING_AND_PRESENTATION	
		WP_COMPLEMENT_SYSTEM	
		HALLMARK_INTERFERON_GAMMA_RESPONSE	
		HALLMARK_MYC_TARGETS_V1	
		REACTOME_TRAFFICKING_AND_PROCESSING_OF_ENDOSOMAL_TLR	
		REACTOME_IRAK4_DEFICIENCY_TLR2_4	
		REACTOME_TOLL_LIKE_RECEPTOR_CASCADES	
		HALLMARK_CHOLESTEROL_HOMEOSTASIS	
		REACTOME_REGULATION_OF_TLR_BY_ENDOGENOUS_LIGAND	
		REACTOME_TRANSPORT_OF_CONNEXONS_TO_THE_PLASMA_MEMBRANE	
		REACTOME_POST_CHAPERONIN_TUBULIN_FOLDING_PATHWAY	
		REACTOME_RHO_GTPASES_ACTIVATE_NADPH_OXIDASES	
		REACTOME_ANTIGEN_PRESENTATION_FOLDING_ASSEMBLY_AND_PEPTIDE_LOADING_OF_CLASS_I_MHC	
		WP_VITAMIN_D_RECEPTOR_PATHWAY	
		REACTOME_ACTIVATION_OF_AMPK_DOWNSTREAM_OF_NMDARS	
		REACTOME_ASPARAGINE_N_LINKED_GLYCOSYLATION	
		REACTOME_BINDING_AND_UPTAKE_OF_LIGANDS_BY_SCAVENGER_RECEPTORS	
		REACTOME_PROTEIN_FOLDING	
		REACTOME_DISEASES_OF_IMMUNE_SYSTEM	
		REACTOME_PERK_REGULATES_GENE_EXPRESSION	
		REACTOME_RHO_GTPASES_ACTIVATE_IQGAPS	
		REACTOME_SEALING_OF_THE_NUCLEAR_ENVELOPE_NE_BY_ESCRT_III	
		REACTOME_GLUCONEOGENESIS	
		HALLMARK_ANGIOGENESIS	
		WP_PRION_DISEASE_PATHWAY	
		REACTOME_GAP_JUNCTION_ASSEMBLY	
		WP_MICROGLIA_PATHOGEN_PHAGOCYTOSIS_PATHWAY	
		KEGG_LYSOSOME	
		REACTOME_REGULATION_OF_INSULIN_LIKE_GROWTH_FACTOR_IGF_TRANSPORT_AND_UPTAKE_BY_INSULIN_LIKE_GROWTH_FACTOR_BINDING_PROTEINS_IGFBPS	
		REACTOME_MHC_CLASS_II_ANTIGEN_PRESENTATION	
		KEGG_AMINO_SUGAR_AND_NUCLEOTIDE_SUGAR_METABOLISM	
		REACTOME_AGGREPHAGY	
		REACTOME_ASSEMBLY_AND_CELL_SURFACE_PRESENTATION_OF_NMDA_RECEPTORS	
		WP_GLYCOLYSIS_AND_GLUCONEOGENESIS	
		REACTOME_CARBOXYTERMINAL_POST_TRANSLATIONAL_MODIFICATIONS_OF_TUBULIN	
		REACTOME_INTERLEUKIN_10_SIGNALING	
		REACTOME_RESPONSE_TO_ELEVATED_PLATELET_CYTOSOLIC_CA2	
		REACTOME_RECYCLING_PATHWAY_OF_L1	
		REACTOME_GAP_JUNCTION_TRAFFICKING_AND_REGULATION	
		REACTOME_COPI_INDEPENDENT_GOLGI_TO_ER_RETROGRADE_TRAFFIC	

MAC3	ICKG_annotation (ranked by pagerank score)	ORA_annotation	LLM_annotation
C3	gastric adenocarcinoma	KEGG_CYTOKINE_CYTOKINE_RECEPTOR_INTERACTION	Immune Response and Inflammation Regulation (0.85)
OLFML3	recovery-like disease	WP_ALLOGRAFT_REJECTION	
C1QC	tumor microenvironment	WP_COMPLEMENT_ACTIVATION	
C1QB	reduced pro-viral gene expression	REACTOME_CHEMOKINE_RECEPTORS_BIND_CHEMOKINES	
PLD4	immune tolerance	WP_COMPLEMENT_SYSTEM_IN_NEURONAL_DEVELOPMENT_AND_PLASTICITY	
FCGBP	innate immune defense	WP_SELECTIVE_EXPRESSION_OF_CHEMOKINE_RECEPTORS_DURING_TCELL_POLARIZATION	
GPR34	distant metastasis/cell migration	KEGG_COMPLEMENT_AND_COAGULATION_CASCADES	
LPAR6	hypoxia-mediated resistance	HALLMARK_INFLAMMATORY_RESPONSE	
CCL3	mediate tumor progression	KEGG_LEISHMANIA_INFECTION	
CCL4	liver regeneration	KEGG_PRION_DISEASES	
C1QA	anti-phagocytic receptors	KEGG_SYSTEMIC_LUPUS_ERYTHEMATOSUS	
LTC4S	immune cell trafficking regulation	WP_MICROGLIA_PATHOGEN_PHAGOCYTOSIS_PATHWAY	
RGS1	pro-tumor behavior	HALLMARK_IL6_JAK_STAT3_SIGNALING	
ALOX5AP	foster metastasis formation	REACTOME_INTERLEUKIN_10_SIGNALING	
AXL	anti-microbial responses	KEGG_CHEMOKINE_SIGNALING_PATHWAY	
CCL4L2	disease responses	WP_COMPLEMENT_AND_COAGULATION_CASCADES	
GIMAP4	plaque necrosis	REACTOME_PEPTIDE_LIGAND_BINDING_RECEPTORS	
CCL3L3	tumor vasculature	WP_OVERVIEW_OF_PROINFLAMMATORY_AND_PROFIBROTIC_MEDIATORS	
FCGR3A	lymphocyte activation	HALLMARK_COAGULATION	
TREM2	tumor promotion	REACTOME_PURINERGIC_SIGNALING_IN_LEISHMANIASIS_INFECTION	
YWHAH	tumor-promoting macrophage responses	REACTOME_CLASS_A_1_RHODOPSIN_LIKE_RECEPTORS	
HTRA1	macrophage depletion	WP_INTERACTIONS_OF_NATURAL_KILLER_CELLS_IN_PANCREATIC_CANCER	
USP53	antitumor efficacy	REACTOME_INITIAL_TRIGGERING_OF_COMPLEMENT	
CX3CR1	systemic bacterial infection	WP_PROSTAGLANDIN_AND_LEUKOTRIENE_METABOLISM_IN_SENESCENCE	
CXCR4	profound microbiome depletion	HALLMARK_APOPTOSIS	
BTG2	effective tumor control	KEGG_HEMATOPOIETIC_CELL_LINEAGE	
CD14	antibody-mediated phagocytosis	WP_OXIDATIVE_DAMAGE_RESPONSE	
CD69	macrophage-mediated phagocytosis	KEGG_TOLL_LIKE_RECEPTOR_SIGNALING_PATHWAY	
CD9	tumor infiltration	WP_TOLLLIKE_RECEPTOR_SIGNALING_PATHWAY	
CSF1R	anti-inflammatory responses	HALLMARK_ALLOGRAFT_REJECTION	
HERPUD1	cross-talk pathways	HALLMARK_TNFA_SIGNALING_VIA_NFKB	
HLA-DQA1	proinflammatory polarization	REACTOME_G_ALPHA_I_SIGNALLING_EVENTS	
IL1B	tumor mass	REACTOME_COMPLEMENT_CASCADE	
MEF2C	macrophage phagocytosis	WP_SPINAL_CORD_INJURY	
SLCO2B1	subclinical atherosclerosis	REACTOME_GPCR_LIGAND_BINDING	
ABI3	enhanced bacterial capture ability	WP_NETWORK_MAP_OF_SARSCOV2_SIGNALING_PATHWAY	
ADAM28	follicular lymphoma	KEGG_CYTOSOLIC_DNA_SENSING_PATHWAY	
C10ORF54	cancer-microenvironment interactions	WP_COVID19_ADVERSE_OUTCOME_PATHWAY	
CD93	transient peripheral transcriptomic alterations	WP_LUNG_FIBROSIS	
EPB41L2	murine peritoneal macrophage differentiation	WP_TYROBP_CAUSAL_NETWORK_IN_MICROGLIA	
ETV5	improved long-term systemic antitumor immunity	REACTOME_CREATION_OF_C4_AND_C2_ACTIVATORS	
IFI44L	tumor outgrowth	REACTOME_BIOSYNTHESIS_OF_SPECIALIZED_PRORESOLVING_MEDIATORS_SPMS	
IFNGR1	dependent cell phagocytosis	WP_CYTOSOLIC_DNASENSING_PATHWAY	
RB1	protective anti-tumor immune responses	WP_LTF_DANGER_SIGNAL_RESPONSE_PATHWAY	
ENTPD1	tumor cell expansion	WP_CHEMOKINE_SIGNALING_PATHWAY	
STAB1	cognitive function	REACTOME_SYNTHESIS_OF_LEUKOTRIENES_LT_AND_EOXINS_EX	
A2M	elicit immune-mediated tumor lysis	WP_NAD_METABOLISM_IN_ONCOGENEINDUCED_SENESCENCE_AND_MITOCHONDRIAL_DYSFUNCTIONASSOCIATED_SENESCENCE	
MARCKS	lesional efferocytosis	WP_EICOSANOID_SYNTHESIS	
MAFB	immunosuppressive macrophage-like characteristics	REACTOME_SIGNALING_BY_INTERLEUKINS	
FAM26F	tumor-promoting microenvironments	HALLMARK_KRAS_SIGNALING_UP	
	longer progression-free survival	WP_COMPLEMENT_SYSTEM	
	low-risk subgroups	WP_PROSTAGLANDIN_SIGNALING	
	local cellular clustering	WP_TYPE_II_INTERFERON_SIGNALING_IFNG	
	immune microenvironment changes		
	disease-associated denervation		
	somatic hypermutation		
	pathogenic envelope viruses		
	decipher hypoxia-induced pro-tumor immune suppression		
	macrophage-induced phagocytosis		
	inflammatory macrophage polarization		

MYC_Mitochondria	KG_annotation (ranked by pagerank scor	ORA_annotation	LLM_annotation
NME1	epithelial ovarian cancer	HALLMARK_MYC_TARGETS_V1	Mitochondrial Protein Synthesis and Cellular Stress Response (0.85)
NHP2	cancer immunosurveillance	HALLMARK_MTORC1_SIGNALING	
C1QBP	autochthonous immune response	REACTOME_FOLDING_OF_ACTIN_BY_CCT_TRIC	
PHB	recurrence prediction potential	HALLMARK_MYC_TARGETS_V2	
SDF2L1	perihilar large duct type	REACTOME_MITOCHONDRIAL_TRANSLATION	
DCTPP1	fungal-associated allergenic disease	REACTOME_FORMATION_OF_TUBULIN_FOLDING_INTERMEDIATES_BY_CCT_TRIC	
SRM	myeloid cell metabolism	REACTOME_MITOCHONDRIAL_PROTEIN_IMPORT	
CCT2	anti-tumor phenotype	REACTOME_COOPERATION_OF_PREFOLDIN_AND_TRIC_CCT_IN_ACTIN_AND_TUBULIN_FOLDING	
CORO1A	myeloid cell function	WP_16P112_PROXIMAL_DELETION_SYNDROME	
FABP5	clonal expansion	REACTOME_COOPERATION_OF_PDCL_PHLP1_AND_TRIC_CCT_IN_G_PROTEIN_BETA_FOLDING	
MRPL12	human myeloma cell growth	REACTOME_ASSOCIATION_OF_TRIC_CCT_WITH_TARGET_PROTEINS_DURING_BIOSYNTHESIS	
PA2G4	kinase activity	REACTOME_TRANSLATION	
SPP1	direct cytolytic infection	REACTOME_BBSOME_MEDIATED_CARGO_TARGETING_TO_CILIUM	
APEX1	significantly increased cell apoptosis	REACTOME_PROTEIN_LOCALIZATION	
RANBP1	enhanced immune cell recruitment	REACTOME_PROTEIN_FOLDING	
ATP5MC1	intratumoral transcriptional heterogeneit	HALLMARK_OXIDATIVE_PHOSPHORYLATION	
CCT5	tumor regulatory mechanisms	REACTOME_CARGO_TRAFFICKING_TO_THE_PERICILIARY_MEMBRANE	
CCT6A	critical auto-inflammatory adverse events	REACTOME_RHOBTB2_GTPASE_CYCLE	
EBNA1BP2	pro-fibrotic activities		
EIF5A	cell pyroptosis		
MRPL3	aging-related inflammation		
NOP16	macrophage-dependent manner		
PPA1			
TIMM13			
TOMM40			
ALOX5AP			
BOLA3			
HSPA5			
HSPD1			
LDHB			
MRPS12			
MRPS34			
RRP7A			
C20ORF27			
CCT3			
CLEC5A			
CYC1			
GGCT			
KCNN4			
MANF			
MRPL4			
MRPL52			
PPP1R14B			
PSMC4			
SNRPD1			
ISOC2			
CCDC85B			
PDCD5			
HNRNPAB			
PRMT1			

Unfolded-protein-respons	ICKG_annotation (ranked by pagerank score)	ORA_annotation	LLM_annotation
HSPA5	inducible nitric oxide synthetase	WP_PHOTODYNAMIC_THERAPYINDUCED_UNFOLDED_PROTEIN_RESPONSE	Endoplasmic Reticulum Stress Response and Protein Folding (0.92)
MANF	cancer immunosurveillance	REACTOME_UNFOLDED_PROTEIN_RESPONSE_UPR	
HSP90B1	autochthonous immune response	REACTOME_ATF6_ATF6_ALPHA_ACTIVATES_CHAPERONE_GENES	
SDF2L1	enhanced cancer-directed phagocytosis	REACTOME_ATF6_ATF6_ALPHA_ACTIVATES_CHAPERONES	
OSTC	anti-tumor immunomodulation	HALLMARK_UNFOLDED_PROTEIN_RESPONSE	
PDIA4	epithelial ovarian cancer	HALLMARK_HYPOXIA	
PDIA6	anticancer therapy	REACTOME_IRE1ALPHA_ACTIVATES_CHAPERONES	
SPCS2	primary myelofibrosis	WP_VEGFAVEGFR2_SIGNALING_PATHWAY	
CRELD2	anticancer cellular immunotherapy	REACTOME_ANTIGEN_PRESENTATION_FOLDING_ASSEMBLY_AND_PEPTIDE_LOADING_OF_CLASS_I_MHC	
DNAJB9	macrophage-directed therapy	HALLMARK_MTORC1_SIGNALING	
TMED2	antitumor administration	KEGG_PROTEIN_EXPORT	
TRAM1	acute tubulointerstitial nephritis	KEGG_ANTIGEN_PROCESSING_AND_PRESENTATION	
VIMP	enhanced immune cell recruitment	WP_PRION_DISEASE_PATHWAY	
HM13	immunotherapy regulation	HALLMARK_INTERFERON_ALPHA_RESPONSE	
LMAN1	aberrant cell proliferation	HALLMARK_P53_PATHWAY	
NDRG1	tumor escape	REACTOME_SRP_DEPENDENT_COTRANSLATIONAL_PROTEIN_TARGETING_TO_MEMBRANE	
NUCB2	tumor-associated macrophages	REACTOME_REGULATION_OF_INSULIN_LIKE_GROWTH_FACTOR_IGF_TRANSPORT_AND_UPTAKE_BY_INSULIN_LIKE_GROWTH_FACTOR_BINDING_PROTEINS_IGFBPS	
PDIA3	gastric tumorigenesis	REACTOME_SYNTHESIS_SECRETION_AND_INACTIVATION_OF_GLUCOSE_DEPENDENT_INSULINOTROPIC_POLYPEPTIDE_GIP	
SEC11C	recurrence prediction potential	REACTOME_THE_NLRP3_INFLAMMASOME	
SELM	potential epithelium-immune cell interactions	REACTOME_SCAVENGING_BY_CLASS_A_RECEPTORS	
SNHG12	male-biased transcriptional activation	REACTOME_SYNTHESIS_SECRETION_AND_DEACYLATION_OF_GHRELIN	
TMEM45A	sex-specific targeted interventions	REACTOME_INFLAMMASOMES	
ADM	sex-biased susceptibility	REACTOME_SYNTHESIS_SECRETION_AND_INACTIVATION_OF_GLUCAGON_LIKE_PEPTIDE_1_GLP_1	
ARF4	decipher immune responses	REACTOME_ASPARAGINE_N_LINKED_GLYCOSYLATION	
CALR	antitumor immune responses	REACTOME_INCRETIN_SYNTHESIS_SECRETION_AND_INACTIVATION	
DNAJB11	immune checkpoint inhibitors	WP_UNFOLDED_PROTEIN_RESPONSE	
ISG15	pro-apoptosis effect	REACTOME_CALNEXIN_CALRETICULIN_CYCLE	
LY6E	activated natural killer cells	REACTOME_PURINERGIC_SIGNALING_IN_LEISHMANIASIS_INFECTION	
PPIB	tumor necrosis factor	HALLMARK_GLYCOLYSIS	
SLC3A2	tumoricidal factors	HALLMARK_INTERFERON_GAMMA_RESPONSE	
SMIM14	clinical melanoma metastasis suppression	REACTOME_BINDING_AND_UPTAKE_OF_LIGANDS_BY_SCAVENGER_RECEPTORS	
TMEM258	micro-environmental regulation	WP_COMPLEMENT_SYSTEM	
TXN	tumor-promoting roles	HALLMARK_ANDROGEN_RESPONSE	
WARS	tumor burden	HALLMARK_PI3K_AKT_MTOR_SIGNALING	
HYOU1	kinase activity	REACTOME_ANTIGEN_PROCESSING_CROSS_PRESENTATION	
SDC2	direct cytolytic infection	REACTOME_PERK_REGULATES_GENE_EXPRESSION	
CDK2AP2	significantly increased cell apoptosis	REACTOME_CARGO_CONCENTRATION_IN_THE_ER	
GBP2	anti-cancer activity	REACTOME_N_GLYCAN_TRIMMING_IN_THE_ER_AND_CALNEXIN_CALRETICULIN_CYCLE	
AARS	lung adenocarcinoma progression	WP_TYPE_II_INTERFERON_SIGNALING_IFNG	
TAP1	engineered immune cell-based cancer immunothera	REACTOME_RESPONSE_TO_ELEVATED_PLATELET_CYTOSOLIC_CA2	
EGLN3	efficient cancer immunotherapy	WP_ENDOPLASMIC_RETICULUM_STRESS_RESPONSE_IN_CORONAVIRUS_INFECTION	
SLC33A1	immune deserts		
DNAJC1	myeloid-related inflammatory changes		
SSR2	endothelial dysfunction		
ZFAS1	solid tumour cancers		
XPOT	immune characteristics		
MT2A	longer progression-free survival		
HMOX1	low-risk subgroups		
DDIT3	cancer burden		
CALU	dissemination trajectories		
	checkpoint inhibitor pneumonitis		
	immune-related neutropenia		
	macrophage-driven mechanisms		
	intratumoral redistribution		
	antitumor effectiveness		
	immune complexes		
	experimental atherosclerosis		

Respiration	CKG_annotation (ranked by pagerank score	ORA_annotation	LLM_annotation
ISG15	gastric adenocarcinoma	REACTOME_RESPIRATORY_ELECTRON_TRANSPORT	Immune Response and Oxidative Stress Management (0.85)
CCL2	extracellular matrix	REACTOME_CELLULAR_RESPONSE_TO_CHEMICAL_STRESS	
COX7B	immune checkpoint inhibitors	WP_NONALCOHOLIC_FATTY_LIVER_DISEASE	
NDUFB2	established metastatic disease	WP_ELECTRON_TRANSPORT_CHAIN_OXPHOS_SYSTEM_IN_MITOCHONDRIA	
POLR2L	epithelial ovarian cancer	KEGG_ALZHEIMERS_DISEASE	
S100A8	anticancer therapy	KEGG_HUNTINGTONS_DISEASE	
ATOX1	potential epithelium-immune cell interactio	REACTOME_RESPIRATORY_ELECTRON_TRANSPORT_ATP_SYNTHESIS_BY_CHEMIOSMOTIC_COUPLING_AND_HEAT_PRODUCTION_BY_UNCOUPLING_PROTEINS	
ATP5J2	male-biased transcriptional activation	KEGG_PARKINSONS_DISEASE	
BLVRB	sex-specific targeted interventions	KEGG_OXIDATIVE_PHOSPHORYLATION	
CHCHD10	sex-biased susceptibility	HALLMARK_OXIDATIVE_PHOSPHORYLATION	
FABP5	decipher immune responses	REACTOME_THE_CITRIC_ACID_TCA_CYCLE_AND_RESPIRATORY_ELECTRON_TRANSPORT	
MRPL41	liver disease	REACTOME_CYTOPROTECTION_BY_HMOX1	
NDUFA3	lymphocyte activation	WP_MITOCHONDRIAL_COMPLEX_I_ASSEMBLY_MODEL_OXPHOS_SYSTEM	
NDUFS6	anticancer cellular immunotherapy	REACTOME_COMPLEX_I_BIOGENESIS	
PSME2	macrophage-directed therapy	WP_OXIDATIVE_PHOSPHORYLATION	
RBX1	antitumor administration	REACTOME_INFLUENZA_INFECTION	
TIMM8B	fungal-associated allergenic disease	REACTOME_REGULATION_OF_EXPRESSION_OF_SLITS_AND_ROBOS	
UQCRQ	systemic bacterial infection	REACTOME_TRANSLATION	
VAMP5	profound microbiome depletion	HALLMARK_INTERFERON_GAMMA_RESPONSE	
RNASE1	recurrence prediction potential	REACTOME_SIGNALING_BY_ROBO_RECEPTORS	
HCST	antitumor immune responses	WP_IL10_ANTIINFLAMMATORY_SIGNALING_PATHWAY	
MYEOV2	enhanced cancer-directed phagocytosis	REACTOME_HEME_DEGRADATION	
CALM3	anti-tumor immunomodulation	REACTOME_REGULATION_OF_HMOX1_EXPRESSION_AND_ACTIVITY	
S100A9	tumor sites	REACTOME_THE_NLRP3_INFLAMMASOME	
C4ORF48	pro-apoptosis effect	WP_MITOCHONDRIAL_COMPLEX_III_ASSEMBLY	
DNPH1	activated natural killer cells	WP_NONGENOMIC_ACTIONS_OF_125_DIHYDROXYVITAMIN_D3	
TXN	spontaneous intestinal tumorigenesis	REACTOME_IRAK4_DEFICIENCY_TLR2_4	
CXCL9	cancer cell survival	KEGG_CARDIAC_MUSCLE_CONTRACTION	
STAB1	tumor necrosis factor	REACTOME_INFLAMMASOMES	
HAMP	tumoricidal factors	REACTOME_REGULATION_OF_TLR_BY_ENDOGENOUS_LIGAND	
NDUFB7	clinical melanoma metastasis suppression	WP_NRF2ARE_REGULATION	
TXNDC17	antitumor effects	REACTOME_TP53_REGULATES_METABOLIC_GENES	
DAB2	critical pro-tumoral roles	REACTOME_RHO_GTPASES_ACTIVATE_NADPH_OXIDASES	
BRI3	br-met outgrowth	KEGG_RIBOSOME	
CTSZ	recruited myeloid cells	WP_CYTOPLASMIC_RIBOSOMAL_PROTEINS	
RPS21	cutaneous melanoma	WP_IL1_AND_MEGAKARYOCYTES_IN_OBESITY	
RPS29	anti-tumor effects	REACTOME_PURINERGIC_SIGNALING_IN_LEISHMANIASIS_INFECTION	
SLIRP	optimal combination regimens	REACTOME_EUKARYOTIC_TRANSLATION_ELONGATION	
RPL38	critical auto-inflammatory adverse events	REACTOME_METABOLISM_OF_PORPHYRINS	
UQCC2	checkpoint inhibitor pneumonitis	REACTOME_MITOCHONDRIAL_TRANSLATION	
MRPL51	immune-related neutropenia	HALLMARK_ADIPOGENESIS	
ATP5EP2	myeloid cell polarization	HALLMARK_ESTROGEN_RESPONSE_LATE	
DUSP23	adaptive/acquired resistance	REACTOME_ANTIMICROBIAL_PEPTIDES	
HSPE1	antitumor immune response	REACTOME_RESPONSE_OF_EIF2AK4_GCN2_TO_AMINO_ACID_DEFICIENCY	
IFI27L2	pancreatic cancer burden	REACTOME_DISEASES_OF_IMMUNE_SYSTEM	
C19ORF70	chemokine receptors	REACTOME_ANTIGEN_PROCESSING_CROSS_PRESENTATION	
GADD45GIP1	immunotherapy regulation	WP_ANTIVIRAL_AND_ANTIINFLAMMATORY_EFFECTS_OF_NRF2_ON_SARSCOV2_PATHWAY	
COX6C	aberrant cell proliferation	WP_PROSTAGLANDIN_SIGNALING	
ATP5I	tumor escape	REACTOME_SRP_DEPENDENT_COTRANSLATIONAL_PROTEIN_TARGETING_TO_MEMBRANE	
HMOX1	myeloid-related inflammatory changes	REACTOME_NONSENSE_MEDIATED_DECAY_NMD	
	endothelial dysfunction	WP_MITOCHONDRIAL_COMPLEX_IV_ASSEMBLY	
	solid tumour cancers	REACTOME_SELENOAMINO_ACID_METABOLISM	
	macrophage-driven mechanisms	REACTOME_DETOXIFICATION_OF_REACTIVE_OXYGEN_SPECIES	
	intratumoral redistribution	WP_TYPE_II_INTERFERON_SIGNALING_IFNG	
	innate anticancer immunity	REACTOME_EUKARYOTIC_TRANSLATION_INITIATION	
	cancer cell phagocytosis	KEGG_PORPHYRIN_AND_CHLOROPHYLL_METABOLISM	
	immune cell-associated diseases		
	increased leukocyte infiltration		
	antitumor effectiveness		
	immune complexes		
	experimental atherosclerosis		

T_reg	ICKG_annotation (ranked by pagerank score)	ORA_annotation	LLM_annotation
IL2RA	disease susceptibility	HALLMARK_IL2_STAT5_SIGNALING	T-cell Activation and Immune Response Regulation (0.85)
TNFRSF18	regulatory lymphocytes	KEGG_CYTOKINE_CYTOKINE_RECEPTOR_INTERACTION	
TNFRSF4	complete tumor eradication	REACTOME_RUNX1_AND_FOXP3_CONTROL_THE_DEVELOPMENT_OF_REGULATORY_T_LYMPHOCYTES_TREGS	
TIGIT	immune escape mechanism	REACTOME_TNFS_BIND_THEIR_PHYSIOLOGICAL_RECEPTORS	
BATF	90% tumor response	REACTOME_TNFR2_NON_CANONICAL_NF_KB_PATHWAY	
CD27	tumor necrosis	HALLMARK_TNFA_SIGNALING_VIA_NFKB	
CARD16	anti-tumor efficacy	WP_FOXP3_IN_COVID19	
FOXP3	activation-induced cell death	WP_GENES_ASSOCIATED_WITH_THE_DEVELOPMENT_OF_RHEUMATOID_ARTHRITIS	
CTLA4	insulin sensitivity	WP_INFLAMMATORY_RESPONSE_PATHWAY	
TNFRSF1B	critical immune factors	HALLMARK_APOPTOSIS	
PMAIP1	systemic immunotoxicity	HALLMARK_IL6_JAK_STAT3_SIGNALING	
CTSC	lysosome-mediated degradation	KEGG_HEMATOPOIETIC_CELL_LINEAGE	
LAIR2	immune repression	WP_ALLOGRAFT_REJECTION	
TNFRSF9	proper organ function	WP_APOPTOSIS_MODULATION_AND_SIGNALING	
GLRX	anti-tumour immune responses	WP_INTERACTIONS_BETWEEN_IMMUNE_CELLS_AND_MICRORNAS_IN_TUMOR_MICROENVIRONMENT	
DNPH1	intestinal barrier function	HALLMARK_ALLOGRAFT_REJECTION	
TBC1D4	gut immune tolerance	HALLMARK_INFLAMMATORY_RESPONSE	
DUSP4	immune tolerance-mediated mucosal homeost	HALLMARK_INTERFERON_GAMMA_RESPONSE	
GBP2	keratinocyte proliferation	REACTOME_INTERLEUKIN_2_SIGNALING	
ICOS	upregulated cytokine pathway signatures	REACTOME_SIGNALING_BY_INTERLEUKINS	
MAGEH1	anti-tumour immunity	WP_CANCER_IMMUNOTHERAPY_BY_CTLA4_BLOCKADE	
TYMP	exhaustion markers	WP_P53_TRANSCRIPTIONAL_GENE_NETWORK	
CXCR6	immune checkpoint dysfunction	REACTOME_COSTIMULATION_BY_THE_CD28_FAMILY	
CD7	optimal primary expansion	WP_TP53_NETWORK	
CST7	complete tumour regression	WP_IMATINIB_AND_CHRONIC_MYELOID_LEUKEMIA	
SAT1	immune-based cancer therapies	WP_CANCER_IMMUNOTHERAPY_BY_PD1_BLOCKADE	
GADD45A	cytotoxic antitumor effects	WP_APOPTOSIS	
IL1R2	effector function inhibition	WP_TCELL_ACTIVATION_SARSCOV2	
GBP5	antitumor properties	REACTOME_INTERFERON_GAMMA_SIGNALING	
UGP2	immune system balance	REACTOME_INTERLEUKIN_RECEPTOR_SHC_SIGNALING	
AC133644.2	immune-tumor cell-cell interactions	REACTOME_METABOLISM_OF_NUCLEOTIDES	
UCP2	improved antitumor activity	REACTOME_INTERFERON_SIGNALING	
PIM2	epigenetic alterations	REACTOME_NUCLEOTIDE_CATABOLISM	
LAYN	suppress antitumor immune responses		
PKM	established signal transduction pathways		
RTKN2	cross-present melanoma-derived tumor antigens		
ACP5	multiple inhibitory receptors		
MIR4435-2HG	ovarian carcinoma		
ARID5B	strong inhibition		
PHLDA1	intra-tumoral pathological response		
IL2RB	marrow-residence genes		
SYNGR2	macrophage activation syndrome		
LTB	lineage-specific gene programs		
CASP1	autoimmune-mediated neuroinflammation		
HLA-DRB1	tumor-mediated immune escape		
IFI6	skin rashes		
NAMPT	type 2/type 17 immune responses		
CORO1B	treatment-associated tumor necrosis		
LGALS1	rational combination treatments		
BTG3	improved tumor growth delay		
	mild cytokine release syndrome		
	adaptive immune checkpoint resistance		
	immune cell recruitment		
	responsive hot tumors		
	medulla-dependent tolerance mechanisms		
	improved eradication		
	transplantable mouse tumor models		
	related plasma cell disorders		
	endogenous adaptive antitumour immunity		
	antiapoptotic genes		
	convergent inflammation		
	engulf cancer cells		
	post-ablation immune therapy		
	dramatic disease regression		
	better tumor control		
	antigen-driven differentiation		
	cancer microvessels		
	peripheral immune tolerance		
	multiple immune checkpoints		
	immunomodulation process		
	intact tumor-cell antigen presentation		
	lymph node homing capability		
	autocrine stimulation		
	proinflammatory immune responses		
	relapse prevention		
	event-free survival		
	significant toxicity		
	precision medicine		
	short-term immunologic response		
	long-term clinical outcomes		
	autoimmune disease treatment		
	higher antitumor activity		
	lymphocyte activatory receptors		
	retrovirus immunity		
	heterosubtypic protection		
	serous borderline ovarian tumours		
	anti-tumor immune phenotypes		
	tumor-specific-stereotyped sequences		
	epigenetically closed regions		
	long-term tumor remission		
	cancer precision medicine		
	larger genetic elements		
	epigenetic state		

Naive1	ICKG_annotation (ranked by pagerank score)	ORA_annotation	LLM_annotation
ANXA1	poorer prognosis	HALLMARK_TNFA_SIGNALING_VIA_NFKB	T-cell Activation and Immune Response Regulation (0.92)
FOS	immune evasion	HALLMARK_INFLAMMATORY_RESPONSE	
CD69	gastric cancer peritoneal metastasis	HALLMARK_HYPOXIA	
CCR7	poor gastric cancer	HALLMARK_APOPTOSIS	
GPR183	interstitial lung disease	HALLMARK_UV_RESPONSE_UP	
KLRB1	ovarian pathogenesis	WP_HOSTPATHOGEN_INTERACTION_OF_HUMAN_CORONAVIRUSES_MAPK_SIGNALING	
IL7R	cytotoxic antitumor effects	WP_CORTICOTROPINRELEASING_HORMONE_SIGNALING_PATHWAY	
GIMAP7	antitumor functions	WP_IL18_SIGNALING_PATHWAY	
CXCR4	tumor environments	REACTOME_NGF_STIMULATED_TRANSCRIPTION	
SLC2A3	disease outcome	WP_IL2_SIGNALING_PATHWAY	
BTG2	cell adhesion	REACTOME_NEGATIVE_REGULATION_OF_MAPK_PATHWAY	
CD55	tumor formation processes	HALLMARK_ESTROGEN_RESPONSE_LATE	
YPEL5	irreversible cirrhosis	HALLMARK_INTERFERON_GAMMA_RESPONSE	
ZFP36L2	cell survival	HALLMARK_P53_PATHWAY	
NR4A2	cancer immune surveillance	REACTOME_INTERLEUKIN_4_AND_INTERLEUKIN_13_SIGNALING	
GIMAP4	functional exhaustion	REACTOME_ACTIVATION_OF_THE_AP_1_FAMILY_OF_TRANSCRIPTION_FACTORS	
PTGER4	pro-immunogenic expression changes	WP_IL3_SIGNALING_PATHWAY	
ALOX5AP	immunogenic gene expression	WP_SPINAL_CORD_INJURY	
TRAT1	tumor immune status	REACTOME_MAPK_FAMILY_SIGNALING_CASCADES	
DUSP1	extracellular matrix proteins	WP_PHOTODYNAMIC_THERAPYINDUCED_AP1_SURVIVAL_SIGNALING	
FOSB	immune checkpoint dysfunction	REACTOME_CLASS_A_1_RHODOPSIN_LIKE_RECEPTORS	
CCL5	heightened tumor cell metabolism	REACTOME_GPCR_LIGAND_BINDING	
DNAJB1	dysregulated perfusion	REACTOME_SIGNALING_BY_INTERLEUKINS	
TXNIP	protective immune memory	WP_NETWORK_MAP_OF_SARSCOV2_SIGNALING_PATHWAY	
SELL	subsequent immune infiltration	WP_NEUROINFLAMMATION	
KLF2	advanced gastric cancer	REACTOME_CHEMOKINE_RECEPTORS_BIND_CHEMOKINES	
GPR171	immune-tumor cell-cell interactions	WP_TGFBETA_SIGNALING_PATHWAY	
GZMA	antibody dynamics	WP_OVERLAP_BETWEEN_SIGNAL_TRANSDUCTION_PATHWAYS_CONTRIBUTING_TO_LMNA_LAMINOPATHIES	
PLAC8	metabolic dysfunction-associated steatohepatitis	WP_PREIMPLANTATION_EMBRYO	
CD48	systemic immunotoxicity	REACTOME_NUCLEAR_EVENTS_KINASE_AND_TRANSCRIPTION_FACTOR_ACTIVATION	
JUN	peritoneal dissemination	KEGG_COLORECTAL_CANCER	
TCF7	gastric cancer development	WP_HEMATOPOIETIC_STEM_CELL_DIFFERENTIATION	
PPP2R5C	intestinal-type gastric carcinoma	WP_MAPK_PATHWAY_IN_CONGENITAL_THYROID_CANCER	
AREG	cellular tropism	WP_QUERCETIN_AND_NFKB_AP1_INDUCED_APOPTOSIS	
RGCC	epigenetic alterations	WP_ONCOSTATIN_M_SIGNALING_PATHWAY	
FOXP1	Œ±vŒ≤3 integrins	WP_AMPLIFICATION_AND_EXPANSION_OF_ONCOGENIC_PATHWAYS_AS_METASTATIC_TRAITS	
FAM177A1	durable tumor remission	WP_CHROMOSOMAL_AND_MICROSATELLITE_INSTABILITY_IN_COLORECTAL_CANCER	
NOSIP	efficacious suppression	WP_PROLACTIN_SIGNALING_PATHWAY	
RARRES3	Œ≥Œ¥ cell-based therapies	REACTOME_TP53_REGULATES_TRANSCRIPTION_OF_ADDITIONAL_CELL_CYCLE_GENES_WHOSE_EXACT_ROLE_IN_THE_P53_PATHWAY_REMAIN_UNCERTAIN	
LMNA	Œ≥Œ¥ reconstitution	WP_REGULATORY_CIRCUITS_OF_THE_STAT3_SIGNALING_PATHWAY	
SNHG8	increased fibrosis	REACTOME_RAF_INDEPENDENT_MAPK1_3_ACTIVATION	
ZNF331	established signal transduction pathways	WP_ESTROGEN_SIGNALING_PATHWAY	
DUSP2	memory-like responses	WP_PHOTODYNAMIC_THERAPYINDUCED_NFE2L2_NRF2_SURVIVAL_SIGNALING	
IER2	autoimmune hepatitis	REACTOME_ESTROGEN_DEPENDENT_NUCLEAR_EVENTS_DOWNSTREAM_OF_ESR_MEMBRANE_SIGNALING	
JUNB	receptor function	WP_HAIR_FOLLICLE_DEVELOPMENT_CYTODIFFERENTIATION_PART_3_OF_3	
SOCS3	viral gene expression	REACTOME_DEFECTIVE_INTRINSIC_PATHWAY_FOR_APOPTOSIS	
TMEM123	drug-resistant tumors	WP_PHYSIOLOGICAL_AND_PATHOLOGICAL_HYPERTROPHY_OF_THE_HEART	
TOB1	type 2 immune response	REACTOME_G_ALPHA_I_SIGNALLING_EVENTS	
ZFP36	type 2 immune responses	WP_NUCLEAR_RECEPTORS_METAPATHWAY	
LEPROTL1	virus-neutralizing antibodies	REACTOME_PEPTIDE_LIGAND_BINDING_RECEPTORS	
	dysregulated tumor glycolysis	WP_PDGFRBETA_PATHWAY	
	impaired anti-tumor activity	WP_SELECTIVE_EXPRESSION_OF_CHEMOKINE_RECEPTORS_DURING_TCELL_POLARIZATION	
	insufficient infiltration	KEGG_TOLL_LIKE_RECEPTOR_SIGNALING_PATHWAY	
	immunocytes' activation	REACTOME_MAPK_TARGETS_NUCLEAR_EVENTS_MEDIATED_BY_MAP_KINASES	
	reduced terminal exhaustion	WP_FAMILIAL_PARTIAL_LIPODYSTROPHY_FPLD	
	increased memory cell characteristics	WP_PROSTAGLANDIN_AND_LEUKOTRIENE_METABOLISM_IN_SENESCENCE	
	treatment-associated tumor necrosis	WP_TOLLLIKE_RECEPTOR_SIGNALING_PATHWAY	
	local tissue protection	WP_WNT_SIGNALING_PATHWAY_AND_PLURIPOTENCY	
	adaptive anti-tumor immunity	WP_GASTRIC_CANCER_NETWORK_2	
	chronic obstructive pulmonary disease	WP_HOSTPATHOGEN_INTERACTION_OF_HUMAN_CORONAVIRUSES_INTERFERON_INDUCTION	
	cystic fibrosis	WP_PROSTAGLANDIN_SIGNALING	
	immune landscape	REACTOME_ESR_MEDIATED_SIGNALING	
	dense tumor infiltration	WP_HEPATOCYTE_GROWTH_FACTOR_RECEPTOR_SIGNALING	
	engulf cancer cells	WP_OXIDATIVE_STRESS_RESPONSE	
	perihilar large duct type	REACTOME_NEGATIVE_REGULATION_OF_THE_PI3K_AKT_NETWORK	
	lymphocyte differentiation	WP_DNA_DAMAGE_RESPONSE_ONLY_ATM_DEPENDENT	
	post allogeneic hematopoietic stem cell transplanta	REACTOME_TRANSCRIPTIONAL_REGULATION_BY_TP53	
	cancer immunoprevention	WP_IL5_SIGNALING_PATHWAY	
	monocyte differentiation	WP_INFLUENCE_OF_LAMINOPATHIES_ON_WNT_SIGNALING	
	organ-adapted protection	WP_PDGF_PATHWAY	
	tumor microenvironments	WP_NEURAL_CREST_CELL_MIGRATION_DURING_DEVELOPMENT	
	intrahepatic cholangiocarcinoma	WP_TNFRELATED_WEAK_INDUCER_OF_APOPTOSIS_TWEAK_SIGNALING_PATHWAY	
	immune checkpoint regulation	WP_MAPK_SIGNALING_PATHWAY	
	autoimmune reactions	WP_IL6_SIGNALING_PATHWAY	
	tumor-infiltrating immunosuppressive leukocytes		
	mixed inflammation		
	lysine-9 methyl-transferase		
	immunoglobulin deficiency		
	anti-tumor dysfunction		
	zeste homolog 2 methyltransferase		

Cell_cycle	ICKG_annotation (ranked by pagerank score)	ORA_annotation	LLM_annotation
STMN1	cell cycle	HALLMARK_E2F_TARGETS	Cell Cycle Regulation and Mitosis (0.95)
TUBA1B	long-term immunological memory	HALLMARK_G2M_CHECKPOINT	
TUBB	tumor-associated antigens	REACTOME_MITOTIC_PROMETAPHASE	
NUSAP1	synthetic peptide-based anticancer vaccines	REACTOME_M_PHASE	
HMGB2	tumor mutational burden	WP_RETINOBLASTOMA_GENE_IN_CANCER	
CKS1B	cancer immune regulation	REACTOME_MITOTIC_METAPHASE_AND_ANAPHASE	
TYMS	gene rearrangement	REACTOME_RESOLUTION_OF_SISTER_CHROMATID_COHESION	
UBE2C	plasma cell neoplasia	REACTOME_SEPARATION_OF_SISTER_CHROMATIDS	
MKI67	tumor keratinocytes	REACTOME_RHO_GTPASES_ACTIVATE_FORMINS	
TOP2A	tumor vascular abnormalities	REACTOME_MITOTIC_G1_PHASE_AND_G1_S_TRANSITION	
PCNA	improved antitumor activity	REACTOME_CELL_CYCLE_CHECKPOINTS	
TK1	myeloma-related disorder	REACTOME_G1_S_SPECIFIC_TRANSCRIPTION	
ZWINT	cancer relapse	REACTOME_MITOTIC_SPINDLE_CHECKPOINT	
BIRC5	cancer-related mortality	HALLMARK_MITOTIC_SPINDLE	
CDK1	anticancer immunity	REACTOME_MITOTIC_G2_G2_M_PHASES	
HIST1H4C	melanoma models	REACTOME_APC_C_MEDIATED_DEGRADATION_OF_CELL_CYCLE_PROTEINS	
DUT	disease outcome	REACTOME_METABOLISM_OF_NUCLEOTIDES	
CENPF	breast cancer clinical treatment selection	REACTOME_RHO_GTPASE_EFFECTORS	
HMGN2	protein folding	REACTOME_G0_AND_EARLY_G1	
MAD2L1	cancer vaccines	REACTOME_INTERCONVERSION_OF_NUCLEOTIDE_DI_AND_TRIPHOSPHATES	
CENPM	antitumor efficacy	WP_GASTRIC_CANCER_NETWORK_1	
SMC4	anti-tumorigenic innate immune cells	KEGG_CELL_CYCLE	
SMC2	anti-tumor innate immunity	HALLMARK_MYC_TARGETS_V1	
RRM2	long-term antitumor memory	REACTOME_NUCLEAR_ENVELOPE_NE_REASSEMBLY	
MCM7	cancer therapy resistance	REACTOME_CONDENSATION_OF_PROMETAPHASE_CHROMOSOMES	
CDKN3	certain hematological malignancies	WP_PYRIMIDINE_METABOLISM	
PTTG1	advanced melanoma	KEGG_GAP_JUNCTION	
KIAA0101	leukemia eradication	REACTOME_S_PHASE	
CKS2	elicit adaptive immune responses	REACTOME_RECRUITMENT_OF_NUMA_TO_MITOTIC_CENTROSOMES	
NUDT1	cancer-colonized organs	REACTOME_SUMOYLATION_OF_DNA_REPLICATION_PROTEINS	
TMEM106C	immune synapse formation	KEGG_PYRIMIDINE_METABOLISM	
UBE2T	respiratory disease	WP_PATHOGENIC_ESCHERICHIA_COLI_INFECTION	
ASF1B	anti-acetylcholine receptor antibody production	KEGG_PATHOGENIC_ESCHERICHIA_COLI_INFECTION	
DNAJC9	cell type specific gene-regulatory programs	REACTOME_TRANSPORT_OF_CONNEXONS_TO_THE_PLASMA_MEMBRANE	
H2AFZ	peripheral blood immune system	REACTOME_SYNTHESIS_OF_DNA	
CENPW	precision cancer immunotherapies	REACTOME_KINESINS	
ASPM	signaling	WP_CELL_CYCLE	
DTYMK	dual-lymphoid activation	REACTOME_POST_CHAPERONIN_TUBULIN_FOLDING_PATHWAY	
FEN1	co-stimulatory domains	WP_PARKINUBIQUITIN_PROTEASOMAL_SYSTEM_PATHWAY	
PCLAF	advanced melanoma patients	REACTOME_FORMATION_OF_TUBULIN_FOLDING_INTERMEDIATES_BY_CCT_TRIC	
TPX2	tumor-infiltrating lymphocyte therapy	REACTOME_CHROMOSOME_MAINTENANCE	
TUBB4B	active antitumoral immunity	REACTOME_AURKA_ACTIVATION_BY_TPX2	
AURKB	putative cancer immune evasion	REACTOME_ACTIVATION_OF_AMPK_DOWNSTREAM_OF_NMDARS	
TMPO	adjuvant dendritic cell vaccination	HALLMARK_DNA_REPAIR	
TUBA1C	substantial global tumor regression	REACTOME_THE_ROLE_OF_GTSE1_IN_G2_M_PROGRESSION_AFTER_G2_CHECKPOINT	
DDX39A	efficient tumor-immune responses	REACTOME_DNA_STRAND_ELONGATION	
KPNA2	marrow-residence genes	REACTOME_RHO_GTPASES_ACTIVATE_IQGAPS	
PHF19	antigen-specific cellular immunity	REACTOME_SEALING_OF_THE_NUCLEAR_ENVELOPE_NE_BY_ESCRT_III	
KIF22	melanoma tumour burden	REACTOME_COOPERATION_OF_PREFOLDIN_AND_TRIC_CCT_IN_ACTIN_AND_TUBULIN_FOLDING	
CCNA2	melanoma development	WP_GASTRIC_CANCER_NETWORK_2	
	cancer-immune status	WP_FLUOROPYRIMIDINE_ACTIVITY	
	plasma cell differentiation	WP_COHESIN_COMPLEX_CORNELIA_DE_LANGE_SYNDROME	
	severe cutaneous adverse reaction	KEGG_DNA_REPLICATION	
	widespread skin lesions	REACTOME_ACTIVATION_OF_NMDA_RECEPTORS_AND_POSTSYNAPTIC_EVENTS	
	virus-derived antigenic peptides	REACTOME_GAP_JUNCTION_ASSEMBLY	
	intrinsic dysregulation	REACTOME_COPI_DEPENDENT_GOLGI_TO_ER_RETROGRADE_TRAFFIC	
	tumor-promoting crosstalk	REACTOME_DNA_REPLICATION	
	immune-related gene signatures	REACTOME_AGGREPHAGY	
	hypersensitivity reaction	REACTOME_ASSEMBLY_AND_CELL_SURFACE_PRESENTATION_OF_NMDA_RECEPTORS	
	mild cytokine release syndrome	REACTOME_CARBOXYTERMINAL_POST_TRANSLATIONAL_MODIFICATIONS_OF_TUBULIN	
	lymph node homing capability	REACTOME_CILIUM_ASSEMBLY	
	myeloid cell clearance	REACTOME_RECYCLING_PATHWAY_OF_L1	
	costimulation-dependent dysfunctional programs	REACTOME_TP53_REGULATES_TRANSCRIPTION_OF_CELL_CYCLE_GENES	
	dysfunctional cells	WP_MICROTUBULE_CYTOSKELETON_REGULATION	
	co-mutated lung adenocarcinoma	REACTOME_EXTENSION_OF_TELOMERES	
	human leukocyte antigen background	REACTOME_GAP_JUNCTION_TRAFFICKING_AND_REGULATION	
	decreased antigen recognition ability	REACTOME_DNA_REPAIR	
	diffuse glioma	REACTOME_COPI_INDEPENDENT_GOLGI_TO_ER_RETROGRADE_TRAFFIC	
	adoptive cellular transfer	REACTOME_INTRAFLAGELLAR_TRANSPORT	
	mammary tumorigenesis	REACTOME_MHC_CLASS_II_ANTIGEN_PRESENTATION	
	potent complement-dependent cytotoxic effects	REACTOME_HSP90_CHAPERONE_CYCLE_FOR_STEROID_HORMONE_RECEPTORS_SHR_IN_THE_PRESENCE_OF_LIGAND	
	bispecific antibody-mediated antitumour activity	REACTOME_GOLGI_TO_ER_RETROGRADE_TRANSPORT	
	interferon regulatory factor 1 transcriptional activat	REACTOME_DEPOLYMERISATION_OF_THE_NUCLEAR_LAMINA	
	heparan sulfate backbone synthesis	REACTOME_PROCESSIVE_SYNTHESIS_ON_THE_LAGGING_STRAND	
	unfolded proteins	WP_BIOMARKERS_FOR_PYRIMIDINE_METABOLISM_DISORDERS	
	microenvironmental immunosuppression	REACTOME_MITOTIC_PROPHASE	
	kidney transplantation	REACTOME_TRANSCRIPTIONAL_REGULATION_BY_TP53	
	renal allograft dysfunction	WP_G1_TO_S_CELL_CYCLE_CONTROL	
	skin cutaneous melanoma	REACTOME_TRANSCRIPTION_OF_E2F_TARGETS_UNDER_NEGATIVE_CONTROL_BY_P107_RBL1_AND_P130_RBL2_IN_COMPLEX_WITH_HDAC1	
	local expansion	WP_REGULATION_OF_SISTER_CHROMATID_SEPARATION_AT_THE_METAPHASEANAPHASE_TRANSITION	
	immune-stromal interaction mechanisms	WP_PYRIMIDINE_METABOLISM_AND_RELATED_DISEASES	
	coagulation cascade signals	REACTOME_TP53_REGULATES_TRANSCRIPTION_OF_GENES_INVOLVED_IN_G2_CELL_CYCLE_ARREST	
		REACTOME_CONDENSATION_OF_PROPHASE_CHROMOSOMES	
		REACTOME_TRANSLOCATION_OF_SLC2A4_GLUT4_TO_THE_PLASMA_MEMBRANE	
		REACTOME_INITIATION_OF_NUCLEAR_ENVELOPE_NE_REFORMATION	
		REACTOME_PROCESSIVE_SYNTHESIS_ON_THE_C_STRAND_OF_THE_TELOMERE	
		REACTOME_TRANSCRIPTION_OF_E2F_TARGETS_UNDER_NEGATIVE_CONTROL_BY_DREAM_COMPLEX	
		REACTOME_APC_C_CDH1_MEDIATED_DEGRADATION_OF_CDC20_AND_OTHER_APC_C_CDH1_TARGETED_PROTEINS_IN_LATE_MITOSIS_EARLY_G1	
		REACTOME_REGULATION_OF_TP53_ACTIVITY	
		REACTOME_LAGGING_STRAND_SYNTHESIS	
		REACTOME_PHOSPHORYLATION_OF_THE_APC_C	
		REACTOME_INHIBITION_OF_THE_PROTEOLYTIC_ACTIVITY_OF_APC_C_REQUIRED_FOR_THE_ONSET_OF_ANAPHASE_BY_MITOTIC_SPINDLE_CHECKPOINT_COMPONENTS	
		REACTOME_PCNA_DEPENDENT_LONG_PATCH_BASE_EXCISION_REPAIR	
		REACTOME_DNA_DOUBLE_STRAND_BREAK_REPAIR	
		REACTOME_PROTEIN_UBIQUITINATION	
		REACTOME_FACTORS_INVOLVED_IN_MEGAKARYOCYTE_DEVELOPMENT_AND_PLATELET_PRODUCTION	
		REACTOME_RECRUITMENT_OF_MITOTIC_CENTROSOME_PROTEINS_AND_COMPLEXES	
		WP_DNA_IRDAMAGE_AND_CELLULAR_RESPONSE_VIA_ATR	
		REACTOME_SELECTIVE_AUTOPHAGY	
		KEGG_PROGESTERONE_MEDIATED_OOCYTE_MATURATION	
		REACTOME_ORGANELLE_BIOGENESIS_AND_MAINTENANCE	
		REACTOME_APC_C_CDC20_MEDIATED_DEGRADATION_OF_CYCLIN_B	
		REACTOME_REGULATION_OF_PLK1_ACTIVITY_AT_G2_M_TRANSITION	
		REACTOME_CYCLIN_A_B1_B2_ASSOCIATED_EVENTS_DURING_G2_M_TRANSITION	
		REACTOME_RESOLUTION_OF_AP_SITES_VIA_THE_MULTIPLE_NUCLEOTIDE_PATCH_REPLACEMENT_PATHWAY	
		REACTOME_SUMOYLATION	
		REACTOME_APC_CDC20_MEDIATED_DEGRADATION_OF_NEK2A	
		REACTOME_SWITCHING_OF_ORIGINS_TO_A_POST_REPLICATIVE_STATE	
		REACTOME_REGULATION_OF_TP53_ACTIVITY_THROUGH_PHOSPHORYLATION	
		REACTOME_ANCHORING_OF_THE_BASAL_BODY_TO_THE_PLASMA_MEMBRANE	
		REACTOME_PROTEIN_FOLDING	
		REACTOME_INTRA_GOLGI_AND_RETROGRADE_GOLGI_TO_ER_TRAFFIC	
		REACTOME_SYNTHESIS_OF_ACTIVE_UBIQUITIN_ROLES_OF_E1_AND_E2_ENZYMES	
		REACTOME_NEUROTRANSMITTER_RECEPTORS_AND_POSTSYNAPTIC_SIGNAL_TRANSMISSION	
		REACTOME_COPI_MEDIATED_ANTEROGRADE_TRANSPORT	
		WP_BASE_EXCISION_REPAIR	
		REACTOME_TERMINATION_OF_TRANSLESION_DNA_SYNTHESIS	
		REACTOME_TELOMERE_C_STRAND_LAGGING_STRAND_SYNTHESIS	
		REACTOME_TELOMERE_MAINTENANCE	
		KEGG_BASE_EXCISION_REPAIR	
		KEGG_OOCYTE_MEIOSIS	
		REACTOME_HEDGEHOG_OFF_STATE	
		REACTOME_REGULATION_OF_TP53_EXPRESSION_AND_DEGRADATION	
		REACTOME_RESOLUTION_OF_ABASIC_SITES_AP_SITES	
		REACTOME_TRANSLESION_SYNTHESIS_BY_Y_FAMILY_DNA_POLYMERASES_BYPASSES_LESIONS_ON_DNA_TEMPLATE	
		REACTOME_L1CAM_INTERACTIONS	
		WP_DNA_REPLICATION	
		WP_ATM_SIGNALING_IN_DEVELOPMENT_AND_DISEASE	
		REACTOME_HCMV_EARLY_EVENTS	
		REACTOME_HOMOLOGY_DIRECTED_REPAIR	
		REACTOME_DNA_DAMAGE_BYPASS	
		WP_ALZHEIMERS_DISEASE	
		REACTOME_TRANSMISSION_ACROSS_CHEMICAL_SYNAPSES	
		WP_HEPATITIS_C_AND_HEPATOCELLULAR_CARCINOMA	
		REACTOME_SIGNALING_BY_HEDGEHOG	
		REACTOME_NUCLEAR_ENVELOPE_BREAKDOWN	
		REACTOME_AUTOPHAGY	
		REACTOME_ER_TO_GOLGI_ANTEROGRADE_TRANSPORT	
		REACTOME_HCMV_INFECTION	
		REACTOME_SCF_SKP2_MEDIATED_DEGRADATION_OF_P27_P21	
		KEGG_P53_SIGNALING_PATHWAY	
		WP_RAC1PAK1P38MMP2_PATHWAY	

Cytotoxic	ICKG_annotation (ranked by pagerank score)	ORA_annotation	LLM_annotation
CCL5	effector cells	WP_INTERACTIONS_OF_NATURAL_KILLER_CELLS_IN_PANCREATIC_CANCER	Cytotoxic T-cell Mediated Immunity (0.92)
GZMA	vaccine-mediated antibody response	WP_ALLOGRAFT_REJECTION	
CCL4	immune evasion	KEGG_ALLOGRAFT_REJECTION	
ID2	significant tumor clearance	KEGG_GRAFT_VERSUS_HOST_DISEASE	
CST7	sustained tumor control	KEGG_TYPE_I_DIABETES_MELLITUS	
GZMB	postoperative disease-free survival	HALLMARK_ALLOGRAFT_REJECTION	
GZMH	subsequent effector differentiation	KEGG_LEISHMANIA_INFECTION	
ALOX5AP	cell migration	KEGG_CELL_ADHESION_MOLECULES_CAMS	
NKG7	cross-present melanoma-derived tumor antigens	KEGG_AUTOIMMUNE_THYROID_DISEASE	
APOBEC3G	important immune functions	REACTOME_PD_1_SIGNALING	
HLA-DRB1	cytokine secretion	WP_NETWORK_MAP_OF_SARSCOV2_SIGNALING_PATHWAY	
IFNG	complete tumor eradication	KEGG_VIRAL_MYOCARDITIS	
GZMK	cell adhesion	WP_CANCER_IMMUNOTHERAPY_BY_PD1_BLOCKADE	
PRF1	decreased antigen recognition ability	REACTOME_CHEMOKINE_RECEPTORS_BIND_CHEMOKINES	
CXCR6	exhaustion markers	KEGG_ASTHMA	
LGALS1	antibody-based therapy	WP_SARSCOV2_INNATE_IMMUNITY_EVASION_AND_CELLSPECIFIC_IMMUNE_RESPONSE	
RBPJ	tumor burdens	REACTOME_MHC_CLASS_II_ANTIGEN_PRESENTATION	
GNLY	immune-edited tumors	WP_OVERVIEW_OF_PROINFLAMMATORY_AND_PROFIBROTIC_MEDIATORS	
KLRB1	cytokine/chemokine release	REACTOME_COSTIMULATION_BY_THE_CD28_FAMILY	
HOPX	critical acute myositis	REACTOME_GENERATION_OF_SECOND_MESSENGER_MOLECULES	
HLA-DPB1	cytotoxic antitumor effects	REACTOME_INTERFERON_GAMMA_SIGNALING	
AC092580.4	insulin resistance	REACTOME_SIGNALING_BY_INTERLEUKINS	
CTSD	proper anti-tumor immune responses	KEGG_INTESTINAL_IMMUNE_NETWORK_FOR_IGA_PRODUCTION	
CXCL13	co-culture systems	KEGG_CYTOKINE_CYTOKINE_RECEPTOR_INTERACTION	
CKLF	proper organ function	KEGG_CHEMOKINE_SIGNALING_PATHWAY	
HLA-DPA1	epithelial regeneration	REACTOME_PEPTIDE_LIGAND_BINDING_RECEPTORS	
CLEC2B	anti-inflammation effect	HALLMARK_COMPLEMENT	
CTSW	tumor environments	HALLMARK_TNFA_SIGNALING_VIA_NFKB	
HLA-DRA	coinhibitory receptors	WP_CYTOKINES_AND_INFLAMMATORY_RESPONSE	
CD99	keratinocyte proliferation	KEGG_SYSTEMIC_LUPUS_ERYTHEMATOSUS	
ANXA1	early tertiary lymphoid structure	WP_SELECTIVE_EXPRESSION_OF_CHEMOKINE_RECEPTORS_DURING_TCELL_POLARIZATION	
PDCD1	histone lysine methyltransferase	WP_TCELL_ACTIVATION_SARSCOV2	
CCL4L2	functional exhaustion	KEGG_ANTIGEN_PROCESSING_AND_PRESENTATION	
C12ORF75	specific cancer-related gene sets	WP_CHEMOKINE_SIGNALING_PATHWAY	
CD6	greater tumor infiltration	KEGG_TOLL_LIKE_RECEPTOR_SIGNALING_PATHWAY	
COTL1	acidic substance-related gene sets	WP_TOLLLIKE_RECEPTOR_SIGNALING_PATHWAY	
LINC00152	immune pathologies	REACTOME_IMMUNOREGULATORY_INTERACTIONS_BETWEEN_A_LYMPHOID_AND_A_NON_LYMPHOID_CELL	
ANXA2	immune checkpoint inhibitor	REACTOME_INTERLEUKIN_10_SIGNALING	
DUSP4	relapsed/refractory multiple myeloma	HALLMARK_HYPOXIA	
GZMM	cancer risk	REACTOME_INTERFERON_SIGNALING	
ANKRD28	allotransplant rejection	REACTOME_G_ALPHA_I_SIGNALLING_EVENTS	
CCL3	cancer genesis	REACTOME_NOTCH2_INTRACELLULAR_DOMAIN_REGULATES_TRANSCRIPTION	
CD63	adoptive cancer immunotherapy	REACTOME_TCR_SIGNALING	
CD96	progressive disease	REACTOME_CLASS_A_1_RHODOPSIN_LIKE_RECEPTORS	
LAG3	protective immune memory	KEGG_CYTOSOLIC_DNA_SENSING_PATHWAY	
ITGB2	fat-associated lymphoid structures	WP_EBOLA_VIRUS_INFECTION_IN_HOST	
PLIN2	cytotoxic gene upregulation	WP_TGFBETA_RECEPTOR_SIGNALING	
ZEB2	mycosis fungoides	KEGG_NATURAL_KILLER_CELL_MEDIATED_CYTOTOXICITY	
JUN	durable tumor immunity	WP_TCELL_ANTIGEN_RECEPTOR_TCR_PATHWAY_DURING_STAPHYLOCOCCUS_AUREUS_INFECTION	
BHLHE40	cancer-related anorexia	WP_TGFBETA_RECEPTOR_SIGNALING_IN_SKELETAL_DYSPLASIAS	
	monocyte activation	WP_LUNG_FIBROSIS	
	cancer-targeting immune cells	WP_MIRNAS_INVOLVEMENT_IN_THE_IMMUNE_RESPONSE_IN_SEPSIS	
	known cancer-associated antigens	REACTOME_METABOLISM_OF_ANGIOTENSINOGEN_TO_ANGIOTENSINS	
	immune resistance mechanisms	WP_GLUCOCORTICOID_RECEPTOR_PATHWAY	
	immune resistance archetypes	WP_CYTOSOLIC_DNASENSING_PATHWAY	
	cancer cell eradication	WP_PATHOGENESIS_OF_SARSCOV2_MEDIATED_BY_NSP9NSP10_COMPLEX	
	cellular proliferation	WP_IL18_SIGNALING_PATHWAY	
	cytokine-mediated cancer immunotherapy	WP_APOPTOSIS	
	durable tumor remission	WP_HAIR_FOLLICLE_DEVELOPMENT_CYTODIFFERENTIATION_PART_3_OF_3	
	efficacious suppression	REACTOME_GPCR_LIGAND_BINDING	
	established signal transduction pathways	HALLMARK_IL2_STAT5_SIGNALING	
	relapsed/refractory hematological malignancies	REACTOME_MAPK_TARGETS_NUCLEAR_EVENTS_MEDIATED_BY_MAP_KINASES	
	anti-leukemia immunosurveillance	WP_CIRCADIAN_RHYTHM_GENES	
	systemic antitumor immunity	WP_DEVELOPMENT_AND_HETEROGENEITY_OF_THE_ILC_FAMILY	
	local bystander activation	REACTOME_SIGNALING_BY_NOTCH2	
	innate-like protection	WP_PROSTAGLANDIN_SIGNALING	
	innate-like memory	KEGG_T_CELL_RECEPTOR_SIGNALING_PATHWAY	
	alloimmune response	REACTOME_GENE_AND_PROTEIN_EXPRESSION_BY_JAK_STAT_SIGNALING_AFTER_INTERLEUKIN_12_STIMULATION	
	autoimmune hepatitis	REACTOME_SMOOTH_MUSCLE_CONTRACTION	
	lineage-specific gene programs	KEGG_LYSOSOME	
	disease-tolerance defenses	WP_INTERACTIONS_BETWEEN_IMMUNE_CELLS_AND_MICRORNAS_IN_TUMOR_MICROENVIRONMENT	
	correlation immunotherapy response	WP_TNFRELATED_WEAK_INDUCER_OF_APOPTOSIS_TWEAK_SIGNALING_PATHWAY	
	efficient tumor-immune responses	WP_PROSTAGLANDIN_SYNTHESIS_AND_REGULATION	
	tumor milieu		
	glioblastoma immunotherapy		
	immune system balance		
	parietal cell loss		
	progression-free interval		
	disease-specific survival		
	targeted single-gene perturbation		
	genetic circuits		
	recurrent clonal expansion		
	responsive hot tumors		
	immune landscape		
	treatment-associated tumor necrosis		
	local tissue protection		
	combination immunotherapy		
	tumor evolution		
	checkpoint-based immunotherapy		
	reduced terminal exhaustion		
	increased memory cell characteristics		
	perihilar large duct type		
	epigenetic reprogramming		
	cytotoxic immune responses		
	ordered immune hierarchies		
	adaptive cancer immunotherapy		
	metabolic exhaustion		
	aberrant phosphorylation		
	advanced cancer		
	cellular therapeutics		
	diverse cancers		
	cancer chemotherapy clinical trials		
	tumor clonal dynamics		
	genetic heterogeneity		
	immune edited tumors		
	dense tumor infiltration		
	post allogeneic hematopoietic stem cell transplantation		
	neoantigen-reactive clonotypes		
	synthetic immune cell therapies		
	significant tumor growth inhibition		
	leukemia-like disease		
	tumor microenvironments		
	intratumour heterogeneity		
	tumour evolution		
	cancer immunoprevention		
	monocyte differentiation		
	organ-adapted protection		
	normal angiogenesis		
	gene delivery		
	lymph node homing capability		
	intrahepatic cholangiocarcinoma		
	aging-associated diseases		
	brain-preserving immune suppression		
	tumor-directed immune activation		
	excessive neuroinflammation		
	tumor hypoxia		
	gene fusions		
	liver cancer tissues		
	cancer-derived immune suppression		
	immune cell distribution		
	oxidative stress/damage		
	immune checkpoint inhibitor therapy		
	late-stage breast cancer		
	breast tumor growth		
	reduced antigen presentation		
	disease hyperprogression		
	myeloid composition		
	myeloid-cell identity		
	clinical cancer vaccines		
	tumor-specific immune tolerance		
	delayed immune reconstitution		
	cancer occurrence		
	disseminated cancer cells		
	inflammation-related cancers		
	immune suppressive microenvironment buildup		
	acquired tumor resistance		
	therapeutic innovation		
	bulky metastatic disease		
	tumour-immune interactions		
	resulting neoantigen generation		
	tumour promotion		
	cancer-associated immune dysfunction		
	liver tumor dynamics		
	primary/distal tumor growth		
	lung cancer resident		
	specific antitumor adaptive immunity		
	anergy induction		
	cellular exhaustion		
	innate immune lineages		
	allogeneic cell cancer immunotherapy		
	immune resistance pathways		
	cancer models		
	immune tolerance mechanism		
	high-risk non-muscle-invasive bladder cancer		
	tumor-immune system models		
	tumor-bearing patients		
	tumor intrinsic mechanisms		
	augment anti-tumor immune responses		
	key immune surveillance sites		
	advanced gynecologic cancer		
	innovative cancer treatment		
	effective anti-cancer immunity		
	young adult cancers		
	non-viral genetic modification		
	mutual intercommunication		
	paediatric cancer care		
	drug-resistance		
	anti-cancer adaptive immunity		
	disease propagation		
	multiple cancer types		
	immune desert subtypes		
	positive prognosis		
	immune-checkpoint proteins		
	residual tumor cells		
	solid tumour cancers		
	compromised immune function		
	immune predation		
	neo-open reading frame peptides		
	childhood solid cancers		
	fetal resorption		
	multiple immune populations		
	engineered immune cell therapies		
	breast cancer-related mortality		
	tumor immune microenvironment phenotypes		
	spontaneous regression		
	immuno-oncology targets		
	antibody function		
	autoimmune reactions		
	tumor-infiltrating immunosuppressive leukocytes		
	excessive immune response		
	mixed inflammation		
	metastasis-free stages		
	lysine-9 methyl-transferase		
	comprehensive pan-cancer analysis		
	methylation landscape		
	endogenous adaptive antitumour immunity		
	post-ablation immune therapy		
	tumour immune escape		
	tumor-specific-stereotyped sequences		
	tumor immune environment		
	immune-regulatory proteins		

Dysfunction	ICKG_annotation (ranked by pagerank score)	ORA_annotation	LLM_annotation
NR3C1	cancer remission	WP_GLUCOCORTICOID_RECEPTOR_PATH	Immune Response Regulation and Signal Transduction (0.85)
CXCL13	antitumor immune responses	WP_ENDODERM_DIFFERENTIATION	
FKBP5	anti-tumor immunity		
ITM2A	myeloid cells		
RNF19A	anti-tumor function		
ICA1	complete tumor eradication		
SH2D1A	subsequent differentiation		
TIGIT	liver tumor progression		
PPP1CC	tumor amino acid metabolism		
IL6ST	autoimmune disease		
LIMS1	cancer cell growth		
NMB	effector population		
TSHZ2	tumor escape		
KLRB1	gastric cancer pathogenesis		
PDCD1	cancer heterogeneity		
TOX2	critical acute myositis		
MAGEH1	cancer immunosurveillance		
SMCO4	tumour-promoting functions		
ALOX5AP	early tertiary lymphoid structure		
FABP5	striking tumor regression		
MAF	cellular immune responses		
RBPJ	local tumor control		
NAP1L4	pancreatic cancer growth		
SLA	histone lysine methyltransferase		
TOX	gastric cancer immunotherapy		
BTLA	exhaustion markers		
CD200	ischemia reperfusion injury-related phenotypes		
CPM	long-term protective immunity		
RGS2	cancer cell extravasation		
SESN1	gamma interferon		
CD84	liver metastasis formation		
PASK	tumour suppression		
TBC1D4	ectopic accumulation		
THADA	cancer cell transmigration		
AKAP13	effector function inhibition		
C9ORF16	spontaneous lung cancer development		
CD40LG	early cancer development		
CHI3L2	lung carcinogenesis		
CHN1	immune-mediated acute liver injury		
DUSP4	anti-inflammation effect		
YWHAQ	successful tumor rejection		
CORO1B	cancer cell death		
COTL1	immune system balance		
TCF7	combination immunotherapy		
TNFRSF18	personalized cancer vaccination strategies		
PCAT29	tertiary lymphoid structure-like lymphocytic aggregates		
PVALB	natural killer cell activity		
SFXN1	intra-pancreatic inoculation		
NUDT16	focal cell death		
AHI1	intratumor microbiota		
	cellular proliferation		
	cell proliferation-inhibiting bystander effects		
	human lung epithelial development		
	metabolic dysfunction-associated steatohepatitis		
	gastric adenocarcinoma tissues		
	cancer formation		
	tumor growth dynamics		
	cancer burden		
	cytokine-mediated cancer immunotherapy		
	cardiac xenotransplantation		
	melanoma metastases		
	robust transduction		
	myeloid cell infiltration		
	irreversible cirrhosis		
	systemic antitumor immunity		
	leukemia/lymphoma conditions		
	increased fibrosis		
	human immune function		
	immune cell recruitment		
	reduced terminal exhaustion		
	increased memory cell characteristics		
	extracellular matrix proteins		
	adaptive anti-tumor immunity		
	dense tumor infiltration		
	endogenous adaptive antitumour immunity		
	post-ablation immune therapy		
	cervical cancers		
	chronic obstructive pulmonary disease		
	cystic fibrosis		
	cell-cycle progression		
	significant tumor growth inhibition		
	ovarian pathogenesis		
	myocardial necrosis		
	immuno-oncology targets		
	antibody function		
	spontaneous regression		
	autoimmune reactions		
	tumor-infiltrating immunosuppressive leukocytes		
	excessive immune response		
	mixed inflammation		
	lymphocyte activatory receptors		
	lysine-9 methyl-transferase		
	antitumor functions		
	host antitumor immunity		
	anti-tumor dysfunction		
	oncogenic viruses		

Interferon	ICKG_annotation (ranked by pagerank score)	ORA_annotation	LLM_annotation
ISG15	immunogenic cell death	HALLMARK_INTERFERON_GAMMA_RESPONSE	Antiviral Interferon Response (0.92)
IFIT1	myeloid cells	HALLMARK_INTERFERON_ALPHA_RESPONSE	
MX1	anti-tumor immunity	REACTOME_INTERFERON_SIGNALING	
RSAD2	cell-mediated immunity	REACTOME_INTERFERON_ALPHA_BETA_SIGNALING	
IFI44L	insulin sensitivity	REACTOME_ANTIVIRAL_MECHANISM_BY_IFN_STIMULATED_GENES	
CMPK2	adaptive immune response	WP_TYPE_I_INTERFERON_INDUCTION_AND_SIGNALING_DURING_SARSCOV2_INFECTION	
HERC5	tumour cell death	WP_TYPE_II_INTERFERON_SIGNALING_IFNG	
IFIT2	programmed cell death	WP_IMMUNE_RESPONSE_TO_TUBERCULOSIS	
IFIT3	established immune responses	WP_HOSTPATHOGEN_INTERACTION_OF_HUMAN_CORONAVIRUSES_INTERFERON_INDUCTION	
IRF7	antitumor response	REACTOME_INTERFERON_GAMMA_SIGNALING	
MX2	tumor antigens	WP_MEASLES_VIRUS_INFECTION	
OAS1	immune-related toxicity	WP_SARSCOV2_INNATE_IMMUNITY_EVASION_AND_CELLSPECIFIC_IMMUNE_RESPONSE	
OASL	antitumor efficacy	WP_NONGENOMIC_ACTIONS_OF_125_DIHYDROXYVITAMIN_D3	
EIF2AK2	immune-mediated anti-tumor immunity	WP_NETWORK_MAP_OF_SARSCOV2_SIGNALING_PATHWAY	
OAS2	cross presentation	REACTOME_NEGATIVE_REGULATORS_OF_DDX58_IFIH1_SIGNALING	
SAMD9	abscopal anticancer activity	REACTOME_MATURATION_OF_SARS_COV_1_NUCLEOPROTEIN	
USP18	long-term cell-mediated anti-tumor immunity	REACTOME_DDX58_IFIH1_MEDIATED_INDUCTION_OF_INTERFERON_ALPHA_BETA	
GBP1	immuno-metabolic regulation	REACTOME_MATURATION_OF_SARS_COV_2_NUCLEOPROTEIN	
IFI6	ankylosing spondylitis	REACTOME_NICOTINAMIDE_SALVAGING	
ISG20	anti-cancer immune responses	REACTOME_REGULATION_OF_IFNA_SIGNALING	
PPM1K	cell cycle regulatory genes	WP_CYTOSOLIC_DNASENSING_PATHWAY	
SAMD9L	effector function capabilities	REACTOME_TRANSLATION_OF_SARS_COV_1_STRUCTURAL_PROTEINS	
STAT1	immune-mediated acute liver injury	REACTOME_NICOTINATE_METABOLISM	
TRIM22	antibody-based immunotherapy	WP_SARS_CORONAVIRUS_AND_INNATE_IMMUNITY	
IFI44	antibody-based constructs	REACTOME_SARS_COV_INFECTIONS	
MT2A	cancer immunology	WP_TYPE_III_INTERFERON_SIGNALING	
NT5C3A	vivo cytotoxicity	HALLMARK_INFLAMMATORY_RESPONSE	
OAS3	cancer-targeted photoimmunotherapy	REACTOME_TRANSLATION_OF_SARS_COV_2_STRUCTURAL_PROTEINS	
IFI35	massive tumor cell death	REACTOME_SARS_COV_1_INFECTION	
TNFSF10	basal cells	WP_IL10_ANTIINFLAMMATORY_SIGNALING_PATHWAY	
UBE2L6	tumour-elicited immune responses	KEGG_CYTOSOLIC_DNA_SENSING_PATHWAY	
XAF1	human lung epithelial development	WP_EBOLA_VIRUS_INFECTION_IN_HOST	
EPSTI1	immune synapse formation	REACTOME_TRAF3_DEPENDENT_IRF_ACTIVATION_PATHWAY	
LY6E	transient cytokine release	WP_NOVEL_INTRACELLULAR_COMPONENTS_OF_RIGILIKE_RECEPTOR_RLR_PATHWAY	
PARP9	myeloid cell infiltration	WP_PATHWAYS_OF_NUCLEIC_ACID_METABOLISM_AND_INNATE_IMMUNE_SENSING	
PLSCR1	cancer evolution	KEGG_RIG_I_LIKE_RECEPTOR_SIGNALING_PATHWAY	
ZBP1	tumor evolution factors	REACTOME_SARS_COV_2_INFECTION	
HELZ2	mechanism-based cancer immunotherapy	WP_HEPATITIS_B_INFECTION	
PARP14	strong antitumor efficacy	REACTOME_INTERLEUKIN_20_FAMILY_SIGNALING	
RBCK1	signaling	WP_PDGFRBETA_PATHWAY	
SHFL	immune status	REACTOME_TRAF6_MEDIATED_IRF7_ACTIVATION	
SP110	cancer immunotherapy response	REACTOME_TERMINATION_OF_TRANSLESION_DNA_SYNTHESIS	
STAT2	hyperprogressive disease	WP_MITOCHONDRIAL_IMMUNE_RESPONSE_TO_SARSCOV2	
DDX58	suppress antitumor immune responses	WP_OVERVIEW_OF_INTERFERONSMEDIATED_SIGNALING_PATHWAY	
HSH2D	tumor size	REACTOME_TRANSLESION_SYNTHESIS_BY_Y_FAMILY_DNA_POLYMERASES_BYPASSES_LESIONS_ON_DNA_TEMPLATE	
PALM2-AKAP2	inhibitory immune cells	REACTOME_NS1_MEDIATED_EFFECTS_ON_HOST_PATHWAYS	
PARP10	left-sided malignant epithelia	REACTOME_METABOLISM_OF_WATER_SOLUBLE_VITAMINS_AND_COFACTORS	
RTP4	damaging immune responses		
CHST12	major immune suppression		
LAP3	superior tumor reduction		
	tumor-specific immune-therapeutics		
	intratumoral neurodegeneration		
	cytokine-mediated toxicities		
	reduced pathological inflammation		
	immune escape mechanism		
	metabolic regulation		
	cancer microvessels		
	peripheral immune tolerance		
	multiple immune checkpoints		
	negative selection		
	mycosis fungoides		
	stromal cell support		
	extracellular vesicles		
	facilitated clonal expansion		
	central immune tolerance		
	tumor-mediated immune escape		
	enhanced tumor immune evasion		
	antitumor phenotypes		
	synergistic regression		
	repertoire selection		
	transendothelial migration		
	negative feedback regulation		
	adaptive immune function		
	anti-tumor therapeutics		
	thymus function		
	interferon regulatory factor 1 transcriptional activation		
	bone-metastatic castration-resistant prostate cancer		
	transient peripheral transcriptomic alterations		
	cellular efflux		
	unique immune microenvironments		
	anti-tumour efficacy		
	proinflammatory immune responses		
	heterosubtypic protection		
	significant antitumor activity		
	potent antitumor activity		
	increased antitumor immunity		
	superior tumor elimination		

Naive2	ICKG_annotation (ranked by pagerank score)	ORA_annotation	LLM_annotation
CCR7	tumor regression	REACTOME_REPRESSION_OF_WNT_TARGET_GENES	T-cell Activation and Differentiation (0.85)
LEF1	cell-associated viral transcripts	KEGG_ACUTE_MYELOID_LEUKEMIA	
MAL	cancer immune regulation	REACTOME_CA2_PATHWAY	
SELL	human immunodeficiency virus	KEGG_THYROID_CANCER	
KLF2	master regulator genes	WP_HAIR_FOLLICLE_DEVELOPMENT_ORGANOGENESIS_PART_2_OF_3	
MYC	anti-tumor efficacy	WP_WNT_SIGNALING_PATHWAY_AND_PLURIPOTENCY	
NOSIP	antitumor properties	HALLMARK_WNT_BETA_CATENIN_SIGNALING	
C6ORF48	protein expression	WP_WNT_SIGNALING	
PLAC8	improved antitumor activity	WP_MAMMARY_GLAND_DEVELOPMENT_PATHWAY_INVOLUTION_STAGE_4_OF_4	
PRKCQ-AS1	cancer risk	KEGG_ENDOMETRIAL_CANCER	
SNORA76	anti-tumour immune responses	WP_WNT_SIGNALING_PATHWAY	
TCF7	antigen-presenting neutrophil delivery	WP_MAMMARY_GLAND_DEVELOPMENT_PATHWAY_PUBERTY_STAGE_2_OF_4	
TRAT1	hyperinducible expression	REACTOME_BETA_CATENIN_INDEPENDENT_WNT_SIGNALING	
APEX1	anti-tumorigenic innate immune cells	KEGG_COLORECTAL_CANCER	
C1ORF162	anti-tumor innate immunity	WP_ENDOMETRIAL_CANCER	
GIMAP5	long-term antitumor memory	KEGG_WNT_SIGNALING_PATHWAY	
LINC00861	vivo oncogenic function	WP_AMPLIFICATION_AND_EXPANSION_OF_ONCOGENIC_PATHWAYS_AS_METASTATIC_TRAITS	
PRKCA	glioma cell progression	KEGG_ARRHYTHMOGENIC_RIGHT_VENTRICULAR_CARDIOMYOPATHY_ARVC	
RSL1D1	cell cycle regulatory genes	WP_CHROMOSOMAL_AND_MICROSATELLITE_INSTABILITY_IN_COLORECTAL_CANCER	
CCDC109B	effector function capabilities	WP_ARRHYTHMOGENIC_RIGHT_VENTRICULAR_CARDIOMYOPATHY	
EPB41L4A-AS1	altered phosphorylation ratios	WP_MAMMARY_GLAND_DEVELOPMENT_PATHWAY_EMBRYONIC_DEVELOPMENT_STAGE_1_OF_4	
FAM65B	immunogenic cancer cell death	KEGG_SMALL_CELL_LUNG_CANCER	
GIMAP1	anti-tumour immunity	KEGG_PATHWAYS_IN_CANCER	
GIMAP2	intratumoral macrophages	WP_IMATINIB_AND_CHRONIC_MYELOID_LEUKEMIA	
ICAM2	glioma cell population	REACTOME_DEGRADATION_OF_BETA_CATENIN_BY_THE_DESTRUCTION_COMPLEX	
ITGB1	antitumor treatment	REACTOME_IMMUNOREGULATORY_INTERACTIONS_BETWEEN_A_LYMPHOID_AND_A_NON_LYMPHOID_CELL	
KLRB1	complete tumour regression	REACTOME_FORMATION_OF_THE_BETA_CATENIN_TCF_TRANSACTIVATING_COMPLEX	
LPAR6	protective immune memory	WP_NCRNAS_INVOLVED_IN_WNT_SIGNALING_IN_HEPATOCELLULAR_CARCINOMA	
RASGRP2	cervical cancer malignancy	HALLMARK_INFLAMMATORY_RESPONSE	
SATB1	anti-tumoral immunity	REACTOME_TRANSCRIPTIONAL_REGULATION_BY_RUNX3	
SH3BP5	immune-based cancer therapies	WP_SMALL_CELL_LUNG_CANCER	
ST13	optimal primary expansion	WP_WNTBETACATENIN_SIGNALING_PATHWAY_IN_LEUKEMIA	
TIMP1	sensory ablation	WP_COMPLEMENT_SYSTEM	
TMEM243	protective antitumor immune responses	WP_HIPPO_SIGNALING_REGULATION_PATHWAYS	
TRABD2A	denervation-dependent antitumor effects	KEGG_MELANOGENESIS	
FHIT	effective antitumor immune responses	REACTOME_SYNDECAN_INTERACTIONS	
CD27	antibody dynamics	WP_LNCRNA_IN_CANONICAL_WNT_SIGNALING_AND_COLORECTAL_CANCER	
TAGAP	tumor-penetrating antibodies	REACTOME_INTERLEUKIN_4_AND_INTERLEUKIN_13_SIGNALING	
ALKBH7	anticancer immunosurveillance	WP_DNA_DAMAGE_RESPONSE_ONLY_ATM_DEPENDENT	
FOXP1	mediate anticancer immunity	WP_GASTRIN_SIGNALING_PATHWAY	
SNHG7	lymphocyte fate determination	WP_GASTRIC_CANCER_NETWORK_2	
RIC3	immune cell fate	WP_SPINAL_CORD_INJURY	
IL6ST	lactic acid accumulation	KEGG_CELL_ADHESION_MOLECULES_CAMS	
TSHZ2	disease biology	WP_INFLUENCE_OF_LAMINOPATHIES_ON_WNT_SIGNALING	
SMDT1	systemic immune checkpoint inhibitors	REACTOME_DEACTIVATION_OF_THE_BETA_CATENIN_TRANSACTIVATING_COMPLEX	
EIF2S3	higher mutation load	WP_IL6_SIGNALING_PATHWAY	
PIM2	humoral adaptive immune responses		
PRMT2	anti-tumor function		
CREBRF	lactic acid secretion		
SORL1	sustained protein translation		
	lasting protective immune responses		
	marrow-residence genes		
	autoimmune hepatitis		
	anti-tumor adaptive immune responses		
	lymphoid cell fate		
	high-affinity antibodies		
	lineage-specific gene programs		
	colorectal carcinoma		
	vaccine-mediated antibody response		
	robust immune responses		
	transplantable mouse tumor models		
	skin rashes		
	type 2/type 17 immune responses		
	local tissue protection		
	long-lived autoreactive cells		
	retarded tumor progression		
	acute inflammation		
	appropriate immune responses		
	immune landscape		
	cell-cycle progression		
	memory cell differentiation		
	human immune homeostasis		
	post allogeneic hematopoietic stem cell transplantation		
	lymph node homing capability		
	cancer immunoprevention		
	monocyte differentiation		
	organ-adapted protection		
	relapsed/refractory hematologic malignancies		
	sustained antitumor activity		
	immunomodulation process		
	intact tumor-cell antigen presentation		
	mammary tumorigenesis		
	short-term immunologic response		
	long-term clinical outcomes		
	autoimmune disease treatment		
	higher antitumor activity		
	proper organ function		
	enhanced immune cell recruitment		
	low grade glioma		
	keratinocyte proliferation		
	breast cancer stem cell-associated signaling pathways		

Glycolysis_MYC	CKG_annotation (ranked by pagerank score	ORA_annotation	LLM_annotation
NME1	cell cycle	HALLMARK_MYC_TARGETS_V1	Protein Synthesis and Cellular Energy Production (0.85)
RANBP1	peripheral neuropathy	HALLMARK_E2F_TARGETS	
CCT5	anti-tumor immunity	REACTOME_METABOLISM_OF_NUCLEOTIDES	
EIF5A	tumoricidal factors	HALLMARK_MYC_TARGETS_V2	
FABP5	anti-tumor function	REACTOME_INTERCONVERSION_OF_NUCLEOTIDE_DI_AND_TRIPHOSPHATES	
C1QBP	cytotoxic extracellular vesicles	REACTOME_FOLDING_OF_ACTIN_BY_CCT_TRIC	
NHP2	systemic toxicity	WP_METHIONINE_DE_NOVO_AND_SALVAGE_PATHWAY	
PDCD5	critical immune factors	HALLMARK_IL2_STAT5_SIGNALING	
PPA1	systemic immunotoxicity	REACTOME_BBSOME_MEDIATED_CARGO_TARGETING_TO_CILIUM	
PRMT1	cancer relapse	REACTOME_FORMATION_OF_TUBULIN_FOLDING_INTERMEDIATES_BY_CCT_TRIC	
SRM	cancer-related mortality	REACTOME_TNFS_BIND_THEIR_PHYSIOLOGICAL_RECEPTORS	
TNFRSF4	striking tumor regression	WP_16P112_PROXIMAL_DELETION_SYNDROME	
IL2RA	protein folding	REACTOME_COOPERATION_OF_PREFOLDIN_AND_TRIC_CCT_IN_ACTIN_AND_TUBULIN_FOLDING	
SMS	local tumor control	REACTOME_COOPERATION_OF_PDCL_PHLP1_AND_TRIC_CCT_IN_G_PROTEIN_BETA_FOLDING	
ATP5G1	malignant tumor immunity	REACTOME_ASSOCIATION_OF_TRIC_CCT_WITH_TARGET_PROTEINS_DURING_BIOSYNTHESIS	
DUT	optimal primary expansion	WP_METABOLIC_REPROGRAMMING_IN_COLON_CANCER	
PKM	immune checkpoint dysfunction	REACTOME_RUNX1_AND_FOXP3_CONTROL_THE_DEVELOPMENT_OF_REGULATORY_T_LYMPHOCYTES_TREGS	
TIMM13	immune-regulation-related signaling pathwa	HALLMARK_MTORC1_SIGNALING	
TNFRSF18	post-treatment relapse	REACTOME_CARGO_TRAFFICKING_TO_THE_PERICILIARY_MEMBRANE	
TXNDC17	immune status	REACTOME_MRNA_SPLICING_MINOR_PATHWAY	
DCTPP1	longer progression-free survival	KEGG_SPLICEOSOME	
DDX21	low-risk subgroups	KEGG_RNA_DEGRADATION	
HSPD1	tumor heterogeneity	WP_MRNA_PROCESSING	
MRPL12	relapsed/refractory acute myeloid leukemia	REACTOME_MRNA_DECAY_BY_5_TO_3_EXORIBONUCLEASE	
NOLC1	cytotoxic antitumor effects	REACTOME_MITOCHONDRIAL_PROTEIN_IMPORT	
PGAM1	immune-tumor cell-cell interactions	REACTOME_TP53_REGULATES_TRANSCRIPTION_OF_GENES_INVOLVED_IN_G2_CELL_CYCLE_ARREST	
PHB	melanoma proliferation	KEGG_CYTOKINE_CYTOKINE_RECEPTOR_INTERACTION	
SNRPD1	sialic acid-binding immunoglobulin-like lecti	WP_UREA_CYCLE_AND_METABOLISM_OF_AMINO_GROUPS	
TUBB	hematopoietic stem cell transplantation	KEGG_BETA_ALANINE_METABOLISM	
EBNA1BP2	cancer diagnosis	KEGG_MISMATCH_REPAIR	
IL1R2	relapsed acute myeloid leukemia	REACTOME_CYTOSOLIC_TRNA_AMINOACYLATION	
PAICS	transplant-related morbidity	WP_PYRIMIDINE_METABOLISM	
PCNA	established signal transduction pathways	REACTOME_DEFECTIVE_INTRINSIC_PATHWAY_FOR_APOPTOSIS	
PRDX1	epigenetic alterations	REACTOME_TRANSLATION	
SNRPE	cancer-colonized organs	REACTOME_ORGANELLE_BIOGENESIS_AND_MAINTENANCE	
CCND2	leukemic-cell resistance	WP_AMINO_ACID_METABOLISM	
CCT2	skin rashes	REACTOME_MRNA_SPLICING	
CCT3	type 2/type 17 immune responses	REACTOME_PROTEIN_FOLDING	
EEF1E1	peripheral blood immune system	HALLMARK_ALLOGRAFT_REJECTION	
LSM2	autoimmune-mediated neuroinflammation	REACTOME_TNFR2_NON_CANONICAL_NF_KB_PATHWAY	
LSM5	immune cytolytic stress	REACTOME_CILIUM_ASSEMBLY	
MRPL14	endogenous tumor immunity		
NDUFAB1	mycosis fungoides		
NDUFB3	treatment-associated tumor necrosis		
TNFRSF9	stromal cell support		
TXN	tumor-intrinsic fatty acid Œ≤-oxidation		
SSBP1	lasting tumor regression		
DNPH1	complete eradication		
TOMM40	monocyte-macrophage diversity		
EIF5B	memory cell differentiation		
	lymphocyte aggregation		
	subsequent differentiation		
	engulf cancer cells		
	immunomodulation process		
	intact tumor-cell antigen presentation		
	lineage-specific gene programs		
	immune-related gene signatures		
	short-term immunologic response		
	long-term clinical outcomes		
	autoimmune disease treatment		
	higher antitumor activity		
	1911 cases		
	cancer immune escape		
	retrovirus immunity		
	lymphocyte activatory receptors		
	unfolded proteins		
	higher basal respiration		
	coagulation cascade signals		

Unassigned	KG_annotation (ranked by pagerank scor	ORA_annotation	LLM_annotation
ARPC4	peripheral neuropathy	REACTOME_RHO_GTPASES_ACTIVATE_WASPS_AND_WAVES	Cytoskeleton organization and immune response (0.85)
CKLF	tumor regression	WP_PROSTAGLANDIN_SYNTHESIS_AND_REGULATION	
LGALS1	cytotoxic extracellular vesicles	WP_PATHOGENIC_ESCHERICHIA_COLI_INFECTION	
ALOX5AP	systemic toxicity	KEGG_PATHOGENIC_ESCHERICHIA_COLI_INFECTION	
CAP1	memory cell differentiation	REACTOME_NEUTROPHIL_DEGRANULATION	
LTB	long-term tumor clearance	REACTOME_GENE_AND_PROTEIN_EXPRESSION_BY_JAK_STAT_SIGNALING_AFTER_INTERLEUKIN_12_STIMULATION	
S100A4	hepatocyte proliferation	REACTOME_EPHB_MEDIATED_FORWARD_SIGNALING	
CORO1B	cytotoxic antitumor effects	REACTOME_INTERLEUKIN_12_SIGNALING	
EMP3	interferon gamma	HALLMARK_COMPLEMENT	
LY6E	epithelial regeneration	HALLMARK_INTERFERON_GAMMA_RESPONSE	
PKM	anti-inflammation effect	HALLMARK_REACTIVE_OXYGEN_SPECIES_PATHWAY	
ANXA6	gene therapy	REACTOME_PARASITE_INFECTION	
ARPC1B	cellular immune responses	KEGG_REGULATION_OF_ACTIN_CYTOSKELETON	
ARPC5	cell division	REACTOME_DISSOLUTION_OF_FIBRIN_CLOT	
C9ORF16	tissue-specific splicing	REACTOME_INTERLEUKIN_12_FAMILY_SIGNALING	
DBI	exhaustion markers	REACTOME_FCGAMMA_RECEPTOR_FCGR_DEPENDENT_PHAGOCYTOSIS	
LSP1	cancer cell death		
PRELID1	tumoricidal factors		
TRAPPC1	cell proliferation-inhibiting bystander effects		
ANXA2	vivo oncogenic function		
APRT	altered phosphorylation ratios		
ATP5J	reconstitution		
CLIC1	cell-based therapies		
COTL1	immune cytolytic stress		
ISG15	established signal transduction pathways		
KLRB1	memory-like responses		
LCP1	tumor-intrinsic fatty acid Œ≤-oxidation		
LGALS3	cytomegalovirus viremia		
MT2A	de novo immune responses		
PARK7	responsive hot tumors		
PSME2	reduced terminal exhaustion		
S100A10	increased memory cell characteristics		
SEC61G	clonal expansion		
UCP2	dense tumor infiltration		
BRK1	adaptive anti-tumor immunity		
ID2	treatment-associated tumor necrosis		
COPS9	lineage-specific gene programs		
PPP1CA	perihilar large duct type		
CORO1A	lymph node homing capability		
TXN	myocardial necrosis		
TSPO	intrahepatic cholangiocarcinoma		
IFI27L2	tissue resident memory		
LCK	interstitial lung disease		
NEDD8	high antitumor efficacy		
PRDX1	immunoglobulin deficiency		
DAD1	oncogenic viruses		
APOBEC3G	tumor-specific-stereotyped sequences		
YWHAQ	long-term tumor remission		
GZMA			
ANXA5			

Stress_HSP	ICKG_annotation (ranked by pagerank score)	ORA_annotation	LLM_annotation
DNAJB1	immune evasion	REACTOME_ATTENUATION_PHASE	Protein Folding and Stress Response (0.85)
FKBP4	anti-tumor immunity	REACTOME_HSF1_ACTIVATION	
DNAJA4	immune modulation	REACTOME_HSF1_DEPENDENT_TRANSACTIVATION	
HSPA1B	anti-tumor function	REACTOME_CELLULAR_RESPONSE_TO_HEAT_STRESS	
HSPD1	multiple myeloma	REACTOME_REGULATION_OF_HSF1_MEDIATED_HEAT_SHOCK_RESPONSE	
HSPH1	immune system dynamics	HALLMARK_TNFA_SIGNALING_VIA_NFKB	
ZFAND2A	immune infiltration evaluations	REACTOME_HSP90_CHAPERONE_CYCLE_FOR_STEROID_HORMONE_RECEPTORS_SHR_IN_THE_PRESENCE_OF_LIGAND	
AHSA1	late-stage disease progression	HALLMARK_APOPTOSIS	
BAG3	co-stimulatory profiles	WP_NUCLEAR_RECEPTORS_METAPATHWAY	
CACYBP	tailored maturation	HALLMARK_UV_RESPONSE_UP	
DEDD2	reduced tumor burden	WP_GLUCOCORTICOID_RECEPTOR_PATHWAY	
HSP90AA1	hepatocyte proliferation	HALLMARK_MTORC1_SIGNALING	
HSPA1A	epithelial regeneration	KEGG_ANTIGEN_PROCESSING_AND_PRESENTATION	
HSPA6	adverse prognosis	KEGG_MAPK_SIGNALING_PATHWAY	
HSPE1	interferon gamma	WP_APOPTOSISRELATED_NETWORK_DUE_TO_ALTERED_NOTCH3_IN_OVARIAN_CANCER	
MRPL18	tumor environments	WP_IL18_SIGNALING_PATHWAY	
SERPINH1	anti-inflammation effect	REACTOME_CHAPERONE_MEDIATED_AUTOPHAGY	
SQSTM1	gene therapy	HALLMARK_MYC_TARGETS_V1	
CLK1	disease outcome	REACTOME_SIGNALING_BY_NUCLEAR_RECEPTORS	
DNAJA1	cancer immune surveillance	REACTOME_RHOBTB2_GTPASE_CYCLE	
DNAJB4	striking tumor regression	WP_CHROMOSOMAL_AND_MICROSATELLITE_INSTABILITY_IN_COLORECTAL_CANCER	
DOK2	tumor immune status	WP_NRF2_PATHWAY	
DUSP1	cell survival	REACTOME_SIGNALING_BY_INTERLEUKINS	
HSPB1	functional exhaustion	WP_MAPK_SIGNALING_PATHWAY	
ID2	local tumor control	REACTOME_RHOBTB_GTPASE_CYCLE	
PLIN2	heterogeneous genomic aberrations	WP_APOPTOSIS_MODULATION_AND_SIGNALING	
REL	precision immunotherapy	WP_IL6_SIGNALING_PATHWAY	
RGS2	subsequent immune infiltration	REACTOME_INTERLEUKIN_4_AND_INTERLEUKIN_13_SIGNALING	
TCP1	noncancer immune responses	WP_ARYL_HYDROCARBON_RECEPTOR_PATHWAY_WP2873	
TSPYL2	increased cellular proliferation	HALLMARK_EPITHELIAL_MESENCHYMAL_TRANSITION	
JUN	cancer defense mechanisms	WP_PHOTODYNAMIC_THERAPYINDUCED_AP1_SURVIVAL_SIGNALING	
PPP1R15A	cancer-related impairment	WP_VEGFAVEGFR2_SIGNALING_PATHWAY	
JUNB	impaired immune response	HALLMARK_TGF_BETA_SIGNALING	
HSP90AB1	bone disease	REACTOME_ESR_MEDIATED_SIGNALING	
MYLIP	cellular immune activation	KEGG_SPLICEOSOME	
TRA2B	potent complement-dependent cytotoxic effects	WP_IL1_SIGNALING_PATHWAY	
CHORDC1	bispecific antibody-mediated antitumour activity	REACTOME_NEUTROPHIL_DEGRANULATION	
SOCS3	interferon regulatory factor 1 transcriptional activat	WP_MEASLES_VIRUS_INFECTION	
BTG2	chemokine receptors	REACTOME_SEMA3A_PAK_DEPENDENT_AXON_REPULSION	
NUDC	cancer therapy resistance	REACTOME_ESTROGEN_DEPENDENT_GENE_EXPRESSION	
ODC1	enhanced anti-tumor immune response	WP_PARKINUBIQUITIN_PROTEASOMAL_SYSTEM_PATHWAY	
IRF1	ovarian carcinoma	REACTOME_AUTOPHAGY	
PMAIP1	antigen-presenting functions	WP_PROLACTIN_SIGNALING_PATHWAY	
ANXA1	malignant evolution	REACTOME_SELECTIVE_AUTOPHAGY	
GADD45B	immunological self-tolerance	WP_EBSTEINBARR_VIRUS_LMP1_SIGNALING	
HBP1	heightened cytokine production	WP_APOPTOSIS	
FABP5	evasion strategies	WP_UNFOLDED_PROTEIN_RESPONSE	
CCL20	antigen degradation	HALLMARK_IL6_JAK_STAT3_SIGNALING	
NEU1	viral gene expression	REACTOME_POTENTIAL_THERAPEUTICS_FOR_SARS	
GEM	tissue regeneration	REACTOME_MAPK6_MAPK4_SIGNALING	
	aggrephagy-related immune cell subtypes	WP_TNFALPHA_SIGNALING_PATHWAY	
	periodontal disease	REACTOME_UPTAKE_AND_ACTIONS_OF_BACTERIAL_TOXINS	
	metastatic renal cell carcinoma	HALLMARK_HYPOXIA	
	de novo immune responses	HALLMARK_P53_PATHWAY	
	monocyte-macrophage diversity		
	ovarian clear cell carcinoma		
	antiapoptotic genes		
	intratumoral inflammation		
	proinflammatory immune responses		
	heterosubtypic protection		
	perihilar large duct type		
	zeste homolog 2 methyltransferase		
	long-term tumor clearance		
	high antitumor efficacy		
	anti-tumor immune phenotypes		

Cytotoxic	ICKG_annotation (ranked by pagerank score)	ORA_annotation	LLM_annotation
GZMB	vaccine-mediated antibody response	KEGG_GRAFT_VERSUS_HOST_DISEASE	Cytotoxic T-cell Mediated Immunity (0.95)
GZMH	tumor burden	WP_ALLOGRAFT_REJECTION	
PRF1	effector cells	KEGG_ALLOGRAFT_REJECTION	
CCL4	fulminant inflammatory bowel disease	HALLMARK_ALLOGRAFT_REJECTION	
GNLY	pancreatic cancer growth	KEGG_TYPE_I_DIABETES_MELLITUS	
GZMA	complete tumor eradication	WP_INTERACTIONS_OF_NATURAL_KILLER_CELLS_IN_PANCREATIC_CANCER	
ALOX5AP	sustained tumor control	KEGG_AUTOIMMUNE_THYROID_DISEASE	
CCL4L2	subsequent effector differentiation	KEGG_VIRAL_MYOCARDITIS	
KLRD1	leukemia-free survival	KEGG_LEISHMANIA_INFECTION	
CD63	cross-present melanoma-derived tumor antigens	KEGG_ANTIGEN_PROCESSING_AND_PRESENTATION	
HOPX	anti-tumor efficacy	REACTOME_PD_1_SIGNALING	
NKG7	important immune functions	KEGG_ASTHMA	
CST7	decreased antigen recognition ability	WP_SARSCOV2_INNATE_IMMUNITY_EVASION_AND_CELLSPECIFIC_IMMUNE_RESPONSE	
GZMK	exhaustion markers	REACTOME_MHC_CLASS_II_ANTIGEN_PRESENTATION	
CCL3	cellular bioenergetics	KEGG_NATURAL_KILLER_CELL_MEDIATED_CYTOTOXICITY	
APOBEC3G	cell death	WP_NETWORK_MAP_OF_SARSCOV2_SIGNALING_PATHWAY	
IFNG	cytotoxic antitumor effects	REACTOME_GENERATION_OF_SECOND_MESSENGER_MOLECULES	
CXCR6	insulin resistance	REACTOME_INTERFERON_GAMMA_SIGNALING	
HLA-DRA	hepatocyte proliferation	KEGG_INTESTINAL_IMMUNE_NETWORK_FOR_IGA_PRODUCTION	
CMC1	cytokine/chemokine release	KEGG_CYTOKINE_CYTOKINE_RECEPTOR_INTERACTION	
HLA-DRB1	anti-tumour immune responses	REACTOME_CHEMOKINE_RECEPTORS_BIND_CHEMOKINES	
TIGIT	proper organ function	WP_TYROBP_CAUSAL_NETWORK_IN_MICROGLIA	
CXCL13	co-culture systems	WP_OVERVIEW_OF_PROINFLAMMATORY_AND_PROFIBROTIC_MEDIATORS	
LAG3	epithelial regeneration	WP_EBOLA_VIRUS_INFECTION_IN_HOST	
CTSW	anti-inflammation effect	KEGG_CELL_ADHESION_MOLECULES_CAMS	
LGALS1	coinhibitory receptors	WP_CYTOKINES_AND_INFLAMMATORY_RESPONSE	
RGS1	keratinocyte proliferation	KEGG_SYSTEMIC_LUPUS_ERYTHEMATOSUS	
HLA-DPA1	early tertiary lymphoid structure	REACTOME_COSTIMULATION_BY_THE_CD28_FAMILY	
KLRB1	specific cancer-related gene sets	WP_SELECTIVE_EXPRESSION_OF_CHEMOKINE_RECEPTORS_DURING_TCELL_POLARIZATION	
CD27	greater tumor infiltration	WP_PROSTAGLANDIN_SIGNALING	
ITGB2	intra-pancreatic inoculation	KEGG_CHEMOKINE_SIGNALING_PATHWAY	
DUSP2	focal cell death	REACTOME_IMMUNOREGULATORY_INTERACTIONS_BETWEEN_A_LYMPHOID_AND_A_NON_LYMPHOID_CELL	
CTSD	intratumor microbiota	HALLMARK_COMPLEMENT	
DUSP4	acidic substance-related gene sets	REACTOME_INTERFERON_SIGNALING	
HAVCR2	cell cycle arrest	REACTOME_NOTCH2_INTRACELLULAR_DOMAIN_REGULATES_TRANSCRIPTION	
SAMSN1	ischemia reperfusion injury-related phenotypes	REACTOME_TCR_SIGNALING	
ITM2C	immune pathologies	WP_MIRNAS_INVOLVEMENT_IN_THE_IMMUNE_RESPONSE_IN_SEPSIS	
TRGC2	relapsed/refractory multiple myeloma	REACTOME_METABOLISM_OF_ANGIOTENSINOGEN_TO_ANGIOTENSINS	
ZNF683	allotransplant rejection	WP_CHEMOKINE_SIGNALING_PATHWAY	
HLA-DPB1	effector function inhibition	WP_PATHOGENESIS_OF_SARSCOV2_MEDIATED_BY_NSP9NSP10_COMPLEX	
CAPG	progressive disease	REACTOME_RAF_INDEPENDENT_MAPK1_3_ACTIVATION	
KLRG1	protective immune memory	WP_CANCER_IMMUNOTHERAPY_BY_PD1_BLOCKADE	
RBPJ	immune checkpoint inhibitor	KEGG_HEMATOPOIETIC_CELL_LINEAGE	
AC092580.4	fat-associated lymphoid structures	WP_TCELL_ACTIVATION_SARSCOV2	
ITM2A	immune system balance	REACTOME_G_ALPHA_I_SIGNALLING_EVENTS	
CD7	complete tumour regression	REACTOME_SIGNALING_BY_INTERLEUKINS	
CKLF	cancer cell infection	REACTOME_PEPTIDE_LIGAND_BINDING_RECEPTORS	
KLRC1	durable tumor immunity	HALLMARK_TNFA_SIGNALING_VIA_NFKB	
RGS2	natural killer cell activity	WP_DEVELOPMENT_AND_HETEROGENEITY_OF_THE_ILC_FAMILY	
ID2	established signal transduction pathways	REACTOME_SIGNALING_BY_NOTCH2	
	anti-leukemia immunosurveillance	KEGG_LYSOSOME	
	cardiac xenotransplantation	REACTOME_NEGATIVE_REGULATION_OF_MAPK_PATHWAY	
	immune-edited tumors	REACTOME_INTERLEUKIN_10_SIGNALING	
	relapsed/refractory hematological malignancies		
	robust transduction		
	autoimmune hepatitis		
	lineage-specific gene programs		
	portal inflammation		
	chronic activation/exhaustion		
	immune-mediated clearance		
	progression-free interval		
	disease-specific survival		
	appropriate immune responses		
	immune cell recruitment		
	parietal cell loss		
	advanced cutaneous melanoma		
	treatment-associated tumor necrosis		
	targeted single-gene perturbation		
	genetic circuits		
	responsive hot tumors		
	combination immunotherapy		
	local tissue protection		
	endogenous adaptive antitumour immunity		
	transplantable mouse tumor models		
	checkpoint-based immunotherapy		
	reduced terminal exhaustion		
	increased memory cell characteristics		
	post-ablation immune therapy		
	perihilar large duct type		
	dense tumor infiltration		
	response heterogeneity		
	dysregulated immune reactions		
	trained immunity induction		
	proinflammatory polarization		
	divergent cellular response		
	leukemia-like disease		
	post allogeneic hematopoietic stem cell transplantation		
	cancer immunoprevention		
	monocyte differentiation		
	organ-adapted protection		
	lymph node homing capability		
	intrahepatic cholangiocarcinoma		
	leukemia cell survival		
	tumor-specific-stereotyped sequences		

Cell_cycle	ICKG_annotation (ranked by pagerank score)	ORA_annotation	LLM_annotation
STMN1	cell cycle	HALLMARK_E2F_TARGETS	Cell Cycle Regulation and Mitotic Division (0.95)
TUBB	anti-tumor immunity	HALLMARK_G2M_CHECKPOINT	
TUBA1B	anti-tumor function	WP_RETINOBLASTOMA_GENE_IN_CANCER	
HMGB2	tumor-associated antigens	REACTOME_MITOTIC_PROMETAPHASE	
CKS1B	synthetic peptide-based anticancer vaccines	REACTOME_M_PHASE	
NUSAP1	tumor mutational burden	REACTOME_MITOTIC_METAPHASE_AND_ANAPHASE	
PCNA	cancer immune regulation	REACTOME_MITOTIC_G1_PHASE_AND_G1_S_TRANSITION	
TYMS	gene rearrangement	REACTOME_RESOLUTION_OF_SISTER_CHROMATID_COHESION	
DUT	plasma cell neoplasia	REACTOME_SEPARATION_OF_SISTER_CHROMATIDS	
UBE2C	tumor keratinocytes	REACTOME_CELL_CYCLE_CHECKPOINTS	
HMGN2	tumor vascular abnormalities	REACTOME_RHO_GTPASES_ACTIVATE_FORMINS	
SMC4	improved antitumor activity	REACTOME_G1_S_SPECIFIC_TRANSCRIPTION	
TOP2A	myeloma-related disorder	REACTOME_MITOTIC_SPINDLE_CHECKPOINT	
HIST1H4C	cancer relapse	HALLMARK_MITOTIC_SPINDLE	
CDK1	cancer-related mortality	HALLMARK_MYC_TARGETS_V1	
MKI67	melanoma models	REACTOME_APC_C_MEDIATED_DEGRADATION_OF_CELL_CYCLE_PROTEINS	
CENPF	anticancer immunity	REACTOME_G0_AND_EARLY_G1	
KIAA0101	disease outcome	WP_GASTRIC_CANCER_NETWORK_1	
BIRC5	striking tumor regression	KEGG_CELL_CYCLE	
CKS2	breast cancer clinical treatment selection	REACTOME_MITOTIC_G2_G2_M_PHASES	
PTTG1	protein folding	REACTOME_CONDENSATION_OF_PROMETAPHASE_CHROMOSOMES	
ZWINT	cancer vaccines	REACTOME_RHO_GTPASE_EFFECTORS	
MAD2L1	adaptive immune response	REACTOME_S_PHASE	
RANBP1	local tumor control	REACTOME_METABOLISM_OF_NUCLEOTIDES	
TK1	antitumor efficacy	REACTOME_SUMOYLATION_OF_DNA_REPLICATION_PROTEINS	
MCM7	anti-tumorigenic innate immune cells	REACTOME_SYNTHESIS_OF_DNA	
CENPM	anti-tumor innate immunity	WP_CELL_CYCLE	
RRM2	long-term antitumor memory	WP_G1_TO_S_CELL_CYCLE_CONTROL	
SMC2	cancer therapy resistance	REACTOME_AURKA_ACTIVATION_BY_TPX2	
DNAJC9	advanced melanoma	REACTOME_NUCLEAR_ENVELOPE_NE_REASSEMBLY	
TMPO	certain hematological malignancies	REACTOME_INTERCONVERSION_OF_NUCLEOTIDE_DI_AND_TRIPHOSPHATES	
RPA3	leukemia eradication	HALLMARK_DNA_REPAIR	
TUBB4B	elicit adaptive immune responses	REACTOME_TRANSCRIPTIONAL_REGULATION_BY_TP53	
CDKN3	cancer-colonized organs	REACTOME_DNA_STRAND_ELONGATION	
H2AFZ	immune synapse formation	REACTOME_TERMINATION_OF_TRANSLESION_DNA_SYNTHESIS	
UBE2T	respiratory disease	REACTOME_REGULATION_OF_TP53_ACTIVITY	
AURKB	anti-acetylcholine receptor antibody production	WP_GASTRIC_CANCER_NETWORK_2	
TMEM106C	cell type specific gene-regulatory programs	WP_PYRIMIDINE_METABOLISM	
ASPM	peripheral blood immune system	WP_FLUOROPYRIMIDINE_ACTIVITY	
NUDT1	precision cancer immunotherapies	KEGG_GAP_JUNCTION	
KIF22	dual-lymphoid activation	WP_COHESIN_COMPLEX_CORNELIA_DE_LANGE_SYNDROME	
H2AFV	signaling	REACTOME_REGULATION_OF_TP53_ACTIVITY_THROUGH_PHOSPHORYLATION	
DEK	co-stimulatory domains	KEGG_DNA_REPLICATION	
SIVA1	advanced melanoma patients	REACTOME_RECRUITMENT_OF_NUMA_TO_MITOTIC_CENTROSOMES	
PCLAF	poor progression-free survival	REACTOME_TRANSLESION_SYNTHESIS_BY_Y_FAMILY_DNA_POLYMERASES_BYPASSES_LESIONS_ON_DNA_TEMPLATE	
FABP5	tumor-infiltrating lymphocyte therapy	KEGG_PYRIMIDINE_METABOLISM	
CCNA2	long-term responses	REACTOME_DNA_REPLICATION	
DDX39A	terminal differentiation	WP_DNA_REPLICATION	
TPX2	melanoma metastases	REACTOME_DNA_DAMAGE_BYPASS	
CARHSP1	putative cancer immune evasion	REACTOME_TP53_REGULATES_TRANSCRIPTION_OF_CELL_CYCLE_GENES	
	marrow-residence genes	WP_MICROTUBULE_CYTOSKELETON_REGULATION	
	adjuvant dendritic cell vaccination	REACTOME_EXTENSION_OF_TELOMERES	
	substantial global tumor regression	WP_PATHOGENIC_ESCHERICHIA_COLI_INFECTION	
	anti-tumor adaptive immune responses	KEGG_PATHOGENIC_ESCHERICHIA_COLI_INFECTION	
	efficient tumor-immune responses	REACTOME_CHROMOSOME_MAINTENANCE	
	plasma cell differentiation	REACTOME_KINESINS	
	antigen-specific cellular immunity	REACTOME_MITOTIC_PROPHASE	
	melanoma tumour burden	REACTOME_DEPOLYMERISATION_OF_THE_NUCLEAR_LAMINA	
	melanoma development	REACTOME_MISMATCH_REPAIR	
	cancer-immune status	REACTOME_PROCESSIVE_SYNTHESIS_ON_THE_LAGGING_STRAND	
	significantly reduced tumor growth	WP_BIOMARKERS_FOR_PYRIMIDINE_METABOLISM_DISORDERS	
	severe cutaneous adverse reaction	REACTOME_TRANSCRIPTION_OF_E2F_TARGETS_UNDER_NEGATIVE_CONTROL_BY_P107_RBL1_AND_P130_RBL2_IN_COMPLEX_WITH_HDAC1	
	widespread skin lesions	WP_REGULATION_OF_SISTER_CHROMATID_SEPARATION_AT_THE_METAPHASEANAPHASE_TRANSITION	
	virus-derived antigenic peptides	REACTOME_TRANSLESION_SYNTHESIS_BY_POLK	
	tumor-promoting crosstalk	WP_PYRIMIDINE_METABOLISM_AND_RELATED_DISEASES	
	intrinsic dysregulation	WP_PARKINUBIQUITIN_PROTEASOMAL_SYSTEM_PATHWAY	
	hypersensitivity reaction	REACTOME_TP53_REGULATES_TRANSCRIPTION_OF_GENES_INVOLVED_IN_G2_CELL_CYCLE_ARREST	
	immune-related gene signatures	REACTOME_CONDENSATION_OF_PROPHASE_CHROMOSOMES	
	mild cytokine release syndrome	REACTOME_APC_C_CDH1_MEDIATED_DEGRADATION_OF_CDC20_AND_OTHER_APC_C_CDH1_TARGETED_PROTEINS_IN_LATE_MITOSIS_EARLY_G1	
	lymph node homing capability	REACTOME_INITIATION_OF_NUCLEAR_ENVELOPE_NE_REFORMATION	
	myeloid cell clearance	REACTOME_PROCESSIVE_SYNTHESIS_ON_THE_C_STRAND_OF_THE_TELOMERE	
	antigen-presenting neutrophil delivery	REACTOME_TRANSCRIPTION_OF_E2F_TARGETS_UNDER_NEGATIVE_CONTROL_BY_DREAM_COMPLEX	
	costimulation-dependent dysfunctional programs	REACTOME_TRANSLESION_SYNTHESIS_BY_POLH	
	dysfunctional cells	REACTOME_LAGGING_STRAND_SYNTHESIS	
	co-mutated lung adenocarcinoma	REACTOME_PHOSPHORYLATION_OF_THE_APC_C	
	human leukocyte antigen background	REACTOME_PROTEIN_UBIQUITINATION	
	decreased antigen recognition ability	REACTOME_THE_ROLE_OF_GTSE1_IN_G2_M_PROGRESSION_AFTER_G2_CHECKPOINT	
	diffuse glioma	REACTOME_INHIBITION_OF_THE_PROTEOLYTIC_ACTIVITY_OF_APC_C_REQUIRED_FOR_THE_ONSET_OF_ANAPHASE_BY_MITOTIC_SPINDLE_CHECKPOINT_COMPONENTS	
	adoptive cellular transfer	REACTOME_PCNA_DEPENDENT_LONG_PATCH_BASE_EXCISION_REPAIR	
	mammary tumorigenesis	REACTOME_TRANSPORT_OF_CONNEXONS_TO_THE_PLASMA_MEMBRANE	
	potent complement-dependent cytotoxic effects	REACTOME_RECRUITMENT_OF_MITOTIC_CENTROSOME_PROTEINS_AND_COMPLEXES	
	bispecific antibody-mediated antitumour activity	KEGG_MISMATCH_REPAIR	
	interferon regulatory factor 1 transcriptional activat	REACTOME_POST_CHAPERONIN_TUBULIN_FOLDING_PATHWAY	
	heparan sulfate backbone synthesis	WP_DNA_MISMATCH_REPAIR	
	unfolded proteins	KEGG_PROGESTERONE_MEDIATED_OOCYTE_MATURATION	
	microenvironmental immunosuppression	REACTOME_REGULATION_OF_PLK1_ACTIVITY_AT_G2_M_TRANSITION	
	immune system development	REACTOME_APC_C_CDC20_MEDIATED_DEGRADATION_OF_CYCLIN_B	
	skin cutaneous melanoma	REACTOME_SUMOYLATION	
	local expansion	REACTOME_CYCLIN_A_B1_B2_ASSOCIATED_EVENTS_DURING_G2_M_TRANSITION	
	immune-stromal interaction mechanisms	REACTOME_GAP_FILLING_DNA_REPAIR_SYNTHESIS_AND_LIGATION_IN_GG_NER	
	coagulation cascade signals	REACTOME_RESOLUTION_OF_AP_SITES_VIA_THE_MULTIPLE_NUCLEOTIDE_PATCH_REPLACEMENT_PATHWAY	
		REACTOME_SWITCHING_OF_ORIGINS_TO_A_POST_REPLICATIVE_STATE	
		REACTOME_APC_CDC20_MEDIATED_DEGRADATION_OF_NEK2A	
		REACTOME_FORMATION_OF_TUBULIN_FOLDING_INTERMEDIATES_BY_CCT_TRIC	
		REACTOME_ANCHORING_OF_THE_BASAL_BODY_TO_THE_PLASMA_MEMBRANE	
		REACTOME_DNA_REPAIR	
		REACTOME_CILIUM_ASSEMBLY	
		REACTOME_ACTIVATION_OF_AMPK_DOWNSTREAM_OF_NMDARS	
		REACTOME_COPI_DEPENDENT_GOLGI_TO_ER_RETROGRADE_TRAFFIC	
		REACTOME_RECOGNITION_OF_DNA_DAMAGE_BY_PCNA_CONTAINING_REPLICATION_COMPLEX	
		REACTOME_SYNTHESIS_OF_ACTIVE_UBIQUITIN_ROLES_OF_E1_AND_E2_ENZYMES	
		REACTOME_RHO_GTPASES_ACTIVATE_IQGAPS	
		REACTOME_SEALING_OF_THE_NUCLEAR_ENVELOPE_NE_BY_ESCRT_III	
		REACTOME_ACTIVATION_OF_THE_PRE_REPLICATIVE_COMPLEX	
		REACTOME_COOPERATION_OF_PREFOLDIN_AND_TRIC_CCT_IN_ACTIN_AND_TUBULIN_FOLDING	
		REACTOME_TELOMERE_MAINTENANCE	
		REACTOME_TELOMERE_C_STRAND_LAGGING_STRAND_SYNTHESIS	
		KEGG_OOCYTE_MEIOSIS	
		REACTOME_ACTIVATION_OF_ATR_IN_RESPONSE_TO_REPLICATION_STRESS	
		REACTOME_REGULATION_OF_TP53_EXPRESSION_AND_DEGRADATION	
		REACTOME_GAP_JUNCTION_ASSEMBLY	
		REACTOME_RESOLUTION_OF_ABASIC_SITES_AP_SITES	
		REACTOME_FANCONI_ANEMIA_PATHWAY	
		REACTOME_MHC_CLASS_II_ANTIGEN_PRESENTATION	
		REACTOME_DUAL_INCISION_IN_GG_NER	
		KEGG_NUCLEOTIDE_EXCISION_REPAIR	
		REACTOME_AGGREPHAGY	
		REACTOME_ASSEMBLY_AND_CELL_SURFACE_PRESENTATION_OF_NMDA_RECEPTORS	
		WP_NUCLEOTIDE_EXCISION_REPAIR	
		REACTOME_GOLGI_TO_ER_RETROGRADE_TRANSPORT	
		REACTOME_CARBOXYTERMINAL_POST_TRANSLATIONAL_MODIFICATIONS_OF_TUBULIN	
		WP_ATM_SIGNALING_IN_DEVELOPMENT_AND_DISEASE	
		REACTOME_HOMOLOGY_DIRECTED_REPAIR	
		REACTOME_RECYCLING_PATHWAY_OF_L1	
		WP_HEPATITIS_C_AND_HEPATOCELLULAR_CARCINOMA	
		REACTOME_GAP_JUNCTION_TRAFFICKING_AND_REGULATION	
		REACTOME_COPI_INDEPENDENT_GOLGI_TO_ER_RETROGRADE_TRAFFIC	
		REACTOME_NUCLEAR_ENVELOPE_BREAKDOWN	
		REACTOME_INTRAFLAGELLAR_TRANSPORT	
		REACTOME_ORGANELLE_BIOGENESIS_AND_MAINTENANCE	
		REACTOME_HSP90_CHAPERONE_CYCLE_FOR_STEROID_HORMONE_RECEPTORS_SHR_IN_THE_PRESENCE_OF_LIGAND	
		REACTOME_SCF_SKP2_MEDIATED_DEGRADATION_OF_P27_P21	
		REACTOME_DNA_DOUBLE_STRAND_BREAK_REPAIR	
		REACTOME_G2_M_CHECKPOINTS	
		REACTOME_FACTORS_INVOLVED_IN_MEGAKARYOCYTE_DEVELOPMENT_AND_PLATELET_PRODUCTION	
		REACTOME_DUAL_INCISION_IN_TC_NER	
		REACTOME_HDR_THROUGH_HOMOLOGOUS_RECOMBINATION_HRR	
		KEGG_P53_SIGNALING_PATHWAY	
		WP_RAC1PAK1P38MMP2_PATHWAY	
		REACTOME_ORC1_REMOVAL_FROM_CHROMATIN	

Memory_Naive1	ICKG_annotation (ranked by pagerank score)	ORA_annotation	LLM_annotation
FOS	poorer prognosis	HALLMARK_TNFA_SIGNALING_VIA_NFKB	Immune Response and Cellular Stress Regulation (0.85)
IL7R	immune evasion	HALLMARK_INFLAMMATORY_RESPONSE	
DUSP1	gastric cancer peritoneal metastasis	HALLMARK_APOPTOSIS	
GPR183	effector cells	HALLMARK_HYPOXIA	
LTB	poor gastric cancer	HALLMARK_UV_RESPONSE_UP	
JUNB	interstitial lung disease	WP_GLUCOCORTICOID_RECEPTOR_PATHWAY	
BTG2	ovarian pathogenesis	WP_NUCLEAR_RECEPTORS_METAPATHWAY	
FOSB	antitumor functions	WP_IL18_SIGNALING_PATHWAY	
NFKBIA	tumor environments	WP_TGFBETA_SIGNALING_PATHWAY	
DNAJB1	skin inflammation	WP_PDGF_PATHWAY	
YPEL5	disease outcome	WP_NEUROINFLAMMATION	
ZNF331	tumor formation processes	WP_CORTICOTROPINRELEASING_HORMONE_SIGNALING_PATHWAY	
GZMK	cell adhesion	WP_QUERCETIN_AND_NFKB_AP1_INDUCED_APOPTOSIS	
CD69	irreversible cirrhosis	WP_IL3_SIGNALING_PATHWAY	
ANXA1	cancer immune surveillance	WP_RANKLRANK_SIGNALING_PATHWAY	
RGCC	cell survival	WP_GASTRIN_SIGNALING_PATHWAY	
PPP1R15A	functional exhaustion	REACTOME_HSP90_CHAPERONE_CYCLE_FOR_STEROID_HORMONE_RECEPTORS_SHR_IN_THE_PRESENCE_OF_LIGAND	
LMNA	pro-immunogenic expression changes	WP_SPINAL_CORD_INJURY	
DUSP2	immunogenic gene expression	HALLMARK_P53_PATHWAY	
NR4A2	tumor immune status	WP_ONCOSTATIN_M_SIGNALING_PATHWAY	
SLC2A3	extracellular matrix proteins	WP_MEASLES_VIRUS_INFECTION	
TNFAIP3	immune checkpoint dysfunction	KEGG_B_CELL_RECEPTOR_SIGNALING_PATHWAY	
CCR7	complex karyotype acute mixed cell leukemia	WP_PROLACTIN_SIGNALING_PATHWAY	
CD55	relapsing/progressive leukemia	WP_PDGFRBETA_PATHWAY	
TXNIP	heightened tumor cell metabolism	WP_OREXIN_RECEPTOR_PATHWAY	
MYADM	dysregulated perfusion	WP_HOSTPATHOGEN_INTERACTION_OF_HUMAN_CORONAVIRUSES_INTERFERON_INDUCTION	
ZFP36	subsequent immune infiltration	WP_APOPTOSIS	
JUN	advanced gastric cancer	WP_TCELL_ACTIVATION_SARSCOV2	
BIRC3	castration resistance	WP_HOSTPATHOGEN_INTERACTION_OF_HUMAN_CORONAVIRUSES_MAPK_SIGNALING	
DNAJA1	metabolic dysfunction-associated steatohepa	WP_APOPTOSIS_MODULATION_AND_SIGNALING	
GADD45B	sialic acid-binding immunoglobulin-like lectins	WP_TCELL_RECEPTOR_TCR_SIGNALING_PATHWAY	
IER2	lactic acid accumulation	WP_TNFALPHA_SIGNALING_PATHWAY	
SNHG8	peritoneal dissemination	REACTOME_NGF_STIMULATED_TRANSCRIPTION	
AREG	gastric cancer development	REACTOME_MYD88_INDEPENDENT_TLR4_CASCADE	
SELL	intestinal-type gastric carcinoma	WP_SMALL_CELL_LUNG_CANCER	
EIF3E	cellular tropism	WP_IL5_SIGNALING_PATHWAY	
LEPROTL1	clinical remission	KEGG_TOLL_LIKE_RECEPTOR_SIGNALING_PATHWAY	
TUBA4A	epigenetic alterations	WP_IL2_SIGNALING_PATHWAY	
CREM	increased fibrosis	WP_TNFRELATED_WEAK_INDUCER_OF_APOPTOSIS_TWEAK_SIGNALING_PATHWAY	
TMEM123	integrins	WP_TOLLLIKE_RECEPTOR_SIGNALING_PATHWAY	
FAM177A1	lactic acid secretion	KEGG_T_CELL_RECEPTOR_SIGNALING_PATHWAY	
KLF6	sustained protein translation	REACTOME_ACTIVATION_OF_THE_AP_1_FAMILY_OF_TRANSCRIPTION_FACTORS	
PIK3IP1	memory-like responses	REACTOME_INTERLEUKIN_4_AND_INTERLEUKIN_13_SIGNALING	
PIK3R1	cytomegalovirus viremia	HALLMARK_IL2_STAT5_SIGNALING	
TOB1	heightened cytokine production	HALLMARK_ESTROGEN_RESPONSE_LATE	
TSC22D3	receptor function	HALLMARK_KRAS_SIGNALING_UP	
RGS2	viral gene expression	HALLMARK_MTORC1_SIGNALING	
TUBB4B	type 2 immune response	WP_VEGFAVEGFR2_SIGNALING_PATHWAY	
RGS1	type 2 immune responses	WP_PHOTODYNAMIC_THERAPYINDUCED_AP1_SURVIVAL_SIGNALING	
CXCR4	drug-resistant tumors	REACTOME_MAPK_FAMILY_SIGNALING_CASCADES	
	virus-neutralizing antibodies	REACTOME_ESR_MEDIATED_SIGNALING	
	dysregulated tumor glycolysis	REACTOME_SIGNALING_BY_INTERLEUKINS	
	sialic acid incorporation	WP_IL4_SIGNALING_PATHWAY	
	insufficient infiltration	REACTOME_NUCLEOTIDE_BINDING_DOMAIN_LEUCINE_RICH_REPEAT_CONTAINING_RECEPTOR_NLR_SIGNALING_PATHWAYS	
	robust immune responses	WP_IL1_SIGNALING_PATHWAY	
	immunocytes' activation	REACTOME_SIGNALING_BY_NTRKS	
	established signal transduction pathways	WP_KIT_RECEPTOR_SIGNALING_PATHWAY	
	chronic obstructive pulmonary disease	REACTOME_NUCLEAR_EVENTS_KINASE_AND_TRANSCRIPTION_FACTOR_ACTIVATION	
	cystic fibrosis	WP_MODULATORS_OF_TCR_SIGNALING_AND_T_CELL_ACTIVATION	
	engulf cancer cells	KEGG_COLORECTAL_CANCER	
	cell cycle progression	KEGG_NOD_LIKE_RECEPTOR_SIGNALING_PATHWAY	
	lymphocyte differentiation	WP_HEMATOPOIETIC_STEM_CELL_DIFFERENTIATION	
	treatment-associated tumor necrosis	WP_TCELL_ANTIGEN_RECEPTOR_TCR_PATHWAY_DURING_STAPHYLOCOCCUS_AUREUS_INFECTION	
	immunoglobulin deficiency	WP_ENDOMETRIAL_CANCER	
	anti-tumor dysfunction	WP_MAPK_PATHWAY_IN_CONGENITAL_THYROID_CANCER	
	zeste homolog 2 methyltransferase	WP_BRAINDERIVED_NEUROTROPHIC_FACTOR_BDNF_SIGNALING_PATHWAY	
	higher basal respiration	REACTOME_GAB1_SIGNALOSOME	
		REACTOME_TNF_RECEPTOR_SUPERFAMILY_TNFSF_MEMBERS_MEDIATING_NON_CANONICAL_NF_KB_PATHWAY	
		WP_MELANOMA	
		WP_THYROID_STIMULATING_HORMONE_TSH_SIGNALING_PATHWAY	
		REACTOME_ER_TO_GOLGI_ANTEROGRADE_TRANSPORT	
		REACTOME_TOLL_LIKE_RECEPTOR_CASCADES	
		KEGG_LEISHMANIA_INFECTION	
		WP_BREAST_CANCER_PATHWAY	
		WP_MYOMETRIAL_RELAXATION_AND_CONTRACTION_PATHWAYS	
		KEGG_MAPK_SIGNALING_PATHWAY	
		WP_CHROMOSOMAL_AND_MICROSATELLITE_INSTABILITY_IN_COLORECTAL_CANCER	
		REACTOME_EXTRA_NUCLEAR_ESTROGEN_SIGNALING	
		REACTOME_NUCLEAR_ENVELOPE_NE_REASSEMBLY	
		WP_EGFEGFR_SIGNALING_PATHWAY	
		WP_CHEMOKINE_SIGNALING_PATHWAY	
		REACTOME_TP53_REGULATES_TRANSCRIPTION_OF_ADDITIONAL_CELL_CYCLE_GENES_WHOSE_EXACT_ROLE_IN_THE_P53_PATHWAY_REMAIN_UNCERTAIN	
		REACTOME_TRANSPORT_OF_CONNEXONS_TO_THE_PLASMA_MEMBRANE	
		WP_VITAMIN_D_IN_INFLAMMATORY_DISEASES	
		REACTOME_POST_CHAPERONIN_TUBULIN_FOLDING_PATHWAY	
		REACTOME_RAF_INDEPENDENT_MAPK1_3_ACTIVATION	
		WP_ESTROGEN_SIGNALING_PATHWAY	
		WP_PHOTODYNAMIC_THERAPYINDUCED_NFE2L2_NRF2_SURVIVAL_SIGNALING	
		KEGG_SMALL_CELL_LUNG_CANCER	
		REACTOME_ESTROGEN_DEPENDENT_NUCLEAR_EVENTS_DOWNSTREAM_OF_ESR_MEMBRANE_SIGNALING	
		REACTOME_SIGNALING_BY_NUCLEAR_RECEPTORS	
		KEGG_APOPTOSIS	
		KEGG_ERBB_SIGNALING_PATHWAY	
		WP_ACUTE_VIRAL_MYOCARDITIS	
		REACTOME_DEFECTIVE_INTRINSIC_PATHWAY_FOR_APOPTOSIS	
		REACTOME_SIGNALING_BY_EGFR_IN_CANCER	
		WP_IL7_SIGNALING_PATHWAY	
		WP_PHYSIOLOGICAL_AND_PATHOLOGICAL_HYPERTROPHY_OF_THE_HEART	
		WP_UNFOLDED_PROTEIN_RESPONSE	
		WP_HAIR_FOLLICLE_DEVELOPMENT_CYTODIFFERENTIATION_PART_3_OF_3	
		REACTOME_FORMATION_OF_TUBULIN_FOLDING_INTERMEDIATES_BY_CCT_TRIC	
		REACTOME_FC_EPSILON_RECEPTOR_FCERI_SIGNALING	
		REACTOME_TRANSPORT_TO_THE_GOLGI_AND_SUBSEQUENT_MODIFICATION	
		WP_ERBB_SIGNALING_PATHWAY	
		KEGG_CHEMOKINE_SIGNALING_PATHWAY	
		REACTOME_TOLL_LIKE_RECEPTOR_9_TLR9_CASCADE	
		REACTOME_G_ALPHA_I_SIGNALLING_EVENTS	
		REACTOME_ACTIVATION_OF_AMPK_DOWNSTREAM_OF_NMDARS	
		WP_SELECTIVE_EXPRESSION_OF_CHEMOKINE_RECEPTORS_DURING_TCELL_POLARIZATION	
		WP_B_CELL_RECEPTOR_SIGNALING_PATHWAY	
		HALLMARK_EPITHELIAL_MESENCHYMAL_TRANSITION	
		HALLMARK_INTERFERON_GAMMA_RESPONSE	
		KEGG_PATHWAYS_IN_CANCER	
		REACTOME_TNFR1_INDUCED_NFKAPPAB_SIGNALING_PATHWAY	
		REACTOME_COPI_MEDIATED_ANTEROGRADE_TRANSPORT	
		REACTOME_MAPK_TARGETS_NUCLEAR_EVENTS_MEDIATED_BY_MAP_KINASES	
		REACTOME_TOLL_LIKE_RECEPTOR_TLR1_TLR2_CASCADE	
		REACTOME_RHO_GTPASES_ACTIVATE_IQGAPS	
		REACTOME_SEALING_OF_THE_NUCLEAR_ENVELOPE_NE_BY_ESCRT_III	
		REACTOME_COOPERATION_OF_PREFOLDIN_AND_TRIC_CCT_IN_ACTIN_AND_TUBULIN_FOLDING	
		WP_HEPATOCYTE_GROWTH_FACTOR_RECEPTOR_SIGNALING	
		WP_OXIDATIVE_STRESS_RESPONSE	
		REACTOME_G_ALPHA_Q_SIGNALLING_EVENTS	
		WP_BURN_WOUND_HEALING	
		REACTOME_REGULATION_OF_TNFR1_SIGNALING	
		WP_SIGNAL_TRANSDUCTION_THROUGH_IL1R	
		WP_NETWORK_MAP_OF_SARSCOV2_SIGNALING_PATHWAY	
		REACTOME_INTERLEUKIN_7_SIGNALING	
		REACTOME_NOD1_2_SIGNALING_PATHWAY	
		REACTOME_GAP_JUNCTION_ASSEMBLY	
		WP_ATM_SIGNALING_PATHWAY	
		WP_MBDNF_AND_PROBDNF_REGULATION_OF_GABA_NEUROTRANSMISSION	
		WP_NEURAL_CREST_CELL_MIGRATION_DURING_DEVELOPMENT	
		KEGG_NEUROTROPHIN_SIGNALING_PATHWAY	
		REACTOME_NEGATIVE_REGULATION_OF_MAPK_PATHWAY	
		WP_IL6_SIGNALING_PATHWAY	
		WP_MAPK_SIGNALING_PATHWAY	
		REACTOME_AGGREPHAGY	
		REACTOME_ASSEMBLY_AND_CELL_SURFACE_PRESENTATION_OF_NMDA_RECEPTORS	
		REACTOME_TNF_SIGNALING	
		WP_FAS_LIGAND_PATHWAY_AND_STRESS_INDUCTION_OF_HEAT_SHOCK_PROTEINS	
		WP_NEURAL_CREST_CELL_MIGRATION_IN_CANCER	
		WP_ADIPOGENESIS	
		REACTOME_CARBOXYTERMINAL_POST_TRANSLATIONAL_MODIFICATIONS_OF_TUBULIN	
		WP_THYMIC_STROMAL_LYMPHOPOIETIN_TSLP_SIGNALING_PATHWAY	
		WP_ARYL_HYDROCARBON_RECEPTOR_PATHWAY_WP2873	
		REACTOME_DEATH_RECEPTOR_SIGNALLING	
		REACTOME_RECYCLING_PATHWAY_OF_L1	
		REACTOME_TP53_REGULATES_TRANSCRIPTION_OF_CELL_CYCLE_GENES	
		WP_EXERCISEINDUCED_CIRCADIAN_REGULATION	
		KEGG_CYTOKINE_CYTOKINE_RECEPTOR_INTERACTION	
		REACTOME_SIGNALING_BY_EGFR	
		WP_HEPATITIS_C_AND_HEPATOCELLULAR_CARCINOMA	
		WP_INTERLEUKIN1_IL1_STRUCTURAL_PATHWAY	
		REACTOME_GAP_JUNCTION_TRAFFICKING_AND_REGULATION	

Interferon	ICKG_annotation (ranked by pagerank score)	ORA_annotation	LLM_annotation
MX1	immunogenic cell death	HALLMARK_INTERFERON_GAMMA_RESPONSE	Antiviral Defense and Interferon Response (0.95)
ISG15	myeloid cells	HALLMARK_INTERFERON_ALPHA_RESPONSE	
IFI6	fulminant inflammatory bowel disease	REACTOME_INTERFERON_SIGNALING	
OASL	cell-mediated immunity	REACTOME_INTERFERON_ALPHA_BETA_SIGNALING	
IFI44L	insulin sensitivity	REACTOME_ANTIVIRAL_MECHANISM_BY_IFN_STIMULATED_GENES	
RSAD2	adaptive immune response	WP_TYPE_I_INTERFERON_INDUCTION_AND_SIGNALING_DURING_SARSCOV2_INFECTION	
IFIT3	tumour cell death	WP_HOSTPATHOGEN_INTERACTION_OF_HUMAN_CORONAVIRUSES_INTERFERON_INDUCTION	
OAS1	programmed cell death	REACTOME_INTERFERON_GAMMA_SIGNALING	
STAT1	established immune responses	WP_TYPE_II_INTERFERON_SIGNALING_IFNG	
PLSCR1	antitumor response	WP_IMMUNE_RESPONSE_TO_TUBERCULOSIS	
SAMD9L	clonal expansion	WP_MEASLES_VIRUS_INFECTION	
TNFSF10	precision immunotherapy	WP_NETWORK_MAP_OF_SARSCOV2_SIGNALING_PATHWAY	
IFI35	tumor antigens	REACTOME_NEGATIVE_REGULATORS_OF_DDX58_IFIH1_SIGNALING	
IFIT1	immune modulation	REACTOME_DDX58_IFIH1_MEDIATED_INDUCTION_OF_INTERFERON_ALPHA_BETA	
GBP1	immune-related toxicity	WP_SARSCOV2_INNATE_IMMUNITY_EVASION_AND_CELLSPECIFIC_IMMUNE_RESPONSE	
HERC5	antitumor efficacy	WP_NONGENOMIC_ACTIONS_OF_125_DIHYDROXYVITAMIN_D3	
ISG20	immune-mediated anti-tumor immunity	REACTOME_TRAF3_DEPENDENT_IRF_ACTIVATION_PATHWAY	
USP18	cross presentation	WP_PATHWAYS_OF_NUCLEIC_ACID_METABOLISM_AND_INNATE_IMMUNE_SENSING	
IFI44	abscopal anticancer activity	WP_NOVEL_INTRACELLULAR_COMPONENTS_OF_RIGILIKE_RECEPTOR_RLR_PATHWAY	
IFIT2	long-term cell-mediated anti-tumor immunity	HALLMARK_INFLAMMATORY_RESPONSE	
IRF7	chronic inflammation	WP_EBOLA_VIRUS_INFECTION_IN_HOST	
NT5C3A	cellular immune activation	KEGG_RIG_I_LIKE_RECEPTOR_SIGNALING_PATHWAY	
SAMD9	potent complement-dependent cytotoxic effects	WP_CYTOSOLIC_DNASENSING_PATHWAY	
TRIM22	bispecific antibody-mediated antitumour activity	REACTOME_TRAF6_MEDIATED_IRF7_ACTIVATION	
UBE2L6	interferon regulatory factor 1 transcriptional activat	REACTOME_NICOTINATE_METABOLISM	
EPSTI1	immuno-metabolic regulation	WP_SARS_CORONAVIRUS_AND_INNATE_IMMUNITY	
MX2	ankylosing spondylitis	WP_MITOCHONDRIAL_IMMUNE_RESPONSE_TO_SARSCOV2	
BST2	cancer therapy resistance	REACTOME_MATURATION_OF_SARS_COV_1_NUCLEOPROTEIN	
EIF2AK2	cell cycle regulatory genes	REACTOME_PYRIMIDINE_CATABOLISM	
OAS3	effector function capabilities	REACTOME_NF_KB_ACTIVATION_THROUGH_FADD_RIP_1_PATHWAY_MEDIATED_BY_CASPASE_8_AND_10	
XAF1	immune-mediated acute liver injury	KEGG_CYTOSOLIC_DNA_SENSING_PATHWAY	
CMPK2	cancer immunology	REACTOME_MATURATION_OF_SARS_COV_2_NUCLEOPROTEIN	
MT2A	antibody-based immunotherapy	WP_BIOMARKERS_FOR_PYRIMIDINE_METABOLISM_DISORDERS	
PPM1K	antibody-based constructs	REACTOME_CYTOSOLIC_SENSORS_OF_PATHOGEN_ASSOCIATED_DNA	
PARP14	interstitial lung disease	WP_PYRIMIDINE_METABOLISM_AND_RELATED_DISEASES	
OAS2	chronic activation/exhaustion	REACTOME_NICOTINAMIDE_SALVAGING	
IFI16	immune-mediated clearance	WP_HEPATITIS_B_INFECTION	
LGALS9	vivo cytotoxicity	HALLMARK_APOPTOSIS	
NMI	cancer-targeted photoimmunotherapy	KEGG_NICOTINATE_AND_NICOTINAMIDE_METABOLISM	
PARP9	massive tumor cell death	REACTOME_TRAF6_MEDIATED_NF_KB_ACTIVATION	
CD38	basal cells	REACTOME_REGULATION_OF_IFNA_SIGNALING	
HELZ2	immune synapse formation	REACTOME_TRANSLATION_OF_SARS_COV_1_STRUCTURAL_PROTEINS	
LAP3	tumour-elicited immune responses	WP_PDGFRBETA_PATHWAY	
LY6E	human lung epithelial development	KEGG_PYRIMIDINE_METABOLISM	
SP110	transient cytokine release	REACTOME_TERMINATION_OF_TRANSLESION_DNA_SYNTHESIS	
IFIH1	myeloid cell infiltration	HALLMARK_TNFA_SIGNALING_VIA_NFKB	
SAT1	post-treatment relapse	REACTOME_NUCLEOTIDE_CATABOLISM	
TYMP	relapsed/refractory acute myeloid leukemia	REACTOME_OVARIAN_TUMOR_DOMAIN_PROTEASES	
DDX58	cancer evolution	REACTOME_TRANSLESION_SYNTHESIS_BY_Y_FAMILY_DNA_POLYMERASES_BYPASSES_LESIONS_ON_DNA_TEMPLATE	
ZBP1	tumor evolution factors		
	mechanism-based cancer immunotherapy		
	strong antitumor efficacy		
	hematopoietic stem cell transplantation		
	signaling		
	immune status		
	genetic associations		
	leukemic-cell resistance		
	tumor heterogeneity		
	hyperprogressive disease		
	superior tumor reduction		
	cancer diagnosis		
	relapsed acute myeloid leukemia		
	transplant-related morbidity		
	terminal differentiation		
	tumor size		
	inhibitory immune cells		
	suppress antitumor immune responses		
	cellular immunotherapeutics		
	cancer biology		
	left-sided malignant epithelia		
	effector function inhibition		
	damaging immune responses		
	rapid antitumor effects		
	plasma cell neoplasia		
	heightened cytokine production		
	durable immune memory		
	immune system balance		
	gene rearrangement		
	reduced pathological inflammation		
	immune escape mechanism		
	metabolic regulation		
	cancer microvessels		
	peripheral immune tolerance		
	multiple immune checkpoints		
	tumor keratinocytes		
	stromal cell support		
	mycosis fungoides		
	costimulation-dependent dysfunctional programs		
	dysfunctional cells		
	extracellular vesicles		
	facilitated clonal expansion		
	central immune tolerance		
	1911 cases		
	294 cases		
	antitumor phenotypes		
	kidney transplantation		
	negative feedback regulation		
	adaptive immune function		
	renal allograft dysfunction		
	anti-tumor therapeutics		
	potent antitumor cytotoxicity		
	precancerous lesions		
	peripheral blood immune system		
	thymus function		
	pylori-induced gastritis		
	immune system development		
	perihilar large duct type		
	leukemia cell survival		
	local expansion		
	cancer immune escape		
	anti-tumour efficacy		
	cell type specific gene-regulatory programs		
	elicit epigenetic dysfunction		
	immunoglobulin deficiency		
	potent antitumor activity		
	increased antitumor immunity		

Unassigned11	CKG_annotation (ranked by pagerank score)	ORA_annotation	LLM_annotation
GIMAP7	poorer prognosis	REACTOME_IMMUNOREGULATORY_INTERACTIONS_BETWEEN_A_LYMPHOID_AND_A_NON_LYMP	Immune Response and Cellular Metabolism Regulation (0.85)
GIMAP4	cancer progression	REACTOME_GENERATION_OF_SECOND_MESSENGER_MOLECULES	
GZMK	tumor burden	WP_TYROBP_CAUSAL_NETWORK_IN_MICROGLIA	
ATRX	gastric cancer peritoneal metastasis	KEGG_VIRAL_MYOCARDITIS	
TXNIP	effector cells		
TRAF3IP3	poor gastric cancer		
ARGLU1	pancreatic cancer growth		
KIAA1551	leukemia-free survival		
KLRG1	cross-present melanoma-derived tumor antigens		
MPHOSPH8	anti-tumor efficacy		
BTN3A2	important immune functions		
CMC1	interstitial lung disease		
PNISR	cancer cell growth		
TBC1D10C	cellular bioenergetics		
DDX17	insulin resistance		
IKZF3	cell migration		
N4BP2L2	insulin sensitivity		
PPP1R18	gastric cancer pathogenesis		
SAMD3	tumor escape		
AKAP9	anti-tumour immune responses		
CAP1	cancer heterogeneity		
CD27	cytokine/chemokine release		
FYB	gastric cancer immunotherapy		
GIMAP1	intra-pancreatic inoculation		
IL7R	focal cell death		
LCK	intratumor microbiota		
NKTR	anti-tumour immunity		
PTP4A2	cell cycle arrest		
STK17A	fat-associated lymphoid structures		
TRGC2	impaired antigen presentation		
APMAP	cellular immune responses		
EVL	complete tumour regression		
GCC2	immune-based cancer therapies		
ITGB2	advanced gastric cancer		
LIMD2	cancer risk		
MBNL1	monocyte activation		
PIK3IP1	autoimmune reconstitution		
PRPF38B	peritoneal dissemination		
RNF213	gastric cancer development		
SH2D1A	intestinal-type gastric carcinoma		
SYNE2	defective negative selection		
HLA-F	receptor function		
KLRB1	marrow-residence genes		
PLEK	cell proliferation-inhibiting bystander effects		
UCP2	memory-like responses		
PRPF4B	cancer immunotherapy resistance		
POLR2J3	lineage-specific gene programs		
GPSM3	cytomegalovirus viremia		
HLA-DRB1	transplantable mouse tumor models		
MBP	previously known tumor antigens		
	mild cytokine release syndrome		
	potent antitumor cytotoxicity		
	checkpoint-based immunotherapy		
	peripheral immune tolerance		
	multiple immune checkpoints		
	cancer microvessels		
	co-mutated lung adenocarcinoma		
	obesity-induced chronic inflammation		
	obesity-induced immune dysregulation		
	obesity-induced immune responses		
	mammary tumorigenesis		
	high antitumor efficacy		
	long-term tumor clearance		
	immunoglobulin deficiency		

Naive2	ICKG_annotation (ranked by pagerank score)	ORA_annotation	LLM_annotation
SELL	poorer prognosis	REACTOME_REPRESSION_OF_WNT_TARGET_GENES	T-cell Activation and Differentiation (0.85)
TMEM123	tumor regression	WP_NSP1_FROM_SARSCOV2_INHIBITS_TRANSLATION_INITIATION_IN_THE_HOST_CELL	
CCR7	gastric cancer peritoneal metastasis	REACTOME_ACTIVATION_OF_THE_MRNA_UPON_BINDING_OF_THE_CAP_BINDING_COMPLEX_AND_EIFS_AND_SUBSEQUENT_BINDING_TO_43S	
LTB	poor gastric cancer	REACTOME_CA2_PATHWAY	
NOSIP	cell-associated viral transcripts	KEGG_THYROID_CANCER	
EIF3L	cancer immune regulation	WP_WNT_SIGNALING_PATHWAY_AND_PLURIPOTENCY	
IL7R	human immunodeficiency virus	HALLMARK_WNT_BETA_CATENIN_SIGNALING	
RSL1D1	master regulator genes	HALLMARK_INFLAMMATORY_RESPONSE	
TCF7	anti-tumor efficacy	WP_WNT_SIGNALING	
KLF2	protein expression	WP_SPINAL_CORD_INJURY	
LDHB	antitumor properties	REACTOME_EUKARYOTIC_TRANSLATION_INITIATION	
LEF1	improved antitumor activity	KEGG_ENDOMETRIAL_CANCER	
PIK3IP1	cancer risk	WP_TRANSLATION_FACTORS	
MAL	anti-tumour immune responses	WP_WNT_SIGNALING_PATHWAY	
SATB1	skin inflammation	KEGG_ACUTE_MYELOID_LEUKEMIA	
SNHG8	tumor formation processes	KEGG_COLORECTAL_CANCER	
CD55	cell adhesion	REACTOME_BETA_CATENIN_INDEPENDENT_WNT_SIGNALING	
FAM65B	antigen-presenting neutrophil delivery	WP_ENDOMETRIAL_CANCER	
PLAC8	hyperinducible expression	KEGG_WNT_SIGNALING_PATHWAY	
C6ORF48	effector function capabilities	WP_AMPLIFICATION_AND_EXPANSION_OF_ONCOGENIC_PATHWAYS_AS_METASTATIC_TRAITS	
FOXP1	cell cycle regulatory genes	WP_CHROMOSOMAL_AND_MICROSATELLITE_INSTABILITY_IN_COLORECTAL_CANCER	
LDLRAP1	anti-tumor innate immunity	REACTOME_TRANSLATION	
PRKCQ-AS1	long-term antitumor memory	REACTOME_DEGRADATION_OF_BETA_CATENIN_BY_THE_DESTRUCTION_COMPLEX	
TXK	anti-tumorigenic innate immune cells	REACTOME_FORMATION_OF_THE_BETA_CATENIN_TCF_TRANSACTIVATING_COMPLEX	
AQP3	vivo oncogenic function	WP_NCRNAS_INVOLVED_IN_WNT_SIGNALING_IN_HEPATOCELLULAR_CARCINOMA	
CD27	altered phosphorylation ratios	WP_IL7_SIGNALING_PATHWAY	
EIF2S3	anti-tumour immunity	HALLMARK_MYC_TARGETS_V1	
EIF3E	glioma cell progression	WP_WNTBETACATENIN_SIGNALING_PATHWAY_IN_LEUKEMIA	
FAIM3	intratumoral macrophages	REACTOME_TRANSCRIPTIONAL_REGULATION_BY_RUNX3	
NDFIP1	heightened tumor cell metabolism	WP_COMPLEMENT_SYSTEM	
PPA1	dysregulated perfusion	WP_HIPPO_SIGNALING_REGULATION_PATHWAYS	
ALKBH7	protective immune memory	KEGG_MELANOGENESIS	
EIF3D	complete tumour regression	WP_LNCRNA_IN_CANONICAL_WNT_SIGNALING_AND_COLORECTAL_CANCER	
GIMAP1	cervical cancer malignancy	WP_GASTRIC_CANCER_NETWORK_2	
MYC	anti-tumoral immunity	WP_HAIR_FOLLICLE_DEVELOPMENT_ORGANOGENESIS_PART_2_OF_3	
NUCB2	advanced gastric cancer	WP_DNA_DAMAGE_RESPONSE_ONLY_ATM_DEPENDENT	
PHB2	glioma cell population		
PRKCA	immune-based cancer therapies		
PRMT2	optimal primary expansion		
RASGRP2	antitumor treatment		
RCAN3	sensory ablation		
SNHG7	antibody dynamics		
SORL1	effective antitumor immune responses		
ST13	protective antitumor immune responses		
UXT	denervation-dependent antitumor effects		
TXNIP	peritoneal dissemination		
AIF1	gastric cancer development		
ERP29	intestinal-type gastric carcinoma		
TRAT1	anticancer immunosurveillance		
ATP5A1	lactic acid accumulation		
	mediate anticancer immunity		
	disease biology		
	systemic immune checkpoint inhibitors		
	Œ±vŒ≤3 integrins		
	lymphocyte fate determination		
	marrow-residence genes		
	higher mutation load		
	sustained protein translation		
	lactic acid secretion		
	receptor function		
	autoimmune hepatitis		
	drug-resistant tumors		
	type 2 immune response		
	type 2 immune responses		
	lymphoid cell fate		
	virus-neutralizing antibodies		
	dysregulated tumor glycolysis		
	tumor-promoting macrophage responses		
	impaired anti-tumor activity		
	sialic acid incorporation		
	insufficient infiltration		
	lineage-specific gene programs		
	vaccine-mediated antibody response		
	colorectal carcinoma		
	lasting protective immune responses		
	robust immune responses		
	transplantable mouse tumor models		
	previously known tumor antigens		
	potent antitumor cytotoxicity		
	glioblastoma immunotherapy		
	local tissue protection		
	skin rashes		
	type 2/type 17 immune responses		
	immune landscape		
	retarded tumor progression		
	acute inflammation		
	cell cycle progression		
	lymphocyte differentiation		
	relapsed/refractory hematologic malignancies		
	post allogeneic hematopoietic stem cell transplantation		
	cancer immunoprevention		
	monocyte differentiation		
	organ-adapted protection		
	lymph node homing capability		
	human immune homeostasis		
	immunomodulation process		
	intact tumor-cell antigen presentation		
	sustained antitumor activity		
	short-term immunologic response		
	long-term clinical outcomes		
	autoimmune disease treatment		
	higher antitumor activity		
	enhanced immune cell recruitment		
	breast cancer stem cell-associated signaling pathways		

Glycolysis_MYC	ICKG_annotation (ranked by pagerank score)	ORA_annotation	LLM_annotation
FABP5	anti-tumor immunity	HALLMARK_MYC_TARGETS_V1	Protein Synthesis and Cellular Energy Metabolism (0.85)
NME1	tumoricidal factors	REACTOME_FOLDING_OF_ACTIN_BY_CCT_TRIC	
C1QBP	anti-tumor function	REACTOME_METABOLISM_OF_NUCLEOTIDES	
RANBP1	pro-inflammatory cytokines	KEGG_NOD_LIKE_RECEPTOR_SIGNALING_PATHWAY	
EIF5A	initiated liver cirrhosis	REACTOME_RHOBTB2_GTPASE_CYCLE	
NHP2	antigen presentation pathways	HALLMARK_MTORC1_SIGNALING	
PKM	striking tumor regression	REACTOME_FORMATION_OF_TUBULIN_FOLDING_INTERMEDIATES_BY_CCT_TRIC	
TNFRSF9	early tertiary lymphoid structure	REACTOME_INTERCONVERSION_OF_NUCLEOTIDE_DI_AND_TRIPHOSPHATES	
CXCL13	significantly increased recognition	REACTOME_COOPERATION_OF_PREFOLDIN_AND_TRIC_CCT_IN_ACTIN_AND_TUBULIN_FOLDING	
SNRPE	durable anti-tumor immune responses	REACTOME_RHOBTB_GTPASE_CYCLE	
ATP5G1	local tumor control	WP_ANDROGEN_RECEPTOR_SIGNALING_PATHWAY	
CCT2	malignant tumor immunity	REACTOME_COOPERATION_OF_PDCL_PHLP1_AND_TRIC_CCT_IN_G_PROTEIN_BETA_FOLDING	
ENO1	optimal primary expansion	REACTOME_ASSOCIATION_OF_TRIC_CCT_WITH_TARGET_PROTEINS_DURING_BIOSYNTHESIS	
PGAM1	ischemia reperfusion injury-related phenotypes	WP_METABOLIC_REPROGRAMMING_IN_COLON_CANCER	
PRDX1	immune-regulation-related signaling pathways	WP_GLYCOLYSIS_AND_GLUCONEOGENESIS	
PRMT1	extracellular matrix	REACTOME_ATF6_ATF6_ALPHA_ACTIVATES_CHAPERONE_GENES	
TNFRSF18	effector cytokine secretion	WP_GLYCOLYSIS_IN_SENESCENCE	
XCL2	longer progression-free survival	HALLMARK_UNFOLDED_PROTEIN_RESPONSE	
CRTAM	low-risk subgroups	HALLMARK_E2F_TARGETS	
PPA1	immune status	REACTOME_ATF6_ATF6_ALPHA_ACTIVATES_CHAPERONES	
RAN	ectopic accumulation	WP_AEROBIC_GLYCOLYSIS	
VDAC1	cytotoxic antitumor effects	HALLMARK_MYC_TARGETS_V2	
XCL1	durable tumor immunity	REACTOME_CHEMOKINE_RECEPTORS_BIND_CHEMOKINES	
CCT5	natural killer cell activity	KEGG_GLYCOLYSIS_GLUCONEOGENESIS	
CCT6A	critical immune factors	REACTOME_NEUTROPHIL_DEGRANULATION	
HSP90B1	systemic immunotoxicity	REACTOME_SCAVENGING_BY_CLASS_A_RECEPTORS	
NDUFAB1	allotransplant rejection	REACTOME_GLYCOLYSIS	
NFKBIA	tertiary lymphoid structure-like lymphocytic aggreg	REACTOME_TOLL_LIKE_RECEPTOR_CASCADES	
PARK7	cumulative death induction	KEGG_CYTOKINE_CYTOKINE_RECEPTOR_INTERACTION	
BIRC3	cross-present melanoma-derived tumor antigens	WP_16P112_PROXIMAL_DELETION_SYNDROME	
DCTPP1	cardiac xenotransplantation	REACTOME_CHAPERONE_MEDIATED_AUTOPHAGY	
DNPH1	established signal transduction pathways	REACTOME_BBSOME_MEDIATED_CARGO_TARGETING_TO_CILIUM	
EIF3I	skin rashes	REACTOME_MITOCHONDRIAL_CALCIUM_ION_TRANSPORT	
FBL	type 2/type 17 immune responses	WP_PYRIMIDINE_METABOLISM	
GTF3C6	robust transduction	REACTOME_DEFECTIVE_INTRINSIC_PATHWAY_FOR_APOPTOSIS	
HSP90AB1	lasting tumor regression	KEGG_PROSTATE_CANCER	
ILF2	complete eradication	REACTOME_GLUCOSE_METABOLISM	
PHB	tumor cell lysis	KEGG_CHEMOKINE_SIGNALING_PATHWAY	
SEC11C	mycosis fungoides	REACTOME_TNFS_BIND_THEIR_PHYSIOLOGICAL_RECEPTORS	
TIMM13	important immune functions	WP_PHOTODYNAMIC_THERAPYINDUCED_UNFOLDED_PROTEIN_RESPONSE	
TXNDC17	local bystander activation	REACTOME_MYD88_INDEPENDENT_TLR4_CASCADE	
CALR	innate-like protection	REACTOME_PROTEIN_FOLDING	
DUT	innate-like memory	HALLMARK_ALLOGRAFT_REJECTION	
TNIP2	sustained tumor control	HALLMARK_TNFA_SIGNALING_VIA_NFKB	
BATF	significantly reduced tumor growth	REACTOME_TNFR2_NON_CANONICAL_NF_KB_PATHWAY	
LSM5	treatment-associated tumor necrosis		
BANF1	epithelial barrier function		
IMPDH2	epithelial cell expulsion		
NOP16	memory cell differentiation		
SNRPD1	neojunction-derived antigens		
	antigen-specific immune responses		
	recurrent clonal expansion		
	improved eradication		
	immunomodulation process		
	intact tumor-cell antigen presentation		
	dramatic disease regression		
	better tumor control		
	lineage-specific gene programs		
	antigen-driven differentiation		
	distant nonirradiated tumors		
	short-term immunologic response		
	long-term clinical outcomes		
	autoimmune disease treatment		
	higher antitumor activity		
	autoimmune-mediated neuroinflammation		
	relapse prevention		
	event-free survival		
	significant toxicity		
	retrovirus immunity		
	lymphocyte activatory receptors		
	elicit epigenetic dysfunction		

Chromatin	ICKG_annotation (ranked by pagerank score)	ORA_annotation	LLM_annotation
NEAT1	cell adhesion	REACTOME_EPHA_MEDIATED_GROWTH_CONE_COLLAPSE	Chromatin remodeling and cytoskeletal organization (0.85)
SYNE2	specific cancer-related gene sets	REACTOME_DISEASES_OF_SIGNAL_TRANSDUCTION_BY_GROWTH_FACTOR_RECEPTORS_AND_SECOND_MESSENGERS	
MACF1	co-culture systems	REACTOME_SIGNALING_BY_ALK_IN_CANCER	
AHNAK	acidic substance-related gene sets	HALLMARK_UV_RESPONSE_DN	
GOLGB1	immune modulation	REACTOME_SEMAPHORIN_INTERACTIONS	
KMT2A	heightened reactive oxygen species production		
AKAP9	lipid peroxidation byproducts		
ANKRD11	disease outcome		
NKTR	tumor immunosuppression		
SMG1	cancer therapy resistance		
SYNE1	tumoricidal capacity		
ATRX	anti-tumor efficacy		
DDX17	effector cytokine secretion		
GPRIN3	cancer cell infection		
SMCHD1	immunocytes' activation		
UTRN	immunoglobulin deficiency		
ZNF292	respiratory disease		
AKNA	precision cancer immunotherapies		
ARGLU1	terminal differentiation		
CHD2	dual-lymphoid activation		
ITGA4	rapid antitumor effects		
PCSK7	tumor size		
PHF3	intratumoral neurodegeneration		
ROCK1	marrow-residence genes		
RORA	left-sided malignant epithelia		
ATF7IP	substantial global tumor regression		
ATM	cancer immunotherapy resistance		
BPTF	portal inflammation		
CBLB	castration resistance		
DDX6	severe cutaneous adverse reaction		
DYNC1H1	widespread skin lesions		
FYN	efficient immune synapse formation		
LINC-PINT	rational combination treatments		
MT-ND6	advanced cutaneous melanoma		
MYH9	costimulation-dependent dysfunctional programs		
NUFIP2	dysfunctional cells		
OGA	immune-mediated clearance		
PDE3B	virus-derived antigenic peptides		
RESF1	hypersensitivity reaction		
RP11-138A9.1	parietal cell loss		
SETX	clinical remission		
XIST	subsequent immune infiltration		
SLFN5	anti-acetylcholine receptor antibody production		
EML4	mild cytokine release syndrome		
GCC2	tumor-specific immunity		
IKZF3	response heterogeneity		
BDP1	dysregulated immune reactions		
RASGRP1	trained immunity induction		
GOLGA4	proinflammatory polarization		
CEP350	divergent cellular response		
	co-mutated lung adenocarcinoma		
	higher tumor grades		
	shorter recurrence-free survival		
	human leukocyte antigen background		
	decreased antigen recognition ability		
	long-term immunologic memory		
	diffuse glioma		
	mammary tumorigenesis		
	immune system development		
	increased osteoblast mineralization		
	heparan sulfate backbone synthesis		
	viral gene expression		
	receptor internalization		
	local expansion		
	thymus function		
	conditionally replicative adenoviruses		
	zeste homolog 2 methyltransferase		

Unassigned2	CKG_annotation (ranked by pagerank score	ORA_annotation	LLM_annotation
COTL1	cell cycle	KEGG_REGULATION_OF_ACTIN_CYTOSKELETON	Cytoskeleton organization and cellular metabolism (0.85)
PPP1CA	peripheral neuropathy	WP_PATHOGENIC_ESCHERICHIA_COLI_INFECTION	
PSME2	tumoricidal factors	KEGG_PATHOGENIC_ESCHERICHIA_COLI_INFECTION	
ARPC1B	cytotoxic extracellular vesicles	REACTOME_RHO_GTPASES_ACTIVATE_WASPS_AND_WAVES	
FKBP1A	systemic toxicity	WP_AEROBIC_GLYCOLYSIS	
LGALS1	aggressive solid tumors	REACTOME_EPHB_MEDIATED_FORWARD_SIGNALING	
CAP1	complete blood cancer regression	REACTOME_EPH_EPHRIN_SIGNALING	
ENO1	clonal expansion	REACTOME_PCP_CE_PATHWAY	
MT2A	antigen presentation pathways	WP_GLYCOLYSIS_AND_GLUCONEOGENESIS	
TPI1	cytotoxic antitumor effects	KEGG_GLYCOLYSIS_GLUCONEOGENESIS	
TUBB	cancer relapse	HALLMARK_GLYCOLYSIS	
PARK7	cancer-related mortality	REACTOME_HOST_INTERACTIONS_OF_HIV_FACTORS	
S100A4	chronic inflammation	REACTOME_GLYCOLYSIS	
APOBEC3G	protein folding	REACTOME_BETA_CATENIN_INDEPENDENT_WNT_SIGNALING	
ARPC5	malignant tumor immunity	REACTOME_DOWNSTREAM_SIGNALING_EVENTS_OF_B_CELL_RECEPTOR_BCR	
CAPZB	cellular immune responses	REACTOME_GLUCONEOGENESIS	
CORO1A	immune-regulation-related signaling pathw	REACTOME_NEUTROPHIL_DEGRANULATION	
EMP3	exhaustion markers	REACTOME_GLUCOSE_METABOLISM	
PKM	longer progression-free survival	KEGG_FC_GAMMA_R_MEDIATED_PHAGOCYTOSIS	
PPP1R18	low-risk subgroups	REACTOME_UCH_PROTEINASES	
ANXA5	cancer cell death	WP_METABOLIC_REPROGRAMMING_IN_COLON_CANCER	
ARPC4	cell proliferation-inhibiting bystander effect	KEGG_PROTEASOME	
ATP5G3	melanoma metastases	HALLMARK_COMPLEMENT	
CALM3	cancer-colonized organs	HALLMARK_INTERFERON_GAMMA_RESPONSE	
CLIC1	peripheral blood immune system	WP_GLYCOLYSIS_IN_SENESCENCE	
GZMA	systemic immunotoxicity	REACTOME_CROSS_PRESENTATION_OF_SOLUBLE_EXOGENOUS_ANTIGENS_ENDOSOMES	
GZMH	critical immune factors	REACTOME_PARASITE_INFECTION	
LSP1	tumor antigen heterogeneity	REACTOME_RHO_GTPASE_EFFECTORS	
PGAM1	allotransplant rejection	REACTOME_NEGATIVE_REGULATION_OF_NOTCH4_SIGNALING	
PSME1	established signal transduction pathways	REACTOME_AUF1_HNRNP_D0_BINDS_AND_DESTABILIZES_MRNA	
S100A11	tumor cell lysis	REACTOME_DEGRADATION_OF_AXIN	
STMN1	damaging immune responses	REACTOME_REGULATION_OF_RUNX3_EXPRESSION_AND_ACTIVITY	
TXN	translation attenuation	REACTOME_SIGNALING_BY_ROBO_RECEPTORS	
ACTB	responsive hot tumors	REACTOME_DEGRADATION_OF_DVL	
BLOC1S1	previously known tumor antigens	REACTOME_STABILIZATION_OF_P53	
C12ORF75	hypoxia-associated immunosuppression	REACTOME_CDT1_ASSOCIATION_WITH_THE_CDC6_ORC_ORIGIN_COMPLEX	
CAPG	immunosuppressive tumor microenvironm	REACTOME_METABOLISM_OF_POLYAMINES	
CKLF	diminished immunogenicity	REACTOME_DEGRADATION_OF_GLI1_BY_THE_PROTEASOME	
IL32	potent antitumor cytotoxicity	REACTOME_SCF_SKP2_MEDIATED_DEGRADATION_OF_P27_P21	
MYL12A	gene therapy medicines	REACTOME_DEFECTIVE_CFTR_CAUSES_CYSTIC_FIBROSIS	
PSMA7	treatment-associated tumor necrosis	REACTOME_HIV_INFECTION	
RAC2	perihilar large duct type	REACTOME_DECTIN_1_MEDIATED_NONCANONICAL_NF_KB_SIGNALING	
SLC25A5	lymph node homing capability	REACTOME_ASYMMETRIC_LOCALIZATION_OF_PCP_PROTEINS	
SNRPE	immune-related gene signatures	WP_PROTEASOME_DEGRADATION	
TBC1D10C	intrahepatic cholangiocarcinoma	REACTOME_HEDGEHOG_LIGAND_BIOGENESIS	
CD52	precancerous lesions	REACTOME_REGULATION_OF_HMOX1_EXPRESSION_AND_ACTIVITY	
SH3BGRL3	tissue residency	REACTOME_FCGAMMA_RECEPTOR_FCGR_DEPENDENT_PHAGOCYTOSIS	
ITM2C	myocardial necrosis	WP_CORI_CYCLE	
PFN1	pylori-induced gastritis	REACTOME_G1_S_DNA_DAMAGE_CHECKPOINTS	
LY6E	unfolded proteins	REACTOME_REGULATION_OF_RAS_BY_GAPS	
	tumor-specific-stereotyped sequences	REACTOME_REGULATION_OF_PTEN_STABILITY_AND_ACTIVITY	
	long-term tumor remission	WP_REGULATION_OF_ACTIN_CYTOSKELETON	
	oncogenic viruses	REACTOME_ORC1_REMOVAL_FROM_CHROMATIN	
	coagulation cascade signals	REACTOME_REGULATION_OF_RUNX2_EXPRESSION_AND_ACTIVITY	
		REACTOME_APC_C_CDH1_MEDIATED_DEGRADATION_OF_CDC20_AND_OTHER_APC_C_CDH1_TARGETED_PROTEINS_IN_LATE_MITOSIS_EARLY_G1	
		REACTOME_CELLULAR_RESPONSE_TO_HYPOXIA	
		REACTOME_CELLULAR_RESPONSE_TO_CHEMICAL_STRESS	
		REACTOME_ABC_TRANSPORTER_DISORDERS	
		REACTOME_THE_ROLE_OF_GTSE1_IN_G2_M_PROGRESSION_AFTER_G2_CHECKPOINT	
		REACTOME_SIGNALING_BY_THE_B_CELL_RECEPTOR_BCR	
		REACTOME_RESPONSE_OF_MTB_TO_PHAGOCYTOSIS	
		REACTOME_SIGNALING_BY_NOTCH4	
		REACTOME_CYCLIN_A_CDK2_ASSOCIATED_EVENTS_AT_S_PHASE_ENTRY	
		REACTOME_DEGRADATION_OF_BETA_CATENIN_BY_THE_DESTRUCTION_COMPLEX	
		REACTOME_HEDGEHOG_ON_STATE	
		REACTOME_REGULATION_OF_MRNA_STABILITY_BY_PROTEINS_THAT_BIND_AU_RICH_ELEMENTS	
		WP_CLEAR_CELL_RENAL_CELL_CARCINOMA_PATHWAYS	
		REACTOME_APC_C_MEDIATED_DEGRADATION_OF_CELL_CYCLE_PROTEINS	
		REACTOME_DISEASES_OF_SIGNAL_TRANSDUCTION_BY_GROWTH_FACTOR_RECEPTORS_AND_SECOND_MESSENGERS	
		REACTOME_SWITCHING_OF_ORIGINS_TO_A_POST_REPLICATIVE_STATE	
		REACTOME_INFECTION_WITH_MYCOBACTERIUM_TUBERCULOSIS	
		WP_VEGFAVEGFR2_SIGNALING_PATHWAY	
		REACTOME_MAPK6_MAPK4_SIGNALING	
		REACTOME_LEISHMANIA_INFECTION	
		REACTOME_TRANSCRIPTIONAL_REGULATION_BY_RUNX3	
		HALLMARK_INTERFERON_ALPHA_RESPONSE	
		KEGG_FOCAL_ADHESION	
		REACTOME_CLEC7A_DECTIN_1_SIGNALING	
		HALLMARK_APICAL_JUNCTION	
		HALLMARK_EPITHELIAL_MESENCHYMAL_TRANSITION	
		HALLMARK_HYPOXIA	
		REACTOME_MITOTIC_G2_G2_M_PHASES	
		REACTOME_TNFR2_NON_CANONICAL_NF_KB_PATHWAY	
		WP_FOCAL_ADHESION	
		REACTOME_INTERLEUKIN_1_SIGNALING	
		REACTOME_ABC_FAMILY_PROTEINS_MEDIATED_TRANSPORT	
		REACTOME_TGF_BETA_RECEPTOR_SIGNALING_ACTIVATES_SMADS	
		REACTOME_SIGNALING_BY_WNT	
		REACTOME_ANTIGEN_PROCESSING_CROSS_PRESENTATION	
		REACTOME_HEDGEHOG_OFF_STATE	
		KEGG_LEUKOCYTE_TRANSENDOTHELIAL_MIGRATION	
		REACTOME_SYNTHESIS_OF_DNA	
		REACTOME_TRANSCRIPTIONAL_REGULATION_BY_RUNX2	
		WP_MICROGLIA_PATHOGEN_PHAGOCYTOSIS_PATHWAY	
		REACTOME_CYTOPROTECTION_BY_HMOX1	
		REACTOME_TCR_SIGNALING	
		REACTOME_RHOF_GTPASE_CYCLE	
		WP_COMMON_PATHWAYS_UNDERLYING_DRUG_ADDICTION	
		REACTOME_RUNX1_REGULATES_TRANSCRIPTION_OF_GENES_INVOLVED_IN_DIFFERENTIATION_OF_HSCS	
		REACTOME_RESPONSE_TO_ELEVATED_PLATELET_CYTOSOLIC_CA2	
		REACTOME_FCERI_MEDIATED_NF_KB_ACTIVATION	
		REACTOME_PTEN_REGULATION	
		HALLMARK_REACTIVE_OXYGEN_SPECIES_PATHWAY	
		REACTOME_C_TYPE_LECTIN_RECEPTORS_CLRS	
		REACTOME_INTERLEUKIN_1_FAMILY_SIGNALING	
		REACTOME_PLATELET_ACTIVATION_SIGNALING_AND_AGGREGATION	

Heat_shock	ICKG_annotation (ranked by pagerank score)	ORA_annotation	LLM_annotation
HSPA1B	poorer prognosis	REACTOME_ATTENUATION_PHASE	Cellular Stress Response and Protein Folding (0.92)
HSPH1	gastric cancer peritoneal metastasis	REACTOME_HSF1_ACTIVATION	
HSPA1A	immune modulation	REACTOME_HSF1_DEPENDENT_TRANSACTIVATION	
HSPA6	poor gastric cancer	REACTOME_CELLULAR_RESPONSE_TO_HEAT_STRESS	
HSPB1	multiple myeloma	REACTOME_REGULATION_OF_HSF1_MEDIATED_HEAT_SHOCK_RESPONSE	
ZFAND2A	immune system dynamics	REACTOME_HSP90_CHAPERONE_CYCLE_FOR_STEROID_HORMONE_RECEPTORS_SHR_IN_THE_PRESENCE_OF_LIGAND	
CACYBP	immune infiltration evaluations	HALLMARK_TNFA_SIGNALING_VIA_NFKB	
HSPD1	late-stage disease progression	HALLMARK_UV_RESPONSE_UP	
AHSA1	co-stimulatory profiles	HALLMARK_APOPTOSIS	
DNAJA1	tailored maturation	WP_NUCLEAR_RECEPTORS_METAPATHWAY	
HSPE1	tumor-specific immune responses	KEGG_MAPK_SIGNALING_PATHWAY	
MRPL18	directed antitumor immunity	HALLMARK_MTORC1_SIGNALING	
PPP1R15A	immune repression	KEGG_ANTIGEN_PROCESSING_AND_PRESENTATION	
SERPINH1	intestinal barrier function	WP_MAPK_SIGNALING_PATHWAY	
TSPYL2	gut immune tolerance	WP_GLUCOCORTICOID_RECEPTOR_PATHWAY	
BAG3	immune tolerance-mediated mucosal homeostasis	REACTOME_ESR_MEDIATED_SIGNALING	
DNAJB1	tumor environments	REACTOME_ESTROGEN_DEPENDENT_GENE_EXPRESSION	
HSP90AA1	disease outcome	WP_VEGFAVEGFR2_SIGNALING_PATHWAY	
IFNG	cancer immune surveillance	WP_ARYL_HYDROCARBON_RECEPTOR_PATHWAY_WP2873	
DEDD2	tumor immune status	REACTOME_SIGNALING_BY_NUCLEAR_RECEPTORS	
FKBP4	heterogeneous genomic aberrations	WP_CANCER_IMMUNOTHERAPY_BY_PD1_BLOCKADE	
HSP90AB1	gastric cancer immunotherapy	WP_UNFOLDED_PROTEIN_RESPONSE	
NEU1	subsequent immune infiltration	WP_PARKINUBIQUITIN_PROTEASOMAL_SYSTEM_PATHWAY	
RGS2	advanced gastric cancer	WP_NRF2_PATHWAY	
CHORDC1	cancer cell infection	REACTOME_SIGNALING_BY_INTERLEUKINS	
DNAJA4	epithelial regeneration	REACTOME_POTENTIAL_THERAPEUTICS_FOR_SARS	
DOK2	immunological self-tolerance	WP_APOPTOSIS_MODULATION_AND_SIGNALING	
GADD45B	cancer defense mechanisms	WP_CORTICOTROPINRELEASING_HORMONE_SIGNALING_PATHWAY	
NUDC	cancer-related impairment	HALLMARK_MYC_TARGETS_V1	
BATF	impaired immune response	WP_PHOTODYNAMIC_THERAPYINDUCED_AP1_SURVIVAL_SIGNALING	
CLK1	bone disease	WP_APOPTOSISRELATED_NETWORK_DUE_TO_ALTERED_NOTCH3_IN_OVARIAN_CANCER	
NR4A1	chemokine receptors	WP_TCELL_ANTIGEN_RECEPTOR_TCR_PATHWAY_DURING_STAPHYLOCOCCUS_AUREUS_INFECTION	
NR4A2	cancer evolution	WP_MEASLES_VIRUS_INFECTION	
PMAIP1	tumor evolution factors	REACTOME_SEMA3A_PAK_DEPENDENT_AXON_REPULSION	
RHOB	engineered macrophage-based therapies	REACTOME_THE_NLRP3_INFLAMMASOME	
SQSTM1	peritoneal dissemination	REACTOME_SEMAPHORIN_INTERACTIONS	
TAF7	gastric cancer development	WP_OREXIN_RECEPTOR_PATHWAY	
TCP1	intestinal-type gastric carcinoma	REACTOME_SARS_COV_INFECTIONS	
TNFSF14	antigen-presenting functions	WP_CHROMOSOMAL_AND_MICROSATELLITE_INSTABILITY_IN_COLORECTAL_CANCER	
JUN	enhanced anti-tumor immune response	REACTOME_INFLAMMASOMES	
DUSP1	elicit adaptive immune response	REACTOME_CHAPERONE_MEDIATED_AUTOPHAGY	
CCL3	ovarian carcinoma	WP_IL18_SIGNALING_PATHWAY	
CCL4L2	noncancer immune responses	REACTOME_RAF_INDEPENDENT_MAPK1_3_ACTIVATION	
STIP1	increased cellular proliferation	REACTOME_RHOBTB2_GTPASE_CYCLE	
FOSB	tumor immune surveillance	WP_HAIR_FOLLICLE_DEVELOPMENT_CYTODIFFERENTIATION_PART_3_OF_3	
DUSP4	immune cell tissue residency	REACTOME_PURINERGIC_SIGNALING_IN_LEISHMANIASIS_INFECTION	
HSPA4	higher-order physiological functions	WP_ANDROGEN_RECEPTOR_SIGNALING_PATHWAY	
ANKRD37	cross-present melanoma-derived tumor antigens	REACTOME_MAPK6_MAPK4_SIGNALING	
TXNIP	evasion strategies	WP_INTERACTIONS_OF_NATURAL_KILLER_CELLS_IN_PANCREATIC_CANCER	
PTGES3	immune phenotype monitoring	REACTOME_RHO_GTPASE_CYCLE	
	immune cell states	REACTOME_UPTAKE_AND_ACTIONS_OF_BACTERIAL_TOXINS	
	antigen degradation	WP_SELECTIVE_EXPRESSION_OF_CHEMOKINE_RECEPTORS_DURING_TCELL_POLARIZATION	
	tumor progressive behaviors	HALLMARK_EPITHELIAL_MESENCHYMAL_TRANSITION	
	receptor function	HALLMARK_P53_PATHWAY	
	viral gene expression	REACTOME_MAPK_TARGETS_NUCLEAR_EVENTS_MEDIATED_BY_MAP_KINASES	
	tissue regeneration	REACTOME_MAPK_FAMILY_SIGNALING_CASCADES	
	aggrephagy-related immune cell subtypes	WP_PROSTAGLANDIN_SIGNALING	
	periodontal disease	REACTOME_NEUTROPHIL_DEGRANULATION	
	progression-free interval		
	disease-specific survival		
	portal inflammation		
	immune checkpoint blockade immunotherapeutic response		
	emerging immune therapies		
	current immune therapies		
	main immune checkpoints		
	bone pre-metastatic niche		
	cancer ecosystems		
	immune-epithelial interactions		
	metastatic renal cell carcinoma		
	chronic activation/exhaustion		
	anti-glioma activity		
	signal transduction		
	combinatorial protein expression		
	lymphocyte composition		
	single-agent immunotherapy		
	single-agent therapy		
	immune cell changes		
	spontaneous autoimmune diabetes		
	type 1 diabetes progression		
	core cell-level functional outputs		
	immune therapeutic effect		
	important immune functions		
	advanced cutaneous melanoma		
	parietal cell loss		
	luminal expansion		
	response heterogeneity		
	dysregulated immune reactions		
	trained immunity induction		
	proinflammatory polarization		
	divergent cellular response		
	ovarian clear cell carcinoma		
	immune cell therapeutics		
	medulla-dependent tolerance mechanisms		
	low grade glioma		
	inhibiting anticancer immunity		
	beneficial/protective effects		
	disease-promoting effects		
	anticancer effects		
	zeste homolog 2 methyltransferase		
	anti-tumor immune phenotypes		

Naive3	ICKG_annotation (ranked by pagerank score)	ORA_annotation	LLM_annotation
KLRB1	sustained tumor control	KEGG_CYTOKINE_CYTOKINE_RECEPTOR_INTERACTION	Immune Response and Inflammation Regulation (0.85)
LTB	leukemia-free survival	HALLMARK_IL2_STAT5_SIGNALING	
RORA	subsequent effector differentiation	HALLMARK_TNFA_SIGNALING_VIA_NFKB	
IL7R	master regulator genes	REACTOME_TNFS_BIND_THEIR_PHYSIOLOGICAL_RECEPTORS	
NCR3	unfolded proteins	HALLMARK_APOPTOSIS	
SPOCK2	immune-related pathways	REACTOME_TNFR2_NON_CANONICAL_NF_KB_PATHWAY	
TMIGD2	overabundant innate inflammatory cytokine produc	HALLMARK_INFLAMMATORY_RESPONSE	
CCR6	reduced tumor burden	REACTOME_CHEMOKINE_RECEPTORS_BIND_CHEMOKINES	
CTSH	subsequent immune infiltration	REACTOME_ARACHIDONIC_ACID_METABOLISM	
FKBP11	adverse prognosis		
CCL20	proper organ function		
HPGD	glioma cell progression		
IL4I1	cancer immunosurveillance		
LST1	tumour-promoting functions		
ALOX5AP	antigen-presenting neutrophil delivery		
AQP3	keratinocyte proliferation		
CEBPD	hyperinducible expression		
CXCR6	adoptive cancer immunotherapy		
GNA15	low-grade lesions		
GPR171	greater tumor infiltration		
GPR65	glioma cell population		
JAML	scar formation		
ODF2L	gradual neurodegeneration		
PERP	low-grade dysplasia		
RBMS1	antitumor treatment		
TANK	invasive pancreatic ductal adenocarcinoma		
TNFRSF25	tumour suppression		
FURIN	autoimmune disease symptoms		
IFI44	metabolic dysfunction-associated steatohepatitis		
IFNGR1	noncancer immune responses		
LGALS3	increased cellular proliferation		
PHACTR2	lymphocyte fate determination		
RBPJ	durable tumor immunity		
ANKRD28	direct anti-tumor effects		
NINJ1	cell-associated viral transcripts		
ERN1	longer progression-free survival		
TNFSF13B	low-risk subgroups		
NFKBIA	higher mutation load		
TTC39C	human immunodeficiency virus		
CELF2	long-term disease control		
HOPX	increased fibrosis		
MAF	immunocytes' activation		
GNLY	combination immunotherapy		
SATB1	alloimmune response		
CERK	long-term survivors		
GPX1	cystic fibrosis		
PFKFB3	chordoma treatment		
TNFRSF18	antibody-drug conjugate therapy		
PRDM1	chronic obstructive pulmonary disease		
SNX9	cell-based immunotherapy		
	reduced terminal exhaustion		
	increased memory cell characteristics		
	adaptive anti-tumor immunity		
	lineage-specific gene programs		
	dense tumor infiltration		
	enhanced immune cell recruitment		
	leukemia-like disease		
	sustained antitumor activity		
	tumor biomarker analyses		
	colitis-associated tumorigenesis		
	anti-tumor dysfunction		

Dysfunction	ICKG_annotation (ranked by pagerank score)	ORA_annotation	LLM_annotation
TNFRSF18	tumoricidal factors	HALLMARK_IL2_STAT5_SIGNALING	T-cell Activation and Immune Response Regulation (0.92)
ARID5B	disease susceptibility	KEGG_CYTOKINE_CYTOKINE_RECEPTOR_INTERACTION	
BATF	tumor eradication	REACTOME_TNFR2_NON_CANONICAL_NF_KB_PATHWAY	
NR3C1	90% tumor response	REACTOME_TNFS_BIND_THEIR_PHYSIOLOGICAL_RECEPTORS	
TIGIT	anti-tumor efficacy	HALLMARK_TNFA_SIGNALING_VIA_NFKB	
CXCL13	autoimmune disease	WP_FOXP3_IN_COVID19	
PKM	anti-tumour immune responses	HALLMARK_IL6_JAK_STAT3_SIGNALING	
TNFRSF4	cancer immunosurveillance	REACTOME_RUNX1_AND_FOXP3_CONTROL_THE_DEVELOPMENT_OF_REGULATORY_T_LYMPHOCYTES_TREGS	
UCP2	tumour-promoting functions	WP_MAMMARY_GLAND_DEVELOPMENT_PATHWAY_INVOLUTION_STAGE_4_OF_4	
C9ORF16	early tertiary lymphoid structure	WP_THYMIC_STROMAL_LYMPHOPOIETIN_TSLP_SIGNALING_PATHWAY	
CD27	anti-tumour immunity	HALLMARK_INFLAMMATORY_RESPONSE	
DNPH1	malignant tumor immunity	REACTOME_INTERLEUKIN_27_SIGNALING	
BIRC3	exhaustion markers	REACTOME_INTERLEUKIN_6_SIGNALING	
CTLA4	ischemia reperfusion injury-related phenotyp	REACTOME_INTERLEUKIN_35_SIGNALLING	
LTB	long-term protective immunity	WP_TCELL_ANTIGEN_RECEPTOR_TCR_PATHWAY_DURING_STAPHYLOCOCCUS_AUREUS_INFECTION	
PARK7	immune-regulation-related signaling pathway	WP_ONCOSTATIN_M_SIGNALING_PATHWAY	
PMAIP1	complete tumour regression	REACTOME_TNF_RECEPTOR_SUPERFAMILY_TNFSF_MEMBERS_MEDIATING_NON_CANONICAL_NF_KB_PATHWAY	
TNFRSF9	cancer cell extravasation	WP_NCRNAS_INVOLVED_IN_STAT3_SIGNALING_IN_HEPATOCELLULAR_CARCINOMA	
CNIH1	immune-based cancer therapies	WP_GENES_ASSOCIATED_WITH_THE_DEVELOPMENT_OF_RHEUMATOID_ARTHRITIS	
DUSP4	longer progression-free survival		
GBP2	low-risk subgroups		
IL6ST	liver metastasis formation		
ITM2A	tumour suppression		
NFKBIA	cancer cell transmigration		
PEBP1	effector function inhibition		
PGAM1	critical immune factors		
SPOCK2	immune system balance		
TBC1D4	combination immunotherapy		
CD82	personalized cancer vaccination strategies		
CORO1B	immune cell fate		
GEM	natural killer cell activity		
ICA1	elicit adaptive immune response		
IL7R	tumor immune surveillance		
KLRB1	noncancer immune responses		
MAF	increased cellular proliferation		
MAGEH1	metabolic dysfunction-associated steatohepatitis		
RBPJ	cross-present melanoma-derived tumor antigens		
SNX9	immune cell tissue residency		
STAT3	established signal transduction pathways		
AP2M1	higher-order physiological functions		
CCR7	cardiac xenotransplantation		
CHN1	immune phenotype monitoring		
CRTAM	immune cell states		
EIF3I	strong inhibition		
FKBP1A	intra-tumoral pathological response		
FKBP5	elicit adaptive immune responses		
GPX4	leukemia/lymphoma conditions		
HSPB1	robust transduction		
ICOS	melanoma metastases		
IGFLR1	emerging immune therapies		
	current immune therapies		
	cancer immunotherapy strategies		
	increased fibrosis		
	human immune function		
	tumor-mediated immune escape		
	cancer ecosystems		
	immune-epithelial interactions		
	main immune checkpoints		
	bone pre-metastatic niche		
	chronic activation/exhaustion		
	anti-glioma activity		
	signal transduction		
	combinatorial protein expression		
	lymphocyte composition		
	single-agent immunotherapy		
	single-agent therapy		
	immune cell changes		
	spontaneous autoimmune diabetes		
	type 1 diabetes progression		
	core cell-level functional outputs		
	immune therapeutic effect		
	adaptive immune checkpoint resistance		
	immune cell recruitment		
	treatment-associated tumor necrosis		
	lineage-specific gene programs		
	cell-cycle progression		
	immunocytes' activation		
	transplantable mouse tumor models		
	luminal expansion		
	peripheral angiogenesis		
	endogenous adaptive antitumour immunity		
	post-ablation immune therapy		
	chronic obstructive pulmonary disease		
	cystic fibrosis		
	immune cell therapeutics		
	ovarian pathogenesis		
	retrovirus immunity		
	lymphocyte activatory receptors		
	antitumor functions		
	inhibiting anticancer immunity		
	beneficial/protective effects		
	disease-promoting effects		
	anticancer effects		
	low grade glioma		
	host antitumor immunity		
	anti-tumor dysfunction		

NK_cytotoxicity	KG_annotation (ranked by pagerank scor	ORA_annotation	LLM_annotation
PRF1	disease progression	WP_ALLOGRAFT_REJECTION	Cytotoxic T-cell Mediated Immunity (0.95)
GZMH	cell death	KEGG_ALLOGRAFT_REJECTION	
GZMA	autoimmune diseases	KEGG_GRAFT_VERSUS_HOST_DISEASE	
GZMB	radiation-induced pyroptosis	KEGG_TYPE_I_DIABETES_MELLITUS	
GZMM	antitumor immune effect	HALLMARK_ALLOGRAFT_REJECTION	
FASLG	anti-tumour activity	KEGG_AUTOIMMUNE_THYROID_DISEASE	
GNLY	anti-tumor immune responses	WP_APOPTOSIS	
NKG7	death receptor-mediated apoptosis	KEGG_NATURAL_KILLER_CELL_MEDIATED_CYTOTOXICITY	
	graft rejection	WP_NANOMATERIAL_INDUCED_APOPTOSIS	
	auto-immune attack	WP_INTERACTIONS_OF_NATURAL_KILLER_CELLS_IN_PANCREATIC_CANCER	
	death receptors	REACTOME_REGULATED_NECROSIS	
	tumor immunoregulation	HALLMARK_APOPTOSIS	
	pore-forming amino-terminal fragments	REACTOME_APOPTOSIS	
	excessive matrix degradation	HALLMARK_COMPLEMENT	
	hemophagocytic lymphohistiocytosis	REACTOME_PROGRAMMED_CELL_DEATH	
	cytotoxic cytokine secretion	REACTOME_REGULATION_BY_C_FLIP	
	tumor cell pyroptosis	REACTOME_NOTCH2_INTRACELLULAR_DOMAIN_REGULATES_TRANSCRIPTION	
	vascular disease	REACTOME_CASPASE_ACTIVATION_VIA_DEATH_RECEPTORS_IN_THE_PRESENCE_OF_LIGAND	
	liver cancer immunotherapy	REACTOME_FOXO_MEDIATED_TRANSCRIPTION_OF_CELL_DEATH_GENES	
	immune cell-induced antitumor response	REACTOME_METABOLISM_OF_ANGIOTENSINOGEN_TO_ANGIOTENSINS	
	superior anti-tumor activity	WP_APOPTOSIS_MODULATION_BY_HSP70	
	durable immune control	WP_HEMATOPOIETIC_STEM_CELL_GENE_REGULATION_BY_GABP_ALPHABETA_COMPLEX	
	neovasculature formation	WP_HOSTPATHOGEN_INTERACTION_OF_HUMAN_CORONAVIRUSES_APOPTOSIS	
	immune-enhancing chemokines	REACTOME_DEFECTIVE_INTRINSIC_PATHWAY_FOR_APOPTOSIS	
	enhanced antifungal effector responses	REACTOME_CASPASE_ACTIVATION_VIA_EXTRINSIC_APOPTOTIC_SIGNALLING_PATHWAY	
	long-term cytomegalovirus control	REACTOME_PYROPTOSIS	
	lung tumourigenesis	REACTOME_RIPK1_MEDIATED_REGULATED_NECROSIS	
	immune system homeostasis	REACTOME_TNFS_BIND_THEIR_PHYSIOLOGICAL_RECEPTORS	
	autoimmune adverse events	REACTOME_SIGNALING_BY_NOTCH2	
	immune overactivation		
	cancer cell transmigration		
	global flaviviruses		
	virus infections		
	heat shock protein		

NK_inhibitory	ICKG_annotation (ranked by pagerank score)	ORA_annotation	LLM_annotation
KIR2DL3	cytokine secretion	REACTOME_IMMUNOREGULATORY_INTERACTIONS_BETWEEN_A_LYMPHOID_AND_A_NON_LYMP	Natural Killer Cell Inhibitory Signaling (0.92)
KIR2DL4	polymorphic self-antigen	KEGG_ANTIGEN_PROCESSING_AND_PRESENTATION	
KLRC1	long-lasting tumor regression	KEGG_NATURAL_KILLER_CELL_MEDIATED_CYTOTOXICITY	
TIGIT	cancer progression	WP_INTERACTIONS_OF_NATURAL_KILLER_CELLS_IN_PANCREATIC_CANCER	
CD96	rheumatoid arthritis	KEGG_GRAFT_VERSUS_HOST_DISEASE	
	donor ligand expression patterns		
	inhibitory immune checkpoints		
	sepsis-induced loss		
	neoplastic cell evasion		
	immune checkpoint receptors		
	complete tumor eradication		
	gastric cancer immunotherapy		
	favorable anticancer responses		
	effective anticancer immunity		
	gastric cancer pathogenesis		
	terminal differentiation		
	interferon gamma		
	antibody-independent pathological functions		
	pre-transplant donor-induced release		
	tumor environment		
	tumor sites		
	chemokine production		
	increased tumor rejection		
	antibody dependent cellular cytotoxicity		
	innate immune functions		
	reduced tumor incidence		
	substantial tissue specificity		
	colorectal cancer surgery		
	validation sets		
	downstream immune responses		
	anti-viral effects		
	key effector genes		
	elicit anticancer immunity		
	adverse cytogenetics		
	fetal-maternal tolerance		
	immune modulation		
	cell migration		
	efficient complex formation		
	subsequent antibody-dependent cellular cytotoxicity		
	antibody-bound targets		
	immune stimulatory effects		
	transient peripheral transcriptomic alterations		
	orchestrated suppression		
	profound local suppression		
	increased tumor-associated macrophages		

NK_stimulatory	ICKG_annotation (ranked by pagerank score)	ORA_annotation	LLM_annotation
FCGR3A	antibody-dependent cellular cytotoxicity	KEGG_NATURAL_KILLER_CELL_MEDIATED_CYTOTOXICITY	Natural Killer Cell Activation and Cytotoxicity (0.92)
NCR3	memory-like induction	WP_INTERACTIONS_OF_NATURAL_KILLER_CELLS_IN_PANCREATIC_CANCER	
KLRF1	memory-like properties	REACTOME_IMMUNOREGULATORY_INTERACTIONS_BETWEEN_A_LYMPHOID_AND_A_NON_LYMPHOID_CELL	
KLRK1	better anti-tumor activity	REACTOME_DAP12_SIGNALING	
KLRC2	immune stimulatory proteins	REACTOME_DAP12_INTERACTIONS	
	cytokine secretion		
	antibody dependent cellular cytotoxicity		
	elicit anticancer immunity		
	tumor immunity		
	enhanced anti-tumor function		
	tumor cell lysis		
	intratumor retention		
	antibody-based cancer therapies		
	robust antitumor activity		
	neutrophil-mediated cytotoxicity		
	efficient complex formation		
	subsequent antibody-dependent cellular cytotoxicity		
	long-lasting tumor regression		
	immune-tolerance		
	immune stimulatory effects		
	antibody-bound targets		
	transient peripheral transcriptomic alterations		
	cancer treatments		
	proven antitumor effects		
	biphenotypic acute lymphoblastic leukemia		
	complete tumor eradication		
	interferon gamma		
	sepsis-induced loss		
	immune checkpoint receptors		
	divergent immune functions		
	antitumor immune effect		
	optimized innate immune responses		
	melanoma control		
	cytotoxicity programs		
	glioblastoma multiforme		
	specific tumor target recognition		
	allelic expression status		
	gene expression status		
	concomitant uterine carcinosarcoma		
	immune-dependent control		
	enhanced cytotoxic function		
	leukemogenesis originates		
	severe acute respiratory syndrome		
	human coronavirus		
	lymphoid precursor cells		
	granuloma formation		
	cancer-suppressive immune system interactions		
	cancer immunotherapy approaches		
	epidermal growth factor receptor		
	efficient immune synapse formation		
	enhanced anti-tumor activity		
	gene-regulatory networks		
	adaptive-like responses		
	stress-associated activating signals		
	natural killer cell-mediated therapy		
	complement-dependent cytotoxicity		
	excessive matrix degradation		
	neuroectodermal origin		
	cell killing		
	immune sensing pathway		
	immune overactivation		
	colorectal cancer surgery		
	melanoma development		
	autoimmune-mediated neuroinflammation		
	death receptor ligand expression		
	intra-lineage plasticity		
	cell-intrinsic tumor regulatory pathways		
	histone deacetylase		
	recurrent glioblastoma		
	convection enhanced delivery		
	gene delivery		
	endogenous immune recognition		
	normal cell physiology		
	restrained immune evasion		
	reduced immune evasion		
	dose ingestion		
	cellular infection		
	esophageal mucosa carcinogenesis		
	disease phenotypes		
	adaptive anticancer immune responses		
	disseminated nontuberculous mycobacterial infections		
	clinical presentation		
	life-threatening complications		
	higher expression		
	profound immune responses		
	increased tumor-associated macrophages		

NK_TaNK	KG_annotation (ranked by pagerank scor	ORA_annotation	LLM_annotation
HSPA1A	prior antigen presentation	HALLMARK_TNFA_SIGNALING_VIA_NFKB	Heat Shock Response and Cellular Stress Management (0.92)
HSPA1B	pro-inflammatory cytokines	REACTOME_ATTENUATION_PHASE	
HSPA6	antitumor immune effect	REACTOME_HSF1_DEPENDENT_TRANSACTIVATION	
DNAJB1	antitumor vaccine candidates	REACTOME_HSF1_ACTIVATION	
BAG3	efficient immune synapse formation	REACTOME_HSP90_CHAPERONE_CYCLE_FOR_STEROID_HORMONE_RECEPTORS_SHR_IN_THE_PRESENCE_OF_LIGAND	
HSPH1	human lung epithelial development	REACTOME_CELLULAR_RESPONSE_TO_HEAT_STRESS	
NR4A1	basal cells	WP_OREXIN_RECEPTOR_PATHWAY	
SERPINE1	immune cell-based therapies	REACTOME_NGF_STIMULATED_TRANSCRIPTION	
SERPINH1	mediated immune escape	REACTOME_REGULATION_OF_HSF1_MEDIATED_HEAT_SHOCK_RESPONSE	
ATF3	heat shock protein	HALLMARK_INFLAMMATORY_RESPONSE	
DNAJB4	vascular disease	WP_IL18_SIGNALING_PATHWAY	
HSPD1	pro-inflammatory cells	REACTOME_NUCLEAR_EVENTS_KINASE_AND_TRANSCRIPTION_FACTOR_ACTIVATION	
DNAJA4	tumor regression	WP_NUCLEAR_RECEPTORS_METAPATHWAY	
DLX2	optimized innate immune responses	KEGG_MAPK_SIGNALING_PATHWAY	
RASL11A	disease remission	HALLMARK_HYPOXIA	
PGF	increased antitumor potential	HALLMARK_P53_PATHWAY	
HSPE1	lasting tumor control	HALLMARK_APOPTOSIS	
HES1	cytotoxicity programs	WP_GLUCOCORTICOID_RECEPTOR_PATHWAY	
ARG2	cytokine-induced killer cells	HALLMARK_UV_RESPONSE_UP	
HSPA2	immune-dependent control	WP_MAPK_SIGNALING_PATHWAY	
RHOB	receptor restimulation	WP_PROSTAGLANDIN_SIGNALING	
GEM	immune consequences	HALLMARK_IL2_STAT5_SIGNALING	
HSP90AA1	tumor-directed effects	WP_APOPTOSISRELATED_NETWORK_DUE_TO_ALTERED_NOTCH3_IN_OVARIAN_CANCER	
FOSL1	adaptive antitumor immunity	REACTOME_INTERLEUKIN_4_AND_INTERLEUKIN_13_SIGNALING	
DNAJA1	unchecked systemic inflammation	REACTOME_SIGNALING_BY_NTRKS	
IFNG	anti-tumor immune surveillance	WP_TNFRELATED_WEAK_INDUCER_OF_APOPTOSIS_TWEAK_SIGNALING_PATHWAY	
BAMBI	autoimmune-mediated neuroinflammatic	WP_ONCOSTATIN_M_SIGNALING_PATHWAY	
ANKRD37		WP_VEGFAVEGFR2_SIGNALING_PATHWAY	
TNFSF9		KEGG_NOD_LIKE_RECEPTOR_SIGNALING_PATHWAY	
CACYBP		WP_HYPERTROPHY_MODEL	
RRAD		WP_ADIPOGENESIS	
SNAI1		WP_ARYL_HYDROCARBON_RECEPTOR_PATHWAY_WP2873	
RGS16		WP_PHOTODYNAMIC_THERAPYINDUCED_NFKB_SURVIVAL_SIGNALING	
ULBP2		WP_PHOTODYNAMIC_THERAPYINDUCED_AP1_SURVIVAL_SIGNALING	
JUN		WP_GASTRIN_SIGNALING_PATHWAY	
B3GNT5		WP_PARKINUBIQUITIN_PROTEASOMAL_SYSTEM_PATHWAY	
FOSB		REACTOME_RAF_INDEPENDENT_MAPK1_3_ACTIVATION	
PPP1R15A		WP_CORTICOTROPINRELEASING_HORMONE_SIGNALING_PATHWAY	
CDKN1A		WP_TGFBETA_RECEPTOR_SIGNALING	
EGR3		WP_QUERCETIN_AND_NFKB_AP1_INDUCED_APOPTOSIS	
HSP90AB1		WP_TCELL_ANTIGEN_RECEPTOR_TCR_PATHWAY_DURING_STAPHYLOCOCCUS_AUREUS_INFECTION	
RBKS		WP_TGFBETA_RECEPTOR_SIGNALING_IN_SKELETAL_DYSPLASIAS	
IER3		WP_BURN_WOUND_HEALING	
NEU1		WP_APOPTOSIS	
RASGEF1B		WP_MEASLES_VIRUS_INFECTION	
FKBP4		KEGG_ANTIGEN_PROCESSING_AND_PRESENTATION	
HSPB1		HALLMARK_ESTROGEN_RESPONSE_EARLY	
MUC12		HALLMARK_INTERFERON_GAMMA_RESPONSE	
PLK2		HALLMARK_MTORC1_SIGNALING	
FSCN1		WP_ARYL_HYDROCARBON_RECEPTOR_PATHWAY_WP2586	
ID1		WP_APOPTOSIS_MODULATION_AND_SIGNALING	
ADM		REACTOME_SIGNALING_BY_INTERLEUKINS	
EGR1		WP_TNFALPHA_SIGNALING_PATHWAY	
CDKN1C		WP_SMALL_CELL_LUNG_CANCER	
FOS		HALLMARK_TGF_BETA_SIGNALING	
SPRY2		WP_RANKLRANK_SIGNALING_PATHWAY	
CD83		WP_EBSTEINBARR_VIRUS_LMP1_SIGNALING	
ZFAND2A		HALLMARK_ESTROGEN_RESPONSE_LATE	
EGR2		WP_OXIDATIVE_DAMAGE_RESPONSE	
CHORDC1		WP_IL1_AND_MEGAKARYOCYTES_IN_OBESITY	
DUSP4		WP_TCELL_ACTIVATION_SARSCOV2	
DUSP1		REACTOME_NEGATIVE_REGULATION_OF_MAPK_PATHWAY	
ICAM1		WP_TCELL_RECEPTOR_TCR_SIGNALING_PATHWAY	
CSRNP1		WP_SPINAL_CORD_INJURY	
PHLDA1		WP_NETWORK_MAP_OF_SARSCOV2_SIGNALING_PATHWAY	
NR4A2		WP_P53_TRANSCRIPTIONAL_GENE_NETWORK	
MMP9		WP_MYOMETRIAL_RELAXATION_AND_CONTRACTION_PATHWAYS	
RASD1		WP_OVERVIEW_OF_PROINFLAMMATORY_AND_PROFIBROTIC_MEDIATORS	
ID3		WP_VITAMIN_B12_METABOLISM	
TNF		WP_TGFBETA_SIGNALING_PATHWAY	
IER5L		HALLMARK_EPITHELIAL_MESENCHYMAL_TRANSITION	
PHLDA2		HALLMARK_KRAS_SIGNALING_UP	
ARL4D		REACTOME_NUCLEOTIDE_BINDING_DOMAIN_LEUCINE_RICH_REPEAT_CONTAINING_RECEPTOR_NLR_SIGNALING_PATHWAYS	
RGCC		KEGG_T_CELL_RECEPTOR_SIGNALING_PATHWAY	
GLA		REACTOME_SELECTIVE_AUTOPHAGY	
DUSP2		WP_PHOTODYNAMIC_THERAPYINDUCED_HIF1_SURVIVAL_SIGNALING	
RND1		HALLMARK_UNFOLDED_PROTEIN_RESPONSE	
MYADM		WP_PREIMPLANTATION_EMBRYO	
HES4		REACTOME_TP53_REGULATES_TRANSCRIPTION_OF_ADDITIONAL_CELL_CYCLE_GENES_WHOSE_EXACT_ROLE_IN_THE_P53_PATHWAY_REMAIN_UNCERTAIN	
DUSP8		WP_GALANIN_RECEPTOR_PATHWAY	
NKRF		WP_HAIR_FOLLICLE_DEVELOPMENT_CYTODIFFERENTIATION_PART_3_OF_3	
NLRP3		REACTOME_CHAPERONE_MEDIATED_AUTOPHAGY	
GADD45B		WP_STING_PATHWAY_IN_KAWASAKILIKE_DISEASE_AND_COVID19	
NFKBIZ		WP_VITAMIN_D_IN_INFLAMMATORY_DISEASES	
SERTAD1		WP_VITAMIN_D_RECEPTOR_PATHWAY	
IER5		WP_KYNURENINE_PATHWAY_AND_LINKS_TO_CELL_SENESCENCE	
RGS2		KEGG_BLADDER_CANCER	
DDIT3		WP_FAS_LIGAND_PATHWAY_AND_STRESS_INDUCTION_OF_HEAT_SHOCK_PROTEINS	
MRPL18		KEGG_P53_SIGNALING_PATHWAY	
DUSP5		REACTOME_MYD88_INDEPENDENT_TLR4_CASCADE	
AREG		WP_SUDDEN_INFANT_DEATH_SYNDROME_SIDS_SUSCEPTIBILITY_PATHWAYS	
CKS2		KEGG_LEISHMANIA_INFECTION	
ERRFI1		REACTOME_TNFR2_NON_CANONICAL_NF_KB_PATHWAY	
GADD45G		WP_FOLATE_METABOLISM	
LMNA		WP_CHROMOSOMAL_AND_MICROSATELLITE_INSTABILITY_IN_COLORECTAL_CANCER	
HSPA8		REACTOME_TP53_REGULATES_TRANSCRIPTION_OF_CELL_CYCLE_GENES	
PMAIP1		WP_HEPATITIS_C_AND_HEPATOCELLULAR_CARCINOMA	
PLK3		WP_NEUROINFLAMMATION	
TNFRSF12A		REACTOME_TNFR1_INDUCED_NFKAPPAB_SIGNALING_PATHWAY	
CD69		REACTOME_ESR_MEDIATED_SIGNALING	
ZC3H12A		WP_NRP1TRIGGERED_SIGNALING_PATHWAYS_IN_PANCREATIC_CANCER	
C17orf67		WP_DEVELOPMENT_AND_HETEROGENEITY_OF_THE_ILC_FAMILY	
VEGFA		WP_DNA_DAMAGE_RESPONSE_ONLY_ATM_DEPENDENT	
HSPA1L		WP_HOSTPATHOGEN_INTERACTION_OF_HUMAN_CORONAVIRUSES_INTERFERON_INDUCTION	
CREM		REACTOME_RESPONSE_OF_EIF2AK1_HRI_TO_HEME_DEFICIENCY	
NOTCH4		REACTOME_AUTOPHAGY	
NFKBIA		WP_HEPATITIS_B_INFECTION	
PLIN2		REACTOME_P75NTR_SIGNALS_VIA_NF_KB	
BTG2		REACTOME_THE_NLRP3_INFLAMMASOME	
ATP1B1		REACTOME_REGULATION_OF_TNFR1_SIGNALING	
UBE2S		WP_SIGNAL_TRANSDUCTION_THROUGH_IL1R	
KDM6B		WP_HOSTPATHOGEN_INTERACTION_OF_HUMAN_CORONAVIRUSES_MAPK_SIGNALING	
TNFAIP3		WP_SELENIUM_MICRONUTRIENT_NETWORK	
AHSA1		REACTOME_TNF_RECEPTOR_SUPERFAMILY_TNFSF_MEMBERS_MEDIATING_NON_CANONICAL_NF_KB_PATHWAY	
NR4A3		KEGG_PATHWAYS_IN_CANCER	
SPRY1		WP_NEOVASCULARISATION_PROCESSES	
SGK1		WP_MIRNAS_INVOLVEMENT_IN_THE_IMMUNE_RESPONSE_IN_SEPSIS	
ICOS		KEGG_SPLICEOSOME	
ZNF331		REACTOME_TOLL_LIKE_RECEPTOR_9_TLR9_CASCADE	
TUBB2A		WP_CIRCADIAN_RHYTHM_GENES	
NFKBIE		WP_PDGF_PATHWAY	
MPZL3		WP_MYD88_DISTINCT_INPUTOUTPUT_PATHWAY	
TSC22D1		WP_BLADDER_CANCER	
ARL5B		WP_NUCLEOTIDEBINDING_OLIGOMERIZATION_DOMAIN_NOD_PATHWAY	
TSPYL2		WP_NEURAL_CREST_DIFFERENTIATION	
TNFSF14		REACTOME_TOLL_LIKE_RECEPTOR_TLR1_TLR2_CASCADE	
DEDD2		REACTOME_INTERLEUKIN_17_SIGNALING	
THBS1		WP_NONGENOMIC_ACTIONS_OF_125_DIHYDROXYVITAMIN_D3	
TNFRSF9		REACTOME_INFLAMMASOMES	
RNF122		REACTOME_AGGREPHAGY	
JUND		REACTOME_TNF_SIGNALING	
ELL2		REACTOME_DEATH_RECEPTOR_SIGNALLING	
SLC5A3		KEGG_CYTOKINE_CYTOKINE_RECEPTOR_INTERACTION	
STIP1		WP_BRAINDERIVED_NEUROTROPHIC_FACTOR_BDNF_SIGNALING_PATHWAY	
XCL1		KEGG_B_CELL_RECEPTOR_SIGNALING_PATHWAY	
IFRD1		REACTOME_EXTRA_NUCLEAR_ESTROGEN_SIGNALING	
KBTBD8		WP_NRF2_PATHWAY	
TIPARP		WP_CANCER_IMMUNOTHERAPY_BY_PD1_BLOCKADE	
BIRC3		WP_ESTROGEN_SIGNALING_PATHWAY	
RELB		REACTOME_ESTROGEN_DEPENDENT_GENE_EXPRESSION	
MCL1		WP_NOCGMPPKG_MEDIATED_NEUROPROTECTION	
HSPA4		REACTOME_THE_ROLE_OF_GTSE1_IN_G2_M_PROGRESSION_AFTER_G2_CHECKPOINT	
TRAF1		WP_UNFOLDED_PROTEIN_RESPONSE	
TUBB4B		REACTOME_DDX58_IFIH1_MEDIATED_INDUCTION_OF_INTERFERON_ALPHA_BETA	
MXD1		REACTOME_TOLL_LIKE_RECEPTOR_CASCADES	
SLC19A2		REACTOME_FORMATION_OF_TUBULIN_FOLDING_INTERMEDIATES_BY_CCT_TRIC	
ZNF10		REACTOME_PURINERGIC_SIGNALING_IN_LEISHMANIASIS_INFECTION	
ARL4A		WP_BREAST_CANCER_PATHWAY	
PHYH		KEGG_TGF_BETA_SIGNALING_PATHWAY	
BCO2		REACTOME_POTENTIAL_THERAPEUTICS_FOR_SARS	
KLF4		REACTOME_SIGNALING_BY_NUCLEAR_RECEPTORS	
KLF6		REACTOME_TNFS_BIND_THEIR_PHYSIOLOGICAL_RECEPTORS	
FAM53C		WP_PHOTODYNAMIC_THERAPYINDUCED_UNFOLDED_PROTEIN_RESPONSE	
NAF1		WP_RAS_AND_BRADYKININ_PATHWAYS_IN_COVID19	
SLC16A1		KEGG_PROSTATE_CANCER	
STX11		WP_PANCREATIC_ADENOCARCINOMA_PATHWAY	
CYSLTR2		WP_TLR4_SIGNALING_AND_TOLERANCE	
TIFA		REACTOME_ACTIVATION_OF_THE_AP_1_FAMILY_OF_TRANSCRIPTION_FACTORS	
LDLR		WP_DEGRADATION_PATHWAY_OF_SPHINGOLIPIDS_INCLUDING_DISEASES	
CCL4L2		REACTOME_EGFR_DOWNREGULATION	
RIPK2		REACTOME_MAPK_TARGETS_NUCLEAR_EVENTS_MEDIATED_BY_MAP_KINASES	
OSM			
PDE4B			
MLF1			
CTNNAL1			
MAP1LC3A			
AMD1			
RBBP8			
NFKB1			
GPR35			
GPR18			
NAB2			
TOB1			
DUSP10			
BPGM			
LETM2			
TAGAP			
BCL6			
ZNF597			
TCP1			
KCNA3			
IRF8			
PFKFB3			
MAFF			
RGS1			
JUNB			
EIF4A3			
RAB30			
AHR			
HIC1			
BTN2A2			
CASS4			
DYNLL1			
NABP1			
DNAJB6			
FAM13A			
PTGER4			
B3GAT2			

Lung-Hanada	ICKG_annotation (ranked by pagerank score)	ORA_annotation	LLM_annotation
CXCL13	heme oxygenase	REACTOME_PD_1_SIGNALING	T-cell Activation and Immune Response Regulation (0.85)
ENTPD1	vaccine-mediated antibody response	WP_ALLOGRAFT_REJECTION	
BATF	melanoma cell ferroptosis	KEGG_ALLOGRAFT_REJECTION	
GZMB	disease-free survival	REACTOME_GENERATION_OF_SECOND_MESSENGER_MOLECULES	
CD27	complete tumor eradication	KEGG_GRAFT_VERSUS_HOST_DISEASE	
TIGIT	anti-tumor efficacy	KEGG_TYPE_I_DIABETES_MELLITUS	
PHLDA1	decreased antigen recognition ability	HALLMARK_ALLOGRAFT_REJECTION	
CD74	liver tumor progression	KEGG_AUTOIMMUNE_THYROID_DISEASE	
HLA-DRA	insulin sensitivity	REACTOME_COSTIMULATION_BY_THE_CD28_FAMILY	
HLA-DRB1	coinhibitory receptors	WP_CANCER_IMMUNOTHERAPY_BY_PD1_BLOCKADE	
HLA-DPB1	anti-tumour immune responses	KEGG_HEMATOPOIETIC_CELL_LINEAGE	
CD3D	pro-tumor activities	KEGG_ANTIGEN_PROCESSING_AND_PRESENTATION	
CD82	severe infection	KEGG_ASTHMA	
ARL3	tumor tissue angiogenesis	HALLMARK_P53_PATHWAY	
HMOX1	early tertiary lymphoid structure	REACTOME_TCR_SIGNALING	
ALOX5AP	co-culture systems	REACTOME_MHC_CLASS_II_ANTIGEN_PRESENTATION	
DUSP4	disease susceptibility	KEGG_INTESTINAL_IMMUNE_NETWORK_FOR_IGA_PRODUCTION	
CARS	anti-tumour immunity	KEGG_VIRAL_MYOCARDITIS	
LSP1	specific cancer-related gene sets	KEGG_LEISHMANIA_INFECTION	
CCND2	failed cancer cell elimination	WP_TCELL_ACTIVATION_SARSCOV2	
TPI1	exhaustion markers	REACTOME_INTERFERON_GAMMA_SIGNALING	
GAPDA	ischemia reperfusion injury-related phenotypes	WP_PATHOGENESIS_OF_SARSCOV2_MEDIATED_BY_NSP9NSP10_COMPLEX	
ITM2A	acidic substance-related gene sets	REACTOME_PURINERGIC_SIGNALING_IN_LEISHMANIASIS_INFECTION	
HMGN3	heightened tumor cell metabolism	WP_CYTOKINES_AND_INFLAMMATORY_RESPONSE	
CHST12	dysregulated perfusion	WP_INTERACTIONS_OF_NATURAL_KILLER_CELLS_IN_PANCREATIC_CANCER	
NAP1L4	complete tumour regression	WP_EBOLA_VIRUS_INFECTION_IN_HOST	
NEGATIVE	relapsed/refractory multiple myeloma	KEGG_CELL_ADHESION_MOLECULES_CAMS	
IL7R	immune-based cancer therapies	KEGG_PRIMARY_IMMUNODEFICIENCY	
TPT1	protective immune memory	KEGG_SYSTEMIC_LUPUS_ERYTHEMATOSUS	
RPS12	progressive disease	REACTOME_ACTIVATION_OF_THE_MRNA_UPON_BINDING_OF_THE_CAP_BINDING_COMPLEX_AND_EIFS_AND_SUBSEQUENT_BINDING_TO_43S	
RPS16	effector function inhibition	HALLMARK_IL2_STAT5_SIGNALING	
S100A10	immune checkpoint inhibitor	HALLMARK_TNFA_SIGNALING_VIA_NFKB	
	ectopic accumulation	REACTOME_INTERFERON_SIGNALING	
	immune system balance	KEGG_P53_SIGNALING_PATHWAY	
	cytotoxic antitumor effects		
	natural killer cell activity		
	tertiary lymphoid structure-like lymphocytic aggregates		
	allotransplant rejection		
	cross-present melanoma-derived tumor antigens		
	cardiac xenotransplantation		
	Œ≥Œ¥ cell-based therapies		
	Œ≥Œ¥ reconstitution		
	paraneoplastic cerebellar degeneration		
	melanoma metastases		
	robust transduction		
	marrow-residence genes		
	drug-resistant tumors		
	anti-leukemia immunosurveillance		
	virus-neutralizing antibodies		
	dysregulated tumor glycolysis		
	impaired anti-tumor activity		
	insufficient infiltration		
	autoimmune hepatitis		
	lineage-specific gene programs		
	immune cell recruitment		
	reduced terminal exhaustion		
	increased memory cell characteristics		
	immune-edited tumors		
	adaptive anti-tumor immunity		
	previously known tumor antigens		
	glioblastoma immunotherapy		
	transplantable mouse tumor models		
	potent antitumor cytotoxicity		
	endogenous adaptive antitumour immunity		
	dense tumor infiltration		
	post-ablation immune therapy		
	local tissue protection		
	cancer microvessels		
	peripheral immune tolerance		
	multiple immune checkpoints		
	post allogeneic hematopoietic stem cell transplantation		
	cancer immunoprevention		
	monocyte differentiation		
	organ-adapted protection		
	treatment-associated tumor necrosis		

PDAC-Meng	ICKG_annotation (ranked by pagerank score)	ORA_annotation	LLM_annotation
GZMA	tumor growth	WP_ALLOGRAFT_REJECTION	Cytotoxic T-cell Mediated Immunity (0.92)
LSP1	fulminant inflammatory bowel disease	HALLMARK_ALLOGRAFT_REJECTION	
LY6E	sustained tumor control	KEGG_ALLOGRAFT_REJECTION	
FKBP1A	disease-free survival	KEGG_GRAFT_VERSUS_HOST_DISEASE	
ACTB	90% tumor response	KEGG_TYPE_I_DIABETES_MELLITUS	
GAPDH	complete blood cancer regression	KEGG_AUTOIMMUNE_THYROID_DISEASE	
HLA-DRA	aggressive solid tumors	WP_NETWORK_MAP_OF_SARSCOV2_SIGNALING_PATHWAY	
CD74	postoperative disease-free survival	WP_INTERACTIONS_OF_NATURAL_KILLER_CELLS_IN_PANCREATIC_CANCER	
HLA-DRB5	cytotoxic antitumor effects	HALLMARK_INTERFERON_GAMMA_RESPONSE	
NKG7	tumor burdens	REACTOME_MHC_CLASS_II_ANTIGEN_PRESENTATION	
GZMH	precision immunotherapy	WP_SARSCOV2_INNATE_IMMUNITY_EVASION_AND_CELLSPECIFIC_IMMUNE_RESPONSE	
CD52	proper organ function	KEGG_VIRAL_MYOCARDITIS	
IFNG	co-culture systems	REACTOME_INTERFERON_GAMMA_SIGNALING	
VCAM1	keratinocyte proliferation	WP_EBOLA_VIRUS_INFECTION_IN_HOST	
PRF1	early tertiary lymphoid structure	KEGG_CELL_ADHESION_MOLECULES_CAMS	
LAG3	chronic inflammation	REACTOME_RUNX1_AND_FOXP3_CONTROL_THE_DEVELOPMENT_OF_REGULATORY_T_LYMPHOCYTES_TREGS	
GZMB	coinhibitory receptors	REACTOME_CHEMOKINE_RECEPTORS_BIND_CHEMOKINES	
LYST	cellular immune activation	WP_CONTROL_OF_IMMUNE_TOLERANCE_BY_VASOACTIVE_INTESTINAL_PEPTIDE	
HAVCR2	disease susceptibility	KEGG_LEISHMANIA_INFECTION	
CTLA4	potent complement-dependent cytotoxic effects	REACTOME_COSTIMULATION_BY_THE_CD28_FAMILY	
DUSP4	bispecific antibody-mediated antitumour activity	WP_PLATELETMEDIATED_INTERACTIONS_WITH_VASCULAR_AND_CIRCULATING_CELLS	
ACP5	interferon regulatory factor 1 transcriptional activat	REACTOME_METABOLISM_OF_ANGIOTENSINOGEN_TO_ANGIOTENSINS	
SNAP47	specific cancer-related gene sets	HALLMARK_COMPLEMENT	
CXCR6	cancer therapy resistance	KEGG_HEMATOPOIETIC_CELL_LINEAGE	
CTSD	decreased tumor growth	WP_TCELL_ACTIVATION_SARSCOV2	
CD38	acidic substance-related gene sets	REACTOME_INTERFERON_SIGNALING	
CCL5	relapsed/refractory multiple myeloma	KEGG_ANTIGEN_PROCESSING_AND_PRESENTATION	
CHST12	exhaustion markers	WP_PATHOGENESIS_OF_SARSCOV2_MEDIATED_BY_NSP9NSP10_COMPLEX	
CXCL13	immune pathologies	WP_CYTOKINES_AND_INFLAMMATORY_RESPONSE	
GALNT2	ischemia reperfusion injury-related phenotypes	REACTOME_PD_1_SIGNALING	
	allotransplant rejection	KEGG_ASTHMA	
	progressive disease	KEGG_CYTOKINE_CYTOKINE_RECEPTOR_INTERACTION	
	cancer cell infection	WP_OVERVIEW_OF_PROINFLAMMATORY_AND_PROFIBROTIC_MEDIATORS	
	critical immune factors	REACTOME_SIGNALING_BY_INTERLEUKINS	
	ectopic accumulation	KEGG_NATURAL_KILLER_CELL_MEDIATED_CYTOTOXICITY	
	effector function inhibition	KEGG_SYSTEMIC_LUPUS_ERYTHEMATOSUS	
	durable tumor immunity	REACTOME_GENERATION_OF_SECOND_MESSENGER_MOLECULES	
	systemic immunotoxicity	WP_INTERACTIONS_BETWEEN_IMMUNE_CELLS_AND_MICRORNAS_IN_TUMOR_MICROENVIRONMENT	
	immune system balance	KEGG_INTESTINAL_IMMUNE_NETWORK_FOR_IGA_PRODUCTION	
	natural killer cell activity	WP_CHEMOKINE_SIGNALING_PATHWAY	
	cancer-related anorexia	WP_VITAMIN_B12_METABOLISM	
	melanoma metastases	WP_RANKLRANK_SIGNALING_PATHWAY	
	tertiary lymphoid structure-like lymphocytic aggreg	WP_TGFBETA_RECEPTOR_SIGNALING	
	severe infection	KEGG_CHEMOKINE_SIGNALING_PATHWAY	
	tumor tissue angiogenesis	WP_TCELL_ANTIGEN_RECEPTOR_TCR_PATHWAY_DURING_STAPHYLOCOCCUS_AUREUS_INFECTION	
	established signal transduction pathways	WP_TGFBETA_RECEPTOR_SIGNALING_IN_SKELETAL_DYSPLASIAS	
	anti-leukemia immunosurveillance	WP_MIRNAS_INVOLVEMENT_IN_THE_IMMUNE_RESPONSE_IN_SEPSIS	
	durable tumor remission	REACTOME_PEPTIDE_LIGAND_BINDING_RECEPTORS	
	efficacious suppression		
	strong inhibition		
	cardiac xenotransplantation		
	intra-tumoral pathological response		
	robust transduction		
	failed cancer cell elimination		
	rapid antitumor effects		
	cellular immunotherapeutics		
	portal inflammation		
	relapsed/refractory hematological malignancies		
	heightened cytokine production		
	immune-edited tumors		
	tumor-mediated immune escape		
	immune-mediated clearance		
	rational combination treatments		
	vivo antitumoral activity		
	disease-tolerance defenses		
	correlation immunotherapy response		
	adaptive immune checkpoint resistance		
	potent antitumor cytotoxicity		
	advanced cutaneous melanoma		
	treatment-associated tumor necrosis		
	previously known tumor antigens		
	tumor stages		
	hypoxia-associated immunosuppression		
	parietal cell loss		
	myeloma-related disorder		
	gene rearrangement		
	lasting tumor regression		
	complete eradication		
	antigen-specific pathogen recognition		
	tumor keratinocytes		
	lineage-specific gene programs		
	targeted single-gene perturbation		
	genetic circuits		
	luminal expansion		
	perihilar large duct type		
	costimulation-dependent dysfunctional programs		
	dysfunctional cells		
	higher tumor grades		
	shorter recurrence-free survival		
	response heterogeneity		
	dysregulated immune reactions		
	trained immunity induction		
	proinflammatory polarization		
	divergent cellular response		
	epigenetic reprogramming		
	cytotoxic immune responses		
	ordered immune hierarchies		
	adaptive cancer immunotherapy		
	intrahepatic cholangiocarcinoma		
	relapsed/refractory neuroblastoma		
	precancerous lesions		
	synovial sarcoma		
	pylori-induced gastritis		
	leukemia cell survival		
	local expansion		
	elicit epigenetic dysfunction		

Lung-Caushi	ICKG_annotation (ranked by pagerank score)	ORA_annotation	LLM_annotation
ZNF683	fulminant inflammatory bowel disease	HALLMARK_IL2_STAT5_SIGNALING	T-cell Activation and Immune Response Regulation (0.92)
GEM	complete tumor eradication	WP_ALLOGRAFT_REJECTION	
TOX2	90% tumor response	WP_CANCER_IMMUNOTHERAPY_BY_PD1_BLOCKADE	
BATF	postoperative disease-free survival	WP_T_CELL_RECEPTOR_AND_COSTIMULATORY_SIGNALING	
GBP5	coinhibitory receptors	WP_INTERACTIONS_BETWEEN_IMMUNE_CELLS_AND_MICRORNAS_IN_TUMOR_MICROENVIRONMENT	
MIR4435-2HG	advanced melanoma		
TNS3	tumor burdens		
GNA15	melanoma metastases		
CXCL13	critical acute myositis		
RBPJ	severe infection		
ENTPD1	pro-tumor activities		
LINC02195	tumor tissue angiogenesis		
GPR25	autoimmune myocarditis		
ITGAE	early tertiary lymphoid structure		
GNLY	greater tumor infiltration		
PDCD1	histone lysine methyltransferase		
CTLA4	chronic infection		
PRDM1	activation-induced cell death		
TNFRSF9	failed cancer cell elimination		
TIGIT	exhaustion markers		
HAVCR2	ischemia reperfusion injury-related phenotypes		
	dysregulated immune responses		
	effector function inhibition		
	immune system balance		
	autoimmune disease symptoms		
	ectopic accumulation		
	critical immune factors		
	tumor antigen		
	cytotoxic antitumor effects		
	immune checkpoint inhibitor		
	post-treatment relapse		
	relapsed/refractory acute myeloid leukemia		
	tertiary lymphoid structure-like lymphocytic aggregates		
	natural killer cell activity		
	systemic immunotoxicity		
	tumor heterogeneity		
	cellular proliferation		
	cytokine-mediated cancer immunotherapy		
	complete tumour regression		
	cancer-related anorexia		
	combination immunotherapy		
	intra-tumoral pathological response		
	established signal transduction pathways		
	cellular immune activation		
	strong inhibition		
	allotransplant rejection		
	cross-present melanoma-derived tumor antigens		
	longer progression-free survival		
	anti-inflammation effect		
	low-risk subgroups		
	initiates protective immune responses		
	cancer diagnosis		
	cardiac xenotransplantation		
	relapsed acute myeloid leukemia		
	transplant-related morbidity		
	anti-tumor function		
	systemic antitumor immunity		
	robust transduction		
	adaptive immune checkpoint resistance		
	tumor-mediated immune escape		
	alloimmune response		
	immune cell recruitment		
	important immune functions		
	natural anti-tumor immunity		
	treatment-associated tumor necrosis		
	disease-tolerance defenses		
	correlation immunotherapy response		
	local bystander activation		
	innate-like protection		
	innate-like memory		
	immune-edited tumors		
	lasting tumor regression		
	complete eradication		
	immune-mediated clearance		
	endogenous adaptive antitumour immunity		
	inhibitory checkpoint receptors		
	macrophage activation syndrome		
	post-ablation immune therapy		
	clinical remission		
	luminal expansion		
	tumor immune-microenvironment		
	transendothelial migration		
	parietal cell loss		
	recurrent clonal expansion		
	leukemia-like disease		
	significant tumor growth inhibition		
	anti-viral immune responses		
	peripheral angiogenesis		
	related plasma cell disorders		
	regulated cell death function		
	epigenetic reprogramming		
	cytotoxic immune responses		
	ordered immune hierarchies		
	adaptive cancer immunotherapy		
	tumor biomarker analyses		
	tumor microenvironments		
	relapsed/refractory neuroblastoma		
	immune-checkpoint blockade		
	tumor immune microenvironment phenotypes		
	immuno-oncology targets		
	antibody function		
	spontaneous regression		
	autoimmune reactions		
	tumor-infiltrating immunosuppressive leukocytes		
	excessive immune response		
	mixed inflammation		
	lysine-9 methyl-transferase		
	retrovirus immunity		
	ovarian pathogenesis		
	lymphocyte activatory receptors		
	leukemia cell survival		
	colitis-associated tumorigenesis		
	epithelial barrier function		
	epithelial cell expulsion		
	local inflammation		
	local chronic inflammation		
	serous borderline ovarian tumours		
	elicit epigenetic dysfunction		
	anti-tumor immune phenotypes		
	genetic modification		

Lowery	ICKG_annotation (ranked by pagerank score)	ORA_annotation	LLM_annotation
ATP10D	immunogenic cell death	HALLMARK_ALLOGRAFT_REJECTION	Immune Response and Cytotoxic Activity (0.85)
GZMB	vaccine-mediated antibody response	HALLMARK_INTERFERON_GAMMA_RESPONSE	
ENTPD1	cell-mediated immunity	REACTOME_PD_1_SIGNALING	
KIR2DL4	antigen-induced exhaustion	KEGG_ALLOGRAFT_REJECTION	
LAYN	heme oxygenase	REACTOME_GENERATION_OF_SECOND_MESSENGER_MOLECULES	
HTRA1	cell survival	KEGG_GRAFT_VERSUS_HOST_DISEASE	
CD70	melanoma cell ferroptosis	KEGG_TYPE_I_DIABETES_MELLITUS	
CXCR6	complete tumor eradication	KEGG_CELL_ADHESION_MOLECULES_CAMS	
HMOX1	sustained tumor control	WP_NETWORK_MAP_OF_SARSCOV2_SIGNALING_PATHWAY	
ADGRG1	subsequent effector differentiation	KEGG_ANTIGEN_PROCESSING_AND_PRESENTATION	
LRRN3	unfolded proteins	WP_ALLOGRAFT_REJECTION	
ACP5	leukemia-free survival	HALLMARK_IL2_STAT5_SIGNALING	
CTSW	immune-mediated anti-tumor immunity	KEGG_LEISHMANIA_INFECTION	
GALNT2	immune escape mechanism	KEGG_AUTOIMMUNE_THYROID_DISEASE	
LINC01480	immune-related pathways	REACTOME_COSTIMULATION_BY_THE_CD28_FAMILY	
CARS	long-term cell-mediated anti-tumor immunity	WP_PATHOGENESIS_OF_SARSCOV2_MEDIATED_BY_NSP9NSP10_COMPLEX	
LAG3	subsequent differentiation	REACTOME_NEUTROPHIL_DEGRANULATION	
TOX	anti-tumor efficacy	WP_EBOLA_VIRUS_INFECTION_IN_HOST	
PTPRCAP	immuno-metabolic regulation	WP_CANCER_IMMUNOTHERAPY_BY_PD1_BLOCKADE	
ASB2	tumour cell death	WP_MODULATORS_OF_TCR_SIGNALING_AND_T_CELL_ACTIVATION	
ITGB7	activation-induced cell death	REACTOME_PURINERGIC_SIGNALING_IN_LEISHMANIASIS_INFECTION	
PTMS	exhaustion markers	KEGG_HEMATOPOIETIC_CELL_LINEAGE	
CD8A	decreased antigen recognition ability	WP_INTERACTIONS_OF_NATURAL_KILLER_CELLS_IN_PANCREATIC_CANCER	
GPR68	cellular bioenergetics	KEGG_INTESTINAL_IMMUNE_NETWORK_FOR_IGA_PRODUCTION	
NSMCE1	antitumor response	KEGG_VIRAL_MYOCARDITIS	
ABI3	established immune responses	KEGG_ASTHMA	
SLC1A4	coinhibitory receptors	HALLMARK_INTERFERON_ALPHA_RESPONSE	
PLEKHF1	cytotoxic antitumor effects	REACTOME_SIGNALING_BY_INTERLEUKINS	
CD8B	advanced melanoma	KEGG_AMYOTROPHIC_LATERAL_SCLEROSIS_ALS	
LINC01871	epithelial regeneration	HALLMARK_INFLAMMATORY_RESPONSE	
CCL4	anti-tumour immune responses	REACTOME_INTERFERON_SIGNALING	
NKG7	anti-inflammation effect	WP_AMYOTROPHIC_LATERAL_SCLEROSIS_ALS	
CLIC3	abscopal anticancer activity	REACTOME_IMMUNOREGULATORY_INTERACTIONS_BETWEEN_A_LYMPHOID_AND_A_NON_LYMPHOID_CELL	
NDFIP2	melanoma metastases	REACTOME_MHC_CLASS_II_ANTIGEN_PRESENTATION	
PLPP1	proper organ function	HALLMARK_COMPLEMENT	
PCED1B	pro-tumor activities	WP_MECHANOREGULATION_AND_PATHOLOGY_OF_YAPTAZ_VIA_HIPPO_AND_NONHIPPO_MECHANISMS	
CXCL13	glioma cell population	WP_CYTOSOLIC_DNASENSING_PATHWAY	
PDCD1	severe infection	WP_INTEGRINMEDIATED_CELL_ADHESION	
PRF1	tumor tissue angiogenesis	KEGG_NATURAL_KILLER_CELL_MEDIATED_CYTOTOXICITY	
HLA-DMA	early tertiary lymphoid structure	REACTOME_TNFS_BIND_THEIR_PHYSIOLOGICAL_RECEPTORS	
GPR25	keratinocyte proliferation	WP_T_CELL_RECEPTOR_AND_COSTIMULATORY_SIGNALING	
CD9	intratumoral inflammation	KEGG_T_CELL_RECEPTOR_SIGNALING_PATHWAY	
TIGIT	neutrophil extracellular traps	KEGG_CYTOSOLIC_DNA_SENSING_PATHWAY	
HLA-DRB5	histone lysine methyltransferase	WP_INFLAMMATORY_RESPONSE_PATHWAY	
SYTL3	specific cancer-related gene sets	KEGG_CYTOKINE_CYTOKINE_RECEPTOR_INTERACTION	
SLF1	leukemia-like disease	WP_APOPTOSIS	
NEK1	immune-evasive viruses	HALLMARK_IL6_JAK_STAT3_SIGNALING	
CASP1	immune-evasive cancers	REACTOME_THE_NLRP3_INFLAMMASOME	
SMC4	immune pathologies	WP_TCELL_RECEPTOR_TCR_SIGNALING_PATHWAY	
TSEN54	failed cancer cell elimination	WP_TCELL_ANTIGEN_RECEPTOR_TCR_PATHWAY_DURING_STAPHYLOCOCCUS_AUREUS_INFECTION	
PLSCR1	acidic substance-related gene sets	REACTOME_TCR_SIGNALING	
GNPTAB	sensory ablation	REACTOME_DETOXIFICATION_OF_REACTIVE_OXYGEN_SPECIES	
HLA-DPB1	protective immune memory	HALLMARK_APOPTOSIS	
PLEKHA1	effective antitumor immune responses	REACTOME_INTERFERON_GAMMA_SIGNALING	
ARHGAP9	protective antitumor immune responses	WP_INTERACTIONS_BETWEEN_IMMUNE_CELLS_AND_MICRORNAS_IN_TUMOR_MICROENVIRONMENT	
ALOX5AP	denervation-dependent antitumor effects	REACTOME_INFLAMMASOMES	
SH3BP1	immune checkpoint inhibitor	REACTOME_INTERFERON_ALPHA_BETA_SIGNALING	
NCF4	relapsed/refractory multiple myeloma		
NELL2	allotransplant rejection		
GATA3	complete tumour regression		
PPM1M	lymphocyte fate determination		
TNFRSF1A	cancer-targeted photoimmunotherapy		
AC022706.1	cell-associated viral transcripts		
MCM5	active antitumoral immunity		
HLA-DRB1	immune system balance		
TNFSF10	human immunodeficiency virus		
TRIM21	higher mutation load		
HDLBP	microglial polarization		
ERN1	virus replication		
CALHM2	durable tumor remission		
SASH3	efficacious suppression		
ACTA2	immune-related gene signatures		
MAST4	autoimmune hepatitis		
CAPG	robust antitumor immunity		
MPST	local tissue protection		
IGFLR1	adaptive anti-tumor immunity		
GZMA	reduced terminal exhaustion		
CD27	increased memory cell characteristics		
ITGAE	endogenous adaptive antitumour immunity		
SLA2	dense tumor infiltration		
RHOC	sustained antitumor activity		
COMMD8	post allogeneic hematopoietic stem cell transplantation		
MYO1G	antiapoptotic genes		
SP140	post-ablation immune therapy		
PHPT1	cancer immunoprevention		
CD2BP2	monocyte differentiation		
PLEKHO1	organ-adapted protection		
STAM	autoimmune reactions		
MRPL16	tumor-infiltrating immunosuppressive leukocytes		
IL2RB	excessive immune response		
ID2	mixed inflammation		
TESPA1			
GOLGA8B			
MIS18BP1			
VAMP5			
DAPK2			
HLA-DPA1			
TSG101			
IL4R			
CCND2			
CTSC			
TRAF3IP3			
NLRC3			
ORAI3			
GNLY			
MIR155HG			
CARD16			
CD82			
ECH1			
JAML			
EEF1G			
ETFB			
DAXX			
RBM4			
HCST			
RAB27A			
YPEL2			
CHST12			
ARPC1B			
PDIA4			
PDIA6			
AC243960.1			
TBC1D10C			
PTPN6			
PYCARD			
BST2			
BTN3A2			
MTG1			
MLEC			
DUSP4			
GSDMD			
SLAMF1			
IFI6			
PCID2			
GIMAP1			
ITGA1			
CSNK2B			
CDK2AP2			
MYO1F			
AC004687.1			
PTTG1			
APOBEC3C			
TSPAN14			
MOB3A			
STXBP2			
LCP2			
PLA2G16			
LINC00649			
CST7			
TADA3			
SIT1			
APOBEC3G			
SUSD3			
CD3G			
CCL5			
CDC25B			
TNFRSF1B			
HMGN3			
THEMIS			
ASF1A			
CTNNB1			
FIBP			
CCDC85B			
POLR3GL			
GIMAP6			
ARL6IP1			
CALCOCO2			
CCPG1			
KLRB1			
ACAA2			
ISG15			
EIF4A1			
CAT			
MANF			
XAB2			
GRINA			
GLO1			
LSM2			
SLFN5			
FKBP1A			
AKNA			
TAP1			
LMO4			
APEH			
C12orf75			
TMEM14A			
DNPH1			
C17orf49			
NUDT5			
MGAT1			
CCDC69			
EIF4EBP1			
PDHB			
ARL3			
UCP2			
IFI35			
HSBP1			
LYST			
MRFAP1L1			
ITGAL			
AIP			
RASAL3			
CAPN1			
ITGB1			
RBPJ			
LBH			
DYNLL1			
NME2			
MT1F			
SYNGR2			
ABTB1			
ZGPAT			
CD63			
ILK			
SKA2			
TMEM204			
ACO2			
HOPX			
CRIP1			
OXNAD1			
CCS			
GRAP2			
GSTO1			
HADHB			
IL16			
PIN4			
CUEDC2			
CALM3			
SAMSN1			
HM13			
SNAP23			
LPCAT4			
FAAP20			
EFHD2			
PRDX3			
CCM2			
C22orf39			
SDHA			
ARRDC1			
MAP4K1			
NDUFA13			
IL27RA			
C14orf119			

Supplementary Tables. Each tab represents the enrichment result for a gene set. First column represents the genes in that gene set, second column represents the ICKG-based enrichment for gene sets, third column represents the significantly enriched annotations based on ORA, and fourth column represents the one term summarization provided by GPT4. Relevant enrichments are highlighted in red.

Supplementary Notes

These ICKGs possess broad potential that extends well beyond the scope of the present study. While the primary aim of our work was to establish the reliability of the graph construction methodology and demonstrate its application for immune gene set annotation, the framework is inherently versatile and can be adapted to a wide range of downstream analyses. In this supplementary note, we highlight several directions that we consider particularly promising and worth future explorations. These include (1) mechanistic reasoning to understand transcriptomic changes induced by gene perturbations, (2) discovery of intermediate signaling events to enable hypothesis generation, (3) enhanced interpretability by providing literature contexts through traceable edge metadata. Collectively, these potential extensions underscore the value of ICKGs not only as a knowledge integration tool, but also as a scalable foundation for future translational research in cancer immunology.

-- Mechanistic Reasoning --

In Figure 3, we have demonstrated that graph-based reasoning can be effectively executed using PageRank, yielding optimal AUC values for perturbation prediction. However, a key strength of these ICKGs is their verifiable connections among biomedical entities. By querying these ICKG subgraphs, we can also visualize the potential mechanisms driving the predicted transcriptomic shifts, thereby deepening our understanding of the graph-based reasoning process.

Through several examples, we illustrate the roles of transcription factors (TFs) in immune signaling across different immune cell types, further supporting the ICKGs from a perspective of biological interpretation, enabling researchers to explore and confirm biological hypotheses and interactions within the graph structure.

BLIMP1 (PRDM1) has been suggested to play a pivotal role in NK cell maturation[36,37]. Starting at *BLIMP1* using PageRank algorithm, we identified activated and inhibited genes downstream of *BLIMP1* in the NK cell KG: *BLIMP1* activates mature NK cell markers (*KLRG1, NKG2D*) while suppressing inflammatory mediators (*TNFA, CXCL10*) and cytotoxic molecules (*PFP, GZMB*) (**Supplementary Note 1a**). By reasoning through the corresponding subgraph, we saw that the activation of *BLIMP1* can result in a cascade of signaling through *EZH2*: activating epigenetic regulators (*HDAC10, H3K*) which in turn activate the *AP1* complex, a core transcriptional regulator for NK function. *EZH2* also suppresses the pro-inflammatory chemokine *CXCL10*, which can result in suppression of core inflammatory signaling molecules and cytotoxic genes (*IFNG, TNFA, GZMB, PFP*) (**Supplementary Note 1b-c**).

IKZF2 has been suggested to promote the accumulation of exhausted T cells in the tumor microenvironment by multiple groups[35,38–40]. Traversing our T cell KG starting at *IKZF2*, we

identified downstream activated and inhibited genes (**Supplementary Note 1d**). Among these activated genes are those associated with effector T cell inhibition and immunosuppressive environment (*FOXP3, MMP, PDCD1LG2*). We furthered queried the KG to examine the underlying signaling events that might have triggered these activations and found that *IKZF2* induces TCR expression, potentially intensifying TCR, which can result in chronic T-cell activation through greater TCR engagement (*TCRA, TCR-CDR3*) (**Supplementary Note 1e**). *IKZF2* had been experimentally perturbed in the T cell scCRISPR screen study[35], and using our T cell KG, we have recovered 92% of the DEGs from the experiment. In addition, we have also predicted an inhibition of *BATF3*, a gene critical for T cells survival and transition to memory[41] but was not captured by the DEGs, and by querying the graph, we see that this change is potentially driven by *CD3* inhibition by *IKZF2* (**Supplementary Note 1f**).

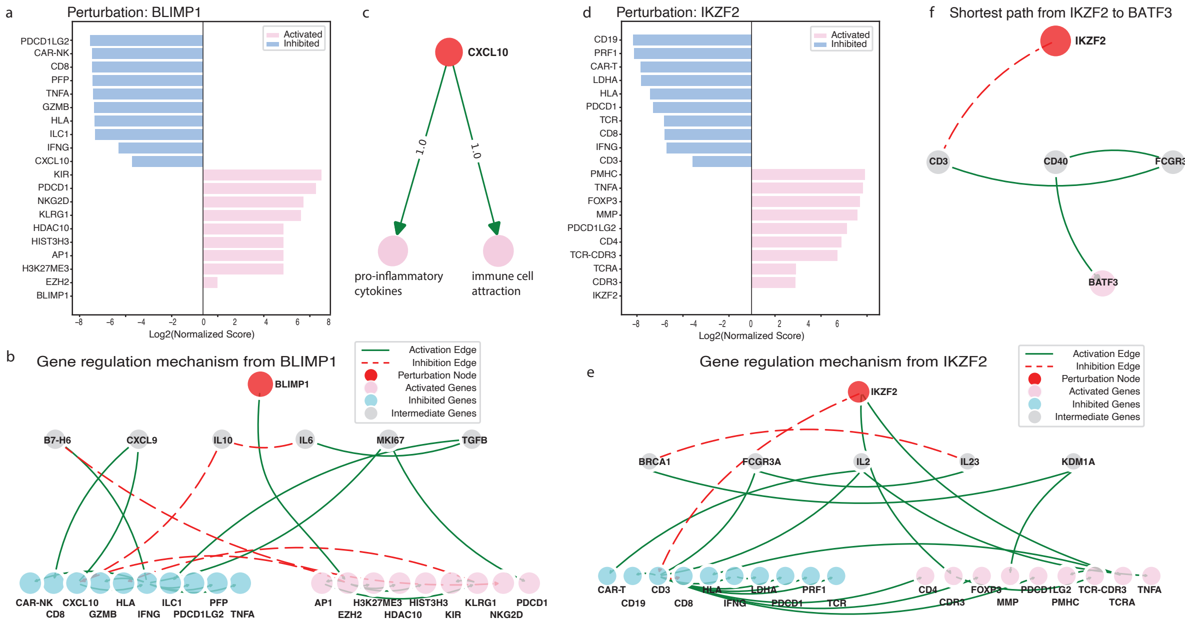

Supplementary Note 1. (a) PageRank ranked genes that are activate (pink) or inhibited (blue) by BLIMP1 in NK KG (b) Multiple mechanisms that BLIMP1 might contribute to the activation of inhibition of downstream genes in NK cell. (c) Two pathway nodes directed activated by CXCL10. (d) PageRank ranked genes that are activate (red) or inhibited (blue) by IKZF2 in T KG. (e) Multiple mechanisms that IKZF2 might contribute to the activation of inhibition of downstream genes in T cell. (f) Shortest path from IKZF2 to BATF3 in the T cell KG.

Reasoning through both B and macrophage ICKG, we also identified signaling events driven by key genes. *BCL6* had been suggested to play crucial role in regulating the formation of germinal center B cells[42,43]. Reasoning through B ICKG, we have identified top activated genes related to cell-growth, metabolism and BCR activation (*MTOR*, *PIK3CG*, *C3, C5A*). Among the top inhibited genes, *BCL6* suppresses *BLIMP1* (a master regulator of plasma cell differentiation[44]), immunoglobulins (*IGD, IGA*), and cytoskeletal regulator *NMMHCIIA*[45] (**Supplementary Note 2a**). These regulatory patterns align with *BCL6's* known function in maintaining B cell identity in germinal center while preventing premature differentiation into antibody-secreting plasma cells. We furthered queried the KG to examine the connections between *BCL6* and these target genes

and found that, although there are many indirect connections, *BCL6* is capable of directly impacting these target genes. (**Supplementary Note 2b**). *SPP1* has been linked with immunosuppressive macrophages[46,47]. Reasoning through macrophage ICKG, we have identified multiple activated and inhibited genes (**Supplementary Note 2c**), including many well-known immunosuppressive genes, such as *CSF1, CLDN1*, and *CTNNB1* that are activated by *SPP1*, and proinflammatory genes, such as *CD47, CD4*, and *FCGR3A*, that are inhibited by *SPP1*. We further queried the KG to examine the potential mechanism and found that SPP1 can directly activate many of the immunosuppressive genes while the inhibition of proinflammation is partially mediated by *CSF2,* and *CD163* (**Supplementary Note 2d**).

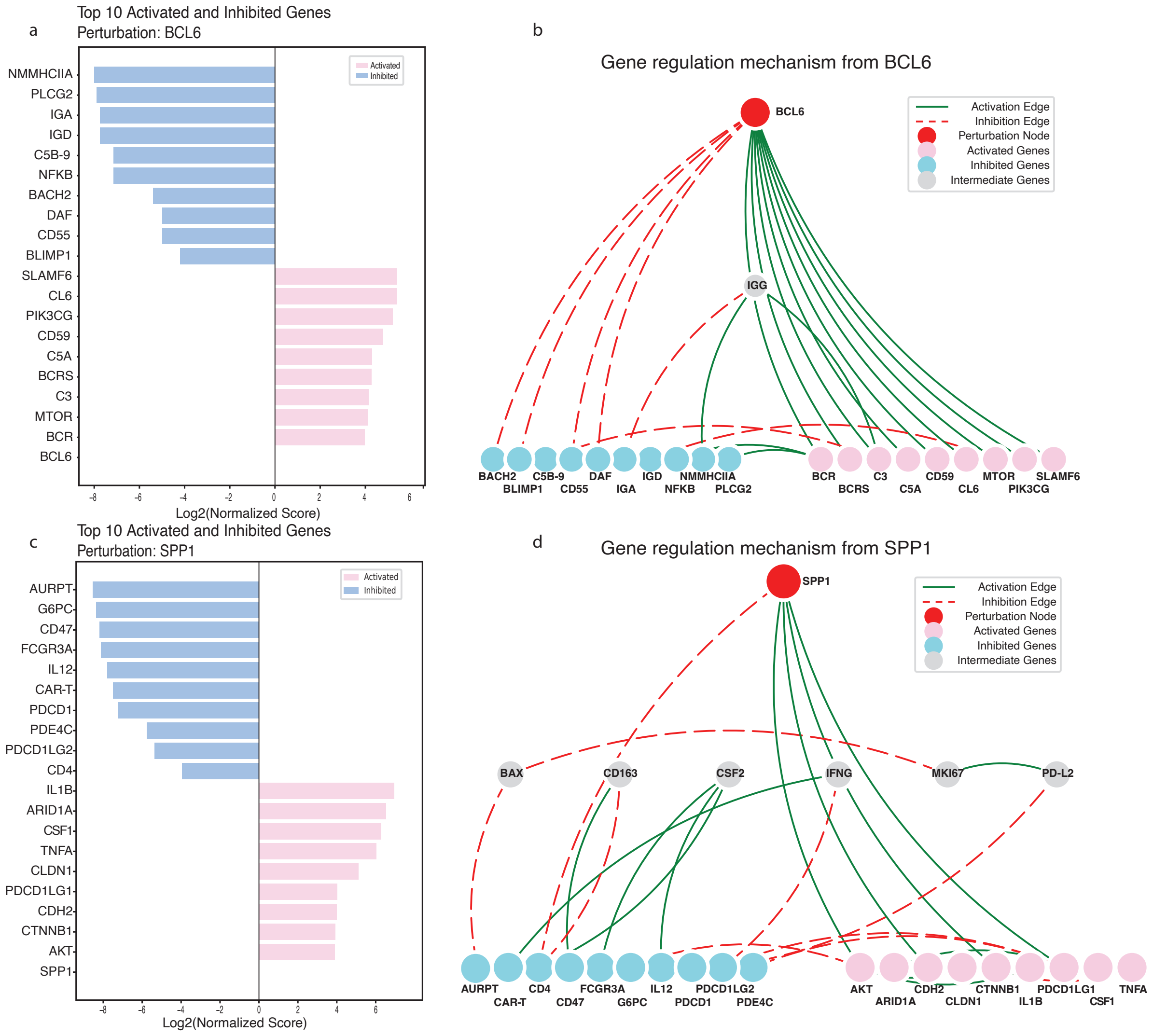

Supplementary Note 2. (a) PageRank ranked genes that are activate (pink) or inhibited (blue) by BCL6 in B KG (b) Multiple mechanisms that BCL6 might contribute to the activation of inhibition of downstream genes in B cell. (c) PageRank ranked genes that are activate (red) or inhibited (blue) by SPP1 in Macrophage KG. (d) Multiple mechanisms that SPP1 might contribute to the activation of inhibition of downstream genes in macrophages.

-- Hypothesis Generation --

As ICKGs contain directed links between genes and phenotype, ICKGs also enable efficient reasoning to uncover how specific genes within a gene set contribute to their associated phenotypes. By tracing the underlying network structure, ICKGs can also nominate gene sets related to a phenotype of interest, thereby highlighting intermediate genes and context-specific interactions. This capacity to move beyond static annotations and reveal mechanistic links provides a framework for hypothesis generation, allowing researchers to propose and test novel pathways or gene–phenotype relationships suggested by the graph.

For example, tumor antigen specific T cells are one of the key players in combating malignancies[58,59], hence detecting them and tracing their trajectories to dysfunction can elucidate new mechanisms of intervention. Lowery et al had mapped NeoTCR clonotypes from metastatic tumors to single-cell transcriptomes and identified signatures of neoantigen-specific tumor-infiltrating lymphocytes[51]. We mapped the signatures onto T cell KG, and observed that this gene set is enriched in "antigen-induced exhaustion", "vaccine-mediated antibody response" and "complete tumor eradication", which are strong and specific languages to depict the function of this gene set. We then mapped "antigen-induced exhaustion" on the KG and performed PageRank to find the most relevant genes among the gene set that lead to this phenotype (**Supplementary Note 3a**). By traversing the KG via PageRank, we unraveled the intermediate signaling events between each gene to the phenotype and found multiple pathways that lead to antigen-induced exhaustion (**Supplementary Note 3b**). Despite having multiple routes, the shortest pathway to antigen-induced exhaustion ultimately converges through CD70. Knowing that CD70 is one of the key genes associated with exhaustion, immunologists can design experiments that target CD70 to prevent T cell overactivation or reverse T cell exhaustion and potentially improve T cell killing capacity. Importantly, the ICKGs enable precise tracing of the exact abstracts and studies from which this mechanism was inferred from, allow human verification of the hypothesis before it is pursued experimentally.

Beside T cells, B cells also contribute to immune responses through antibody production and antigen presentation in the tumor microenvironment[61,62], while germinal center (GC) B cells, a specialized subset, further refine this response by undergoing affinity maturation and facilitating T cell recruitment[63–65]. Therefore, we leveraged our B cell KG to identify genes associated with GC B cells formation. By reasoning on the ICKG, we identified genes that are highly relevant to GC B cell formation (**Supplementary Note 3c**), particularly in supporting proliferation, immune signaling, and cell interactions. The network highlights multiple pathways driving GC B cell formation, with MYC, BCL6, and CXCR5 as central regulators[66,67]. These factors coordinate B cell proliferation, positioning, and differentiation within germinal centers. TNF signaling and CD40/CD40L interactions further support B cell activation[68], while IG genes enable antibody class switching. Metabolic regulators like MTOR and PHGDH provide energy for active cells[69,70], and

inhibitory pathways (e.g., DDX3X) maintain balance to prevent overactivation. Together, these pathways enable germinal centers to produce high-affinity B cells crucial for adaptive immunity, fostering the environment for germinal center B cell maturation and activity (**Supplementary Note 3d**).

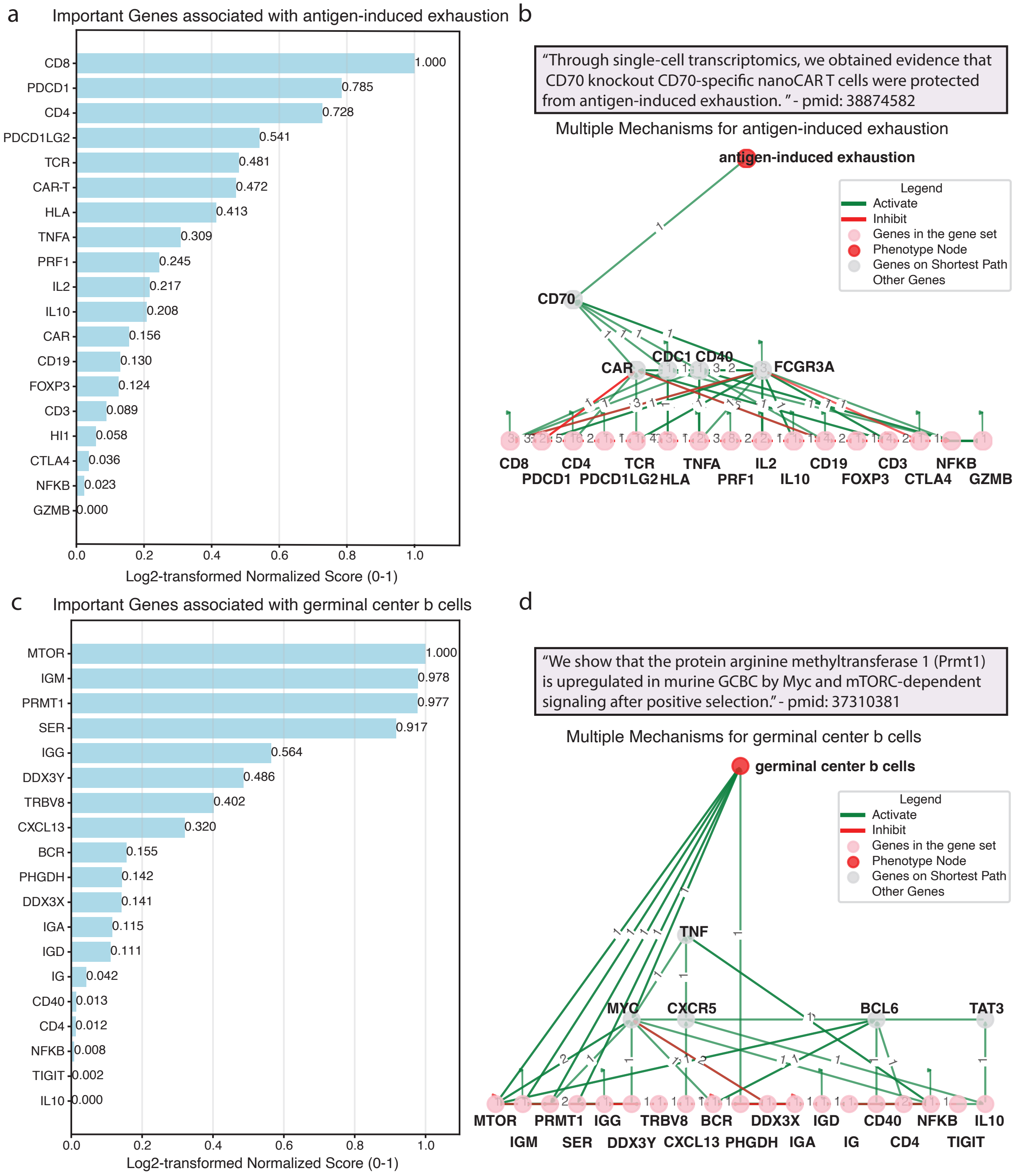

Supplementary Note 3. (a) PageRank ranked genes that are associated with the node "antigen-induced exhaustion". (b) Multiple mechanisms that selected genes in the tumor-reactive T cell signatures are contributing to antigen-induced exhaustion. Genes colored in pink are genes included in the gene set and genes in grey represents intermediate signaling events. (c) PageRank ranked

genes that are associated with the node "germinal center b cells" and the exact sentence from which the relationship is inferred. (d) Multiple mechanisms that selected genes are contributing to the formation of germinal center b cells and the exact sentence from which the relationship is inferred. The texts on top of (b) and (d) are the exact texts from the source publication from which the last edge relationship is inferred.

---------------------------------- Interpretability via traceable contexts --------------------------------z

Both above-mentioned applications allow users to query and visualize subgraphs of interest, along with edge-level metadata that links each relationship to the exact publication from which it was inferred. This design enhances human interpretability by providing contextual evidence for each connection, enabling researchers to assess not only the existence of a relationship but also its biological and experimental basis. Unlike most of the LLM approaches with black-box models that offer predictions without transparent justification or reasoning steps, ICKGs maintain traceability at every step, thereby facilitating "human-in-the-loop" verification, fostering trust, interpretability, and reproducibility for downstream analyses.
